# Global, Regional, and National Consumption of Sugar-Sweetened Beverages, Fruit Juices, and Milk: A Systematic Assessment of Beverage Intake in 187 Countries

**DOI:** 10.1371/journal.pone.0124845

**Published:** 2015-08-05

**Authors:** Gitanjali M. Singh, Renata Micha, Shahab Khatibzadeh, Peilin Shi, Stephen Lim, Kathryn G. Andrews, Rebecca E. Engell, Majid Ezzati, Dariush Mozaffarian, Global Burden of Diseases Nutrition and Chronic Diseases Expert Group (NutriCoDE)

**Affiliations:** 1 Friedman School of Nutrition Science and Health Policy, Tufts University, Boston, MA, United States of America; 2 Department of Epidemiology, Harvard School of Public Health, Boston, MA, United States of America; 3 Institute of Health Metrics and Evaluation, Seattle, WA, United States of America; 4 Department of Global Environmental Health, School of Public Health, Imperial College London, London, United Kingdom; 5 Department of Food Science and Human Nutrition, Agricultural University of Athens, Athens, Greece; University of East Anglia, UNITED KINGDOM

## Abstract

**Background:**

Sugar-sweetened beverages (SSBs), fruit juice, and milk are components of diet of major public health interest. To-date, assessment of their global distributions and health impacts has been limited by insufficient comparable and reliable data by country, age, and sex.

**Objective:**

To quantify global, regional, and national levels of SSB, fruit juice, and milk intake by age and sex in adults over age 20 in 2010.

**Methods:**

We identified, obtained, and assessed data on intakes of these beverages in adults, by age and sex, from 193 nationally- or subnationally-representative diet surveys worldwide, representing over half the world’s population. We also extracted data relevant to milk, fruit juice, and SSB availability for 187 countries from annual food balance information collected by the United Nations Food and Agriculture Organization. We developed a hierarchical Bayesian model to account for measurement incomparability, study representativeness, and sampling and modeling uncertainty, and to combine and harmonize nationally representative dietary survey data and food availability data.

**Results:**

In 2010, global average intakes were 0.58 (95%UI: 0.37, 0.89) 8 oz servings/day for SSBs, 0.16 (0.10, 0.26) for fruit juice, and 0.57 (0.39, 0.83) for milk. There was significant heterogeneity in consumption of each beverage by region and age. Intakes of SSB were highest in the Caribbean (1.9 servings/day; 1.2, 3.0); fruit juice consumption was highest in Australia and New Zealand (0.66; 0.35, 1.13); and milk intake was highest in Central Latin America and parts of Europe (1.06; 0.68, 1.59). Intakes of all three beverages were lowest in East Asia and Oceania. Globally and within regions, SSB consumption was highest in younger adults; fruit juice consumption showed little relation with age; and milk intakes were highest in older adults.

**Conclusions:**

Our analysis highlights the enormous spectrum of beverage intakes worldwide, by country, age, and sex. These data are valuable for highlighting gaps in dietary surveillance, determining the impacts of these beverages on global health, and targeting dietary policy.

## Introduction

Sugar-sweetened beverages (SSBs), fruit juice, and milk are components of diet that substantially affect health. SSB intake has been linked with weight gain, diabetes, metabolic syndrome, and dental caries [1–8]. Although moderate consumption of fruit juice may be an important source of vitamins, minerals, and antioxidants, excessive fruit juice consumption has been associated with weight gain and development of dental caries [9–11]. Milk is an important source of vitamin D, calcium, protein, and calories especially in children and the elderly, while high intakes have been linked with incidence of prostate cancer [12,13].

Although beverage consumption substantially impacts health, few nationally-representative studies on dietary intakes of SSBs, fruit juice, and milk are publically available [14–18]. Of the existing data published on beverage consumption in countries worldwide, no study has yet assessed global geographic, age, or time trends comprehensively such that both within- and between-country comparisons can be made. This paucity of comprehensive global estimates limits the ability to assess the effects of beverage intakes on disease burdens and hinders evaluation of dietary policies and interventions worldwide. Moreover, since age and sex are major determinants of dietary patterns and their effects on disease, comparable information on variation in SSB, fruit juice, and milk consumption by age and sex is vital for effectively targeting health policy within countries.

To comparably quantify levels of consumption of these major non-alcoholic caloric beverages, we systematically reviewed, compiled, and extracted national, mostly individual-level data on the consumption of SSBs, fruit juice, and milk, from countries around the world. We similarly assessed data on levels of calcium consumption worldwide, given its relevance to milk intake. In addition, we extracted and assessed data relevant to SSB, fruit juice, and milk annual availability in 187 countries from the United Nations Food and Agriculture Organization (FAO) food balance database [19]. We developed and applied statistical methods to address data comparability and missingess in the beverage intake survey data, to combine individual-level beverage intake data with country-level beverage availability data, and to quantify the combined uncertainty from all data sources. We report comprehensive and comparable estimates of global, regional, and national consumption patterns of SSBs, fruit juices, and milk, by age and sex, in high, middle, and low-income countries.

## Methods

This work was performed by the Nutrition and Chronic Diseases Expert Group (NutriCoDE) as part of the 2010 Global Burden of Diseases, Injuries, and Risk Factors (GBD) Study [20,21]. Our methods for identification, access, and selection of dietary risk factors and data have been reported elsewhere [22–24]. Briefly, we performed systematic searches to identify survey microdata on age- and sex-specific intakes of SSBs, fruit juices, and milk from countries around the world between 1990 and 2010. We assessed the sampling methods and diet assessment methods of all surveys identified, and included only those with nationally- or subnationally-representative samples and valid diet assessment methods. We extracted data from survey microdata using standardized methods to ensure comparability across surveys. In addition, we retrieved, assessed, and extracted annual data relevant to SSB, fruit juice, and milk availability in each of 187 countries from FAO food availability data collected annually between 1990 and 2010. Finally, we used hierarchical statistical modeling methods to combine and harmonize food availability data with dietary intake data, to account for different survey sampling methodologies and diet assessment methods, to estimate missing dietary intake data in data-sparse countries, and to capture sampling and modeling uncertainty. Using these methods, we were able to quantify consumption levels of SSBs, fruit juices, and milk by age and sex in 187 countries worldwide over at 20 year period. Given its relevance to milk intake, calcium was also assessed worldwide.

### Identification of national beverage data

Between Mar 2008 and Sep 2010 we identified survey data on SSB, fruit juice, and milk intake in adults (≥20 years of age), through systematic searches of multiple literature databases including MEDLINE, Embase, CAB abstracts, WHOLIST (WHO library), and SIGLE (grey literature database), hand-searches of reference lists, and direct contact with authors [22]. We defined the beverage categories to correspond as closely as possible to definitions used in published meta-analyses of their health effects [6,10,12], as well as to incorporate definitions used in survey data that we collected from around the world. Specifically, we defined SSBs as sugar-sweetened beverages containing over 50 kcal/8oz serving, including sodas, fruit drinks, sports/energy drinks, pre-sweetened iced tea, and homemade sugar-sweetened beverages such as frescas. Fruit juices were defined as beverages containing 100% fruit or vegetable juice with no added sweeteners. Milk included both skim, lowfat, and whole milk and other dairy drinks. Calcium intake was assessed as total dietary intake of calcium, excluding supplements.

Our direct contact with experts worldwide (Corresponding Members of NutriCoDE) proved to be the most fruitful data source and we developed a protocol and timeline for requesting primary de-identified (fully anonymized) data in a standardized format. Surveys were included if they were from a population-based sample (e.g. primarily nationally- or subnationally-representative populations with no evidence of selection bias) and if the data were based on a standard diet assessment tool. If no individual-level survey data on dietary intakes could be identified for a country, we evaluated other sources of data, such as household consumption and expenditure surveys [22].

We also identified country-level data relevant to SSB, fruit juice, and milk availability in all 187 nations of interest using food balance data collected by the FAO for every year between 1980 and 2010. These food balance data measure the total per-capita annual availability of particular foods for human consumption, taking into consideration agricultural production, imports, and exports in each country. While the FAO does collect information on milk availability in all 187 countries, it does not collect data on the availability of SSBs or fruit juices. Since SSBs comprise a major source of dietary added sugars [5,25,26] we used FAO total sugar availability data as a proxy for information on SSB country-level availability. Similarly, since roughly 40% of fresh fruit available per capita is converted into fruit juice[27], we used FAO fruit availability data as a proxy for information on fruit juice availability in all 187 countries.

### Extraction and standardization of beverage data

We used standardized data retrieval methods which have been described elsewhere [21,23,24]. From each dietary survey obtained from Corresponding Members, published literature, or other sources, we extracted data on mean and standard deviation of intake of SSBs, fruit juice, and milk, as well as information on survey location, time period, representativeness, sampling design, and sample size into a standardized electronic extraction spreadsheet. We extracted uniform metrics and units of beverage intake from all surveys to the extent possible. We assessed data plausibility and checked for extraction errors. Quality of diet assessment methods used in each survey were assessed using methods described previously [22]. We used consistent methods to analyze and aggregate all survey data to ensure comparability across surveys. For nationally-representative survey microdata, we included sampling weights, primary sampling unit, and stratum in our analyses, when available. In surveys employing multiple 24 hr diet recalls, we quantified mean intakes by averaging all days of dietary assessment (usually 2 days), and we used a corrected population standard deviation (SD) to account for within- versus between-person variation [28]. Dietary intakes were standardized using the residual method [28] to 2000 kcal/d, thereby producing more comparable estimates across age, sex, and country.

### Pooling and statistical analysis of beverage data

Although we used systematic data retrieval and standardization methods as described above, the data we extracted from national/subnational surveys of individual-level dietary intakes were not always comparable, varying in representativeness, urban or rural coverage, age groups, dietary instruments, or dietary metrics. Furthermore, the data we extracted from FAO food balance sheets accounted for overall availability of the beverages of interest at the country level and not individual-level consumption of these beverages. To combine individual-level beverage intake data with country-level beverage availability data, to address issues of data incomparability, and to capture the uncertainty in estimates of beverage intake due to measurement error, sampling uncertainty, and modeling uncertainty, we used established age-integrating Bayesian hierarchical modeling methods [23,24,29]. This model estimated the mean consumption of each beverage and its uncertainty for each age-sex-country-year subgroup. Importantly, the model harmonized FAO food availability data with intake data in countries that had both types of data and calibrated FAO data to more closely approximate intake data in countries that had only availability data[30]. For each beverage, the primary model inputs were survey-level quantitative data, including country-, time-, age-, and sex- specific consumption levels; data on the numbers of subjects in each stratum; survey-level indicator covariates for sampling representativeness, dietary assessment method, and type of dietary metric; country-level year-specific data relevant to availability of that beverage from the FAO; and country, region (21 regions), and super-region (7 groupings of regions) random effects.

Additional details about the model are presented in Appendix A in S1 File. To ensure that pooled estimates give weight to the best available data, the model included additional offset and variance components to account for differences between national vs. subnational surveys, individual-level vs. household-level dietary data, primary vs. secondary dietary metrics, and optimal vs. suboptimal dietary assessment methods in each case giving greater weight to the preferred characteristic. Models were fit using a randomized Markov Chain Monte Carlo (MCMC) algorithm based on the Adaptive Metropolis step function. Models were assessed for convergence of MCMC iterations and validated using goodness-of-fit tests as described elsewhere [23,24,29]. Qualitative evaluation of model estimates for beverage consumption levels was also conducted by comparing the estimates with known high-quality data and by contacting subject-matter experts to assess the plausibility of model estimates.

Our modeling approach quantified uncertainty in beverage consumption estimates as completely as possible, including sampling and measurement uncertainty in dietary intakes in national survey data, uncertainty associated with suboptimal metrics, subnational samples, or household-level surveys, and uncertainty associated with incomplete global coverage of national individual-level survey data. To quantify the combined uncertainty from the aforementioned sources, we used Monte Carlo simulations, drawing 1,000 times from the posterior distribution of each exposure for each age, sex, country, and year. We computed the mean exposure from the 1000 draws, and the 95% uncertainty intervals were calculated as the 2.5th and 97.5th percentiles of the 1000 draws. Absolute and relative difference in exposure between 1990 and 2010 was calculated at the draw level to account for the full spectrum of uncertainty. Using these methods, we quantified the consumption levels (mean and uncertainty intervals) of SSBs, milk, and fruit juice among men and women in seven age groups in 187 countries in 1990 and 2010.

In addition, we analyzed patterns in beverage consumption stratified by BMI and country income level. We analyzed the cross-country age- and sex-specific correlations of the three beverages with country-, age-, and sex-specific BMI data obtained through the Global Burden of Diseases, Injuries and Risk Factors 2010 Study [31]. Country income-level classifications were obtained from the World Bank, and were developed using the World Bank Atlas method [32]: low-income countries are those with per-capita gross national income (GNI) (in U.S. dollars) ≤ $1045 in 2013; middle-income countries have per-capita GNI > $1045 and < $12,746; high-income countries are those with per-capita GNI per capita ≥ $12,746. Lower-middle-income and upper-middle-income economies are separated at per-capita GNI of $4,125. All analyses were performed in Python or R.

## Results

Global survey data on individual-level SSB consumption were derived from 62 surveys, including 51 countries and 612,000 individuals, and representing 63% of the world’s adult population (Table 1). 88.2% of the survey data on SSB consumption were nationally-representative, and 72.1% were from low- and middle-income countries. Data on fruit juice intake were from 56 surveys, 86.7% of which were nationally-representative. These data on fruit juice consumption included information on 569,000 individuals from 46 countries worldwide, representing 58% of the world’s population. Milk consumption data included 75 surveys worldwide, based on information from 77 countries and 689,000 individuals, with 79.2% of the data representative at the national level and 53.4% of the data from low- and middle-income countries. Country-level FAO data relevant to the annual availability of each of the three beverages were available for all 187 countries in our analysis for every year between 1980 and 2010 (Table 1).

**Table 1:** Availability and characteristics of individual-level dietary survey data and U.N. Food and Agriculture Organization (FAO) food availability data used to estimate sugar-sweetened beverage, fruit juice, and milk consumption levels by world region.

Region^1^	Adult population (millions)		Sugar-sweetened beverages		Fruit juice		Milk	
			Individual-level dietary survey data	FAO country-level food availability data^2^	Individual-level dietary survey data	FAO country-level food availability data^3^	Individual-level dietary survey data	FAO country-level food availability data^4^
**East Asia,Southeast Asia,and South Asia**	2146	**Number of surveys(% nationally representative)**	12 (75.0)	22	9 (77.8)	22	19 (57.9)	22
		**Time period of data**	1995–2008	1980–2010	1995–2008	1980–2010	1991–2008	1980–2010
		**Total sample size**	302,292	NA^5^	360,909	NA	375,657	NA
**Central Asia, Eastern Europe, and Central Europe**	273	**Number of surveys (% nationally representative)**	4 (75.0)	29	6 (66.7)	29	11 (72.7)	29
		**Time period of data**	2000–2005	1980–2010	1993–2005	1980–2010	1993–2005	1980–2010
		**Total sample size**	12,977	NA	18,095	NA	28,645	NA
**Western Europe**	301	**Number of surveys(% nationally representative)**	32 (90.6)	23	31 (93.5)	23	31 (93.5)	23
		**Time period of data**	1986–2009	1980–2010	1986–2009	1980–2010	1986–2009	1980–2010
		**Total sample size**	94,408	NA	92,698	NA	93,624	NA
**North Africa and the Middle East**	225	**Number of surveys (% nationally representative)**	3 (66.7)	19	1 (100)	19	3 (66.7)	19
		**Time period of data**	2005–2009	1980–2010	2008–2009	1980–2010	2005–2009	1980–2010
		**Total sample size**	76,790	NA	2,592	NA	73,798	NA
**Sub-Saharan Africa**	320	**Number of surveys(% nationally representative)**	1 (100)	65	1 (100)	65	2 (50)	65
		**Time period of data**	1995	1980–2010	1995	1980–2010	1995	1980–2010
		**Total sample size**	1,502	NA	1,502	NA	1,699	NA
**Latin America and the Caribbean**	319	**Number of surveys(% nationally representative)**	5 (80)	37	3 (66.7)	37	4 (75.0)	37
		**Time period of data**	1993–2007	1980–2010	1993–2007	1980–2010	2003–2006	1980–2010
		**Total sample size**	32,282	NA	1371	NA	23,438	NA
**U.S. and Canada**	226	**Number of surveys(% nationally representative)**	2 (100)	2	2 (100)	2	2 (100)	2
		**Time period of data**	1990–2006	1980–2010	1990–2006	1980–2010	1990–2006	1980–2010
		**Total sample size**	70,011	NA	70,011	NA	70,011	NA
**Australia and New Zealand**	17.4	**Number of surveys(% nationally representative)**	3 (100)	2	3 (100)	2	3 (100)	2
		**Time period of data**	1995–2002	1980–2010	1995–2002	1980–2010	1995–2002	1980–2010
		**Total sample size**	21,709	NA	21,709	NA	21,709	NA
**Globe**	3827.4	**Number of surveys(% nationally representative)**	62 (88.2)	187	56 (86.7)	187	75 (79.2)	187
		**Time period of data**	1986–2009	1980–2010	1986–2009	1980–2010	1986–2009	1980–2010
		**Total sample size**	611,971	NA	568,887	NA	688,581	NA

^1^ The countries in each region are as follows: **East Asia, Southeast Asia, and South Asia—** East Asia: China, Hong Kong SAR (China), Macau SAR (China), Democratic People's Republic of Korea, Taiwan. Southeast Asia: Cambodia, Indonesia, Lao People's Democratic Republic, Malaysia, Maldives, Myanmar, Philippines, Sri Lanka, Thailand, Timor-Leste, Viet Nam. South Asia: Afghanistan, Bangladesh, Bhutan, India, Nepal, Pakistan Oceania: Cook Islands, Fiji, French Polynesia, Kiribati, Marshall Islands, Micronesia (Federated States of), Nauru, Palau, Papua New Guinea, Samoa, Solomon Islands, Tonga, Vanuatu **Central Asia, Eastern Europe, and Central Europe—** Central Asia: Armenia, Azerbaijan, Georgia, Kazakhstan, Kyrgyzstan, Mongolia, Tajikistan, Turkmenistan, Uzbekistan Eastern Europe: Belarus, Estonia, Latvia, Lithuania, Moldova, Russian Federation, Ukraine Central Europe: Albania, Bosnia and Herzegovina, Bulgaria, Croatia, Czech Republic, Hungary, Montenegro, Poland, Romania, Serbia, Slovakia, Slovenia, Macedonia (Former Yugoslav Republic of) **Western Europe—** Andorra, Austria, Belgium, Cyprus, Denmark, Finland, France, Germany, Greece, Greenland, Iceland, Ireland, Israel, Italy, Luxembourg, Malta, Netherlands, Norway, Portugal, Spain, Sweden, Switzerland, United Kingdom **North Africa and the Middle East—** Algeria, Bahrain, Egypt, Iran (Islamic Republic of), Iraq, Jordan, Kuwait, Lebanon, Libyan Arab Jamahiriya, Morocco, Occupied Palestinian Territory, Oman, Qatar, Saudi Arabia, Syrian Arab Republic, Tunisia, Turkey, United Arab Emirates, Yemen **Sub-Saharan Africa—** Central Africa: Angola, Central African Republic, Congo, Democratic Republic of the Congo, Equatorial Guinea, Gabon, East Africa Burundi, Comoros, Djibouti, Eritrea, Ethiopia, Kenya, Madagascar, Malawi, Mauritius, Mozambique, Rwanda, Seychelles, Somalia, Sudan, Uganda, United Republic of Tanzania, Zambia East Africa: Burundi, Comoros, Djibouti, Eritrea, Ethiopia, Kenya, Madagascar, Malawi, Mauritius, Mozambique, Rwanda, Seychelles, Somalia, Sudan, Uganda, United Republic of Tanzania, Zambia West Africa: Benin, Burkina Faso, Cameroon, Cape Verde, Chad, Côte d'Ivoire, Gambia, Ghana, Guinea, Guinea-Bissau, Liberia, Mali, Mauritania, Niger, Nigeria, Senegal, Sierra Leone, São Tomé and Príncipe, Togo Southern Africa: Botswana, Lesotho, Namibia, South Africa, Swaziland, Zimbabwe **Latin America and the Caribbean—** Andean Latin America: Bolivia, Ecuador, Peru Central Latin America: Colombia, Costa Rica, El Salvador, Guatemala, Honduras, Mexico, Nicaragua, Panama, Venezuela Southern Latin America: Argentina, Chile, Uruguay Tropical Latin America: Brazil, Paraguay Caribbean: Antigua and Barbuda, Bahamas, Barbados, Belize, Bermuda, British Virgin Islands, Cuba, Dominica, Dominican Republic, Grenada, Guyana, Haiti, Jamaica, Netherlands Antilles, Puerto Rico, Saint Kitts and Nevis, Saint Lucia, Saint Vincent and the Grenadines, Suriname, Trinidad and Tobago **U.S. and Canada—** United States of America, Canada **Australia and New Zealand—** Australia, New Zealand

^2^ The U.N. Food and Agriculture Organization (FAO) did not collect country-level data on sugar-sweetened beverage availability, per se, in the time period 1980–2010, so as a proxy we used FAO data on the total availability of sugar in the 187 countries of interest. FAO availability data captures the net availability of particular food items for human consumption in a given country and year, accounting for imports, exports, and agricultural production.

^3^The FAO did not collect country-level data on fruit juice availability, per se, in the time period 1980–2010, so as a proxy we used FAO data on the total availability of fruits in the 187 countries of interest.

^4^FAO availability data for milk captures the net availability of milk for human consumption in a given country and year, accounting for imports, exports, and agricultural production.

^5^As FAO food balance data represents country-level availability of food, sample size is not relevant to this type of data and is listed as "NA" (not applicable) for all FAO data in the table above.

### Global distribution of sugar-sweetened beverage consumption

In 2010, global SSB consumption in adults over age 20 averaged 0.58 (95%UI: 0.37, 0.89) 8 oz servings/day (Table 2). SSB consumption was highest in men aged 20–39 (1.04, 95%UI: 0.63, 1.7 servings/day), and lowest in women aged 60 and over (0.34, 95%UI: 0.20, 0.53 servings/day). In general, SSB consumption was higher in upper-middle income countries (0.80, 95%UI: 0.51, 1.22 servings/day) and lower-middle income countries (0.59, 95%UI: 0.34, 0.95 servings/day) than in high income (0.51, 95%UI: 0.37, 0.71 servings/day) or low income (0.35, 95%UI: 0.20, 0.56 servings/day) countries.

**Table 2:** Mean consumption of SSBs, fruit juice, milk, and calcium by age, sex, and country income level.

	Mean (95%UI) of beverage intakes			
	SSB(servings/day)	Fruit juice(servings/day)	Milk(servings/day)	Calcium(mg/day)
**Globe**				
**Women**				
Ages 20 to 39	0.94 (0.56,1.50)	0.23 (0.13,0.37)	0.55 (0.36,0.82)	626 (515,758)
Ages 40 to 59	0.50 (0.30,0.80)	0.16 (0.09,0.27)	0.53 (0.35,0.80)	641 (528,776)
Ages 60 and older	0.34 (0.20,0.53)	0.16 (0.09,0.26)	0.68 (0.44,1.01)	685 (564,827)
Women overall	0.56 (0.33,0.89)	0.18 (0.10,0.29)	0.60 (0.39,0.90)	656 (540,793)
**Men**				
Ages 20 to 39	1.04 (0.63,1.66)	0.18 (0.11,0.30)	0.51 (0.33,0.76)	574 (472,695)
Ages 40 to 59	0.55 (0.33,0.87)	0.13 (0.08,0.22)	0.48 (0.32,0.72)	587 (483,710)
Ages 60 and older	0.37 (0.22,0.58)	0.13 (0.07,0.21)	0.62 (0.41,0.92)	627 (516,758)
Men overall	0.61 (0.37,0.97)	0.14 (0.08,0.24)	0.55 (0.36,0.82)	600 (494,726)
**Both sexes overall**	0.58 (0.37,0.89)	0.16 (0.10,0.26)	0.57 (0.39,0.83)	629 (527,747)
**Country income level** ^1^				
**High income**				
**Women**				
Ages 20 to 39	0.84 (0.58,1.20)	0.36 (0.24,0.53)	0.70 (0.52,0.95)	780 (686,887)
Ages 40 to 59	0.43 (0.29,0.61)	0.25 (0.17,0.38)	0.68 (0.50,0.92)	798 (703,908)
Ages 60 and older	0.29 (0.20,0.42)	0.25 (0.16,0.37)	0.85 (0.63,1.15)	851 (751,967)
Women overall	0.49 (0.33,0.70)	0.28 (0.19,0.42)	0.76 (0.56,1.02)	816 (719,927)
**Men**				
Ages 20 to 39	0.95 (0.65,1.35)	0.29 (0.19,0.43)	0.65 (0.48,0.88)	716 (629,815)
Ages 40 to 59	0.47 (0.32,0.67)	0.21 (0.14,0.31)	0.61 (0.45,0.82)	730 (643,831)
Ages 60 and older	0.32 (0.22,0.45)	0.20 (0.13,0.30)	0.77 (0.57,1.04)	778 (686,886)
Men overall	0.54 (0.37,0.77)	0.23 (0.15,0.34)	0.69 (0.51,0.93)	747 (657,850)
**Both sexes overall**	0.51 (0.37,0.71)	0.25 (0.18,0.36)	0.72 (0.55,0.95)	782 (697,879)
**Upper middle income**				
**Women**				
Ages 20 to 39	1.29 (0.77,2.06)	0.30 (0.16,0.52)	0.68 (0.44,1.04)	667 (544,814)
Ages 40 to 59	0.69 (0.42,1.11)	0.22 (0.12,0.37)	0.67 (0.43,1.01)	684 (558,833)
Ages 60 and older	0.46 (0.28,0.74)	0.20 (0.11,0.35)	0.85 (0.55,1.28)	730 (598,888)
Women overall	0.77 (0.46,1.22)	0.23 (0.13,0.41)	0.75 (0.48,1.13)	699 (571,851)
**Men**				
Ages 20 to 39	1.42 (0.85,2.25)	0.24 (0.13,0.42)	0.63 (0.40,0.95)	611 (499,743)
Ages 40 to 59	0.75 (0.45,1.20)	0.18 (0.09,0.31)	0.61 (0.39,0.91)	625 (511,759)
Ages 60 and older	0.51 (0.31,0.80)	0.17 (0.09,0.29)	0.77 (0.50,1.16)	668 (547,811)
Men overall	0.84 (0.50,1.33)	0.19 (0.10,0.33)	0.68 (0.44,1.03)	639 (523,777)
**Both sexes overall**	0.80 (0.51,1.22)	0.21 (0.12,0.35)	0.72 (0.48,1.05)	670 (559,801)
**Lower middle income**				
**Women**				
Ages 20 to 39	0.94 (0.51,1.60)	0.16 (0.08,0.28)	0.46 (0.27,0.74)	564 (439,713)
Ages 40 to 59	0.51 (0.28,0.87)	0.11 (0.06,0.20)	0.45 (0.27,0.72)	578 (450,730)
Ages 60 and older	0.34 (0.19,0.57)	0.11 (0.05,0.20)	0.57 (0.34,0.91)	617 (481,779)
Women overall	0.56 (0.31,0.95)	0.12 (0.06,0.22)	0.50 (0.30,0.80)	591 (460,746)
**Men**				
Ages 20 to 39	1.04 (0.57,1.76)	0.13 (0.06,0.23)	0.42 (0.25,0.68)	516 (401,653)
Ages 40 to 59	0.56 (0.30,0.95)	0.09 (0.05,0.17)	0.41 (0.24,0.65)	528 (412,668)
Ages 60 and older	0.37 (0.20,0.63)	0.09 (0.04,0.16)	0.52 (0.31,0.83)	565 (440,714)
Men overall	0.61 (0.34,1.04)	0.10 (0.05,0.18)	0.46 (0.27,0.74)	540 (421,684)
**Both sexes overall**	0.59 (0.34,0.95)	0.11 (0.06,0.19)	0.48 (0.30,0.74)	566 (452,700)
**Low income**				
**Women**				
Ages 20 to 39	0.56 (0.30,0.96)	0.04 (0.02,0.08)	0.29 (0.16,0.47)	454 (355,572)
Ages 40 to 59	0.30 (0.16,0.50)	0.03 (0.01,0.06)	0.28 (0.16,0.46)	465 (363,585)
Ages 60 and older	0.20 (0.11,0.33)	0.03 (0.01,0.06)	0.35 (0.20,0.58)	497 (389,625)
Women overall	0.33 (0.18,0.56)	0.03 (0.02,0.06)	0.31 (0.18,0.51)	476 (372,599)
**Men**				
Ages 20 to 39	0.62 (0.34,1.06)	0.04 (0.02,0.07)	0.26 (0.15,0.43)	416 (326,526)
Ages 40 to 59	0.33 (0.18,0.55)	0.03 (0.01,0.05)	0.25 (0.14,0.41)	426 (334,538)
Ages 60 and older	0.22 (0.12,0.37)	0.02 (0.01,0.05)	0.32 (0.18,0.52)	455 (357,575)
Men overall	0.36 (0.20,0.62)	0.03 (0.01,0.05)	0.28 (0.16,0.46)	436 (342,550)
**Both sexes overall**	0.35 (0.20,0.56)	0.03 (0.02,0.06)	0.30 (0.18,0.47)	456 (366,563)

^1^Income categorizations based on the World Bank classification system: (http://data.worldbank.org/about/country-classifications/country-and-lending-groups).

**Fig 1:**
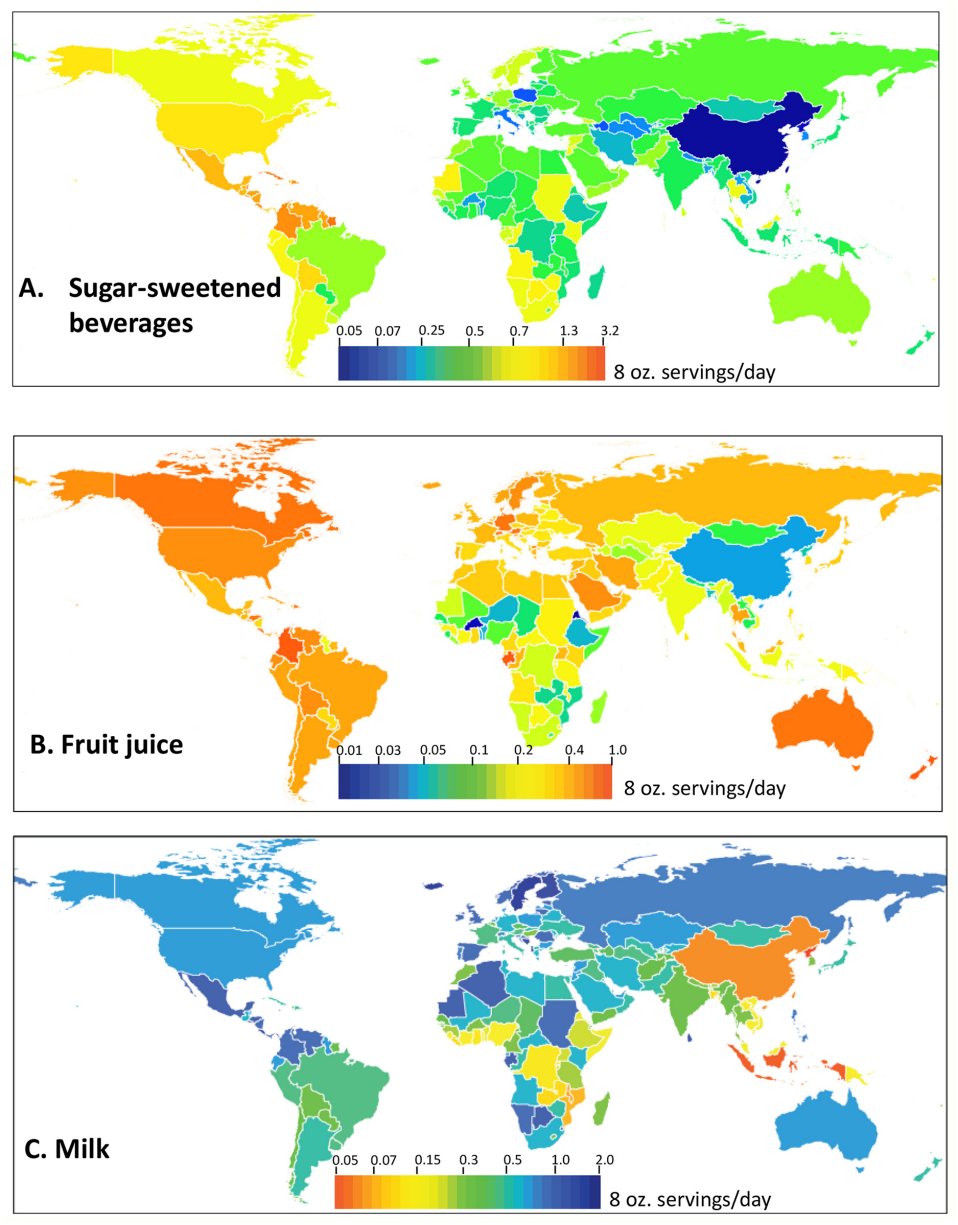
Consumption of non-alcoholic caloric beverages in 187 countries worldwide. A) SSBs, B) Fruit juice, C) Milk. Mean country-level beverage consumption levels in servings/day are represented by the color scales in each panel. Note that the scale range differs in each panel.

There was also large heterogeneity across geographical regions: almost a 10-fold difference between highest and lowest regional intake levels. Of 21 world regions, SSB consumption was highest in the Caribbean (1.9, 95%CI: 1.2, 3.0 servings/day), and lowest in East Asia (0.20, 95%CI: 0.16, 0.25 servings/day). SSB consumption was also high in Central Latin America, high-income North America, and Andean Latin America, with average intakes of over 0.8 servings per day of SSBs (Fig 1A and Table C in S1 File).

### Global distribution of fruit juice consumption

Adults worldwide in 2010 consumed an average of 0.16 (95%UI: 0.10, 0.26) servings/day of fruit juice, with greatest intake in women aged 20–39 (0.23, 95%UI: 0.13, 0.37) (Table 2). Fruit juice consumption on average increased with country income level, highest in high-income countries (0.25, 95%UI: 0.18, 0.36), and lowest in low-income countries (0.03, 95%UI: 0.02, 0.06).

Across geographic regions, fruit juice intake ranged from 0.66 (95%UI: 0.36, 1.13) servings/day to 0.013 (95%UI: 0.011, 0.017) servings/day, highest in Australasia and lowest in East Asia. Adults in Australasia, high-income North America, Central Latin America, and Andean Latin America consumed over a third of a serving per day of fruit juice, and adults in South Asia and East Asia consumed less than a quarter of a serving per day (Table C in S1 File).

### Global distributions of milk consumption and calcium intake

Milk consumption among adults averaged 0.57 (95%UI: 0.39, 0.83) servings/day globally and was on average highest in older adults than in younger adults: 0.68 (95%UI: 0.39, 0.90) servings/day in women age 60 and over and 0.51 (95%UI: 0.33, 0.76) servings/day in men aged 20–39 (Table 2). Adults in wealthier countries typically drank more milk than in poorer countries (high income: 0.72, 95%UI: 0.55, 0.95; upper-middle income: 0.72, 95%UI: 0.48, 1.05; lower-middle income: 0.48, 95%UI: 0.30, 0.74; low-income: 0.30, 95%UI: 0.18, 0.47 servings/day).

Across 21 world regions, Central Latin America was the region with highest milk intake (1.06, 95%UI: 0.68, 1.59 servings/day), and milk consumption also exceeded three-quarters of a serving in Europe and Southern Sub-Saharan Africa. Adults in East Asia and Oceania consumed the least milk, less than a quarter of a serving per day (Table C in S1 File).

Calcium intake was highly correlated with milk consumption in 2010 (r = 0.71), with highest levels in Western Europe (911, 95%UI: 824, 1009 mg/day) and U.S/Canada (872, 95%UI: 828, 918 mg/day), lowest levels in Eastern Sub-Saharan Africa (441, 95%UI: 348, 554) and a global average of 629 (95%UI: 527, 747) mg/day.

### National distributions of beverage consumption

#### SSBs

Across 187 countries, Trinidad and Tobago had the highest average consumption of SSBs, at 2.5 (95%UI: 1.5, 4.0) servings/day, and adults in Barbados, Suriname, Cuba, Saint Vincent and the Grenadines, the Dominican Republic, and Grenada drank over 2 servings/day of SSBs (Fig 1A and Table C in S1 File). China had the lowest levels of SSB consumption (0.05, 95%UI: 0.04, 0.06 servings/day), and SSB intake levels in North Korea and Azerbaijan were similarly low. Adults in the U.S. had the 26^th^-highest consumption of SSBs out of 187 countries, averaging 1.0 (95%UI: 0.9, 1.2) servings/day.

#### Fruit juice

Fruit juice consumption was highest in New Zealand (0.83, 95%UI: 0.44, 1.44 servings/day), and also exceeded three-quarters of a serving/day in Colombia (Fig 1B and Table B in S1 File). Adults in Eritrea, Burkina Faso, China, and Togo all had very low fruit juice consumption, close to zero servings/day. In the United States, adults on average drank about a third of a serving of fruit juice per day (0.36, 95%UI: 0.31, 0.41), ranking 21st of 187 countries worldwide.

#### Milk

Adults in Sweden and Iceland consumed the most milk in 2010, at 1.6 (95%UI: 1.4, 1.8) servings/day, and adults in Costa Rica, Bosnia and Herzegovina, Finland, and Sri Lanka also consumed over 1.3 servings/day on average (Fig 1C and Table B in S1 File). North Korea, and Indonesia had the lowest levels of milk consumption at less than 0.05 servings/day. In the United States, milk consumption among adults averaged 0.69 (95%UI: 0.61, 0.77) servings/day, ranking 64th out of 187 countries. Calcium intake followed similar patterns to milk intake and was highest in Finland and Iceland, where adults consumed over 1000 mg/day, and lowest in Mozambique and Malawi, where consumption was below 325 mg/day.

### Age, sex, and time trends in beverage consumption

#### SSBs

**Fig 2:**
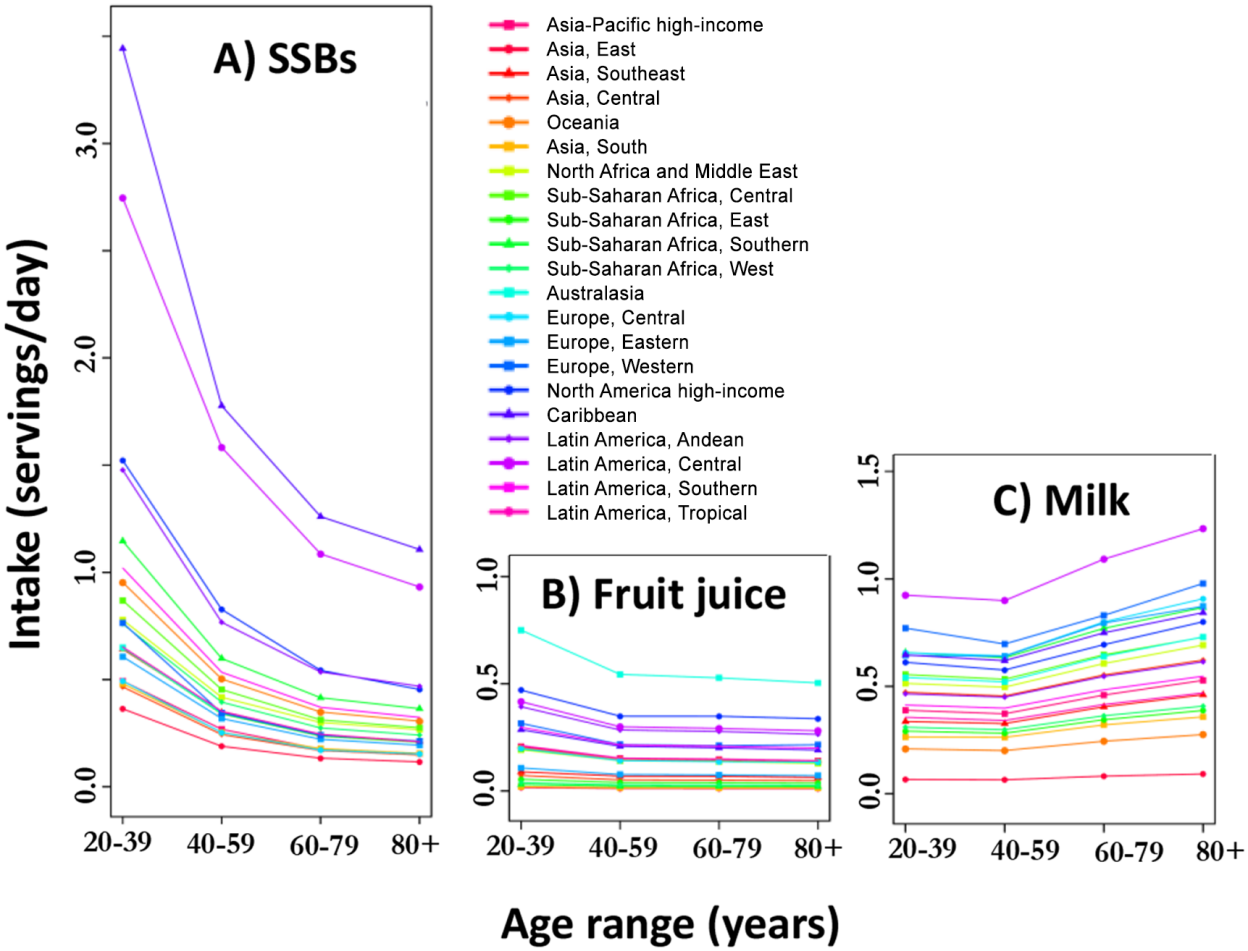
Global non-alcoholic caloric beverage consumption in 21 regions by age. A) SSBs, B) Fruit juice, C) Milk. Consumption levels are shown in four age groups for each region and each region is color-coded as shown in the legend.

SSB consumption generally followed an inverse age gradient, highest in adults under age 40, and lowest in adults over age 60 (Fig 2A and Table 2). There was a steep inverse age gradient in regions in Latin America and the Caribbean and in high-income North America, which was attenuated in regions of lower SSB consumption, such as East and South Asia By age and sex, regional consumption of SSBs was highest in men aged 20–39 in the Caribbean at 3.4 (95%UI: 2.0, 5.6) servings/day (Table C in S1 File). Men and women under age 60 in the Caribbean and Central Latin America also consumed over 1.5 servings/day of SSBs. Regional intake of SSBs was lowest in women over age 60 in East Asia (0.12, 95%UI: 0.09, 0.15 servings/day).

**Fig 3:**
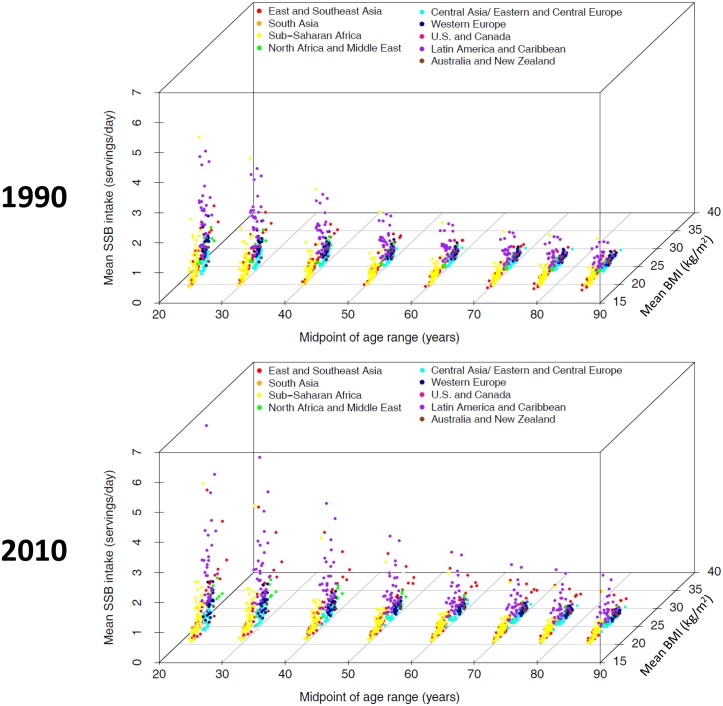
Regional age and time trends in SSB consumption and BMI. Each three-dimensional plot shows age, mean BMI, and mean SSB intake on the x-, y-, and z-axes respectively. Each point represents one age group in one country and the points are color-coded by super-region as shown in the legend. The top panel shows data from 1990 and the bottom panel shows data from 2010.

At the country level, in 2010 men aged 20–29 in Trinidad and Tobago (5.1, 95%UI: 2.9, 8.7 servings/day) consumed the most SSBs in the world, and women over age 80 in China consumed the least (0.026, 95%CI: 0.022, 0.031 servings/day) (Table B in S1 File). The cross-country correlation between SSB consumption and mean body-mass index (BMI) also followed an inverse age gradient, with strongest correlation in adults under age 45, and weakest correlation in in adults over age 65 (Fig 3). Between 1990 and 2010, SSB consumption increased in several countries in Latin America and the Caribbean and Southeast Asia, however, at the regional level these changes were not statistically significant (Fig A in S1 File).

#### Fruit juice

In contrast to SSBs, regional fruit juice consumption showed little relationship with age (Fig 2B). Women aged 20–39 in Australasia had the highest levels of fruit juice intake, at 0.92 (95%CI: 0.47, 1.7) servings/day and men over age 60 in East Asia had the lowest, at 0.01 (0.01, 0.02) servings/day (Table C in S1 File).

Among all countries, fruit juice consumption was highest in women aged 20–29 in New Zealand (1.3, 95%UI: 0.6, 2.3 servings/day) and close to zero in men and women of all ages in Eritrea and Burkina Faso. Changes in fruit juice consumption between 1990 and 2010 were not statistically significant at the regional level (Fig A in S1 File) and showed little relationship with mean BMI (Fig C in S1 File).

#### Milk

Unlike either SSB or fruit juice consumption, milk intake in many regions was lower in younger ages and higher in older ages (Fig 2C; Fig B in S1 File). At the regional level, women over age 60 in Central Latin America drank the most milk (1.3, 95%UI: 0.8, 1.9 servings/day), and men under age 60 in East Asia drank the least (0.07, 95%UI: 0.05, 0.08 servings/day). As with milk consumption, calcium intake increased with age, highest in women over age 60 in Western Europe and high-income North America, and lowest in men under age 40 in Eastern Sub-Saharan Africa and East Asia (Table C in S1 File).

By country, milk intake was highest in the world in women over age 80 in Sweden and Iceland (2.1, 95%UI: 1.8, 2.4 servings/day) and lowest in men under age 50 in North Korea (0.03, 95%UI: 0.02, 0.06 servings/day) (Table B in S1 File). At the regional level, changes in milk consumption between 1990 and 2010 were generally not statistically significant.

## Discussion

These results, based on collection and harmonization of both individual-level national dietary intake surveys and national food availability data provide comprehensive estimates of global, regional, and national consumption of SSBs, fruit juice, and milk, by age and sex. These are the first quantitative estimates of non-alcoholic beverage consumption in 187 countries of the world, and provide information that can inform several areas in global health. First, these results identify gaps in current dietary data around the world, indicating the need for improved dietary surveillance in particular world regions. Second, these data provide the basis for quantitative assessment of the impact of beverage intakes on disease burdens [33]. Third, this work provides estimates of beverage intakes that will be useful baselines for measuring the efficacy of policies and interventions related both to undernutrition and overnutrition.

In 2010, beverage consumption varied significantly by region. SSB intake was highest in the Americas, particularly in parts of Latin America and the Caribbean, where both commercial and homemade SSBs are widely consumed [34]. Fruit juice intake was highest in Australia and New Zealand, perhaps reflecting high levels of production and marketing in those countries [35]. Milk intake was highest in parts of Northern Europe where dairy farming is widespread and dairy products have traditionally beendietary mainstays [36,37]. Consumption of SSBs, fruit juices, and milk was particularly low in East Asia, perhaps indicating the sociocultural importance of tea-drinking in that region, the widespread consumption of soy-based beverages, as well as the high prevalence of lactose-intolerance [38–41]. Milk consumption was also low in parts of East Africa, which may reflect lower availability of milk [42], the prevalence of lactose-intolerance [38], preferred intake of alternative traditional beverages[43], or other cultural and macroeconomic factors that require further investigation.

Although there was little difference in beverage consumption levels and trends between men and women, beverage consumption showed major variation by age, with younger adults drinking more SSBs, and older adults drinking more milk. Higher consumption of SSB in younger adults may stem from a generational effect, which may in part be due to heavier marketing and advertising of SSBs to younger populations [44,45]. Higher consumption of milk in older adults may be due to dietary guidelines promoting milk consumption to increase calcium intake and prevent bone mineral loss, especially among older women at risk for osteoporosis [26], or may also be due to a generational effect.

Several strengths of this study can be noted. We conducted systematic searches and contacted experts worldwide to collect, evaluate, and analyze global individual-level data on consumption levels of SSBs, milk, and fruit juice. Data on dietary intakes of these beverages were primarily from nationally-representative surveys and were age-, sex-, and time-specific. We used standardized methods for data extraction and analysis across surveys and beverage categories to ensure comparability of the dietary information collected, and we assessed surveys for quality of measurement methods to maximize data validity. In addition to individual-level information on beverage consumption from national surveys, we also extracted data relevant to SSB, fruit juice, and milk annual availability in each of the 187 countries in our analysis between 1990 and 2010 from FAO food balance sheets, and harmonized these data with intake data using hierarchical modeling methods. Having both high-quality national survey data on beverage intake, albeit with partial global coverage, in addition to beverage availability data with complete global coverage provided as comprehensive data as possible for estimating global levels of consumption of SSBs, fruit juices, and milk. We used established hierarchical modelling methods to combine national survey data on individual-level beverage intakes with country-level data relevant to beverage availability, to address differences in dietary intake data representativeness and measurement methodologies, and to capture uncertainty due to measurement error, sampling uncertainty, and modeling uncertainty. The model was informed by time-varying covariates, incorporated uncertainty in primary data, and was subjected to external validation. These methods have allowed us to investigate in considerable detail global trends in the consumption of three major beverage categories.

These results should be interpreted with some limitations in mind. We noted sparsity of individual-level beverage intake data in particular geographical regions and time periods despite our systematic approach to survey identification. In particular, fewer data sources on individual-level intakes were available to inform estimates of beverage consumption in 1990 than in 2010, and comparatively fewer data sources on individual-level intakes were available in Oceania and much of Sub-Saharan Africa, and some South Asian countries. However, we incorporated into our analysis data on beverage availability from FAO food balance sheets for each of 187 countries in every year between 1990 and 2010 so that estimates for every country in the analysis were informed by data from multiple time points. Additionally, identification of areas of data sparsity is important in itself for identifying gaps in global dietary surveillance and planning future surveys. The food availability data used in this analysis are reported by individual countries and may therefore heterogeneously capture availability from non-commercial channels, which could be important sources of beverages in many low/middle income countries[46]; however, we used established methods to harmonize food availability data with measured intake levels to minimize bias due to such factors. Some surveys with pre-categorized dietary data had slightly different classifications of SSBs, fruit juices, and milk than those used in our study; however, such cases were limited and our modeling approach downweighted studies using non-optimal metrics in the model,. As this analysis was part of a larger initative that focused on chronic disease-related dietary intakes (the GBD Nutrition and Chronic Diseases Expert Group (NutriCoDE)), we did not collect data in children in this round of analysis, although children are included in further data collection efforts that are currently underway.

Our efforts to systematically collect, evaluate, and pool data from both individual-level national dietary intake surveys and country-level food availability data have provided a comprehensive assessment of the global consumption of SSBs, fruit juices, and milk. Our results also highlight the sparsity of data on individual-level dietary intakes in particular world regions, illustrating the need for improved future dietary surveillance using validated, standardized, nationally-representative surveys. Given that carrying out such surveys can be expensive and logistically challenging, our work also provides a robust modeling methodology by which dietary intakes can be estimated in data-sparse regions. These results are valuable for providing a detailed picture of global beverage consumption levels, useful both in quantifying disease burdens related to beverage intake and in framing health policies and interventions directed at reducing these disease burdens.

## Supporting Information

S1 File Supporting Appendix, Tables, and Figures. (PDF)
