# Correction: Global, Regional, and National Consumption of Sugar-Sweetened Beverages, Fruit Juices, and Milk: A Systematic Assessment of Beverage Intake in 187 Countries

**DOI:** 10.1371/journal.pone.0214344

**Published:** 2019-03-27

**Authors:** Gitanjali M. Singh, Renata Micha, Shahab Khatibzadeh, Peilin Shi, Stephen Lim, Kathryn G. Andrews, Rebecca E. Engell, Majid Ezzati, Dariush Mozaffarian, Global Burden of Diseases Nutrition and Chronic Diseases Expert Group (NutriCoDE)

In the Acknowledgments, there is an error in affiliation for Abdulrahman O. Musaiger of the Global Burden of Diseases Nutrition and Chronic Diseases Expert Group (NutriCoDE) authorship group. The correct affiliation is: Arab Center for Nutrition, University of Bahrain, Bahrain.
