# Supplementary material for: Global, Regional, and National Consumption of Sugar-Sweetened Beverages, Fruit Juices, and Milk: A Systematic Assessment of Beverage Intake in 187 Countries
Source: PLoS One. 2015 Aug 5;10(8):e0124845. doi: 10.1371/journal.pone.0124845 (PMC4526649; doi:10.1371/journal.pone.0124845)
Supplement: S1 File — (PDF) [file pone.0124845.s001.pdf]

Supporting Information

Appendix A

Statistical methods

Despite efforts to collect as complete and comprehensive data as possible on global consumption levels of SSBs, fruit juices, milk, and calcium, data in some countries were not available. To account for these missing data, we used an age-integrating Bayesian hierarchial model, DisMod3, developed as part of the Global Burden of Disease 2010 project (1). This model pooled data from multiple sources and modeled missing data using informative time-varying covariates, borrowing information across geographical region and time period while also incorporating uncertainty due to measurement error and model specification. The model was fit using a randomized Markov Chain Monte Carlo (MCMC) algorithm based on the Adaptive Metropolis step function and estimated fixed effects for study-specific and national-level covariates and random effects for GBD region and country. The MCMC was implemented in the PyMC Python module(2) to generate 1000 draws of dietary intake for each country-age-sex strata after discarding iterations from the burn-in convergence period. Goodness-of-fit was assessed using a hold-out cross-validation approach, as described elsewhere (1, 3, 4). Across all 1000 simulations for a given country, year, sex combination, we used the median value and 2.5 and 97.5 percentile of each set of draws of dietary intake as the estimate and the upper and lower confidence intervals for this outcome. The model outputs estimates of dietary intakes for both men and women in single year age groups for 1990 and 2010 for 187 countries worldwide.

Both study-specific and national-level covariates were included in the model. The study-specific covariates accounted for inconsistencies in the raw data; for example, data that are subnational (rather than nationally representative), or data collected using less-optimal methods. These study-specific covariates are indicator variables used in the model to adjust data collected using less-optimal methods to the level consistent with those of the gold standard data. For sugar-sweetened beverages, three

study-specific indicator variables were used to indicate the quality of measurement method and the national representativeness of the data. In collecting dietary data, diet records, recalls, and FFQs provided estimates of individual-level consumption and are therefore the gold-standard methods, while household availability/budget surveys estimate food intake/expenditure at the household level and are therefore less-optimal. The first study-specific variable indicates whether the data were collected using the optimal methods of diet records, recalls, or FFQs or the sub-optimal methods of household availability/budget surveys. The second study-specific variable denotes whether the data were collected based on the optimal definition of the exposure: total intake of any sugar-sweetened beverage with ≥50 kcal per 8 oz (226.8 grams) serving, including carbonated beverages, soft drinks, sodas, energy drinks, fruit drinks etc.) or a suboptimal metric. The third is an indicator variable for whether the data are from non-nationally-representative studies/surveys. In addition, we allowed the variance of sub-national-level data to be different from nationally-representative data.

Country-level data on food availability was used in the model to inform the global and country-level trends. We used data from the Food Balance Sheets of the Food and Agriculture Organization of the United Nations (FAO) which captures a country's annual food availability for human consumption based on reported local production, imports, and exports, adjusted for other uses (livestock, seed) (5). We used the FAO food balance sheets to construct a variable that measures the annual food availability per capita for sugars, by combining: sugar and sweeteners, sugar (raw equivalent), sugar beet, sugar cane, non-centrifugal sugar, and sugarcrops. Table S1 presents the effect size estimated for the study-level and national covariates from the DisMod3 model. Figure S1 provides an example of DisMod3 region-level outputs.

Appendix A Table 1. Effect sizes and standard errors for study-level and national covariates

Covariate	Effect size mean	Effect size SE
**Study level covariates**		
Suboptimal dietary assessment method	0.001	0.096
Suboptimal exposure definition	0.213	0.278
Non-nationally representative data	0.459	0.086
Sex	0.088	0.029
**National covariates**		
Log per capita sugar availability based on FAO data	0.789	0.123

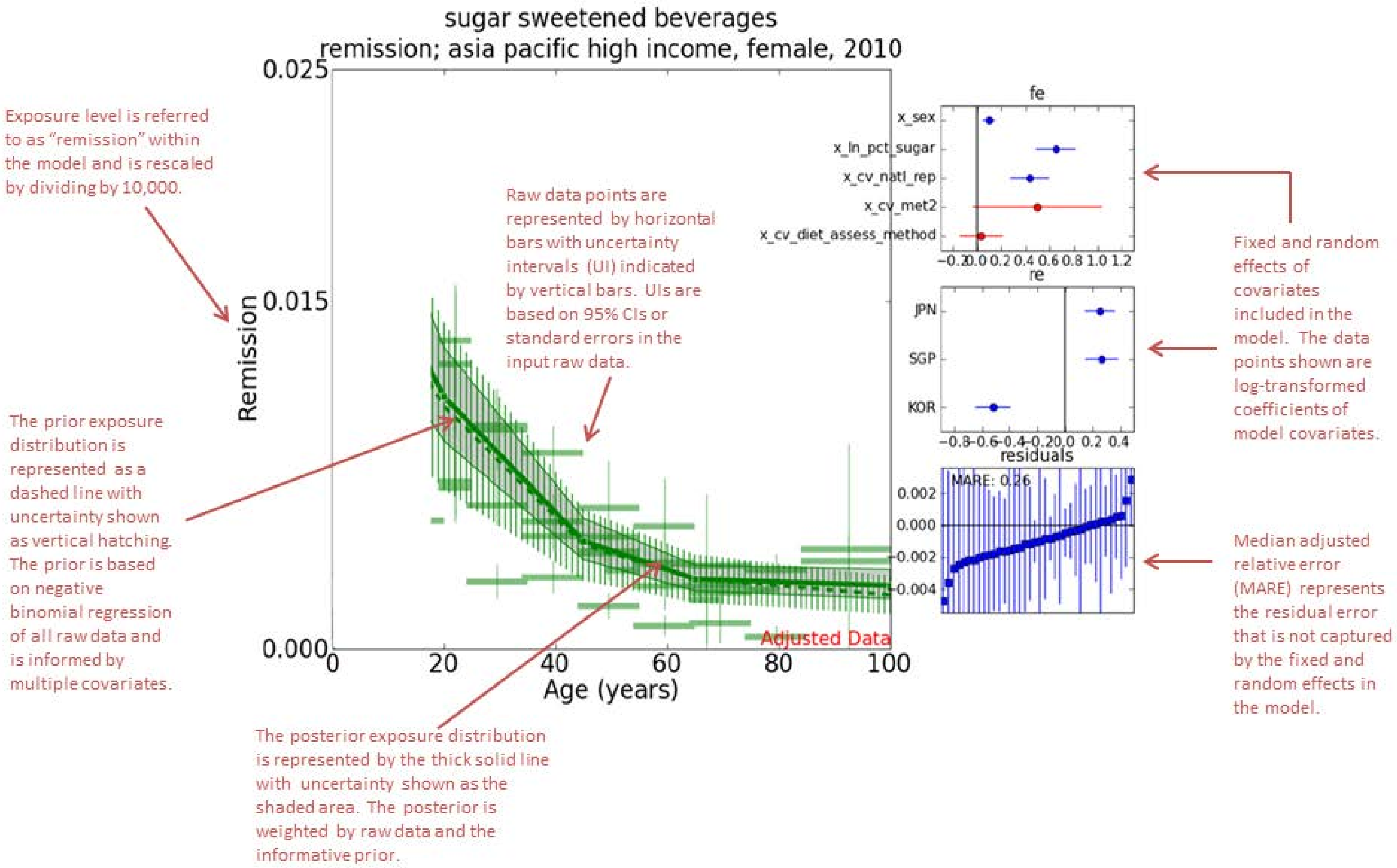

Appendix A Figure. Graphical representation and explanation of region-level outputs of the Bayesian hierarchical model. Results for women in the high-income Asia Pacific region in 2010 are shown as an example.

References:

1. Lim SS, Vos T, Flaxman AD, Danaei G, Shibuya K, Adair-Rohani H, Amann M, Anderson HR, Andrews KG, Aryee M, Atkinson C, Bacchus LJ, Bahalim AN, Balakrishnan K, Balmes J, Barker-Collo S, Baxter A, Bell ML, Blore JD, Blyth F, Bonner C, Borges G, Bourne R, Boussinesq M, Brauer M, Brooks P, Bruce NG, Brunekreef B, Bryan-Hancock C, Bucello C, Buchbinder R, Bull F, Burnett RT, Byers TE, Calabria B, Carapetis J, Carnahan E, Chafe Z, Charlson F, Chen H, Chen JS, Cheng AT, Child JC, Cohen A, Colson KE, Cowie BC, Darby S, Darling S, Davis A, Degenhardt L, Dentener F, Des Jarlais DC, Devries K, Dherani M, Ding EL, Dorsey ER, Driscoll T, Edmond K, Ali SE, Engell RE, Erwin PJ, Fahimi S, Falder G, Farzadfar F, Ferrari A, Finucane MM, Flaxman S, Fowkes FG, Freedman G, Freeman MK, Gakidou E, Ghosh S, Giovannucci E, Gmel G, Graham K, Grainger R, Grant B, Gunnell D, Gutierrez HR, Hall W, Hoek HW, Hogan A, Hosgood HD, 3rd, Hoy D, Hu H, Hubbell BJ, Hutchings SJ, Ibeanusi SE, Jacklyn GL, Jasrasaria R, Jonas JB, Kan H, Kanis JA, Kassebaum N, Kawakami N, Khang YH, Khatibzadeh S, Khoo JP, Kok C, Laden F, Lalloo R, Lan Q, Lathlean T, Leasher JL, Leigh J, Li Y, Lin JK, Lipshultz SE, London S, Lozano R, Lu Y, Mak J, Malekzadeh R, Mallinger L, Marcenes W, March L, Marks R, Martin R, McGale P, McGrath J, Mehta S, Mensah GA, Merriman TR, Micha R, Michaud C, Mishra V, Mohd Hanafiah K, Mokdad AA, Morawska L, Mozaffarian D, Murphy T, Naghavi M, Neal B, Nelson PK, Nolla JM, Norman R, Olives C, Omer SB, Orchard J, Osborne R, Ostro B, Page A, Pandey KD, Parry CD, Passmore E, Patra J, Pearce N, Pelizzari PM, Petzold M, Phillips MR, Pope D, Pope CA, 3rd, Powles J, Rao M, Razavi H, Rehfuess EA, Rehm JT, Ritz B, Rivara FP, Roberts T, Robinson C, Rodriguez-Portales JA, Romieu I, Room R, Rosenfeld LC, Roy A, Rushton L, Salomon JA, Sampson U, Sanchez-Riera L, Sanman E, Sapkota A, Seedat S, Shi P, Shield K, Shivakoti R, Singh GM, Sleet DA, Smith E, Smith KR, Stapelberg NJ, Steenland K, Stockl H, Stovner LJ, Straif K, Straney L, Thurston GD, Tran JH, Van Dingenen R, van Donkelaar A, Veerman JL, Vijayakumar L, Weintraub R, Weissman MM, White RA, Whiteford H, Wiersma ST, Wilkinson JD, Williams HC, Williams W, Wilson N, Woolf AD, Yip P, Zielinski JM, Lopez AD, Murray CJ, Ezzati M, AlMazroa MA, Memish ZA. A comparative risk assessment of burden of disease and injury attributable to 67 risk factors and risk factor clusters in 21 regions, 1990-2010: a systematic analysis for the Global Burden of Disease Study 2010. Lancet. 2012;380(9859):2224-60. Epub 2012/12/19. doi: 10.1016/s0140-6736(12)61766-8. PubMed PMID: 23245609.
2. Patil A, Huard D, C F. PyMC: Markov Chain Monte Carlo for Python, version 2.0. Journal of Statistical Software.
3. Micha R, Khatibzadeh S, Shi P, Fahimi S, Lim S, Andrews KG, Engell RE, Powles J, Ezzati M, Mozaffarian D. Global, regional, and national consumption levels of dietary fats and oils in 1990 and 2010: a systematic analysis including 266 country-specific nutrition surveys. BMJ (Clinical research ed). 2014;348:g2272. Epub 2014/04/17. doi: 10.1136/bmj.g2272. PubMed PMID: 24736206; PMCID: 3987052.
4. Powles J, Fahimi S, Micha R, Khatibzadeh S, Shi P, Ezzati M, Engell RE, Lim SS, Danaei G, Mozaffarian D. Global, regional and national sodium intakes in 1990 and 2010: a systematic analysis of 24 h urinary sodium excretion and dietary surveys worldwide. BMJ open. 2013;3(12):e003733. Epub 2013/12/25. doi: 10.1136/bmjopen-2013-003733. PubMed PMID: 24366578; PMCID: 3884590.
5. FAO. U.N. Food and Agriculture Organization Food Balance Sheets. http://faostatfaoorg/site/354/defaultaspx. 2013.

Figure A

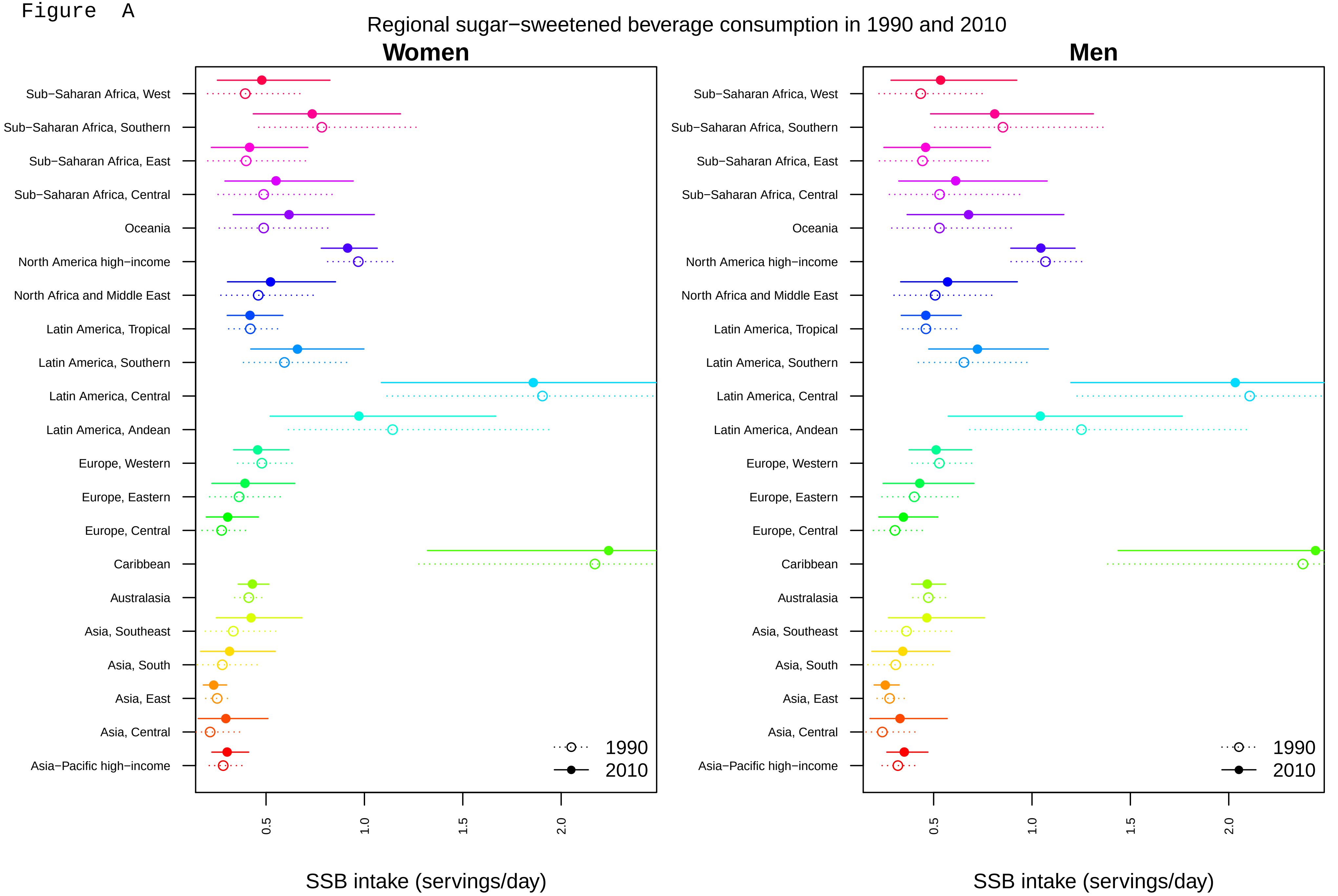
Regional sugar−sweetened beverage consumption in 1990 and 2010
Women
Men
Sub−Saharan Africa, West
Sub−Saharan Africa, Southern
Sub−Saharan Africa, East
Sub−Saharan Africa, Central
Oceania
North America high−income
North Africa and Middle East
Latin America, Tropical
Latin America, Southern
Latin America, Central
Latin America, Andean
Europe, Western
Europe, Eastern
Europe, Central
Caribbean
Australasia
Asia, Southeast
Asia, South
Asia, East
Asia, Central
Asia−Pacific high−income
1990
2010
0.5
1.0
1.5
2.0
SSB intake (servings/day)

Figure B: Mean milk consumption and mean BMI by age group for adults over age 20 in 1990 and 2010.

1990

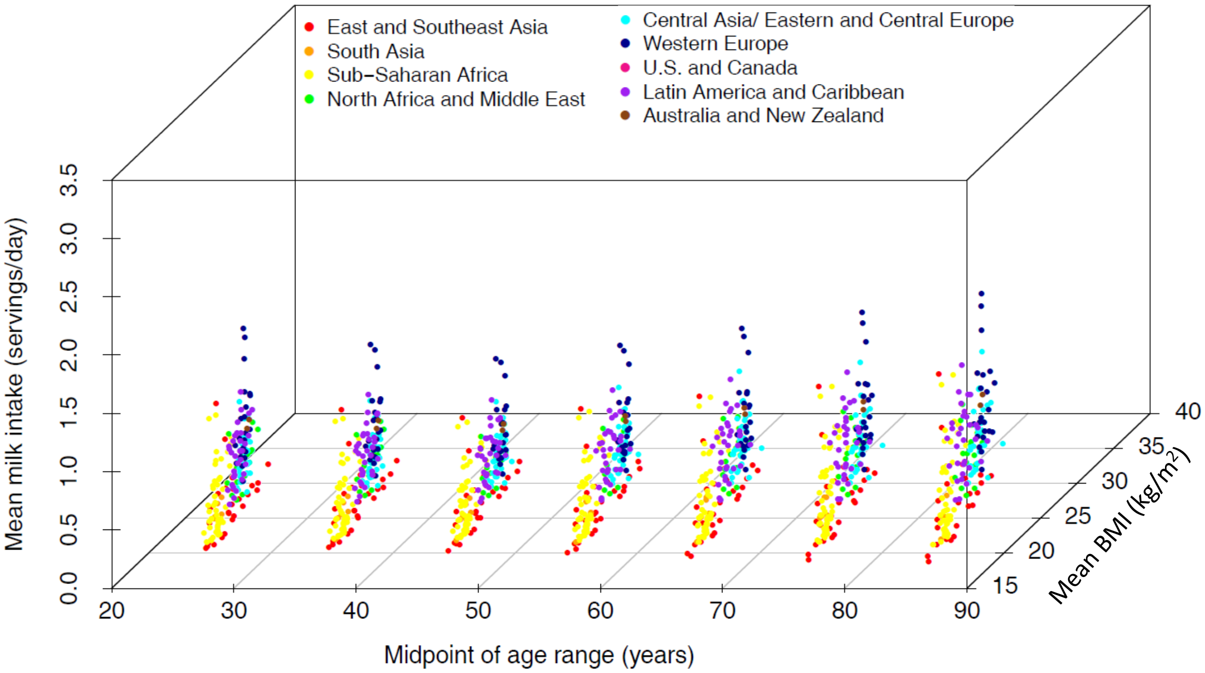

2010

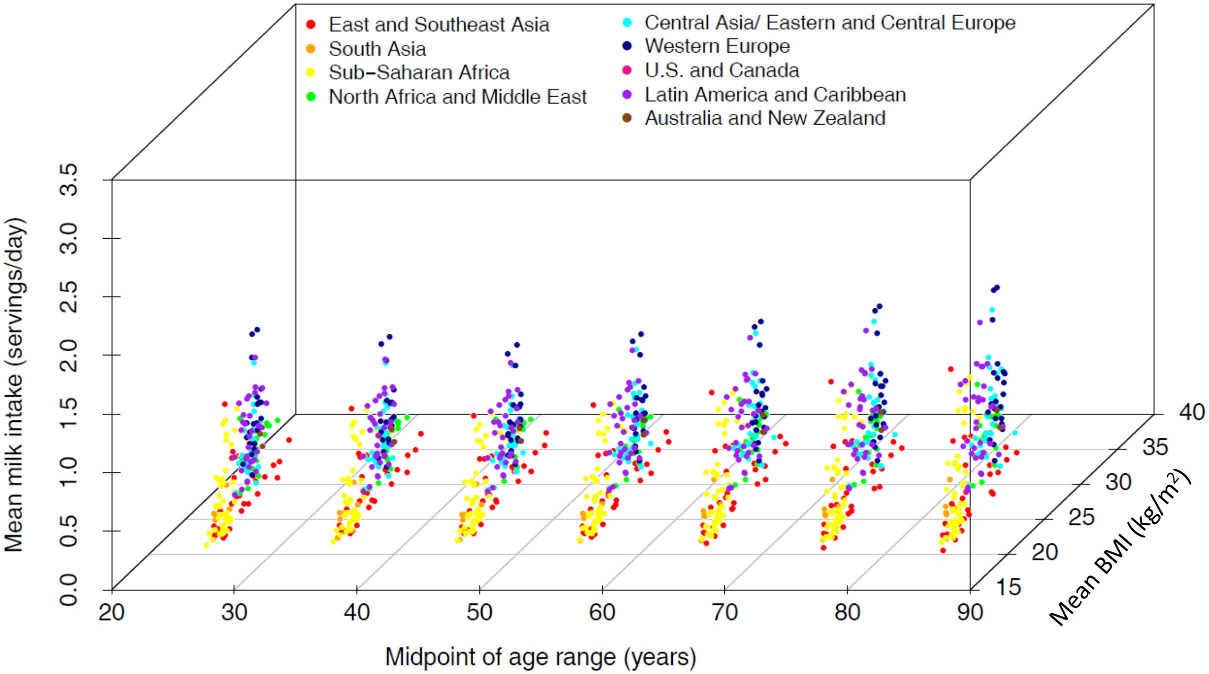

Figure C: Mean fruit juice consumption and mean BMI by age group for adults over age 20 in 1990 and 2010.

1990

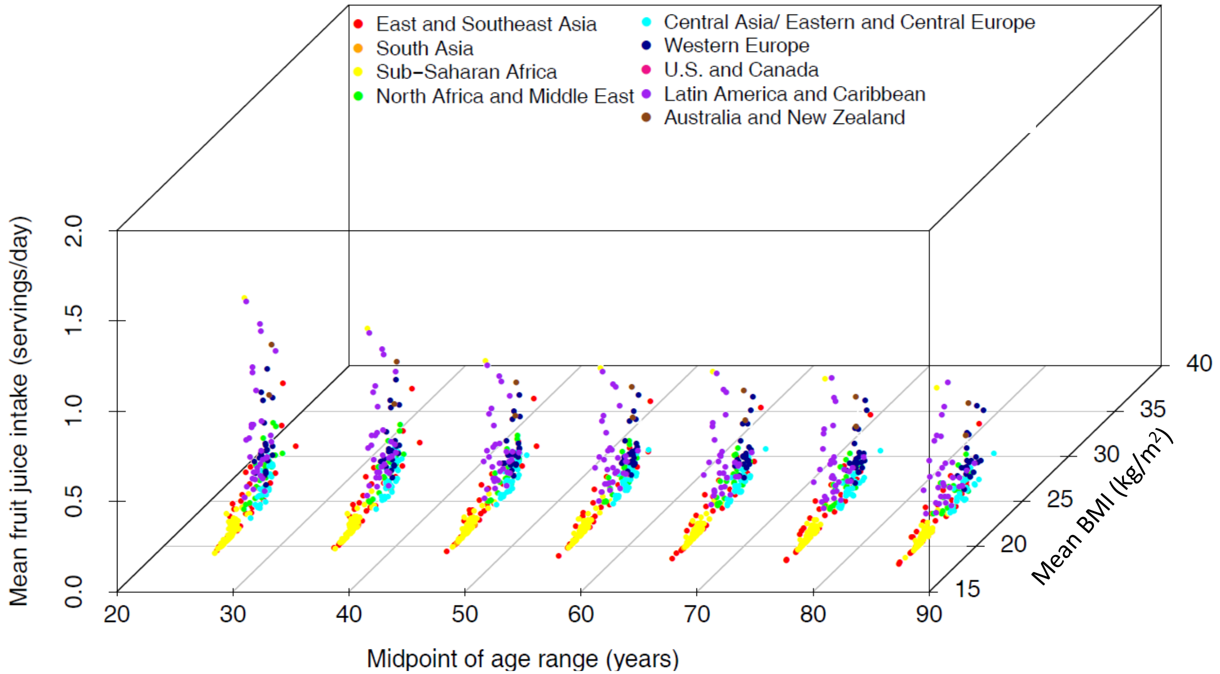

2010

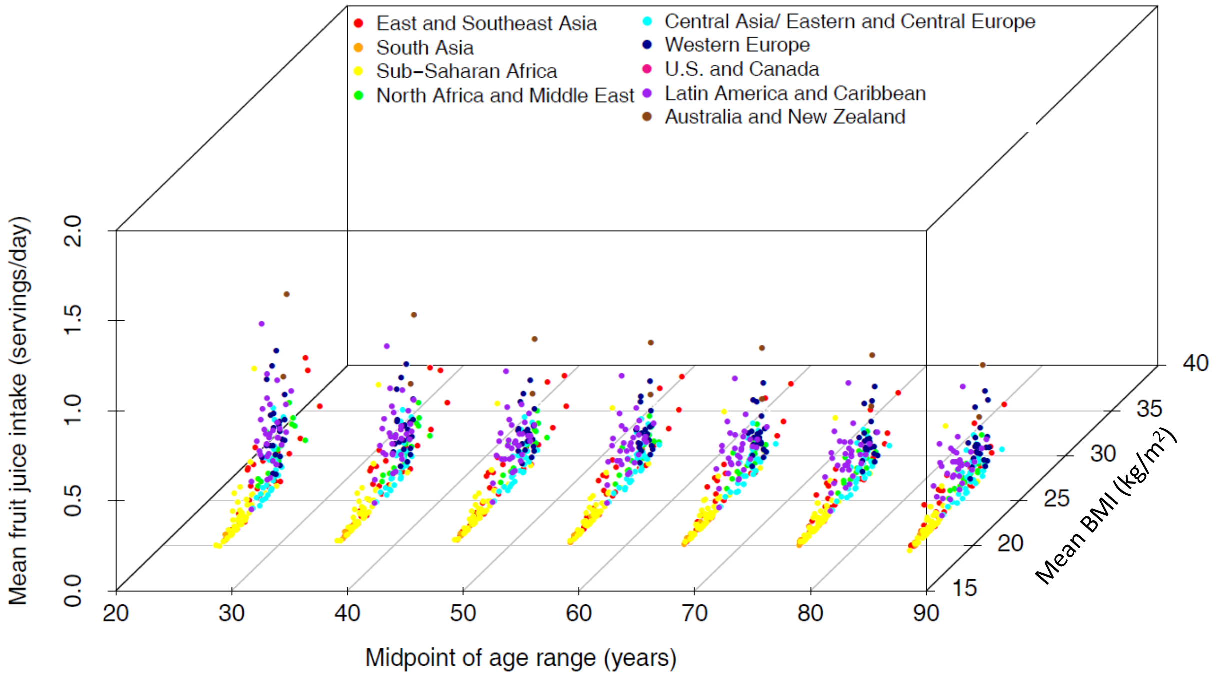

Figure D

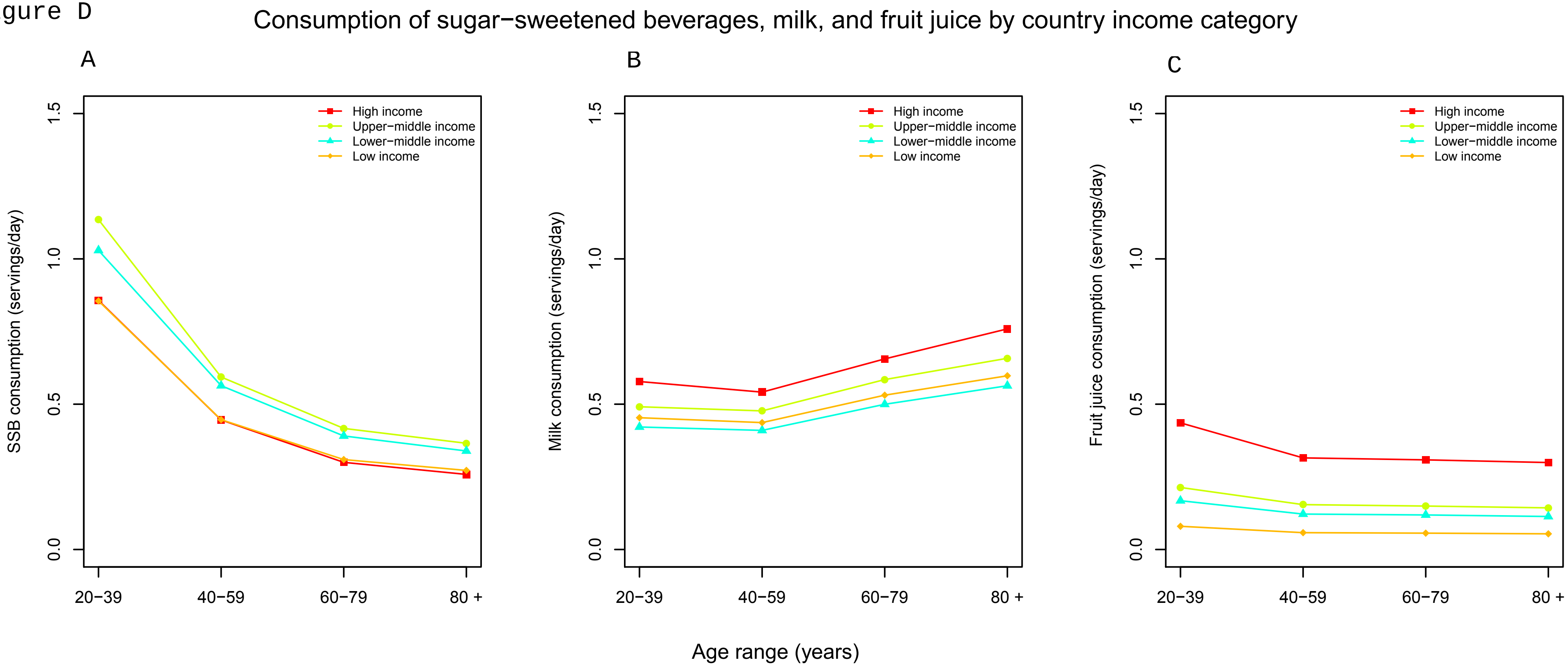
Consumption of sugar−sweetened beverages, milk, and fruit juice by country income category
A
B
C
SSB consumption (servings/day)
Milk consumption (servings/day)
Fruit juice consumption (servings/day)
High income
Upper−middle income
Lower−middle income
Low income
0.0
0.5
1.0
1.5
20−39
40−59
60−79
80 +
Age range (years)

Table A: Characteristics of data sources on sugar-sweetened beverage intake.

Country	Study name	Time period	Age range (years)	Sample size	Percent female	Coverage	Diet assessment method	Sampling method
Argentina	Encuesta Nacional de Nutrición y Salud - National Nutrition and Health Survey (ENNyS)	2004-2005	20-49	4429	100.0	national	single short-term diet recall	multi-stage sampling
Australia	National Nutrition Survey	1995-1996	20-100	10851	53.2	national	single short-term diet recall	multi-stage sampling
Austria	Austrian Community Health and Nutrition Survey	1999	20-100	7098	NR	national	household availability/budget survey	multi-stage sampling
Austria	Austrian Study on Nutritional Status	2005-2006	20-64	2123	NR	national	single short-term diet recall	multi-stage sampling
Barbados	Identifying new genetic and obesity-related factors contributing to prostate and breast cancer risk in persons of African descent	2004-2007	42-88	279	45.5	national	food-frequency questionnaire	one-stage sampling
Belgium	Belgian Community Health and Nutrition Survey	1999	20-100	3745	NR	national	household availability/budget survey	multi-stage sampling
Belgium	Belgian National Food Consumption Survey	2004	20-100	2240	49.4	national	multiple short term diet recall	multi-stage sampling
Belgium	Belgian Community Health and Nutrition Survey	1987-1988	20-100	3235	NR	national	household availability/budget survey	multi-stage sampling
Belgium	Belgian Community Health and Nutrition Survey	1996-1997	20-100	2041	NR	national	household availability/budget survey	multi-stage sampling
Brazil	Household Health Survey (ISA-SP)	2003	20-100	57	47.4	regional	single short-term diet recall	multi-stage sampling
Bulgaria	National Nutrition Survey	2004	20-75	853	51.9	national	single short-term diet recall	one-stage sampling
Canada	Canadian Community Health and Nutrition Survey	2004	20-101	19728	55.6	national	multiple short term diet recall	one-stage sampling
China	2002 China National Nutrition and Health Survey	2002	20-101	49071	52.8	national	food-frequency questionnaire	one-stage sampling
Colombia	Database from National survey of nutritional situation in Colombia	2005	20-64	11409	53.5	national	multiple short term diet recall	one-stage sampling
Croatia	Croatian Community Health and Nutrition Survey	1999	20-100	2937	NR	national	household availability/budget survey	multi-stage sampling
Croatia	Croatian Community Health and Nutrition Survey	2004	20-100	2847	NR	national	household availability/budget survey	multi-stage sampling
Cyprus	Cypriot Community Health and Nutrition Survey	2003	20-100	2990	NR	national	household availability/budget survey	convenience sampling
Cyprus	Cypriot Community Health and Nutrition Survey	1996-1997	20-100	3308	NR	national	household availability/budget survey	multi-stage sampling

Country	Study name	Time period	Age range (years)	Sample size	Percent female	Coverage	Diet assessment method	Sampling method
Czech Republic	Individual food consumption study	2003-2004	20-64	1751	NR	national	multiple short term diet recall	multi-stage sampling
Finland	Finn Community Health and Nutrition Survey	1985	20-100	8200	NR	national	household availability/budget survey	multi-stage sampling
Finland	Finn Community Health and Nutrition Survey	1990	20-100	8258	NR	national	household availability/budget survey	convenience sampling
Finland	FINDIET 1992	1992	25-64	1861	53.3	national	multiple short term diet recall	multi-stage sampling
Finland	Finn Community Health and Nutrition Survey	1998	20-100	4359	NR	national	household availability/budget survey	one-stage sampling
Finland	FINDIET 2007	2007	25-74	2039	53.0	national	multiple short term diet recall	multi-stage sampling
France	Enquete Individuelle et Nationale sur les Consommations Alimentaires	1999	20-64	1195	NR	national	multiple short term diet recall	multi-stage sampling
France	Etude nationale nutrition santé (ENNS); National Nutrition and Health survey	2006-2007	20-74	2676	63.6	national	multiple short term diet recall	multi-stage sampling
Germany	German Community Health and Nutrition Survey	1988	20-100	17855	NR	national	household availability/budget survey	multi-stage sampling
Germany	German Community Health and Nutrition Survey	1993	20-100	15825	NR	national	household availability/budget survey	one-stage sampling
Germany	German Community Health and Nutrition Survey	1998	20-100	12680	NR	national	household availability/budget survey	one-stage sampling
Germany	German Nutrition Survey 1998	1997-1999	20-79	3861	56.2	national	food-frequency questionnaire	one-stage sampling
Greece	Greek Community Health Survey and Nutrition Nutrition	1981	20-100	6034	NR	national	household availability/budget survey	multi-stage sampling
Greece	Greek Community Health Survey and Nutrition Nutrition	1987	20-100	6489	NR	national	household availability/budget survey	multi-stage sampling
Greece	Greek Community Health Survey and Nutrition Nutrition	1998	20-100	6258	NR	national	household availability/budget survey	multi-stage sampling
Greece	Greek Community Health Survey and Nutrition Nutrition	2004	20-100	6555	NR	national	household availability/budget survey	multi-stage sampling
Greece	Greek component of the European Prospective Investigation into Cancer and Nutrition (EPIC)	1994-1999	25-84	28034	58.8	sub-regional	food-frequency questionnaire	multi-stage sampling
Greece	ATTICA Study	2001-2002	20-74	989	44.7	regional	food-frequency questionnaire	one-stage sampling
Greenland	Inuit Health in Transition	2005-2009	20-84	2663	56.0	national	food-frequency questionnaire	multi-stage sampling

Country	Study name	Time period	Age range (years)	Sample size	Percent female	Coverage	Diet assessment method	Sampling method
Hungary	Hungarian Community Health and Nutrition Survey	1991	20-100	11813	NR	national	household availability/budget survey	one-stage sampling
Hungary	The 3rd National Hungarian Survey	2003-2004	20-64	927	NR	national	multiple short term diet recall	multi-stage sampling
Iceland	Dietary Survey of the Icelanders	1990	20-80	1095	51.5	national	single short-term diet recall	one-stage sampling
Iceland	The Diet of Icelanders, Dietary Survey of The Icelandic Nutrition Council 2002	2002	20-80	1118	53.8	national	food-frequency questionnaire	one-stage sampling
Iran	Non-communicable disease surveillance in Islamic Republic of Iran	2005	20-64	72717	50.1	national	single short-term diet recall	multi-stage sampling
Iran	Comprehensive National Household Food Consumption Survey	1990-1995	20-100	69	NR	national	multiple short term diet recall	multi-stage sampling
Ireland	Irish Community Health and Nutrition Survey	1987	20-100	7705	NR	national	household availability/budget survey	multi-stage sampling
Ireland	Irish Community Health and Nutrition Survey	1994	20-100	7877	NR	national	household availability/budget survey	multi-stage sampling
Ireland	Irish Community Health and Nutrition Survey	1999	20-100	7644	NR	national	household availability/budget survey	multi-stage sampling
Ireland	North South Ireland Food Consumption Survey (NSIFCS)	1997-1999	20-64	1373	NR	national	multiple short term diet recall	multi-stage sampling
Israel	Mabat First Israeli National Health and Nutrition Survey	1999-2001	25-34	3240	52.5	national	single short-term diet recall	multi-stage sampling
Israel	Mabat National Health and Nutrition Survey of the Elderly (Zahav)	2005-2006	65-74	1782	53.3	national	single short-term diet recall	multi-stage sampling
Italy	Italian Community Health and Nutrition Survey	1990	20-100	33172	NR	national	household availability/budget survey	multi-stage sampling
Italy	Italian Community Health and Nutrition Survey	1993	20-100	34273	NR	national	household availability/budget survey	multi-stage sampling
Italy	Italian Community Health and Nutrition Survey	1996	20-100	22740	NR	national	household availability/budget survey	multi-stage sampling
Italy	INN-CA 1994-96	1994-1996	20-100	1617	55.5	national	multiple short term diet recall	multi-stage sampling
Italy	INRAN-SCAI 2005-06	2005-2006	20-100	2777	55.4	national	multiple short term diet recall	multi-stage sampling
Jamaica	Social and dietary determinants of body max index of adult Jamaicans	1993-1995	25-74	922	60.7	national	food-frequency questionnaire	multi-stage sampling
Japan	National Nutrition Survey (in Japan)	1995	20-100	10766	53.8	national	single short-term diet recall	multi-stage sampling
Japan	National Nutrition Survey (in Japan)	1998	20-100	10917	53.6	national	single short-term diet recall	multi-stage sampling
Japan	The Japan Public Health Center-based Prospective Study (JPHC Study)	1995-1998	45-74	97768	53.4	sub-regional	food-frequency questionnaire	multi-stage sampling

Country	Study name	Time period	Age range (years)	Sample size	Percent female	Coverage	Diet assessment method	Sampling method
Japan	The Japan Public Health Center-based Prospective Study (JPHC Study)	2000-2003	50-79	92302	53.3	sub-regional	food-frequency questionnaire	multi-stage sampling
Latvia	Latvian Community Health and Nutrition Survey	2002	20-100	3949	NR	national	household availability/budget survey	multi-stage sampling
Latvia	Latvian Community Health and Nutrition Survey	2003	20-100	3631	NR	national	household availability/budget survey	one-stage sampling
Latvia	Latvian Community Health and Nutrition Survey	2004	20-100	3913	NR	national	household availability/budget survey	one-stage sampling
Lebanon	National survey: Behavioral risk factor survey, Lebanon: Comparison estimates based on cell phone interviews with face-to face interviews. Nutrition Component: Socioeconomic and dietary determinants of obesity in Lebanon	2008-2009	20-100	2592	53.4	national	food-frequency questionnaire	one-stage sampling
Luxambourg	Luxembourgian Community Health and Nutrition Survey	1993	20-100	3008	NR	national	household availability/budget survey	multi-stage sampling
Malaysia	Malaysian Adult Nutrition Survey 2003	2002-2003	20-59	6857	51.7	national	food-frequency questionnaire	one-stage sampling
Malta	Maltese Community Health and Nutrition Survey	1994	20-100	2722	NR	national	household availability/budget survey	quota sampling
Malta	Maltese Community Health and Nutrition Survey	1995	20-100	2748	NR	national	household availability/budget survey	multi-stage sampling
Malta	Maltese Community Health and Nutrition Survey	2000	20-100	2586	NR	national	household availability/budget survey	multi-stage sampling
Mexico	National Health and Nutrition Survey 2006 (Mexico)	2005-2006	20-59	15186	62.5	national	food-frequency questionnaire	multi-stage sampling
Montenegro	Montenegrin Community Health and Nutrition Survey	2003	20-100	380	NR	national	household availability/budget survey	multi-stage sampling
Montenegro	Montenegrin Community Health and Nutrition Survey	2004	20-100	380	NR	national	household availability/budget survey	one-stage sampling
Netherlands	Dutch National Food Consumption Survey	1992	20-92	2426	NR	national	multiple short term diet recall	multi-stage sampling
Netherlands	Dutch National Food Consumption Survey	2003	20-31	591	44.8	national	multiple short term diet recall	multi-stage sampling
Netherlands	Dutch National Food Consumption Survey	1987-1988	20-82	4112	53.9	national	multiple short term diet recall	multi-stage sampling
Netherlands	Dutch National Food Consumption Survey	1997-1998	20-89	4612	55.1	national	multiple short term diet recall	multi-stage sampling
New Zealand	NZ National Nutrition Survey 1997	1997	20-100	4289	58.7	national	food-frequency questionnaire	one-stage sampling

Country	Study name	Time period	Age range (years)	Sample size	Percent female	Coverage	Diet assessment method	Sampling method
Norway	Norwegian national dietary survey 1997 (Norkost 1997)	1997	20-79	2494	51.6	national	food-frequency questionnaire	multi-stage sampling
Norway	Norwegian Community Health and Nutrition Survey	1986-1988	20-100	7724	NR	national	household availability/budget survey	multi-stage sampling
Norway	Norwegian Community Health and Nutrition Survey	1992-1994	20-100	6518	NR	national	household availability/budget survey	multi-stage sampling
Norway	Norwegian Community Health and Nutrition Survey	1996-1998	20-100	6504	NR	national	household availability/budget survey	multi-stage sampling
Poland	Polish Community Health and Nutrition Survey	1988	20-100	29664	NR	national	household availability/budget survey	one-stage sampling
Poland	Household Food Consumption and Anthropometric Survey	2000	20-100	2821	55.8	national	single short-term diet recall	one-stage sampling
Poland	Pol-MONICA bis Warsaw Project	2001	20-74	1329	50.5	regional	single short-term diet recall	multi-stage sampling
Poland	WOBASZ - National Multicenter Health Survey	2003-2005	20-74	6661	53.0	national	single short-term diet recall	one-stage sampling
Portugal	Portuguese Community Health and Nutrition Survey	1990	20-100	12403	NR	national	household availability/budget survey	multi-stage sampling
Portugal	Portuguese Community Health and Nutrition Survey	1995	20-100	10554	NR	national	household availability/budget survey	one-stage sampling
Portugal	Portuguese Community Health and Nutrition Survey	2000	20-100	10020	NR	national	household availability/budget survey	one-stage sampling
Republic of Korea	Korea National Health and Nutrition Examination Survey (KNHANES) III	2005	20-100	6440	55.3	national	single short-term diet recall	multi-stage sampling
Serbia	Serbian Community Health and Nutrition Survey	2003	20-100	4800	NR	national	household availability/budget survey	one-stage sampling
Serbia	Serbian Community Health and Nutrition Survey	2004	20-100	4800	NR	national	household availability/budget survey	one-stage sampling
Singapore	National Nutrition Survey 1998	1998	20-69	2270	53.9	national	food-frequency questionnaire	one-stage sampling
Singapore	National Nutrition Survey 2004	2004	20-69	1314	50.2	national	food-frequency questionnaire	one-stage sampling
Slovakia	Slovakian Community Health and Nutrition Survey	1997	20-100	1671	NR	national	household availability/budget survey	quota sampling
Slovakia	Slovakian Community Health and Nutrition Survey	2000	20-100	1647	NR	national	household availability/budget survey	quota sampling

Country	Study name	Time period	Age range (years)	Sample size	Percent female	Coverage	Diet assessment method	Sampling method
Slovakia	Slovakian Community Health and Nutrition Survey	2003	20-100	1645	NR	national	household availability/budget survey	quota sampling
Slovakia	Monitoring of the nutritional status of particular groups of adult population	2006	20-54	2208	NR	national	single short-term diet recall	multi-stage sampling
Slovenia	Slovenian Community Health and Nutrition Survey	1998	20-100	4979	NR	national	household availability/budget survey	multi-stage sampling
Slovenia	Slovenian Community Health and Nutrition Survey	2000	20-100	4904	NR	national	household availability/budget survey	one-stage sampling
Slovenia	Slovenian Community Health and Nutrition Survey	2002	20-100	3687	NR	national	household availability/budget survey	one-stage sampling
South Africa	Food and nutrient availability in South African Households	1995	20-99	918	54.7	national	food-frequency questionnaire	one-stage sampling
Spain	Spanish Community Health and Nutrition Survey	1980-1981	20-100	30331	NR	national	household availability/budget survey	one-stage sampling
Spain	Spanish Community Health and Nutrition Survey	1990-1991	20-100	30331	NR	national	household availability/budget survey	one-stage sampling
Spain	Spanish Community Health and Nutrition Survey	1998-1999	20-100	14644	NR	national	household availability/budget survey	multi-stage sampling
Sri Lanka	Abdominal obesity and its association with selected risk factors of coronary heart disease in an adult population in the district of Colombo; MD Thesis, University of Colombo, Sri Lanka	2004	20-64	1400	48.6	regional	food-frequency questionnaire	multi-stage sampling
Sweden	Dietary habits and nutrient intake in Sweden 1989	1989	20-74	1513	50.9	national	single short-term diet recall	multi-stage sampling
Sweden	Swedish Community Health and Nutrition Survey	1989	20-100	2970	NR	national	household availability/budget survey	multi-stage sampling
Sweden	Swedish Community Health and Nutrition Survey	1996	20-74	2026	NR	national	household availability/budget survey	multi-stage sampling
Sweden	Dietary habits and nutrient intake in Sweden 1997-98	1997-1998	20-79	1173	51.8	national	single short-term diet recall	multi-stage sampling
Switzerland	Etude Bus Santé	2004-2009	35-74	3319	51.1	sub-regional	food-frequency questionnaire	multi-stage sampling
Taiwan	2005-2008 Nutrition and Health Survey in Taiwan	2005-2008	20-100	2908	50.3	national	single short-term diet recall	multi-stage sampling
Thailand	National Food and Nutrition Survey, Thailand	2003	20-59	1610	NR	national	single short-term diet recall	multi-stage sampling
Turkey	Dietary intake of adult population living in Ankara	2005	20-84	1484	76.5	sub-regional	single short-term diet recall	multi-stage sampling

Country	Study name	Time period	Age range (years)	Sample size	Percent female	Coverage	Diet assessment method	Sampling method
United Kingdom	Dietary and Nutritional Survey of British Adults	1986-1987	20-64	2197	50.5	national	single short-term diet recall	multi-stage sampling
United Kingdom	National Diet and Nutrition Survey: people aged 65 years and over	1994-1995	65-100	1275	50.4	national	single short-term diet recall	one-stage sampling
United Kingdom	National Diet and Nutrition Survey: adults aged 19-64 years	2000-2001	20-64	1724	55.6	national	single short-term diet recall	multi-stage sampling
United States of America	NHANES 2003-2006	2003-2006	20-85	17150	52.4	national	multiple short term diet recall	multi-stage sampling

Table B. Country-level mean consumption of fruit juice, milk, and sugar-sweetened beverages for men and women in 2010.

Country	Sex	Midpoint of age range	Mean juice intake (servings/day)	Juice, lower uncertainty interval	Juice, upper uncertainty interval	Mean milk intake (servings/day)	Milk, lower uncertainty interval	Milk, upper uncertainty interval	Mean SSB intake (servings/day)	SSB, lower uncertainty interval	SSB, upper uncertainty interval	Mean calcium intake (servings/day)	Calcium, lower uncertainty interval	Calcium, upper uncertainty interval
Afghanistan	F	25	0.039	0.018	0.072	0.34	0.19	0.56	0.63	0.35	1.05	572.8	444.2	729.6
Afghanistan	F	35	0.033	0.015	0.059	0.32	0.18	0.54	0.48	0.27	0.80	576.7	445.5	731.5
Afghanistan	F	45	0.027	0.012	0.048	0.31	0.17	0.51	0.35	0.19	0.59	581.8	447.1	735.6
Afghanistan	F	55	0.026	0.012	0.046	0.34	0.19	0.57	0.28	0.16	0.48	596.5	461.7	756.2
Afghanistan	F	65	0.026	0.012	0.048	0.38	0.20	0.64	0.23	0.13	0.40	613.1	475.1	785.3
Afghanistan	F	75	0.025	0.011	0.047	0.41	0.22	0.69	0.21	0.12	0.36	628.7	485.5	805.5
Afghanistan	F	90	0.025	0.011	0.047	0.44	0.24	0.74	0.19	0.11	0.33	646.0	497.1	826.6
Afghanistan	M	25	0.032	0.015	0.064	0.32	0.18	0.54	0.71	0.40	1.19	523.8	396.0	672.1
Afghanistan	M	35	0.027	0.012	0.052	0.30	0.17	0.49	0.54	0.30	0.89	527.0	399.1	673.5
Afghanistan	M	45	0.022	0.010	0.042	0.28	0.15	0.48	0.37	0.21	0.61	531.4	403.1	680.7
Afghanistan	M	55	0.021	0.010	0.041	0.31	0.17	0.50	0.30	0.17	0.50	544.5	413.4	697.0
Afghanistan	M	65	0.021	0.010	0.043	0.35	0.19	0.58	0.25	0.14	0.42	559.5	424.9	713.3
Afghanistan	M	75	0.021	0.010	0.041	0.38	0.21	0.63	0.23	0.13	0.38	573.6	437.5	728.3
Afghanistan	M	90	0.020	0.010	0.039	0.41	0.23	0.68	0.21	0.12	0.36	589.0	451.2	753.9
Angola	F	25	0.112	0.053	0.205	0.66	0.34	1.14	1.36	0.71	2.36	507.8	383.2	664.5
Angola	F	35	0.094	0.046	0.166	0.62	0.32	1.03	1.02	0.53	1.78	511.4	386.9	665.6
Angola	F	45	0.076	0.037	0.139	0.58	0.29	0.97	0.70	0.36	1.29	516.0	390.8	686.0
Angola	F	55	0.073	0.036	0.131	0.64	0.32	1.08	0.56	0.29	0.98	529.2	403.8	686.2
Angola	F	65	0.073	0.036	0.135	0.71	0.35	1.23	0.46	0.23	0.78	544.3	415.1	711.4
Angola	F	75	0.071	0.035	0.131	0.77	0.39	1.30	0.41	0.21	0.71	558.3	428.2	726.4
Angola	F	90	0.069	0.034	0.128	0.83	0.43	1.40	0.38	0.20	0.65	573.9	437.5	744.8
Angola	M	25	0.090	0.043	0.174	0.59	0.30	1.03	1.52	0.79	2.71	462.7	349.8	608.9
Angola	M	35	0.075	0.036	0.139	0.55	0.29	0.94	1.13	0.61	1.96	466.1	353.5	609.1
Angola	M	45	0.061	0.028	0.116	0.52	0.27	0.91	0.77	0.41	1.39	470.5	355.5	613.6
Angola	M	55	0.059	0.028	0.109	0.57	0.30	0.97	0.61	0.33	1.07	482.3	364.1	634.5
Angola	M	65	0.059	0.028	0.110	0.63	0.33	1.08	0.50	0.26	0.87	495.7	373.6	649.3
Angola	M	75	0.058	0.027	0.106	0.69	0.37	1.17	0.45	0.24	0.78	508.3	384.3	667.1
Angola	M	90	0.056	0.026	0.102	0.74	0.40	1.29	0.42	0.23	0.72	522.3	398.0	685.1
Albania	F	25	0.217	0.107	0.398	1.02	0.54	1.67	0.33	0.19	0.56	670.3	525.3	857.5
Albania	F	35	0.180	0.089	0.334	0.96	0.52	1.59	0.25	0.14	0.41	673.4	526.3	862.2
Albania	F	45	0.146	0.071	0.272	0.92	0.51	1.55	0.17	0.09	0.28	678.0	529.1	869.7
Albania	F	55	0.137	0.068	0.256	1.03	0.55	1.74	0.14	0.08	0.23	695.2	540.8	890.6
Albania	F	65	0.134	0.067	0.249	1.16	0.63	1.96	0.11	0.06	0.20	715.3	551.9	911.4
Albania	F	75	0.132	0.067	0.242	1.26	0.69	2.10	0.10	0.06	0.18	734.2	569.9	933.1
Albania	F	90	0.129	0.065	0.242	1.37	0.75	2.33	0.10	0.05	0.16	756.4	588.1	960.0
Albania	M	25	0.175	0.084	0.325	0.93	0.50	1.55	0.38	0.21	0.64	616.2	467.9	790.9
Albania	M	35	0.144	0.071	0.264	0.87	0.47	1.41	0.28	0.16	0.48	617.1	471.4	788.5
Albania	M	45	0.116	0.056	0.210	0.83	0.45	1.34	0.19	0.10	0.32	619.7	472.7	801.4
Albania	M	55	0.111	0.054	0.199	0.93	0.50	1.49	0.15	0.08	0.25	634.4	484.4	815.6
Albania	M	65	0.110	0.054	0.197	1.05	0.56	1.70	0.12	0.07	0.20	652.1	498.0	845.3
Albania	M	75	0.109	0.054	0.193	1.14	0.62	1.84	0.11	0.06	0.18	670.4	514.9	861.4
Albania	M	90	0.108	0.053	0.197	1.24	0.67	2.01	0.10	0.06	0.17	691.3	534.4	885.8
Andorra	F	25	0.439	0.239	0.777	0.77	0.44	1.26	0.84	0.48	1.40	898.8	696.6	1143.3
Andorra	F	35	0.352	0.189	0.625	0.73	0.41	1.19	0.60	0.34	0.99	905.5	705.4	1155.6
Andorra	F	45	0.274	0.145	0.480	0.69	0.39	1.14	0.38	0.21	0.62	913.7	705.6	1173.3
Andorra	F	55	0.266	0.143	0.463	0.74	0.42	1.22	0.30	0.17	0.49	933.7	727.0	1193.2
Andorra	F	65	0.272	0.144	0.477	0.80	0.46	1.33	0.26	0.15	0.42	956.6	744.1	1223.7
Andorra	F	75	0.271	0.144	0.475	0.88	0.51	1.46	0.24	0.14	0.39	980.9	765.9	1255.0
Andorra	F	90	0.269	0.143	0.466	0.99	0.56	1.63	0.22	0.12	0.36	1011.8	787.8	1296.5
Andorra	M	25	0.353	0.188	0.629	0.74	0.43	1.25	0.97	0.54	1.67	824.5	636.0	1040.2
Andorra	M	35	0.286	0.151	0.508	0.68	0.39	1.14	0.69	0.38	1.17	827.9	645.3	1045.0
Andorra	M	45	0.226	0.118	0.393	0.62	0.36	1.05	0.42	0.23	0.71	832.9	646.6	1057.9
Andorra	M	55	0.214	0.114	0.377	0.66	0.38	1.12	0.33	0.18	0.55	850.8	660.1	1075.0
Andorra	M	65	0.213	0.113	0.386	0.73	0.42	1.24	0.27	0.15	0.47	871.6	675.6	1109.2
Andorra	M	75	0.216	0.116	0.383	0.81	0.46	1.38	0.25	0.14	0.43	893.5	691.9	1132.6
Andorra	M	90	0.219	0.115	0.376	0.90	0.52	1.56	0.23	0.13	0.40	920.1	721.4	1157.3
United Arab Emirates	F	25	0.384	0.192	0.727	0.68	0.38	1.16	0.80	0.43	1.38	672.5	514.9	857.1
United Arab Emirates	F	35	0.321	0.157	0.601	0.65	0.37	1.10	0.62	0.33	1.05	677.2	519.3	859.5
United Arab Emirates	F	45	0.263	0.127	0.486	0.62	0.35	1.05	0.44	0.24	0.74	683.1	517.2	865.9
United Arab Emirates	F	55	0.253	0.123	0.464	0.68	0.38	1.14	0.36	0.20	0.60	700.0	534.5	886.7
United Arab Emirates	F	65	0.253	0.123	0.463	0.76	0.42	1.28	0.30	0.16	0.51	719.1	557.4	908.7
United Arab Emirates	F	75	0.248	0.122	0.456	0.82	0.46	1.35	0.27	0.15	0.45	738.2	568.6	925.2
United Arab Emirates	F	90	0.240	0.118	0.459	0.89	0.50	1.45	0.25	0.13	0.41	760.3	580.3	956.4
United Arab Emirates	M	25	0.315	0.154	0.578	0.63	0.33	1.10	0.90	0.51	1.49	618.7	476.4	789.8
United Arab Emirates	M	35	0.263	0.132	0.484	0.59	0.31	1.04	0.68	0.38	1.11	622.4	485.9	793.5
United Arab Emirates	M	45	0.215	0.109	0.403	0.56	0.30	0.97	0.47	0.26	0.78	627.5	492.2	805.9
United Arab Emirates	M	55	0.207	0.105	0.385	0.62	0.33	1.06	0.38	0.21	0.62	642.9	504.0	821.7
United Arab Emirates	M	65	0.208	0.103	0.393	0.69	0.36	1.21	0.32	0.18	0.53	660.4	516.6	846.4
United Arab Emirates	M	75	0.202	0.101	0.376	0.75	0.40	1.30	0.29	0.16	0.48	677.5	530.1	869.4
United Arab Emirates	M	90	0.194	0.097	0.357	0.82	0.43	1.41	0.27	0.15	0.43	696.5	544.4	890.5
Argentina	F	25	0.402	0.197	0.724	0.53	0.45	0.62	1.06	0.89	1.24	545.7	507.7	584.6
Argentina	F	35	0.333	0.167	0.591	0.49	0.44	0.55	0.80	0.70	0.90	548.9	523.0	576.0
Argentina	F	45	0.271	0.136	0.484	0.47	0.40	0.55	0.56	0.46	0.65	553.3	519.6	589.6
Argentina	F	55	0.260	0.132	0.461	0.52	0.46	0.58	0.44	0.38	0.50	567.0	541.2	595.5
Argentina	F	65	0.260	0.131	0.460	0.58	0.50	0.67	0.35	0.30	0.41	582.8	546.0	623.7
Argentina	F	75	0.255	0.128	0.450	0.63	0.56	0.70	0.32	0.28	0.37	598.1	568.4	631.0
Argentina	F	90	0.247	0.121	0.428	0.69	0.61	0.76	0.29	0.26	0.34	616.8	587.9	647.8
Argentina	M	25	0.327	0.166	0.588	0.49	0.42	0.57	1.17	0.98	1.39	502.8	470.8	539.1
Argentina	M	35	0.272	0.139	0.494	0.46	0.41	0.51	0.88	0.76	1.00	505.7	480.8	531.8
Argentina	M	45	0.221	0.113	0.412	0.44	0.37	0.51	0.60	0.50	0.71	509.7	475.4	546.2
Argentina	M	55	0.212	0.109	0.380	0.48	0.43	0.54	0.47	0.41	0.55	522.2	495.6	550.1
Argentina	M	65	0.212	0.109	0.371	0.54	0.46	0.61	0.39	0.33	0.45	536.6	501.7	571.1
Argentina	M	75	0.208	0.110	0.364	0.58	0.52	0.64	0.35	0.30	0.41	551.0	524.7	579.2
Argentina	M	90	0.202	0.106	0.361	0.63	0.56	0.70	0.33	0.29	0.37	568.0	541.6	596.0
Armenia	F	25	0.304	0.142	0.568	0.48	0.33	0.68	0.55	0.29	0.95	679.2	516.7	853.7
Armenia	F	35	0.252	0.121	0.461	0.45	0.31	0.62	0.41	0.22	0.68	683.4	523.7	855.1
Armenia	F	45	0.204	0.097	0.373	0.43	0.29	0.60	0.28	0.14	0.46	689.1	530.2	871.7
Armenia	F	55	0.197	0.096	0.360	0.47	0.32	0.65	0.22	0.11	0.36	705.4	545.3	886.9
Armenia	F	65	0.197	0.093	0.350	0.52	0.36	0.73	0.18	0.09	0.30	724.3	555.1	916.0
Armenia	F	75	0.193	0.092	0.342	0.57	0.39	0.78	0.17	0.09	0.27	743.6	569.4	937.0
Armenia	F	90	0.186	0.090	0.336	0.62	0.42	0.86	0.15	0.08	0.25	767.1	593.8	967.4
Armenia	M	25	0.250	0.123	0.454	0.44	0.30	0.63	0.60	0.33	1.05	616.1	470.6	784.3
Armenia	M	35	0.207	0.104	0.378	0.42	0.28	0.59	0.45	0.25	0.77	620.0	476.1	791.7
Armenia	M	45	0.168	0.081	0.308	0.39	0.26	0.56	0.30	0.17	0.52	625.4	481.2	806.3
Armenia	M	55	0.161	0.078	0.296	0.43	0.29	0.61	0.24	0.13	0.40	640.8	496.2	813.8
Armenia	M	65	0.161	0.076	0.302	0.48	0.32	0.69	0.20	0.11	0.34	658.7	502.2	846.0

Country	Sex	Midpoint of age range	Mean juice intake (servings/day)	Juice, lower uncertainty interval	Juice, upper uncertainty interval	Mean milk intake (servings/day)	Milk, lower uncertainty interval	Milk, upper uncertainty interval	Mean SSB intake (servings/day)	SSB, lower uncertainty interval	SSB, upper uncertainty interval	Mean calcium intake (servings/day)	Calcium, lower uncertainty interval	Calcium, upper uncertainty interval
Armenia	M	75	0.157	0.076	0.289	0.52	0.36	0.74	0.18	0.10	0.31	676.2	518.0	869.8
Armenia	M	90	0.153	0.077	0.276	0.57	0.39	0.81	0.17	0.09	0.28	696.3	538.0	902.4
Antigua and Barbuda	F	25	0.413	0.197	0.738	1.13	0.61	1.91	2.98	1.59	5.05	909.2	697.8	1157.0
Antigua and Barbuda	F	35	0.345	0.168	0.606	1.06	0.59	1.78	2.22	1.23	3.72	914.5	701.1	1164.2
Antigua and Barbuda	F	45	0.283	0.142	0.497	1.01	0.55	1.72	1.51	0.82	2.54	921.6	704.8	1177.6
Antigua and Barbuda	F	55	0.270	0.136	0.479	1.10	0.60	1.86	1.21	0.67	2.06	944.1	726.5	1206.1
Antigua and Barbuda	F	65	0.267	0.132	0.483	1.22	0.67	2.05	1.01	0.55	1.73	969.9	750.6	1233.6
Antigua and Barbuda	F	75	0.261	0.128	0.471	1.33	0.73	2.20	0.92	0.50	1.58	995.6	769.7	1256.5
Antigua and Barbuda	F	90	0.254	0.122	0.452	1.45	0.79	2.45	0.84	0.47	1.42	1026.8	791.2	1298.7
Antigua and Barbuda	M	25	0.336	0.162	0.605	1.04	0.55	1.74	3.22	1.75	5.37	833.8	637.5	1079.1
Antigua and Barbuda	M	35	0.279	0.139	0.495	0.97	0.52	1.61	2.38	1.32	3.98	838.4	648.9	1085.5
Antigua and Barbuda	M	45	0.229	0.112	0.419	0.92	0.49	1.52	1.61	0.90	2.71	844.8	651.1	1085.4
Antigua and Barbuda	M	55	0.220	0.110	0.410	1.00	0.54	1.66	1.28	0.72	2.16	865.2	668.3	1117.3
Antigua and Barbuda	M	65	0.219	0.107	0.404	1.12	0.60	1.88	1.07	0.60	1.84	888.8	689.1	1143.4
Antigua and Barbuda	M	75	0.213	0.105	0.392	1.21	0.65	2.01	0.98	0.54	1.64	912.6	709.1	1167.8
Antigua and Barbuda	M	90	0.205	0.100	0.377	1.31	0.71	2.19	0.90	0.49	1.49	941.1	733.1	1212.2
Australia	F	25	0.759	0.400	1.360	0.68	0.59	0.80	0.88	0.72	1.05	734.9	685.5	786.4
Australia	F	35	0.629	0.332	1.101	0.64	0.56	0.73	0.66	0.56	0.77	740.9	698.9	782.3
Australia	F	45	0.512	0.259	0.899	0.61	0.52	0.71	0.45	0.37	0.54	748.3	701.5	800.8
Australia	F	55	0.493	0.255	0.852	0.68	0.59	0.76	0.36	0.31	0.43	766.5	723.9	811.3
Australia	F	65	0.493	0.259	0.858	0.76	0.65	0.87	0.31	0.26	0.36	787.1	733.7	841.0
Australia	F	75	0.483	0.254	0.828	0.82	0.72	0.93	0.28	0.24	0.33	807.3	759.8	855.8
Australia	F	90	0.467	0.247	0.809	0.90	0.79	1.03	0.25	0.21	0.30	833.0	784.1	882.3
Australia	M	25	0.600	0.296	1.081	0.63	0.53	0.73	0.97	0.80	1.16	671.6	627.1	719.2
Australia	M	35	0.498	0.255	0.876	0.58	0.51	0.67	0.72	0.62	0.84	676.4	639.0	715.4
Australia	M	45	0.405	0.201	0.734	0.55	0.47	0.65	0.50	0.41	0.60	682.5	639.4	730.2
Australia	M	55	0.390	0.197	0.700	0.61	0.54	0.70	0.40	0.34	0.47	699.9	662.4	741.9
Australia	M	65	0.390	0.193	0.686	0.68	0.59	0.80	0.33	0.27	0.39	719.6	674.5	765.4
Australia	M	75	0.382	0.188	0.671	0.75	0.65	0.85	0.30	0.25	0.35	738.9	699.1	781.2
Australia	M	90	0.369	0.185	0.657	0.82	0.72	0.92	0.28	0.24	0.32	762.5	717.7	804.8
Austria	F	25	0.897	0.609	1.273	0.48	0.34	0.67	0.81	0.54	1.16	783.9	722.4	849.6
Austria	F	35	0.720	0.495	1.020	0.45	0.32	0.63	0.58	0.39	0.82	789.7	728.9	849.8
Austria	F	45	0.561	0.380	0.802	0.43	0.30	0.61	0.37	0.24	0.52	796.7	729.2	865.9
Austria	F	55	0.544	0.372	0.764	0.46	0.33	0.64	0.29	0.20	0.42	814.2	750.2	879.6
Austria	F	65	0.555	0.379	0.787	0.50	0.36	0.69	0.25	0.17	0.36	834.3	768.2	907.7
Austria	F	75	0.555	0.378	0.779	0.55	0.40	0.76	0.23	0.16	0.33	855.3	793.1	924.2
Austria	F	90	0.551	0.372	0.784	0.62	0.44	0.86	0.21	0.14	0.31	882.6	815.3	952.1
Austria	M	25	0.716	0.481	1.016	0.46	0.31	0.63	0.96	0.64	1.39	724.1	661.7	790.0
Austria	M	35	0.580	0.392	0.817	0.42	0.29	0.58	0.67	0.45	0.97	727.1	671.4	787.6
Austria	M	45	0.458	0.309	0.646	0.38	0.27	0.53	0.41	0.27	0.59	731.5	670.9	796.3
Austria	M	55	0.434	0.295	0.610	0.41	0.28	0.56	0.32	0.21	0.46	747.1	689.5	811.2
Austria	M	65	0.433	0.294	0.608	0.45	0.31	0.62	0.27	0.18	0.39	765.5	707.4	835.8
Austria	M	75	0.437	0.298	0.608	0.49	0.35	0.68	0.25	0.16	0.36	784.7	728.7	850.1
Austria	M	90	0.443	0.301	0.621	0.55	0.38	0.76	0.23	0.15	0.33	808.3	746.0	878.9
Azerbaijan	F	25	0.033	0.016	0.061	0.43	0.23	0.72	0.17	0.08	0.29	639.1	486.1	831.1
Azerbaijan	F	35	0.027	0.013	0.050	0.40	0.22	0.68	0.13	0.06	0.21	642.9	493.3	830.1
Azerbaijan	F	45	0.022	0.010	0.042	0.38	0.20	0.64	0.09	0.04	0.15	648.0	497.5	843.3
Azerbaijan	F	55	0.021	0.010	0.039	0.42	0.22	0.69	0.07	0.04	0.12	663.4	510.3	856.2
Azerbaijan	F	65	0.021	0.010	0.039	0.47	0.25	0.76	0.06	0.03	0.10	681.4	519.5	879.5
Azerbaijan	F	75	0.021	0.010	0.038	0.51	0.27	0.83	0.05	0.03	0.09	699.7	535.8	906.5
Azerbaijan	F	90	0.020	0.010	0.037	0.55	0.29	0.92	0.05	0.03	0.08	720.0	554.7	932.3
Azerbaijan	M	25	0.027	0.013	0.051	0.39	0.23	0.67	0.19	0.10	0.33	585.8	441.5	743.0
Azerbaijan	M	35	0.023	0.011	0.042	0.37	0.21	0.61	0.14	0.08	0.24	589.5	446.0	742.2
Azerbaijan	M	45	0.018	0.008	0.034	0.35	0.20	0.58	0.10	0.05	0.17	594.6	451.5	754.8
Azerbaijan	M	55	0.018	0.008	0.033	0.39	0.22	0.64	0.08	0.04	0.13	609.2	464.9	767.1
Azerbaijan	M	65	0.018	0.008	0.034	0.43	0.25	0.72	0.06	0.03	0.11	626.1	476.2	788.3
Azerbaijan	M	75	0.017	0.008	0.032	0.47	0.26	0.78	0.06	0.03	0.10	642.9	491.7	809.1
Azerbaijan	M	90	0.017	0.008	0.031	0.50	0.28	0.84	0.05	0.03	0.09	660.9	507.6	837.8
Burundi	F	25	0.088	0.036	0.176	0.10	0.05	0.17	0.30	0.15	0.52	340.5	256.3	438.4
Burundi	F	35	0.073	0.031	0.147	0.10	0.05	0.16	0.22	0.12	0.38	342.5	260.1	442.7
Burundi	F	45	0.059	0.025	0.118	0.09	0.05	0.15	0.15	0.08	0.25	345.4	260.9	448.6
Burundi	F	55	0.057	0.025	0.115	0.10	0.05	0.17	0.12	0.06	0.20	354.1	269.9	460.1
Burundi	F	65	0.057	0.024	0.117	0.11	0.06	0.19	0.10	0.05	0.17	364.1	277.4	475.1
Burundi	F	75	0.056	0.024	0.114	0.12	0.07	0.20	0.09	0.05	0.15	373.6	286.2	484.0
Burundi	F	90	0.054	0.023	0.111	0.13	0.07	0.22	0.08	0.04	0.14	384.3	294.7	496.9
Burundi	M	25	0.072	0.032	0.144	0.09	0.05	0.16	0.34	0.18	0.59	307.5	237.1	397.4
Burundi	M	35	0.059	0.026	0.117	0.09	0.05	0.15	0.25	0.14	0.43	309.5	239.8	394.9
Burundi	M	45	0.048	0.020	0.094	0.08	0.04	0.14	0.17	0.09	0.30	312.2	239.7	400.5
Burundi	M	55	0.046	0.020	0.091	0.09	0.05	0.16	0.14	0.07	0.24	319.9	246.2	407.9
Burundi	M	65	0.046	0.020	0.092	0.10	0.05	0.18	0.11	0.06	0.20	328.5	252.8	421.5
Burundi	M	75	0.045	0.020	0.089	0.11	0.06	0.19	0.10	0.06	0.18	337.1	260.2	430.3
Burundi	M	90	0.044	0.019	0.087	0.12	0.06	0.20	0.09	0.05	0.16	346.8	268.0	443.4
Belgium	F	25	0.457	0.346	0.591	0.35	0.29	0.42	1.31	1.05	1.63	772.7	718.8	830.1
Belgium	F	35	0.367	0.283	0.462	0.33	0.28	0.39	0.94	0.76	1.16	778.4	728.5	829.8
Belgium	F	45	0.286	0.222	0.363	0.31	0.26	0.38	0.59	0.47	0.74	785.4	727.9	846.9
Belgium	F	55	0.277	0.218	0.347	0.34	0.28	0.39	0.47	0.38	0.58	802.7	748.8	858.8
Belgium	F	65	0.283	0.220	0.355	0.36	0.31	0.43	0.41	0.32	0.51	822.3	760.7	880.2
Belgium	F	75	0.283	0.221	0.353	0.40	0.34	0.47	0.38	0.30	0.46	843.3	785.7	898.5
Belgium	F	90	0.281	0.217	0.353	0.45	0.37	0.53	0.35	0.28	0.43	869.6	811.1	929.0
Belgium	M	25	0.369	0.282	0.470	0.34	0.28	0.40	1.54	1.23	1.88	710.4	658.4	766.6
Belgium	M	35	0.299	0.230	0.373	0.31	0.26	0.36	1.08	0.87	1.32	713.3	667.4	761.9
Belgium	M	45	0.235	0.179	0.296	0.28	0.24	0.33	0.66	0.52	0.83	717.7	665.2	773.0
Belgium	M	55	0.223	0.173	0.278	0.30	0.25	0.35	0.52	0.41	0.63	733.1	686.4	781.8
Belgium	M	65	0.222	0.170	0.283	0.33	0.27	0.39	0.43	0.34	0.53	750.9	699.5	806.8
Belgium	M	75	0.225	0.175	0.284	0.36	0.31	0.43	0.39	0.31	0.48	769.9	720.9	821.8
Belgium	M	90	0.228	0.177	0.290	0.41	0.34	0.48	0.37	0.29	0.45	792.8	739.7	848.1
Benin	F	25	0.002	0.001	0.004	0.18	0.10	0.30	0.27	0.14	0.47	418.7	324.4	524.4
Benin	F	35	0.002	0.001	0.003	0.17	0.09	0.28	0.20	0.11	0.34	421.1	328.9	527.5
Benin	F	45	0.001	0.001	0.002	0.16	0.09	0.27	0.14	0.07	0.24	424.5	329.6	537.4
Benin	F	55	0.001	0.001	0.002	0.18	0.10	0.29	0.11	0.06	0.19	435.3	340.4	545.8
Benin	F	65	0.001	0.001	0.002	0.20	0.11	0.33	0.09	0.05	0.15	447.5	350.8	563.7
Benin	F	75	0.001	0.001	0.002	0.21	0.12	0.35	0.08	0.04	0.14	459.0	360.8	578.0
Benin	F	90	0.001	0.001	0.002	0.23	0.13	0.39	0.07	0.04	0.13	472.0	372.7	594.7
Benin	M	25	0.002	0.001	0.003	0.17	0.09	0.27	0.30	0.15	0.52	383.4	293.0	492.4
Benin	M	35	0.001	0.001	0.003	0.16	0.09	0.25	0.22	0.12	0.39	385.9	295.4	494.3
Benin	M	45	0.001	0.001	0.002	0.15	0.08	0.24	0.15	0.08	0.27	389.1	298.0	499.4
Benin	M	55	0.001	0.000	0.002	0.16	0.09	0.26	0.12	0.06	0.21	398.5	307.5	508.3
Benin	M	65	0.001	0.001	0.002	0.18	0.10	0.30	0.10	0.05	0.17	409.4	318.1	525.0
Benin	M	75	0.001	0.000	0.002	0.20	0.11	0.32	0.09	0.05	0.16	420.0	326.3	534.2

Country	Sex	Midpoint of age range	Mean juice intake (servings/day)	Juice, lower uncertainty interval	Juice, upper uncertainty interval	Mean milk intake (servings/day)	Milk, lower uncertainty interval	Milk, upper uncertainty interval	Mean SSB intake (servings/day)	SSB, lower uncertainty interval	SSB, upper uncertainty interval	Mean calcium intake (servings/day)	Calcium, lower uncertainty interval	Calcium, upper uncertainty interval
Benin	M	90	0.001	0.000	0.002	0.22	0.12	0.35	0.08	0.04	0.15	432.1	334.0	544.8
Burkina Faso	F	25	0.000	0.000	0.000	0.26	0.16	0.40	0.29	0.15	0.50	487.7	413.4	561.9
Burkina Faso	F	35	0.000	0.000	0.000	0.24	0.15	0.37	0.22	0.11	0.37	490.5	420.6	565.1
Burkina Faso	F	45	0.000	0.000	0.000	0.23	0.14	0.36	0.15	0.08	0.25	494.4	420.4	571.5
Burkina Faso	F	55	0.000	0.000	0.000	0.26	0.16	0.40	0.12	0.06	0.20	506.9	435.7	581.9
Burkina Faso	F	65	0.000	0.000	0.000	0.29	0.18	0.44	0.10	0.05	0.17	521.3	444.5	600.2
Burkina Faso	F	75	0.000	0.000	0.000	0.31	0.20	0.47	0.09	0.05	0.15	534.6	459.1	611.0
Burkina Faso	F	90	0.000	0.000	0.000	0.34	0.21	0.50	0.08	0.04	0.14	549.1	471.7	626.2
Burkina Faso	M	25	0.000	0.000	0.000	0.24	0.15	0.37	0.33	0.17	0.55	448.8	382.1	521.6
Burkina Faso	M	35	0.000	0.000	0.000	0.23	0.15	0.34	0.24	0.13	0.41	451.7	387.2	519.6
Burkina Faso	M	45	0.000	0.000	0.000	0.22	0.13	0.32	0.17	0.09	0.28	455.5	387.3	526.2
Burkina Faso	M	55	0.000	0.000	0.000	0.24	0.15	0.36	0.13	0.07	0.22	466.5	400.3	538.2
Burkina Faso	M	65	0.000	0.000	0.000	0.27	0.17	0.40	0.11	0.06	0.18	479.3	410.8	560.6
Burkina Faso	M	75	0.000	0.000	0.000	0.29	0.18	0.43	0.10	0.05	0.16	491.7	423.1	569.3
Burkina Faso	M	90	0.000	0.000	0.000	0.31	0.20	0.46	0.09	0.05	0.15	505.4	433.2	581.9
Bangladesh	F	25	0.003	0.001	0.006	0.11	0.08	0.14	0.26	0.13	0.44	348.8	310.2	390.4
Bangladesh	F	35	0.003	0.001	0.005	0.10	0.08	0.13	0.19	0.10	0.32	351.3	313.2	390.0
Bangladesh	F	45	0.002	0.001	0.004	0.10	0.08	0.13	0.13	0.07	0.22	354.5	315.3	396.6
Bangladesh	F	55	0.002	0.001	0.004	0.11	0.09	0.14	0.11	0.06	0.17	362.9	323.5	404.9
Bangladesh	F	65	0.002	0.001	0.004	0.12	0.09	0.16	0.09	0.05	0.14	372.7	329.8	417.4
Bangladesh	F	75	0.002	0.001	0.003	0.14	0.10	0.17	0.08	0.04	0.13	382.6	341.1	425.8
Bangladesh	F	90	0.002	0.001	0.003	0.15	0.11	0.19	0.07	0.04	0.12	394.3	353.0	437.2
Bangladesh	M	25	0.003	0.001	0.005	0.10	0.08	0.13	0.28	0.15	0.50	319.7	283.4	360.4
Bangladesh	M	35	0.002	0.001	0.004	0.10	0.07	0.12	0.21	0.11	0.37	321.8	287.6	359.6
Bangladesh	M	45	0.002	0.001	0.003	0.09	0.07	0.12	0.15	0.08	0.26	324.6	288.6	366.5
Bangladesh	M	55	0.002	0.001	0.003	0.10	0.08	0.13	0.12	0.06	0.20	332.7	296.8	372.3
Bangladesh	M	65	0.002	0.001	0.003	0.12	0.09	0.15	0.10	0.05	0.17	342.1	304.4	382.4
Bangladesh	M	75	0.002	0.001	0.003	0.12	0.10	0.16	0.09	0.05	0.15	351.2	313.7	391.2
Bangladesh	M	90	0.002	0.001	0.003	0.13	0.10	0.17	0.08	0.04	0.14	361.8	323.6	404.3
Bulgaria	F	25	0.207	0.144	0.292	0.68	0.56	0.83	0.43	0.32	0.57	573.2	526.9	622.2
Bulgaria	F	35	0.172	0.122	0.240	0.64	0.54	0.76	0.32	0.24	0.41	575.9	538.2	616.1
Bulgaria	F	45	0.140	0.095	0.198	0.62	0.51	0.74	0.21	0.16	0.28	579.9	536.7	623.7
Bulgaria	F	55	0.132	0.092	0.182	0.69	0.57	0.82	0.17	0.13	0.22	594.8	555.2	634.9
Bulgaria	F	65	0.129	0.089	0.178	0.77	0.64	0.94	0.15	0.11	0.19	611.7	567.0	658.3
Bulgaria	F	75	0.126	0.089	0.174	0.84	0.70	1.00	0.13	0.10	0.17	628.1	586.5	671.9
Bulgaria	F	90	0.123	0.087	0.168	0.92	0.76	1.09	0.12	0.09	0.16	647.1	603.6	693.0
Bulgaria	M	25	0.163	0.114	0.226	0.63	0.51	0.76	0.50	0.38	0.65	527.8	484.0	572.7
Bulgaria	M	35	0.135	0.096	0.183	0.59	0.48	0.70	0.37	0.29	0.48	528.5	490.5	568.1
Bulgaria	M	45	0.109	0.076	0.151	0.56	0.45	0.69	0.25	0.19	0.32	530.6	486.5	577.1
Bulgaria	M	55	0.104	0.074	0.142	0.63	0.52	0.75	0.20	0.15	0.25	543.4	502.4	582.6
Bulgaria	M	65	0.103	0.073	0.142	0.71	0.58	0.86	0.16	0.12	0.21	558.4	513.8	601.6
Bulgaria	M	75	0.102	0.073	0.139	0.77	0.64	0.92	0.14	0.11	0.19	574.2	532.5	614.7
Bulgaria	M	90	0.101	0.073	0.137	0.84	0.69	1.01	0.14	0.10	0.17	592.7	550.2	636.0
Bahrain	F	25	0.354	0.182	0.680	0.74	0.40	1.25	0.93	0.50	1.56	671.9	603.4	750.4
Bahrain	F	35	0.296	0.153	0.550	0.71	0.39	1.21	0.71	0.39	1.17	676.5	612.5	747.1
Bahrain	F	45	0.242	0.124	0.439	0.68	0.38	1.18	0.51	0.28	0.84	682.6	614.1	761.8
Bahrain	F	55	0.233	0.120	0.417	0.74	0.41	1.26	0.41	0.23	0.69	699.5	634.2	775.2
Bahrain	F	65	0.232	0.119	0.413	0.82	0.45	1.39	0.35	0.19	0.58	718.9	644.2	802.1
Bahrain	F	75	0.227	0.119	0.404	0.89	0.49	1.49	0.31	0.17	0.52	737.4	664.6	818.8
Bahrain	F	90	0.221	0.118	0.388	0.97	0.53	1.64	0.28	0.16	0.48	758.8	684.1	839.6
Bahrain	M	25	0.288	0.141	0.522	0.66	0.36	1.10	1.03	0.55	1.75	614.7	548.2	685.7
Bahrain	M	35	0.241	0.118	0.435	0.62	0.33	1.00	0.78	0.43	1.31	618.4	558.7	684.4
Bahrain	M	45	0.198	0.093	0.366	0.59	0.32	0.95	0.54	0.29	0.90	623.5	559.4	698.6
Bahrain	M	55	0.191	0.092	0.351	0.65	0.35	1.03	0.44	0.24	0.73	638.8	574.3	707.9
Bahrain	M	65	0.191	0.093	0.352	0.72	0.38	1.16	0.37	0.20	0.63	656.4	586.3	726.7
Bahrain	M	75	0.185	0.090	0.340	0.79	0.41	1.25	0.33	0.18	0.57	673.1	605.6	740.9
Bahrain	M	90	0.178	0.085	0.319	0.86	0.46	1.36	0.31	0.17	0.52	692.3	624.5	762.8
Bahamas	F	25	0.285	0.140	0.503	0.75	0.41	1.25	3.26	1.83	5.34	828.5	642.9	1074.8
Bahamas	F	35	0.238	0.120	0.419	0.70	0.39	1.17	2.42	1.38	3.91	833.1	644.7	1077.9
Bahamas	F	45	0.196	0.100	0.354	0.66	0.36	1.11	1.65	0.91	2.71	839.5	650.1	1089.7
Bahamas	F	55	0.186	0.095	0.330	0.73	0.40	1.19	1.32	0.74	2.18	859.9	670.3	1108.2
Bahamas	F	65	0.184	0.092	0.323	0.81	0.44	1.32	1.10	0.62	1.79	883.6	689.4	1141.0
Bahamas	F	75	0.180	0.090	0.317	0.87	0.49	1.42	1.00	0.57	1.64	907.0	711.5	1169.3
Bahamas	F	90	0.175	0.088	0.306	0.95	0.53	1.53	0.92	0.52	1.49	934.5	728.9	1198.7
Bahamas	M	25	0.231	0.122	0.405	0.70	0.38	1.21	3.55	1.91	5.93	756.9	584.2	970.0
Bahamas	M	35	0.193	0.103	0.341	0.66	0.37	1.12	2.62	1.41	4.31	761.2	593.6	976.5
Bahamas	M	45	0.158	0.083	0.288	0.62	0.34	1.07	1.77	0.95	2.94	767.1	597.5	992.9
Bahamas	M	55	0.152	0.081	0.270	0.68	0.38	1.17	1.41	0.77	2.30	785.5	611.7	1008.7
Bahamas	M	65	0.151	0.080	0.274	0.76	0.42	1.31	1.18	0.64	1.94	806.9	628.1	1034.3
Bahamas	M	75	0.148	0.078	0.265	0.82	0.45	1.40	1.08	0.59	1.75	828.4	643.2	1061.1
Bahamas	M	90	0.142	0.074	0.248	0.88	0.48	1.50	0.99	0.53	1.60	853.3	662.5	1090.4
Bosnia and Herzegovina	F	25	0.112	0.057	0.203	1.39	1.08	1.73	0.30	0.16	0.51	619.0	474.7	782.6
Bosnia and Herzegovina	F	35	0.093	0.047	0.166	1.31	1.03	1.61	0.22	0.12	0.37	621.9	479.1	783.9
Bosnia and Herzegovina	F	45	0.075	0.037	0.134	1.26	0.99	1.56	0.15	0.08	0.26	626.4	478.4	789.3
Bosnia and Herzegovina	F	55	0.071	0.036	0.126	1.40	1.10	1.73	0.12	0.07	0.21	642.2	490.1	804.3
Bosnia and Herzegovina	F	65	0.069	0.036	0.127	1.57	1.23	1.98	0.10	0.05	0.17	660.6	503.1	832.3
Bosnia and Herzegovina	F	75	0.068	0.035	0.124	1.71	1.36	2.14	0.09	0.05	0.16	678.2	513.9	848.9
Bosnia and Herzegovina	F	90	0.066	0.034	0.123	1.87	1.47	2.33	0.09	0.05	0.14	698.6	535.2	874.9
Bosnia and Herzegovina	M	25	0.089	0.046	0.157	1.28	1.00	1.63	0.36	0.19	0.62	569.7	432.8	720.5
Bosnia and Herzegovina	M	35	0.074	0.038	0.131	1.20	0.96	1.49	0.27	0.15	0.45	570.7	430.7	722.1
Bosnia and Herzegovina	M	45	0.060	0.030	0.108	1.15	0.91	1.44	0.18	0.10	0.30	573.2	428.9	731.7
Bosnia and Herzegovina	M	55	0.057	0.029	0.102	1.28	1.02	1.57	0.14	0.08	0.24	586.8	440.4	742.3
Bosnia and Herzegovina	M	65	0.057	0.028	0.100	1.45	1.12	1.79	0.11	0.06	0.20	602.9	456.1	768.1
Bosnia and Herzegovina	M	75	0.056	0.028	0.099	1.58	1.24	1.93	0.10	0.06	0.18	620.0	469.4	787.1
Bosnia and Herzegovina	M	90	0.056	0.028	0.097	1.71	1.36	2.11	0.10	0.05	0.16	639.3	481.9	806.6
Belarus	F	25	0.091	0.044	0.162	0.60	0.42	0.85	0.53	0.30	0.92	732.8	560.6	933.1
Belarus	F	35	0.075	0.038	0.134	0.57	0.40	0.79	0.40	0.23	0.68	739.0	564.0	938.9
Belarus	F	45	0.060	0.030	0.109	0.55	0.38	0.78	0.27	0.16	0.47	746.4	572.3	949.1
Belarus	F	55	0.058	0.029	0.103	0.60	0.43	0.85	0.22	0.13	0.36	763.5	581.2	964.3
Belarus	F	65	0.058	0.030	0.101	0.67	0.47	0.96	0.18	0.10	0.31	782.9	595.0	995.5
Belarus	F	75	0.057	0.029	0.099	0.72	0.51	1.02	0.16	0.09	0.28	802.8	615.1	1012.7
Belarus	F	90	0.055	0.027	0.096	0.78	0.56	1.10	0.15	0.09	0.26	827.0	637.4	1045.6
Belarus	M	25	0.076	0.039	0.138	0.54	0.37	0.73	0.58	0.31	0.98	662.7	512.3	846.5
Belarus	M	35	0.063	0.033	0.112	0.51	0.35	0.69	0.43	0.23	0.72	665.8	515.0	845.7
Belarus	M	45	0.051	0.027	0.089	0.49	0.34	0.66	0.30	0.16	0.51	670.6	515.4	857.7
Belarus	M	55	0.049	0.026	0.087	0.55	0.39	0.74	0.23	0.13	0.41	688.4	530.2	876.2
Belarus	M	65	0.049	0.025	0.087	0.63	0.44	0.86	0.19	0.11	0.34	708.9	546.0	906.4
Belarus	M	75	0.048	0.025	0.085	0.67	0.47	0.91	0.18	0.10	0.30	728.1	559.6	930.1
Belarus	M	90	0.046	0.025	0.083	0.71	0.50	0.97	0.16	0.09	0.27	749.9	576.9	961.4

Country	Sex	Midpoint of age range	Mean juice intake (servings/day)	Juice, lower uncertainty interval	Juice, upper uncertainty interval	Mean milk intake (servings/day)	Milk, lower uncertainty interval	Milk, upper uncertainty interval	Mean SSB intake (servings/day)	SSB, lower uncertainty interval	SSB, upper uncertainty interval	Mean calcium intake (servings/day)	Calcium, lower uncertainty interval	Calcium, upper uncertainty interval
Belize	F	25	0.531	0.259	0.972	0.69	0.38	1.13	3.55	1.89	5.88	817.1	621.8	1023.9
Belize	F	35	0.444	0.217	0.807	0.65	0.35	1.04	2.64	1.44	4.33	821.7	625.8	1029.4
Belize	F	45	0.364	0.175	0.655	0.61	0.34	1.00	1.79	0.96	3.03	828.1	628.4	1040.2
Belize	F	55	0.347	0.170	0.612	0.67	0.37	1.09	1.44	0.80	2.38	848.3	645.3	1063.2
Belize	F	65	0.342	0.166	0.606	0.75	0.42	1.24	1.20	0.65	1.99	871.7	667.6	1093.2
Belize	F	75	0.335	0.165	0.584	0.81	0.45	1.32	1.09	0.60	1.79	894.8	684.1	1123.7
Belize	F	90	0.326	0.161	0.565	0.88	0.49	1.43	1.00	0.54	1.63	922.4	704.4	1162.5
Belize	M	25	0.430	0.218	0.767	0.66	0.37	1.11	3.96	2.22	7.05	747.7	576.5	934.4
Belize	M	35	0.358	0.185	0.629	0.62	0.35	1.03	2.93	1.66	5.14	751.9	579.3	941.8
Belize	M	45	0.293	0.150	0.519	0.58	0.33	1.01	1.98	1.10	3.52	757.7	584.8	951.0
Belize	M	55	0.281	0.144	0.496	0.64	0.36	1.08	1.58	0.89	2.80	776.0	600.8	975.9
Belize	M	65	0.281	0.142	0.487	0.71	0.39	1.20	1.32	0.74	2.28	797.2	616.8	1005.2
Belize	M	75	0.273	0.139	0.472	0.77	0.43	1.29	1.20	0.67	2.06	818.6	635.6	1027.8
Belize	M	90	0.263	0.136	0.461	0.83	0.47	1.40	1.10	0.61	1.89	844.1	654.6	1060.5
Bolivia	F	25	0.586	0.289	1.020	0.32	0.18	0.54	2.00	1.10	3.44	577.8	437.7	752.0
Bolivia	F	35	0.486	0.244	0.826	0.30	0.17	0.50	1.49	0.82	2.55	581.6	444.2	752.7
Bolivia	F	45	0.397	0.194	0.698	0.28	0.15	0.48	1.02	0.54	1.75	586.7	445.6	760.1
Bolivia	F	55	0.381	0.192	0.657	0.31	0.17	0.52	0.81	0.43	1.41	601.8	459.7	776.5
Bolivia	F	65	0.380	0.191	0.669	0.35	0.20	0.58	0.67	0.36	1.16	618.9	470.3	804.5
Bolivia	F	75	0.373	0.188	0.653	0.38	0.21	0.62	0.60	0.33	1.03	634.5	485.7	820.7
Bolivia	F	90	0.362	0.182	0.626	0.41	0.23	0.65	0.56	0.31	0.93	652.2	495.8	839.3
Bolivia	M	25	0.483	0.225	0.879	0.29	0.16	0.49	2.11	1.19	3.49	528.5	401.5	681.1
Bolivia	M	35	0.400	0.192	0.720	0.28	0.15	0.47	1.57	0.91	2.61	531.6	407.9	676.7
Bolivia	M	45	0.325	0.150	0.606	0.26	0.14	0.45	1.07	0.59	1.88	535.8	408.6	683.0
Bolivia	M	55	0.314	0.152	0.566	0.29	0.16	0.49	0.85	0.48	1.49	549.0	421.4	694.4
Bolivia	M	65	0.316	0.155	0.570	0.32	0.17	0.54	0.70	0.40	1.22	564.0	429.7	717.0
Bolivia	M	75	0.308	0.149	0.552	0.35	0.19	0.58	0.64	0.37	1.11	578.6	442.2	731.0
Bolivia	M	90	0.297	0.145	0.526	0.38	0.20	0.63	0.59	0.34	1.03	595.1	456.8	747.9
Brazil	F	25	0.423	0.350	0.500	0.46	0.40	0.54	0.79	0.66	0.93	552.4	516.4	591.3
Brazil	F	35	0.352	0.309	0.397	0.43	0.39	0.48	0.60	0.53	0.68	555.6	530.0	582.1
Brazil	F	45	0.288	0.239	0.342	0.41	0.35	0.48	0.42	0.35	0.49	560.0	523.7	596.7
Brazil	F	55	0.277	0.241	0.315	0.45	0.41	0.50	0.34	0.29	0.38	574.4	546.9	603.3
Brazil	F	65	0.276	0.232	0.323	0.51	0.44	0.57	0.28	0.24	0.33	591.0	556.9	625.3
Brazil	F	75	0.270	0.234	0.306	0.55	0.49	0.60	0.25	0.22	0.29	606.6	578.3	634.1
Brazil	F	90	0.260	0.230	0.293	0.59	0.54	0.66	0.23	0.20	0.26	625.3	596.3	654.6
Brazil	M	25	0.351	0.289	0.420	0.43	0.37	0.49	0.88	0.74	1.03	504.1	472.1	536.2
Brazil	M	35	0.291	0.254	0.333	0.40	0.36	0.44	0.66	0.58	0.75	507.1	484.9	530.8
Brazil	M	45	0.237	0.195	0.282	0.38	0.32	0.43	0.46	0.39	0.54	511.3	479.5	542.9
Brazil	M	55	0.228	0.198	0.260	0.41	0.37	0.46	0.37	0.32	0.42	524.0	501.0	547.4
Brazil	M	65	0.228	0.193	0.268	0.46	0.40	0.53	0.30	0.26	0.35	538.5	507.1	571.0
Brazil	M	75	0.223	0.195	0.255	0.50	0.45	0.55	0.27	0.24	0.31	552.7	527.5	579.2
Brazil	M	90	0.215	0.189	0.245	0.54	0.49	0.60	0.25	0.22	0.28	569.8	544.5	596.3
Barbados	F	25	0.180	0.133	0.244	0.48	0.39	0.58	4.39	3.45	5.46	671.1	611.1	729.6
Barbados	F	35	0.151	0.112	0.200	0.45	0.38	0.54	3.26	2.67	3.95	674.9	623.5	728.4
Barbados	F	45	0.124	0.090	0.171	0.43	0.35	0.52	2.21	1.76	2.74	680.2	623.2	742.1
Barbados	F	55	0.118	0.088	0.157	0.47	0.39	0.56	1.78	1.46	2.17	696.7	644.3	752.5
Barbados	F	65	0.116	0.086	0.157	0.52	0.43	0.63	1.48	1.20	1.80	715.9	654.6	781.8
Barbados	F	75	0.114	0.086	0.151	0.57	0.47	0.67	1.35	1.12	1.62	734.9	677.3	794.2
Barbados	F	90	0.111	0.083	0.145	0.62	0.52	0.74	1.24	1.03	1.48	758.3	697.1	821.2
Barbados	M	25	0.148	0.109	0.203	0.45	0.36	0.54	4.79	3.77	5.97	613.5	560.9	670.3
Barbados	M	35	0.123	0.093	0.166	0.42	0.35	0.49	3.55	2.90	4.29	616.9	573.7	666.9
Barbados	M	45	0.101	0.072	0.138	0.40	0.32	0.48	2.39	1.90	2.93	621.8	574.4	674.6
Barbados	M	55	0.097	0.072	0.131	0.43	0.36	0.51	1.91	1.55	2.30	636.8	590.0	687.1
Barbados	M	65	0.097	0.071	0.131	0.48	0.40	0.58	1.60	1.28	1.95	654.0	603.0	710.5
Barbados	M	75	0.094	0.070	0.126	0.52	0.44	0.62	1.46	1.20	1.75	671.6	623.3	724.2
Barbados	M	90	0.091	0.068	0.120	0.56	0.48	0.66	1.34	1.12	1.61	692.5	641.1	746.7
Brunei Darussalam	F	25	0.435	0.214	0.770	0.66	0.37	1.10	0.56	0.29	0.98	634.8	578.0	694.2
Brunei Darussalam	F	35	0.363	0.178	0.641	0.63	0.35	1.04	0.42	0.22	0.73	640.2	587.9	693.2
Brunei Darussalam	F	45	0.297	0.144	0.535	0.61	0.33	1.03	0.29	0.15	0.49	646.8	589.9	703.5
Brunei Darussalam	F	55	0.286	0.139	0.509	0.67	0.37	1.12	0.22	0.11	0.39	662.4	611.5	715.9
Brunei Darussalam	F	65	0.287	0.138	0.510	0.75	0.40	1.26	0.18	0.09	0.31	679.9	622.7	734.8
Brunei Darussalam	F	75	0.280	0.137	0.499	0.80	0.43	1.32	0.17	0.09	0.30	697.3	643.3	751.1
Brunei Darussalam	F	90	0.270	0.132	0.482	0.84	0.46	1.36	0.16	0.08	0.29	717.3	659.8	777.4
Brunei Darussalam	M	25	0.354	0.177	0.626	0.61	0.34	1.02	0.65	0.35	1.12	579.9	529.0	633.7
Brunei Darussalam	M	35	0.295	0.153	0.514	0.57	0.32	0.94	0.50	0.27	0.83	584.7	537.7	634.8
Brunei Darussalam	M	45	0.242	0.123	0.420	0.54	0.30	0.89	0.35	0.19	0.59	590.5	540.0	643.7
Brunei Darussalam	M	55	0.232	0.118	0.403	0.59	0.33	0.97	0.27	0.15	0.46	604.6	558.7	656.0
Brunei Darussalam	M	65	0.231	0.116	0.404	0.67	0.37	1.09	0.22	0.12	0.37	620.6	565.6	679.4
Brunei Darussalam	M	75	0.227	0.114	0.392	0.73	0.40	1.20	0.19	0.11	0.34	638.1	585.8	694.6
Brunei Darussalam	M	90	0.220	0.112	0.384	0.80	0.45	1.31	0.18	0.10	0.30	659.0	608.1	715.8
Bhutan	F	25	0.012	0.006	0.022	0.24	0.14	0.40	0.43	0.22	0.78	432.7	342.3	543.9
Bhutan	F	35	0.010	0.005	0.017	0.24	0.13	0.39	0.32	0.17	0.56	435.8	342.3	542.4
Bhutan	F	45	0.008	0.004	0.014	0.23	0.13	0.38	0.22	0.12	0.39	439.6	344.3	551.4
Bhutan	F	55	0.008	0.004	0.014	0.25	0.14	0.41	0.17	0.09	0.30	450.1	354.7	560.5
Bhutan	F	65	0.008	0.004	0.014	0.28	0.16	0.46	0.14	0.08	0.25	462.1	363.9	576.8
Bhutan	F	75	0.008	0.004	0.014	0.30	0.17	0.49	0.13	0.07	0.23	474.6	375.1	595.0
Bhutan	F	90	0.007	0.004	0.013	0.33	0.19	0.54	0.12	0.06	0.21	489.0	383.4	612.3
Bhutan	M	25	0.010	0.005	0.017	0.23	0.13	0.38	0.45	0.24	0.78	393.8	304.7	502.4
Bhutan	M	35	0.008	0.004	0.014	0.22	0.12	0.36	0.34	0.18	0.58	396.3	309.7	506.0
Bhutan	M	45	0.007	0.003	0.012	0.21	0.12	0.36	0.23	0.12	0.40	399.8	313.7	511.5
Bhutan	M	55	0.006	0.003	0.011	0.23	0.13	0.39	0.19	0.10	0.31	409.8	319.8	522.6
Bhutan	M	65	0.006	0.003	0.011	0.26	0.15	0.44	0.15	0.08	0.26	421.3	327.4	537.9
Bhutan	M	75	0.006	0.003	0.011	0.28	0.16	0.47	0.14	0.07	0.23	432.6	337.6	555.1
Bhutan	M	90	0.006	0.003	0.011	0.30	0.17	0.51	0.13	0.07	0.21	445.3	347.7	569.8
Botswana	F	25	0.062	0.031	0.113	1.14	0.62	1.97	1.43	0.77	2.42	518.9	405.1	668.2
Botswana	F	35	0.051	0.026	0.090	1.07	0.58	1.87	1.07	0.60	1.79	522.6	407.5	669.6
Botswana	F	45	0.041	0.021	0.073	1.02	0.54	1.81	0.73	0.41	1.22	527.3	409.1	674.1
Botswana	F	55	0.040	0.020	0.070	1.12	0.60	1.97	0.58	0.33	0.95	539.4	419.3	690.4
Botswana	F	65	0.040	0.019	0.071	1.24	0.65	2.19	0.47	0.26	0.79	553.2	429.7	707.7
Botswana	F	75	0.039	0.019	0.070	1.35	0.72	2.35	0.43	0.24	0.71	567.8	440.6	726.4
Botswana	F	90	0.038	0.019	0.067	1.46	0.78	2.54	0.40	0.22	0.66	584.7	455.0	748.9
Botswana	M	25	0.051	0.025	0.090	1.00	0.56	1.68	1.58	0.84	2.71	474.7	364.9	604.6
Botswana	M	35	0.042	0.022	0.074	0.94	0.53	1.56	1.18	0.64	2.04	477.7	370.7	609.1
Botswana	M	45	0.034	0.017	0.063	0.89	0.50	1.49	0.81	0.42	1.40	481.7	374.8	617.0
Botswana	M	55	0.033	0.017	0.058	0.98	0.55	1.60	0.64	0.34	1.11	493.1	387.6	626.9
Botswana	M	65	0.033	0.017	0.060	1.09	0.61	1.81	0.52	0.28	0.92	506.1	393.6	641.1
Botswana	M	75	0.032	0.017	0.058	1.18	0.67	1.93	0.48	0.26	0.83	519.6	405.9	658.6
Botswana	M	90	0.031	0.016	0.054	1.28	0.73	2.10	0.44	0.24	0.75	535.0	416.1	681.3
Central African Republic	F	25	0.055	0.027	0.099	0.65	0.34	1.11	0.52	0.28	0.87	508.1	381.2	676.8

Country	Sex	Midpoint of age range	Mean juice intake (servings/day)	Juice, lower uncertainty interval	Juice, upper uncertainty interval	Mean milk intake (servings/day)	Milk, lower uncertainty interval	Milk, upper uncertainty interval	Mean SSB intake (servings/day)	SSB, lower uncertainty interval	SSB, upper uncertainty interval	Mean calcium intake (servings/day)	Calcium, lower uncertainty interval	Calcium, upper uncertainty interval
Central African Republic	F	35	0.045	0.023	0.082	0.61	0.32	1.03	0.39	0.21	0.65	511.4	383.7	676.3
Central African Republic	F	45	0.037	0.018	0.068	0.58	0.30	1.01	0.27	0.14	0.45	516.0	386.0	690.4
Central African Republic	F	55	0.035	0.018	0.062	0.64	0.33	1.06	0.21	0.11	0.36	529.3	398.0	699.1
Central African Republic	F	65	0.035	0.018	0.062	0.71	0.37	1.20	0.17	0.09	0.28	544.4	407.1	719.1
Central African Republic	F	75	0.034	0.018	0.060	0.77	0.40	1.28	0.16	0.09	0.26	558.5	420.1	730.2
Central African Republic	F	90	0.033	0.017	0.057	0.83	0.44	1.36	0.14	0.08	0.24	574.4	432.3	752.5
Central African Republic	M	25	0.044	0.021	0.082	0.60	0.30	1.07	0.58	0.31	0.99	468.6	350.7	610.6
Central African Republic	M	35	0.036	0.018	0.065	0.56	0.28	0.95	0.43	0.24	0.74	472.1	357.6	609.9
Central African Republic	M	45	0.030	0.015	0.053	0.53	0.26	0.90	0.30	0.16	0.51	476.6	357.1	622.0
Central African Republic	M	55	0.029	0.014	0.052	0.58	0.29	0.98	0.23	0.13	0.40	488.6	369.9	633.9
Central African Republic	M	65	0.029	0.014	0.053	0.65	0.33	1.13	0.19	0.10	0.32	502.1	378.8	651.0
Central African Republic	M	75	0.028	0.014	0.051	0.70	0.36	1.22	0.17	0.09	0.29	514.9	389.6	667.7
Central African Republic	M	90	0.027	0.014	0.049	0.76	0.39	1.28	0.16	0.09	0.28	529.2	396.1	687.6
Canada	F	25	0.689	0.577	0.824	0.66	0.57	0.77	1.13	0.96	1.32	852.1	792.5	910.3
Canada	F	35	0.569	0.492	0.658	0.61	0.55	0.68	0.85	0.74	0.97	856.4	810.6	904.5
Canada	F	45	0.462	0.385	0.550	0.58	0.50	0.66	0.59	0.50	0.69	862.5	808.9	920.6
Canada	F	55	0.456	0.391	0.524	0.63	0.56	0.70	0.46	0.40	0.53	883.1	836.7	929.9
Canada	F	65	0.468	0.399	0.551	0.69	0.60	0.80	0.37	0.32	0.43	906.8	855.3	962.5
Canada	F	75	0.467	0.407	0.538	0.75	0.67	0.85	0.34	0.29	0.39	930.4	884.7	981.9
Canada	F	90	0.462	0.400	0.532	0.83	0.74	0.94	0.31	0.27	0.35	960.0	906.0	1014.1
Canada	M	25	0.585	0.492	0.690	0.62	0.54	0.72	1.29	1.09	1.51	780.9	728.5	833.7
Canada	M	35	0.488	0.426	0.560	0.58	0.52	0.65	0.98	0.86	1.12	784.4	740.4	829.3
Canada	M	45	0.402	0.334	0.473	0.54	0.47	0.62	0.69	0.59	0.81	789.7	739.0	843.5
Canada	M	55	0.394	0.343	0.450	0.59	0.52	0.66	0.54	0.47	0.63	808.4	764.4	854.9
Canada	M	65	0.400	0.340	0.468	0.65	0.57	0.74	0.43	0.37	0.50	830.1	779.8	889.2
Canada	M	75	0.395	0.343	0.456	0.71	0.63	0.79	0.38	0.33	0.44	852.6	806.3	904.9
Canada	M	90	0.384	0.330	0.442	0.78	0.69	0.88	0.34	0.30	0.39	879.9	832.6	927.9
Switzerland	F	25	0.846	0.645	1.103	0.88	0.49	1.52	0.48	0.33	0.66	912.4	705.9	1177.6
Switzerland	F	35	0.679	0.523	0.886	0.83	0.46	1.42	0.34	0.24	0.47	919.0	714.2	1175.1
Switzerland	F	45	0.530	0.402	0.695	0.79	0.43	1.35	0.21	0.15	0.30	927.2	722.2	1182.1
Switzerland	F	55	0.514	0.391	0.661	0.84	0.46	1.45	0.17	0.12	0.24	947.6	737.7	1208.4
Switzerland	F	65	0.524	0.394	0.676	0.91	0.51	1.61	0.15	0.10	0.20	970.9	752.2	1245.6
Switzerland	F	75	0.524	0.396	0.679	1.00	0.56	1.76	0.14	0.10	0.19	995.5	773.2	1275.6
Switzerland	F	90	0.520	0.395	0.688	1.12	0.62	1.94	0.13	0.09	0.17	1027.2	794.9	1316.9
Switzerland	M	25	0.671	0.504	0.871	0.82	0.46	1.40	0.56	0.39	0.79	843.8	647.3	1081.9
Switzerland	M	35	0.544	0.418	0.700	0.75	0.41	1.26	0.40	0.27	0.55	847.3	654.3	1079.3
Switzerland	M	45	0.429	0.327	0.561	0.69	0.38	1.16	0.24	0.17	0.35	852.4	651.8	1085.1
Switzerland	M	55	0.407	0.313	0.525	0.73	0.40	1.23	0.19	0.13	0.26	870.7	663.9	1108.3
Switzerland	M	65	0.405	0.305	0.530	0.80	0.44	1.37	0.16	0.11	0.22	892.1	682.3	1138.6
Switzerland	M	75	0.409	0.315	0.534	0.89	0.49	1.51	0.14	0.10	0.20	914.4	699.6	1157.9
Switzerland	M	90	0.416	0.319	0.553	1.00	0.55	1.67	0.13	0.09	0.19	941.9	724.0	1190.9
Chile	F	25	0.363	0.185	0.640	0.35	0.19	0.60	1.13	0.60	1.88	495.0	380.5	629.4
Chile	F	35	0.301	0.157	0.519	0.33	0.18	0.56	0.85	0.47	1.39	497.9	386.5	629.5
Chile	F	45	0.244	0.124	0.420	0.32	0.17	0.54	0.59	0.32	0.99	501.9	385.8	635.8
Chile	F	55	0.235	0.122	0.412	0.35	0.19	0.59	0.47	0.26	0.79	514.4	396.9	657.2
Chile	F	65	0.234	0.122	0.411	0.39	0.21	0.66	0.38	0.21	0.64	528.7	405.7	688.2
Chile	F	75	0.230	0.121	0.400	0.42	0.23	0.71	0.34	0.19	0.57	542.6	418.4	696.4
Chile	F	90	0.223	0.116	0.393	0.46	0.25	0.78	0.31	0.17	0.52	559.6	434.2	704.6
Chile	M	25	0.290	0.153	0.518	0.33	0.17	0.57	1.25	0.69	2.11	459.9	354.1	593.0
Chile	M	35	0.241	0.127	0.429	0.31	0.17	0.53	0.93	0.52	1.54	462.5	356.6	593.4
Chile	M	45	0.197	0.100	0.347	0.30	0.16	0.51	0.64	0.35	1.06	466.1	357.5	600.9
Chile	M	55	0.189	0.095	0.336	0.33	0.18	0.56	0.51	0.28	0.84	477.6	370.6	612.3
Chile	M	65	0.189	0.097	0.342	0.36	0.20	0.64	0.42	0.23	0.70	490.9	379.0	635.7
Chile	M	75	0.185	0.096	0.332	0.39	0.21	0.68	0.38	0.21	0.63	504.0	388.4	649.8
Chile	M	90	0.180	0.092	0.323	0.43	0.23	0.74	0.35	0.19	0.57	519.8	397.5	671.7
China	F	25	0.001	0.001	0.002	0.06	0.05	0.08	0.09	0.08	0.11	321.2	298.8	344.7
China	F	35	0.001	0.001	0.001	0.06	0.05	0.07	0.07	0.06	0.08	323.6	305.2	343.0
China	F	45	0.001	0.001	0.001	0.06	0.05	0.06	0.05	0.04	0.06	326.6	304.6	350.0
China	F	55	0.001	0.001	0.001	0.06	0.05	0.07	0.04	0.03	0.04	335.1	315.7	355.3
China	F	65	0.001	0.001	0.001	0.07	0.06	0.08	0.03	0.03	0.04	344.5	322.3	367.4
China	F	75	0.001	0.001	0.001	0.07	0.07	0.09	0.03	0.02	0.03	353.5	332.7	373.9
China	F	90	0.001	0.001	0.001	0.08	0.07	0.09	0.03	0.02	0.03	363.8	342.7	385.1
China	M	25	0.001	0.001	0.001	0.06	0.05	0.07	0.10	0.08	0.13	293.4	273.1	314.7
China	M	35	0.001	0.001	0.001	0.05	0.05	0.06	0.08	0.06	0.09	295.6	279.1	312.0
China	M	45	0.001	0.001	0.001	0.05	0.04	0.06	0.05	0.04	0.06	298.3	279.0	317.9
China	M	55	0.001	0.001	0.001	0.06	0.05	0.06	0.04	0.03	0.05	306.0	289.7	322.7
China	M	65	0.001	0.001	0.001	0.06	0.05	0.08	0.03	0.03	0.04	314.8	294.5	334.6
China	M	75	0.001	0.001	0.001	0.07	0.06	0.08	0.03	0.03	0.04	323.4	305.6	340.6
China	M	90	0.001	0.001	0.001	0.08	0.07	0.09	0.03	0.02	0.03	333.0	315.6	351.6
Côte d'Ivoire	F	25	0.055	0.025	0.101	0.15	0.08	0.24	0.55	0.30	0.94	396.8	307.7	500.6
Côte d'Ivoire	F	35	0.046	0.021	0.083	0.14	0.08	0.23	0.41	0.23	0.70	399.1	313.0	502.9
Côte d'Ivoire	F	45	0.037	0.017	0.068	0.13	0.07	0.22	0.28	0.15	0.49	402.2	313.9	508.4
Côte d'Ivoire	F	55	0.036	0.017	0.065	0.15	0.08	0.24	0.22	0.12	0.38	412.5	321.9	521.2
Côte d'Ivoire	F	65	0.035	0.017	0.066	0.16	0.09	0.27	0.18	0.10	0.31	424.2	328.2	539.7
Côte d'Ivoire	F	75	0.035	0.017	0.064	0.18	0.10	0.29	0.17	0.09	0.27	435.1	339.6	549.7
Côte d'Ivoire	F	90	0.034	0.016	0.062	0.19	0.10	0.32	0.15	0.09	0.25	447.3	350.0	563.0
Côte d'Ivoire	M	25	0.045	0.021	0.085	0.13	0.07	0.23	0.62	0.33	1.04	362.8	282.9	453.9
Côte d'Ivoire	M	35	0.037	0.018	0.070	0.13	0.07	0.21	0.46	0.25	0.77	365.2	282.4	457.9
Côte d'Ivoire	M	45	0.030	0.014	0.056	0.12	0.06	0.21	0.31	0.17	0.53	368.4	283.3	466.3
Côte d'Ivoire	M	55	0.029	0.014	0.054	0.13	0.07	0.22	0.25	0.13	0.41	377.3	292.3	475.3
Côte d'Ivoire	M	65	0.029	0.014	0.055	0.15	0.08	0.25	0.20	0.11	0.35	387.5	301.4	488.7
Côte d'Ivoire	M	75	0.029	0.014	0.053	0.16	0.09	0.27	0.19	0.10	0.31	397.5	311.7	500.2
Côte d'Ivoire	M	90	0.028	0.014	0.051	0.17	0.09	0.29	0.17	0.09	0.29	408.9	318.2	519.3
Cameroon	F	25	0.133	0.060	0.246	0.26	0.13	0.44	0.79	0.42	1.31	454.3	351.3	582.8
Cameroon	F	35	0.111	0.052	0.207	0.24	0.13	0.41	0.59	0.31	0.98	456.9	355.7	581.7
Cameroon	F	45	0.090	0.043	0.171	0.23	0.13	0.39	0.40	0.21	0.68	460.5	355.8	585.8
Cameroon	F	55	0.086	0.042	0.160	0.25	0.14	0.43	0.32	0.17	0.54	472.2	368.3	598.1
Cameroon	F	65	0.086	0.041	0.156	0.29	0.16	0.49	0.26	0.14	0.44	485.5	377.1	616.8
Cameroon	F	75	0.084	0.040	0.154	0.31	0.17	0.52	0.24	0.13	0.40	498.0	386.9	636.3
Cameroon	F	90	0.082	0.040	0.150	0.33	0.18	0.56	0.22	0.12	0.37	512.2	399.6	656.1
Cameroon	M	25	0.110	0.051	0.205	0.24	0.13	0.41	0.89	0.46	1.50	415.6	320.6	532.7
Cameroon	M	35	0.092	0.043	0.170	0.23	0.13	0.38	0.66	0.35	1.11	418.3	325.4	533.4
Cameroon	M	45	0.075	0.034	0.140	0.22	0.12	0.36	0.45	0.24	0.76	421.8	328.6	540.0
Cameroon	M	55	0.072	0.034	0.133	0.24	0.13	0.39	0.36	0.19	0.61	432.1	338.1	553.6
Cameroon	M	65	0.072	0.034	0.136	0.27	0.14	0.44	0.29	0.15	0.51	443.7	347.7	570.4
Cameroon	M	75	0.071	0.033	0.131	0.29	0.15	0.47	0.27	0.14	0.46	455.3	356.7	587.1
Cameroon	M	90	0.069	0.032	0.126	0.31	0.17	0.51	0.25	0.13	0.42	468.5	366.7	604.9
Democratic Republic of the Congo	F	25	0.029	0.013	0.052	0.12	0.07	0.21	0.40	0.21	0.66	347.2	263.5	443.4

Country	Sex	Midpoint of age range	Mean juice intake (servings/day)	Juice, lower uncertainty interval	Juice, upper uncertainty interval	Mean milk intake (servings/day)	Milk, lower uncertainty interval	Milk, upper uncertainty interval	Mean SSB intake (servings/day)	SSB, lower uncertainty interval	SSB, upper uncertainty interval	Mean calcium intake (servings/day)	Calcium, lower uncertainty interval	Calcium, upper uncertainty interval
Democratic Republic of the Congo	F	35	0.024	0.012	0.042	0.12	0.07	0.20	0.30	0.16	0.48	349.6	264.0	445.4
Democratic Republic of the Congo	F	45	0.019	0.010	0.035	0.11	0.06	0.19	0.21	0.11	0.34	352.7	266.7	452.8
Democratic Republic of the Congo	F	55	0.019	0.009	0.033	0.12	0.07	0.20	0.16	0.09	0.26	361.9	276.4	459.3
Democratic Republic of the Congo	F	65	0.018	0.009	0.033	0.13	0.07	0.23	0.13	0.07	0.21	372.3	282.7	480.6
Democratic Republic of the Congo	F	75	0.018	0.009	0.032	0.14	0.08	0.24	0.12	0.07	0.19	381.9	290.7	489.7
Democratic Republic of the Congo	F	90	0.017	0.009	0.030	0.16	0.09	0.26	0.11	0.06	0.18	392.5	299.9	500.4
Democratic Republic of the Congo	M	25	0.023	0.011	0.043	0.11	0.06	0.19	0.45	0.24	0.77	317.3	244.7	401.6
Democratic Republic of the Congo	M	35	0.019	0.009	0.035	0.10	0.06	0.18	0.33	0.18	0.57	319.6	248.4	403.7
Democratic Republic of the Congo	M	45	0.016	0.007	0.029	0.10	0.05	0.17	0.23	0.12	0.39	322.6	250.0	409.9
Democratic Republic of the Congo	M	55	0.015	0.007	0.027	0.11	0.06	0.18	0.18	0.10	0.31	330.7	257.0	418.5
Democratic Republic of the Congo	M	65	0.015	0.007	0.028	0.12	0.07	0.21	0.15	0.08	0.25	340.0	258.9	434.2
Democratic Republic of the Congo	M	75	0.015	0.007	0.027	0.13	0.07	0.22	0.13	0.07	0.23	348.6	267.3	441.8
Democratic Republic of the Congo	M	90	0.014	0.007	0.026	0.14	0.08	0.24	0.12	0.07	0.21	358.1	277.1	447.8
Congo	F	25	0.265	0.130	0.491	0.54	0.28	0.94	1.14	0.58	1.99	491.0	371.0	647.9
Congo	F	35	0.221	0.110	0.396	0.51	0.27	0.84	0.86	0.44	1.46	494.3	378.2	648.2
Congo	F	45	0.180	0.085	0.329	0.48	0.25	0.81	0.59	0.30	1.04	498.8	376.9	654.4
Congo	F	55	0.173	0.084	0.308	0.53	0.28	0.88	0.47	0.24	0.80	511.6	388.8	660.9
Congo	F	65	0.172	0.084	0.310	0.59	0.31	1.01	0.38	0.20	0.65	526.2	400.6	681.3
Congo	F	75	0.168	0.084	0.299	0.64	0.33	1.07	0.35	0.18	0.60	539.8	413.2	700.3
Congo	F	90	0.162	0.082	0.289	0.69	0.36	1.13	0.32	0.17	0.55	555.3	424.7	720.5
Congo	M	25	0.214	0.105	0.401	0.49	0.26	0.86	1.29	0.68	2.25	448.1	336.5	586.9
Congo	M	35	0.177	0.090	0.322	0.46	0.25	0.77	0.96	0.51	1.67	451.1	344.1	585.9
Congo	M	45	0.145	0.071	0.263	0.44	0.23	0.76	0.65	0.34	1.16	455.2	345.3	595.4
Congo	M	55	0.139	0.069	0.250	0.48	0.26	0.81	0.52	0.28	0.90	466.7	355.3	605.4
Congo	M	65	0.139	0.067	0.259	0.54	0.29	0.93	0.42	0.22	0.75	479.9	359.9	626.9
Congo	M	75	0.136	0.066	0.250	0.58	0.32	1.02	0.38	0.20	0.67	492.1	373.0	638.6
Congo	M	90	0.132	0.066	0.235	0.63	0.34	1.10	0.36	0.19	0.62	505.8	381.1	659.5
Colombia	F	25	1.219	0.614	2.222	0.95	0.80	1.14	3.26	2.27	4.45	540.8	500.6	583.4
Colombia	F	35	1.009	0.514	1.827	0.90	0.77	1.05	2.54	1.84	3.40	544.8	508.8	579.4
Colombia	F	45	0.819	0.412	1.487	0.86	0.72	1.04	1.85	1.32	2.54	549.8	506.6	589.7
Colombia	F	55	0.787	0.401	1.422	0.94	0.80	1.08	1.48	1.07	1.98	563.5	525.5	598.8
Colombia	F	65	0.788	0.394	1.456	1.04	0.88	1.22	1.20	0.86	1.61	579.0	537.1	623.4
Colombia	F	75	0.771	0.388	1.416	1.13	0.97	1.30	1.07	0.77	1.44	593.5	554.4	634.4
Colombia	F	90	0.747	0.379	1.355	1.23	1.06	1.41	0.97	0.70	1.30	610.8	570.6	651.7
Colombia	M	25	1.011	0.490	1.892	0.87	0.72	1.01	3.55	2.55	4.76	495.0	457.0	535.5
Colombia	M	35	0.835	0.418	1.523	0.82	0.70	0.94	2.76	2.02	3.64	497.8	463.5	532.0
Colombia	M	45	0.677	0.321	1.253	0.78	0.66	0.92	2.02	1.44	2.73	501.8	461.9	541.6
Colombia	M	55	0.651	0.316	1.211	0.86	0.74	0.99	1.62	1.18	2.17	514.7	479.5	548.1
Colombia	M	65	0.653	0.316	1.220	0.95	0.81	1.12	1.32	0.93	1.79	529.5	490.6	569.7
Colombia	M	75	0.641	0.312	1.202	1.03	0.89	1.19	1.18	0.84	1.58	543.5	506.7	579.9
Colombia	M	90	0.624	0.300	1.195	1.12	0.96	1.32	1.07	0.79	1.45	559.7	523.4	597.0
Comoros	F	25	0.181	0.083	0.356	0.16	0.09	0.27	0.75	0.40	1.26	375.8	287.9	484.8
Comoros	F	35	0.150	0.069	0.291	0.15	0.08	0.26	0.56	0.31	0.93	378.0	290.5	483.1
Comoros	F	45	0.122	0.057	0.237	0.14	0.08	0.24	0.38	0.21	0.64	381.0	291.9	487.3
Comoros	F	55	0.117	0.055	0.226	0.16	0.08	0.26	0.30	0.17	0.49	390.7	302.0	501.7
Comoros	F	65	0.117	0.054	0.231	0.17	0.09	0.29	0.25	0.14	0.41	401.8	310.8	518.0
Comoros	F	75	0.115	0.053	0.228	0.19	0.10	0.32	0.22	0.13	0.37	412.2	317.4	529.8
Comoros	F	90	0.111	0.051	0.215	0.20	0.11	0.35	0.21	0.12	0.34	424.0	321.1	540.9
Comoros	M	25	0.146	0.067	0.275	0.15	0.08	0.23	0.82	0.45	1.37	341.8	264.6	438.0
Comoros	M	35	0.121	0.057	0.232	0.14	0.08	0.22	0.62	0.34	0.99	344.1	265.4	440.5
Comoros	M	45	0.098	0.045	0.190	0.13	0.07	0.21	0.42	0.23	0.69	347.1	267.2	445.4
Comoros	M	55	0.094	0.044	0.180	0.14	0.08	0.23	0.33	0.18	0.55	355.6	276.3	457.8
Comoros	M	65	0.094	0.044	0.179	0.16	0.09	0.26	0.27	0.15	0.45	365.3	285.2	471.1
Comoros	M	75	0.092	0.043	0.174	0.17	0.10	0.28	0.25	0.14	0.41	374.8	292.6	480.1
Comoros	M	90	0.089	0.042	0.163	0.18	0.11	0.30	0.23	0.12	0.38	385.4	299.5	492.3
Cape Verde	F	25	0.069	0.033	0.126	0.93	0.46	1.59	1.38	0.72	2.44	601.3	453.4	790.3
Cape Verde	F	35	0.057	0.028	0.104	0.87	0.43	1.48	1.03	0.55	1.78	604.7	456.4	788.2
Cape Verde	F	45	0.046	0.023	0.084	0.83	0.42	1.40	0.70	0.37	1.21	609.6	456.1	798.5
Cape Verde	F	55	0.045	0.022	0.081	0.91	0.48	1.53	0.56	0.30	0.95	624.9	470.8	816.2
Cape Verde	F	65	0.044	0.022	0.084	1.02	0.53	1.71	0.46	0.25	0.81	642.8	483.1	842.0
Cape Verde	F	75	0.044	0.022	0.082	1.11	0.57	1.84	0.42	0.22	0.73	659.4	500.4	858.3
Cape Verde	F	90	0.042	0.021	0.078	1.20	0.62	1.97	0.38	0.21	0.67	678.1	515.0	887.4
Cape Verde	M	25	0.055	0.027	0.105	0.86	0.44	1.52	1.58	0.80	2.82	550.9	416.8	731.5
Cape Verde	M	35	0.046	0.022	0.083	0.80	0.42	1.41	1.18	0.62	2.06	554.4	418.2	728.4
Cape Verde	M	45	0.037	0.018	0.067	0.76	0.39	1.34	0.80	0.41	1.43	559.1	418.0	730.8
Cape Verde	M	55	0.036	0.018	0.065	0.84	0.43	1.44	0.64	0.33	1.14	572.5	432.4	747.0
Cape Verde	M	65	0.036	0.017	0.068	0.93	0.48	1.64	0.52	0.26	0.95	588.3	449.9	774.0
Cape Verde	M	75	0.035	0.017	0.065	1.01	0.53	1.76	0.47	0.24	0.86	603.8	463.0	796.6
Cape Verde	M	90	0.034	0.017	0.062	1.09	0.56	1.89	0.44	0.22	0.78	620.8	475.9	818.2
Costa Rica	F	25	0.714	0.355	1.262	1.47	0.82	2.50	3.31	1.80	5.63	775.0	590.5	978.1
Costa Rica	F	35	0.591	0.293	1.018	1.39	0.78	2.27	2.58	1.41	4.40	780.7	595.0	982.7
Costa Rica	F	45	0.479	0.232	0.837	1.33	0.72	2.17	1.89	1.03	3.24	788.1	598.0	1004.1
Costa Rica	F	55	0.461	0.228	0.799	1.45	0.80	2.32	1.51	0.82	2.54	807.7	617.2	1032.4
Costa Rica	F	65	0.462	0.232	0.797	1.61	0.90	2.61	1.21	0.66	2.03	830.0	637.4	1060.1
Costa Rica	F	75	0.452	0.231	0.777	1.74	0.98	2.85	1.09	0.60	1.80	850.8	656.3	1089.7
Costa Rica	F	90	0.436	0.229	0.751	1.90	1.06	3.16	0.98	0.54	1.61	875.9	673.2	1113.3
Costa Rica	M	25	0.611	0.303	1.060	1.31	0.74	2.21	3.59	1.94	5.84	713.7	551.8	911.2
Costa Rica	M	35	0.505	0.250	0.872	1.24	0.70	2.06	2.79	1.55	4.45	717.8	556.7	925.0
Costa Rica	M	45	0.409	0.199	0.713	1.18	0.65	1.96	2.04	1.11	3.36	723.5	558.0	926.8
Costa Rica	M	55	0.394	0.194	0.685	1.30	0.72	2.16	1.64	0.90	2.67	742.1	573.0	947.3
Costa Rica	M	65	0.395	0.197	0.700	1.45	0.79	2.45	1.33	0.74	2.20	763.4	586.6	965.9
Costa Rica	M	75	0.388	0.196	0.682	1.57	0.88	2.68	1.19	0.67	1.96	783.5	606.1	995.5
Costa Rica	M	90	0.376	0.190	0.650	1.70	0.95	2.90	1.08	0.61	1.75	807.5	631.2	1031.0
Cuba	F	25	0.444	0.216	0.780	0.56	0.30	0.92	4.15	2.33	6.88	778.3	606.4	1004.5
Cuba	F	35	0.370	0.183	0.646	0.52	0.29	0.86	3.08	1.76	5.14	782.7	607.4	1003.6
Cuba	F	45	0.304	0.153	0.522	0.50	0.27	0.83	2.10	1.17	3.56	788.7	605.1	1014.6

Country	Sex	Midpoint of age range	Mean juice intake (servings/day)	Juice, lower uncertainty interval	Juice, upper uncertainty interval	Mean milk intake (servings/day)	Milk, lower uncertainty interval	Milk, upper uncertainty interval	Mean SSB intake (servings/day)	SSB, lower uncertainty interval	SSB, upper uncertainty interval	Mean calcium intake (servings/day)	Calcium, lower uncertainty interval	Calcium, upper uncertainty interval
Cuba	F	55	0.290	0.146	0.496	0.55	0.30	0.91	1.68	0.94	2.85	808.0	627.4	1029.1
Cuba	F	65	0.286	0.142	0.503	0.61	0.33	1.02	1.40	0.78	2.38	830.2	644.7	1059.7
Cuba	F	75	0.280	0.140	0.491	0.66	0.36	1.10	1.28	0.70	2.14	852.1	662.9	1087.0
Cuba	F	90	0.272	0.140	0.479	0.72	0.40	1.18	1.17	0.63	1.96	879.0	681.9	1120.4
Cuba	M	25	0.371	0.183	0.658	0.52	0.30	0.87	4.45	2.39	7.76	716.3	556.7	914.7
Cuba	M	35	0.309	0.158	0.537	0.48	0.28	0.80	3.29	1.76	5.64	720.5	563.4	917.1
Cuba	M	45	0.253	0.130	0.439	0.46	0.26	0.77	2.22	1.19	3.91	726.1	567.3	929.7
Cuba	M	55	0.243	0.127	0.426	0.50	0.29	0.82	1.78	0.96	3.05	743.6	582.8	945.4
Cuba	M	65	0.243	0.123	0.439	0.56	0.32	0.90	1.48	0.78	2.50	763.8	599.1	980.7
Cuba	M	75	0.237	0.121	0.424	0.60	0.35	0.98	1.35	0.73	2.28	784.2	617.5	1005.9
Cuba	M	90	0.227	0.117	0.397	0.65	0.38	1.08	1.24	0.67	2.08	808.9	639.2	1044.4
Cyprus	F	25	0.433	0.263	0.663	0.77	0.54	1.06	0.80	0.51	1.22	906.2	706.6	1160.5
Cyprus	F	35	0.348	0.214	0.536	0.73	0.52	1.00	0.57	0.37	0.86	912.9	717.2	1170.9
Cyprus	F	45	0.271	0.165	0.418	0.69	0.49	0.95	0.36	0.23	0.54	921.2	720.6	1183.0
Cyprus	F	55	0.263	0.163	0.400	0.74	0.53	1.01	0.29	0.18	0.44	941.4	741.6	1208.3
Cyprus	F	65	0.268	0.165	0.411	0.80	0.56	1.11	0.25	0.16	0.38	964.3	753.5	1236.5
Cyprus	F	75	0.268	0.164	0.410	0.88	0.63	1.22	0.23	0.15	0.35	988.8	772.3	1268.6
Cyprus	F	90	0.266	0.161	0.408	0.98	0.70	1.37	0.21	0.13	0.32	1018.9	795.4	1300.2
Cyprus	M	25	0.352	0.220	0.529	0.74	0.52	1.04	0.93	0.60	1.37	838.3	639.4	1065.3
Cyprus	M	35	0.285	0.182	0.426	0.68	0.48	0.94	0.66	0.43	0.96	841.9	644.7	1073.5
Cyprus	M	45	0.225	0.142	0.338	0.62	0.44	0.86	0.40	0.26	0.59	847.3	648.7	1082.7
Cyprus	M	55	0.213	0.136	0.321	0.66	0.47	0.91	0.31	0.20	0.46	865.2	662.3	1107.0
Cyprus	M	65	0.212	0.131	0.317	0.72	0.51	1.01	0.26	0.17	0.39	886.2	677.3	1138.1
Cyprus	M	75	0.214	0.135	0.321	0.80	0.57	1.10	0.24	0.15	0.35	908.5	696.5	1164.3
Cyprus	M	90	0.218	0.139	0.323	0.90	0.65	1.22	0.22	0.14	0.32	935.8	713.4	1201.0
Czech Republic	F	25	0.135	0.083	0.207	0.38	0.25	0.56	0.47	0.30	0.70	597.0	471.3	755.3
Czech Republic	F	35	0.112	0.071	0.169	0.36	0.24	0.53	0.35	0.23	0.52	599.8	473.7	755.1
Czech Republic	F	45	0.091	0.057	0.139	0.35	0.23	0.51	0.24	0.15	0.36	604.0	474.5	763.9
Czech Republic	F	55	0.086	0.054	0.130	0.39	0.26	0.56	0.19	0.12	0.29	619.4	485.3	775.8
Czech Republic	F	65	0.084	0.052	0.126	0.44	0.28	0.63	0.16	0.10	0.24	637.0	500.0	800.5
Czech Republic	F	75	0.082	0.051	0.123	0.47	0.31	0.69	0.15	0.09	0.22	654.0	514.9	823.0
Czech Republic	F	90	0.081	0.049	0.120	0.52	0.34	0.76	0.14	0.09	0.20	674.6	535.9	844.7
Czech Republic	M	25	0.108	0.068	0.167	0.36	0.23	0.52	0.55	0.36	0.82	555.9	427.5	715.5
Czech Republic	M	35	0.089	0.058	0.134	0.33	0.22	0.49	0.41	0.27	0.60	556.7	430.9	714.8
Czech Republic	M	45	0.072	0.047	0.110	0.32	0.21	0.47	0.28	0.18	0.41	558.9	429.6	721.1
Czech Republic	M	55	0.069	0.045	0.103	0.36	0.23	0.52	0.22	0.14	0.32	572.4	438.9	737.7
Czech Republic	M	65	0.068	0.044	0.103	0.40	0.26	0.59	0.18	0.11	0.26	588.3	447.5	767.5
Czech Republic	M	75	0.068	0.045	0.102	0.44	0.29	0.64	0.16	0.10	0.24	604.8	461.2	784.7
Czech Republic	M	90	0.067	0.043	0.101	0.48	0.31	0.70	0.15	0.10	0.22	624.6	479.1	805.9
Germany	F	25	0.964	0.765	1.174	0.61	0.51	0.74	0.83	0.66	1.02	1001.8	938.3	1069.1
Germany	F	35	0.774	0.629	0.930	0.58	0.48	0.69	0.59	0.48	0.72	1009.2	952.9	1068.2
Germany	F	45	0.603	0.486	0.738	0.55	0.45	0.66	0.37	0.29	0.47	1018.3	950.2	1089.5
Germany	F	55	0.585	0.480	0.709	0.59	0.49	0.70	0.30	0.24	0.37	1040.6	980.8	1102.9
Germany	F	65	0.597	0.485	0.732	0.64	0.53	0.76	0.26	0.21	0.32	1066.3	1001.4	1135.5
Germany	F	75	0.597	0.487	0.722	0.70	0.58	0.83	0.24	0.19	0.29	1093.2	1030.3	1156.6
Germany	F	90	0.593	0.480	0.719	0.78	0.65	0.94	0.22	0.17	0.27	1127.5	1062.5	1192.5
Germany	M	25	0.778	0.630	0.951	0.58	0.48	0.70	0.97	0.78	1.21	924.0	859.9	992.9
Germany	M	35	0.630	0.521	0.758	0.53	0.44	0.62	0.68	0.55	0.85	927.8	873.5	983.3
Germany	M	45	0.497	0.403	0.607	0.49	0.40	0.58	0.42	0.33	0.53	933.5	874.3	996.3
Germany	M	55	0.472	0.387	0.574	0.52	0.43	0.62	0.33	0.26	0.40	953.4	901.9	1009.4
Germany	M	65	0.470	0.379	0.582	0.57	0.47	0.69	0.27	0.21	0.34	976.9	916.8	1045.6
Germany	M	75	0.474	0.389	0.581	0.63	0.53	0.75	0.25	0.20	0.31	1001.3	944.4	1063.3
Germany	M	90	0.481	0.396	0.582	0.70	0.59	0.84	0.23	0.19	0.28	1030.8	972.5	1091.6
Djibouti	F	25	0.029	0.013	0.054	0.46	0.25	0.81	1.47	0.75	2.62	478.1	366.4	610.0
Djibouti	F	35	0.024	0.011	0.045	0.43	0.24	0.76	1.10	0.56	1.92	480.9	372.4	609.8
Djibouti	F	45	0.020	0.009	0.037	0.41	0.22	0.72	0.75	0.37	1.34	484.8	377.2	618.7
Djibouti	F	55	0.019	0.009	0.035	0.45	0.25	0.78	0.60	0.30	1.06	497.1	387.3	628.9
Djibouti	F	65	0.019	0.009	0.035	0.51	0.28	0.88	0.49	0.25	0.86	511.2	392.8	645.0
Djibouti	F	75	0.018	0.009	0.035	0.55	0.30	0.93	0.44	0.23	0.78	524.4	403.4	662.0
Djibouti	F	90	0.018	0.009	0.034	0.59	0.32	1.00	0.41	0.21	0.73	539.3	415.3	679.1
Djibouti	M	25	0.024	0.010	0.045	0.43	0.23	0.75	1.58	0.81	2.75	434.6	333.9	562.8
Djibouti	M	35	0.020	0.009	0.037	0.40	0.21	0.68	1.18	0.61	2.07	437.5	339.6	563.0
Djibouti	M	45	0.016	0.007	0.031	0.38	0.20	0.64	0.81	0.41	1.45	441.3	338.0	568.2
Djibouti	M	55	0.015	0.007	0.030	0.42	0.22	0.71	0.64	0.33	1.14	452.2	350.4	578.6
Djibouti	M	65	0.015	0.007	0.030	0.47	0.25	0.80	0.53	0.26	0.94	464.6	357.9	596.1
Djibouti	M	75	0.015	0.007	0.029	0.50	0.27	0.86	0.48	0.24	0.86	476.6	368.1	612.7
Djibouti	M	90	0.014	0.007	0.027	0.54	0.29	0.93	0.44	0.23	0.77	489.8	379.3	629.4
Dominica	F	25	0.654	0.322	1.194	0.98	0.51	1.67	3.36	1.79	5.69	887.1	681.2	1128.4
Dominica	F	35	0.546	0.266	0.991	0.92	0.48	1.51	2.50	1.34	4.18	892.2	687.3	1137.4
Dominica	F	45	0.449	0.220	0.819	0.87	0.47	1.45	1.70	0.90	2.89	899.2	684.4	1142.8
Dominica	F	55	0.428	0.210	0.761	0.95	0.52	1.56	1.36	0.74	2.30	921.1	704.3	1165.5
Dominica	F	65	0.422	0.210	0.777	1.06	0.57	1.76	1.13	0.61	1.92	946.4	723.2	1199.8
Dominica	F	75	0.413	0.206	0.752	1.15	0.63	1.90	1.03	0.57	1.76	971.4	741.8	1228.1
Dominica	F	90	0.401	0.207	0.720	1.25	0.68	2.07	0.95	0.53	1.62	1001.6	767.7	1264.8
Dominica	M	25	0.534	0.273	0.940	0.92	0.50	1.58	3.66	2.00	6.19	814.6	633.6	1036.8
Dominica	M	35	0.444	0.223	0.791	0.86	0.47	1.47	2.71	1.51	4.56	819.3	641.8	1033.9
Dominica	M	45	0.363	0.182	0.641	0.81	0.45	1.41	1.82	1.01	3.07	825.7	644.2	1040.1
Dominica	M	55	0.349	0.180	0.622	0.89	0.50	1.51	1.46	0.81	2.46	845.6	662.4	1067.3
Dominica	M	65	0.348	0.178	0.616	0.99	0.55	1.68	1.22	0.67	2.13	868.7	677.8	1099.5
Dominica	M	75	0.339	0.173	0.603	1.07	0.60	1.79	1.11	0.62	1.91	891.8	691.2	1128.2
Dominica	M	90	0.326	0.163	0.575	1.16	0.65	1.94	1.02	0.57	1.71	919.7	719.2	1162.3
Denmark	F	25	0.569	0.288	0.990	0.75	0.42	1.28	0.99	0.55	1.61	959.6	885.4	1026.0
Denmark	F	35	0.457	0.232	0.791	0.71	0.40	1.21	0.71	0.39	1.15	966.7	900.5	1032.8
Denmark	F	45	0.357	0.180	0.615	0.68	0.39	1.16	0.45	0.25	0.73	975.3	902.4	1057.4
Denmark	F	55	0.346	0.176	0.593	0.72	0.41	1.22	0.36	0.19	0.58	996.8	930.1	1068.5
Denmark	F	65	0.352	0.181	0.602	0.78	0.44	1.32	0.31	0.17	0.50	1021.2	949.4	1100.1
Denmark	F	75	0.352	0.180	0.604	0.86	0.48	1.45	0.28	0.15	0.46	1047.0	978.2	1119.2
Denmark	F	90	0.350	0.177	0.616	0.96	0.53	1.63	0.26	0.14	0.42	1080.1	1008.2	1155.8
Denmark	M	25	0.455	0.234	0.784	0.72	0.39	1.19	1.15	0.64	1.87	881.4	814.1	954.2
Denmark	M	35	0.368	0.189	0.645	0.65	0.36	1.09	0.81	0.45	1.32	885.0	823.5	951.4
Denmark	M	45	0.291	0.145	0.523	0.60	0.33	1.03	0.50	0.27	0.83	890.4	820.3	962.4
Denmark	M	55	0.276	0.137	0.486	0.64	0.35	1.08	0.39	0.21	0.64	909.5	848.7	975.0
Denmark	M	65	0.274	0.137	0.486	0.70	0.39	1.19	0.32	0.18	0.52	931.7	860.4	1004.1
Denmark	M	75	0.277	0.139	0.486	0.78	0.42	1.31	0.29	0.17	0.48	955.0	889.1	1024.1
Denmark	M	90	0.281	0.144	0.494	0.87	0.47	1.49	0.27	0.15	0.45	983.8	917.1	1059.7
Dominican Republic	F	25	0.710	0.350	1.311	0.58	0.32	1.00	3.76	2.08	6.29	786.7	602.5	1008.1
Dominican Republic	F	35	0.594	0.299	1.095	0.55	0.31	0.92	2.80	1.57	4.66	791.2	608.4	1021.9
Dominican Republic	F	45	0.489	0.244	0.917	0.52	0.29	0.86	1.90	1.06	3.13	797.3	613.4	1029.3
Dominican Republic	F	55	0.466	0.235	0.874	0.57	0.32	0.95	1.52	0.86	2.47	816.8	627.6	1052.7

Country	Sex	Midpoint of age range	Mean juice intake (servings/day)	Juice, lower uncertainty interval	Juice, upper uncertainty interval	Mean milk intake (servings/day)	Milk, lower uncertainty interval	Milk, upper uncertainty interval	Mean SSB intake (servings/day)	SSB, lower uncertainty interval	SSB, upper uncertainty interval	Mean calcium intake (servings/day)	Calcium, lower uncertainty interval	Calcium, upper uncertainty interval
Dominican Republic	F	65	0.460	0.229	0.847	0.63	0.35	1.07	1.27	0.74	2.07	839.2	640.7	1079.7
Dominican Republic	F	75	0.450	0.222	0.828	0.69	0.38	1.17	1.16	0.67	1.91	861.5	668.3	1102.3
Dominican Republic	F	90	0.437	0.218	0.807	0.75	0.42	1.27	1.06	0.60	1.78	888.2	689.2	1141.3
Dominican Republic	M	25	0.569	0.284	0.994	0.54	0.30	0.91	4.10	2.20	6.94	714.4	553.2	919.6
Dominican Republic	M	35	0.474	0.238	0.831	0.50	0.29	0.84	3.04	1.66	5.05	718.5	559.7	916.7
Dominican Republic	M	45	0.388	0.193	0.689	0.48	0.27	0.82	2.05	1.11	3.37	724.1	561.7	925.8
Dominican Republic	M	55	0.372	0.187	0.654	0.52	0.30	0.88	1.64	0.91	2.67	741.5	577.9	936.5
Dominican Republic	M	65	0.371	0.183	0.647	0.58	0.33	0.98	1.37	0.76	2.25	761.7	589.2	962.4
Dominican Republic	M	75	0.362	0.179	0.632	0.63	0.36	1.06	1.25	0.69	2.04	782.1	605.0	986.2
Dominican Republic	M	90	0.348	0.177	0.611	0.68	0.39	1.14	1.15	0.62	1.86	806.6	629.2	1017.1
Algeria	F	25	0.204	0.103	0.370	1.01	0.73	1.38	0.71	0.37	1.26	674.2	517.6	862.3
Algeria	F	35	0.171	0.087	0.307	0.96	0.69	1.29	0.55	0.29	0.96	678.7	529.1	866.5
Algeria	F	45	0.140	0.071	0.258	0.92	0.66	1.25	0.39	0.20	0.68	684.7	533.7	884.9
Algeria	F	55	0.134	0.070	0.240	1.01	0.73	1.35	0.32	0.17	0.55	701.9	545.5	906.2
Algeria	F	65	0.134	0.069	0.236	1.12	0.81	1.51	0.27	0.14	0.47	721.5	560.0	929.2
Algeria	F	75	0.131	0.068	0.232	1.21	0.88	1.62	0.24	0.13	0.42	740.0	575.7	948.6
Algeria	F	90	0.128	0.065	0.225	1.31	0.95	1.76	0.22	0.12	0.38	761.1	590.2	979.7
Algeria	M	25	0.167	0.084	0.304	0.92	0.67	1.24	0.81	0.44	1.37	613.6	461.6	784.1
Algeria	M	35	0.139	0.070	0.257	0.86	0.65	1.15	0.61	0.33	1.00	617.4	467.6	784.4
Algeria	M	45	0.114	0.056	0.211	0.82	0.60	1.11	0.42	0.23	0.70	622.5	470.5	804.6
Algeria	M	55	0.110	0.056	0.205	0.90	0.67	1.20	0.34	0.19	0.56	637.9	479.8	816.1
Algeria	M	65	0.110	0.054	0.207	1.01	0.73	1.36	0.28	0.15	0.47	655.6	487.9	838.5
Algeria	M	75	0.107	0.052	0.203	1.10	0.81	1.47	0.26	0.14	0.43	672.2	503.5	856.3
Algeria	M	90	0.103	0.050	0.194	1.20	0.90	1.61	0.24	0.13	0.40	690.4	524.1	880.3
Ecuador	F	25	0.633	0.309	1.154	0.74	0.38	1.26	1.34	0.69	2.34	696.2	525.6	910.2
Ecuador	F	35	0.526	0.258	0.953	0.69	0.36	1.17	1.00	0.52	1.71	700.8	535.5	907.1
Ecuador	F	45	0.429	0.208	0.791	0.65	0.33	1.13	0.68	0.35	1.17	706.9	537.7	910.3
Ecuador	F	55	0.412	0.204	0.739	0.71	0.37	1.22	0.54	0.28	0.92	725.0	557.6	930.2
Ecuador	F	65	0.411	0.201	0.765	0.80	0.40	1.37	0.44	0.24	0.77	745.6	570.8	962.6
Ecuador	F	75	0.403	0.202	0.740	0.86	0.45	1.48	0.40	0.22	0.69	764.6	591.4	977.2
Ecuador	F	90	0.391	0.196	0.697	0.94	0.49	1.63	0.37	0.20	0.63	786.9	610.5	1014.8
Ecuador	M	25	0.516	0.250	0.962	0.69	0.36	1.15	1.45	0.78	2.43	640.3	499.2	822.2
Ecuador	M	35	0.428	0.210	0.787	0.64	0.35	1.08	1.08	0.59	1.79	644.0	506.2	821.8
Ecuador	M	45	0.347	0.164	0.675	0.61	0.33	1.05	0.73	0.38	1.23	649.1	506.9	836.2
Ecuador	M	55	0.335	0.162	0.619	0.67	0.36	1.15	0.58	0.32	0.96	664.9	522.6	843.4
Ecuador	M	65	0.337	0.161	0.643	0.75	0.41	1.26	0.48	0.26	0.79	683.0	533.0	873.4
Ecuador	M	75	0.329	0.157	0.612	0.81	0.45	1.37	0.44	0.24	0.72	700.9	551.3	894.0
Ecuador	M	90	0.317	0.154	0.574	0.88	0.48	1.49	0.40	0.22	0.66	722.2	563.5	931.7
Egypt	F	25	0.197	0.098	0.344	0.57	0.39	0.78	0.60	0.33	1.04	586.5	455.6	752.1
Egypt	F	35	0.164	0.083	0.283	0.54	0.38	0.73	0.46	0.25	0.78	590.6	460.1	752.8
Egypt	F	45	0.135	0.067	0.238	0.52	0.36	0.71	0.33	0.17	0.56	596.0	462.4	764.2
Egypt	F	55	0.129	0.065	0.225	0.57	0.40	0.77	0.27	0.14	0.45	610.8	471.2	786.0
Egypt	F	65	0.129	0.065	0.222	0.63	0.44	0.87	0.22	0.12	0.38	627.6	481.6	811.7
Egypt	F	75	0.127	0.064	0.215	0.69	0.47	0.93	0.20	0.11	0.35	643.7	499.5	830.7
Egypt	F	90	0.123	0.061	0.209	0.74	0.51	1.02	0.18	0.10	0.31	661.9	517.3	848.7
Egypt	M	25	0.166	0.084	0.301	0.52	0.36	0.71	0.66	0.37	1.12	537.6	416.9	689.2
Egypt	M	35	0.139	0.070	0.247	0.49	0.35	0.67	0.49	0.28	0.83	540.9	421.3	682.8
Egypt	M	45	0.113	0.056	0.202	0.47	0.33	0.65	0.34	0.19	0.56	545.4	423.6	693.4
Egypt	M	55	0.109	0.055	0.190	0.51	0.36	0.71	0.28	0.15	0.46	558.8	434.2	706.9
Egypt	M	65	0.109	0.055	0.194	0.57	0.39	0.80	0.23	0.13	0.40	574.2	444.5	731.3
Egypt	M	75	0.106	0.054	0.188	0.62	0.43	0.87	0.21	0.12	0.36	588.7	457.2	742.5
Egypt	M	90	0.102	0.051	0.181	0.68	0.47	0.95	0.20	0.11	0.33	604.9	471.6	761.8
Eritrea	F	25	0.000	0.000	0.000	0.19	0.10	0.32	0.49	0.26	0.81	388.1	298.5	493.8
Eritrea	F	35	0.000	0.000	0.000	0.18	0.10	0.30	0.37	0.19	0.60	390.3	298.2	489.7
Eritrea	F	45	0.000	0.000	0.000	0.17	0.09	0.29	0.25	0.13	0.43	393.5	297.3	496.7
Eritrea	F	55	0.000	0.000	0.000	0.18	0.10	0.32	0.20	0.11	0.33	403.6	307.0	509.4
Eritrea	F	65	0.000	0.000	0.000	0.20	0.11	0.36	0.16	0.09	0.27	415.0	317.1	526.4
Eritrea	F	75	0.000	0.000	0.000	0.22	0.12	0.38	0.15	0.08	0.24	425.7	325.5	536.6
Eritrea	F	90	0.000	0.000	0.000	0.24	0.13	0.41	0.14	0.07	0.22	438.1	332.7	550.3
Eritrea	M	25	0.000	0.000	0.000	0.17	0.10	0.29	0.54	0.29	0.92	353.6	278.1	451.0
Eritrea	M	35	0.000	0.000	0.000	0.16	0.09	0.27	0.41	0.22	0.69	356.0	280.3	453.1
Eritrea	M	45	0.000	0.000	0.000	0.15	0.08	0.26	0.28	0.15	0.48	359.1	280.2	457.5
Eritrea	M	55	0.000	0.000	0.000	0.17	0.09	0.29	0.22	0.12	0.37	367.9	290.6	468.6
Eritrea	M	65	0.000	0.000	0.000	0.19	0.10	0.32	0.18	0.10	0.31	378.0	297.2	484.9
Eritrea	M	75	0.000	0.000	0.000	0.20	0.11	0.35	0.16	0.09	0.28	387.7	306.7	495.9
Eritrea	M	90	0.000	0.000	0.000	0.22	0.12	0.37	0.15	0.08	0.25	398.7	314.8	509.3
Spain	F	25	0.157	0.089	0.251	0.95	0.71	1.23	0.53	0.34	0.75	877.5	804.6	955.9
Spain	F	35	0.127	0.072	0.202	0.89	0.68	1.14	0.38	0.24	0.54	883.9	813.3	960.6
Spain	F	45	0.099	0.057	0.159	0.85	0.65	1.11	0.24	0.16	0.35	891.8	812.3	973.7
Spain	F	55	0.096	0.055	0.153	0.91	0.69	1.16	0.19	0.13	0.27	911.4	835.7	988.8
Spain	F	65	0.098	0.056	0.160	0.99	0.74	1.27	0.16	0.10	0.24	933.9	848.3	1021.7
Spain	F	75	0.098	0.056	0.158	1.08	0.82	1.40	0.15	0.10	0.22	957.6	880.8	1044.2
Spain	F	90	0.097	0.056	0.156	1.21	0.91	1.57	0.14	0.09	0.20	987.3	910.9	1069.4
Spain	M	25	0.125	0.074	0.200	0.90	0.67	1.16	0.63	0.40	0.94	808.1	737.1	878.7
Spain	M	35	0.101	0.059	0.160	0.82	0.63	1.04	0.45	0.29	0.67	811.4	747.2	877.9
Spain	M	45	0.080	0.046	0.128	0.75	0.57	0.95	0.27	0.18	0.42	816.3	750.5	890.4
Spain	M	55	0.076	0.045	0.120	0.80	0.61	1.02	0.21	0.14	0.32	833.8	773.3	901.1
Spain	M	65	0.075	0.045	0.119	0.88	0.66	1.14	0.18	0.11	0.26	854.2	785.9	927.8
Spain	M	75	0.076	0.045	0.122	0.97	0.74	1.24	0.16	0.10	0.24	875.8	807.6	948.1
Spain	M	90	0.077	0.045	0.124	1.09	0.83	1.39	0.15	0.10	0.23	901.9	829.8	979.1
Estonia	F	25	0.266	0.133	0.504	0.94	0.75	1.14	0.79	0.42	1.31	673.1	618.5	732.3
Estonia	F	35	0.220	0.110	0.419	0.89	0.73	1.07	0.59	0.32	0.96	678.7	630.5	729.7
Estonia	F	45	0.177	0.085	0.339	0.86	0.70	1.06	0.40	0.22	0.68	685.6	632.6	747.0
Estonia	F	55	0.171	0.083	0.323	0.95	0.78	1.14	0.32	0.18	0.53	701.3	653.3	756.1
Estonia	F	65	0.171	0.084	0.337	1.06	0.85	1.28	0.26	0.14	0.43	719.2	665.1	779.1
Estonia	F	75	0.167	0.084	0.324	1.14	0.93	1.37	0.24	0.13	0.39	737.5	687.9	792.3
Estonia	F	90	0.162	0.082	0.306	1.23	1.01	1.50	0.22	0.12	0.36	760.0	706.5	820.0
Estonia	M	25	0.213	0.107	0.374	0.86	0.69	1.05	0.87	0.47	1.43	615.0	571.6	666.5
Estonia	M	35	0.176	0.093	0.302	0.81	0.66	0.97	0.65	0.36	1.06	618.0	577.1	663.1
Estonia	M	45	0.142	0.074	0.248	0.78	0.63	0.96	0.44	0.24	0.73	622.5	575.1	672.3
Estonia	M	55	0.136	0.072	0.234	0.88	0.72	1.06	0.35	0.19	0.57	639.0	594.5	687.4
Estonia	M	65	0.136	0.071	0.239	1.00	0.81	1.22	0.29	0.16	0.48	658.1	608.4	711.4
Estonia	M	75	0.134	0.070	0.233	1.07	0.88	1.28	0.26	0.14	0.43	675.8	631.1	726.0
Estonia	M	90	0.129	0.069	0.227	1.13	0.92	1.35	0.24	0.14	0.39	696.0	652.6	747.4
Ethiopia	F	25	0.002	0.001	0.004	0.20	0.11	0.34	0.34	0.19	0.58	394.4	296.4	507.2
Ethiopia	F	35	0.002	0.001	0.003	0.19	0.11	0.32	0.26	0.14	0.43	396.8	297.1	508.8
Ethiopia	F	45	0.001	0.001	0.002	0.18	0.10	0.31	0.17	0.10	0.30	400.0	297.2	516.4
Ethiopia	F	55	0.001	0.001	0.002	0.20	0.11	0.34	0.14	0.08	0.24	410.2	306.8	525.6
Ethiopia	F	65	0.001	0.001	0.002	0.22	0.12	0.37	0.11	0.06	0.19	421.8	317.8	541.0

Country	Sex	Midpoint of age range	Mean juice intake (servings/day)	Juice, lower uncertainty interval	Juice, upper uncertainty interval	Mean milk intake (servings/day)	Milk, lower uncertainty interval	Milk, upper uncertainty interval	Mean SSB intake (servings/day)	SSB, lower uncertainty interval	SSB, upper uncertainty interval	Mean calcium intake (servings/day)	Calcium, lower uncertainty interval	Calcium, upper uncertainty interval
Ethiopia	F	75	0.001	0.001	0.002	0.24	0.13	0.40	0.10	0.06	0.17	432.7	328.4	553.9
Ethiopia	F	90	0.001	0.001	0.002	0.26	0.15	0.43	0.10	0.05	0.16	445.0	336.0	569.8
Ethiopia	M	25	0.001	0.001	0.003	0.18	0.10	0.30	0.38	0.20	0.64	361.8	276.0	470.6
Ethiopia	M	35	0.001	0.001	0.002	0.17	0.09	0.28	0.28	0.15	0.47	364.2	277.7	468.8
Ethiopia	M	45	0.001	0.000	0.002	0.16	0.09	0.26	0.19	0.10	0.32	367.4	277.8	475.2
Ethiopia	M	55	0.001	0.000	0.002	0.18	0.10	0.29	0.15	0.08	0.25	376.5	284.7	482.5
Ethiopia	M	65	0.001	0.000	0.002	0.20	0.11	0.33	0.12	0.06	0.21	386.7	295.3	497.2
Ethiopia	M	75	0.001	0.000	0.002	0.22	0.12	0.35	0.11	0.06	0.19	396.7	300.5	508.8
Ethiopia	M	90	0.001	0.000	0.002	0.23	0.13	0.38	0.10	0.05	0.18	407.9	308.7	522.7
Finland	F	25	0.338	0.269	0.411	1.40	1.18	1.65	0.81	0.67	0.97	1129.8	1047.6	1217.2
Finland	F	35	0.272	0.222	0.328	1.32	1.13	1.54	0.58	0.48	0.69	1138.1	1067.3	1216.0
Finland	F	45	0.212	0.173	0.260	1.26	1.04	1.49	0.37	0.30	0.44	1148.4	1065.1	1233.8
Finland	F	55	0.205	0.169	0.249	1.34	1.14	1.57	0.29	0.24	0.35	1173.8	1098.4	1257.3
Finland	F	65	0.210	0.170	0.256	1.45	1.23	1.72	0.25	0.21	0.31	1202.4	1116.0	1297.5
Finland	F	75	0.210	0.172	0.251	1.60	1.37	1.87	0.23	0.19	0.28	1232.9	1151.2	1324.2
Finland	F	90	0.208	0.170	0.254	1.79	1.52	2.09	0.21	0.17	0.26	1271.8	1182.5	1359.8
Finland	M	25	0.272	0.221	0.330	1.34	1.13	1.59	0.95	0.77	1.14	1039.5	967.3	1111.4
Finland	M	35	0.221	0.181	0.262	1.22	1.04	1.42	0.67	0.55	0.80	1043.8	980.5	1108.7
Finland	M	45	0.174	0.140	0.210	1.12	0.94	1.32	0.41	0.34	0.50	1050.3	978.4	1125.8
Finland	M	55	0.165	0.135	0.197	1.20	1.02	1.38	0.32	0.26	0.39	1072.9	1005.8	1142.6
Finland	M	65	0.164	0.133	0.199	1.31	1.10	1.54	0.27	0.22	0.32	1098.9	1020.9	1176.5
Finland	M	75	0.166	0.136	0.199	1.45	1.24	1.68	0.24	0.20	0.29	1126.6	1050.3	1202.1
Finland	M	90	0.168	0.138	0.205	1.62	1.38	1.90	0.23	0.19	0.27	1160.1	1085.4	1240.0
Fiji	F	25	0.071	0.032	0.129	0.34	0.19	0.58	1.15	0.61	1.91	515.2	394.6	661.8
Fiji	F	35	0.059	0.028	0.107	0.32	0.18	0.53	0.86	0.47	1.43	518.1	399.3	662.4
Fiji	F	45	0.048	0.022	0.089	0.30	0.17	0.51	0.58	0.32	1.00	522.1	400.1	673.5
Fiji	F	55	0.046	0.022	0.084	0.33	0.19	0.56	0.46	0.26	0.79	535.4	411.1	687.5
Fiji	F	65	0.046	0.021	0.084	0.37	0.21	0.63	0.38	0.21	0.64	550.6	420.7	708.0
Fiji	F	75	0.045	0.021	0.082	0.40	0.23	0.67	0.35	0.19	0.58	565.0	433.1	724.3
Fiji	F	90	0.044	0.020	0.081	0.43	0.25	0.71	0.32	0.18	0.53	581.2	447.7	736.3
Fiji	M	25	0.060	0.028	0.111	0.32	0.17	0.55	1.27	0.67	2.14	467.4	354.5	607.4
Fiji	M	35	0.050	0.024	0.090	0.30	0.16	0.52	0.95	0.51	1.58	469.9	357.2	605.4
Fiji	M	45	0.040	0.019	0.074	0.28	0.15	0.50	0.65	0.33	1.10	473.5	357.9	614.5
Fiji	M	55	0.039	0.019	0.070	0.31	0.17	0.54	0.52	0.27	0.86	485.8	367.4	627.9
Fiji	M	65	0.039	0.019	0.069	0.34	0.18	0.60	0.43	0.22	0.71	499.9	377.9	646.1
Fiji	M	75	0.038	0.019	0.068	0.37	0.20	0.65	0.39	0.20	0.64	513.0	390.0	661.2
Fiji	M	90	0.037	0.018	0.066	0.40	0.22	0.70	0.36	0.19	0.58	527.3	403.7	676.2
France	F	25	0.326	0.258	0.408	0.52	0.43	0.62	0.53	0.42	0.65	903.9	841.9	969.4
France	F	35	0.262	0.209	0.323	0.49	0.42	0.57	0.38	0.30	0.46	910.6	854.4	966.9
France	F	45	0.204	0.162	0.257	0.47	0.39	0.56	0.24	0.19	0.30	918.8	856.4	982.9
France	F	55	0.198	0.159	0.244	0.50	0.42	0.58	0.19	0.15	0.23	939.0	881.0	996.0
France	F	65	0.202	0.160	0.249	0.54	0.46	0.64	0.16	0.13	0.20	961.9	896.1	1026.8
France	F	75	0.202	0.161	0.247	0.60	0.51	0.69	0.15	0.12	0.18	986.5	923.3	1047.1
France	F	90	0.200	0.158	0.251	0.67	0.56	0.78	0.14	0.11	0.17	1017.7	953.7	1079.4
France	M	25	0.263	0.206	0.332	0.50	0.42	0.59	0.62	0.50	0.76	830.2	771.4	890.9
France	M	35	0.213	0.170	0.264	0.45	0.39	0.53	0.44	0.36	0.53	833.6	780.9	887.0
France	M	45	0.168	0.133	0.211	0.42	0.35	0.49	0.27	0.21	0.33	838.7	780.9	900.9
France	M	55	0.159	0.127	0.198	0.44	0.38	0.52	0.21	0.17	0.26	856.7	807.7	910.0
France	M	65	0.159	0.123	0.198	0.49	0.41	0.58	0.17	0.14	0.22	877.5	819.2	934.8
France	M	75	0.160	0.128	0.200	0.54	0.46	0.63	0.16	0.13	0.20	899.7	846.5	955.0
France	M	90	0.163	0.131	0.208	0.61	0.51	0.71	0.15	0.12	0.18	926.9	870.6	986.0
Micronesia (Federated States of)	F	25	0.165	0.082	0.289	0.24	0.12	0.40	0.93	0.48	1.58	468.3	362.0	604.8
Micronesia (Federated States of)	F	35	0.137	0.070	0.237	0.22	0.12	0.37	0.69	0.37	1.15	471.0	364.4	613.5
Micronesia (Federated States of)	F	45	0.112	0.054	0.197	0.21	0.11	0.35	0.47	0.25	0.79	474.8	359.4	621.8
Micronesia (Federated States of)	F	55	0.107	0.054	0.190	0.23	0.12	0.39	0.37	0.20	0.62	486.8	378.5	628.9
Micronesia (Federated States of)	F	65	0.107	0.054	0.192	0.26	0.14	0.44	0.31	0.17	0.52	500.4	388.2	638.3
Micronesia (Federated States of)	F	75	0.105	0.053	0.186	0.28	0.15	0.47	0.28	0.15	0.47	513.7	398.3	651.1
Micronesia (Federated States of)	F	90	0.102	0.050	0.180	0.30	0.16	0.51	0.26	0.14	0.43	528.9	411.1	674.5
Micronesia (Federated States of)	M	25	0.140	0.070	0.263	0.21	0.12	0.37	1.01	0.55	1.66	432.7	328.0	552.1
Micronesia (Federated States of)	M	35	0.116	0.057	0.214	0.20	0.11	0.34	0.76	0.42	1.25	435.0	331.5	555.8
Micronesia (Federated States of)	M	45	0.093	0.045	0.173	0.19	0.10	0.33	0.52	0.29	0.87	438.4	333.1	560.5
Micronesia (Federated States of)	M	55	0.090	0.044	0.160	0.21	0.11	0.36	0.41	0.23	0.70	449.8	342.1	574.9
Micronesia (Federated States of)	M	65	0.090	0.044	0.161	0.23	0.13	0.40	0.34	0.19	0.56	462.8	350.6	592.8
Micronesia (Federated States of)	M	75	0.088	0.043	0.157	0.25	0.14	0.44	0.31	0.17	0.50	475.1	360.8	609.2
Micronesia (Federated States of)	M	90	0.086	0.043	0.156	0.27	0.15	0.47	0.28	0.16	0.47	488.5	372.4	623.3
Gabon	F	25	1.023	0.452	2.206	1.11	0.55	2.02	1.04	0.55	1.75	576.4	424.2	787.3
Gabon	F	35	0.849	0.386	1.742	1.04	0.53	1.86	0.78	0.43	1.33	580.3	428.9	785.6
Gabon	F	45	0.691	0.304	1.401	0.99	0.49	1.78	0.54	0.29	0.93	585.5	431.2	786.5
Gabon	F	55	0.663	0.301	1.344	1.08	0.54	1.94	0.43	0.23	0.73	600.8	445.5	805.7
Gabon	F	65	0.662	0.296	1.417	1.21	0.60	2.14	0.35	0.19	0.60	618.0	457.2	832.2
Gabon	F	75	0.647	0.286	1.369	1.31	0.65	2.28	0.32	0.17	0.54	634.1	472.4	853.0
Gabon	F	90	0.625	0.279	1.279	1.42	0.71	2.52	0.29	0.16	0.49	652.6	484.8	881.8
Gabon	M	25	0.801	0.347	1.567	1.03	0.52	1.78	1.17	0.62	2.04	526.3	393.6	704.7
Gabon	M	35	0.665	0.298	1.282	0.96	0.50	1.70	0.87	0.46	1.50	530.0	394.5	706.7
Gabon	M	45	0.542	0.237	1.047	0.91	0.46	1.60	0.59	0.31	1.05	534.9	394.8	713.9
Gabon	M	55	0.521	0.231	1.020	1.00	0.52	1.72	0.47	0.25	0.83	548.3	412.4	730.6
Gabon	M	65	0.522	0.221	1.016	1.11	0.58	1.92	0.38	0.20	0.66	563.6	422.1	750.3
Gabon	M	75	0.511	0.220	1.007	1.21	0.62	2.09	0.35	0.18	0.60	578.0	435.9	762.9
Gabon	M	90	0.496	0.219	0.953	1.31	0.66	2.29	0.32	0.17	0.55	594.9	445.5	781.0
United Kingdom	F	25	0.320	0.266	0.380	0.94	0.79	1.09	0.91	0.76	1.07	872.9	822.1	926.0
United Kingdom	F	35	0.257	0.217	0.299	0.89	0.77	1.02	0.65	0.56	0.75	879.3	836.5	923.4
United Kingdom	F	45	0.201	0.167	0.238	0.85	0.72	0.99	0.41	0.34	0.49	887.2	835.1	943.8
United Kingdom	F	55	0.195	0.166	0.228	0.90	0.79	1.04	0.33	0.28	0.39	906.7	862.7	953.6
United Kingdom	F	65	0.199	0.167	0.235	0.98	0.84	1.14	0.28	0.24	0.34	928.9	876.8	983.0
United Kingdom	F	75	0.198	0.168	0.231	1.08	0.93	1.24	0.26	0.22	0.30	952.4	906.3	999.8

Country	Sex	Midpoint of age range	Mean juice intake (servings/day)	Juice, lower uncertainty interval	Juice, upper uncertainty interval	Mean milk intake (servings/day)	Milk, lower uncertainty interval	Milk, upper uncertainty interval	Mean SSB intake (servings/day)	SSB, lower uncertainty interval	SSB, upper uncertainty interval	Mean calcium intake (servings/day)	Calcium, lower uncertainty interval	Calcium, upper uncertainty interval
United Kingdom	F	90	0.197	0.163	0.229	1.20	1.04	1.39	0.24	0.20	0.28	982.5	934.4	1030.9
United Kingdom	M	25	0.257	0.216	0.307	0.90	0.77	1.05	1.06	0.89	1.26	803.4	755.0	851.4
United Kingdom	M	35	0.209	0.178	0.244	0.82	0.71	0.93	0.75	0.63	0.87	806.7	767.2	848.4
United Kingdom	M	45	0.164	0.138	0.196	0.75	0.64	0.87	0.46	0.38	0.56	811.6	764.0	859.6
United Kingdom	M	55	0.156	0.133	0.183	0.80	0.69	0.92	0.36	0.30	0.43	829.0	788.2	870.5
United Kingdom	M	65	0.155	0.130	0.185	0.88	0.75	1.02	0.30	0.25	0.35	849.3	801.7	900.2
United Kingdom	M	75	0.157	0.133	0.184	0.97	0.84	1.11	0.27	0.23	0.32	870.7	826.0	913.7
United Kingdom	M	90	0.159	0.134	0.190	1.09	0.94	1.25	0.25	0.21	0.30	896.8	849.1	946.6
Georgia	F	25	0.303	0.148	0.558	0.50	0.33	0.71	1.02	0.51	1.85	676.9	522.0	864.1
Georgia	F	35	0.251	0.124	0.447	0.46	0.32	0.67	0.76	0.39	1.37	680.9	524.9	859.8
Georgia	F	45	0.204	0.101	0.371	0.44	0.29	0.65	0.52	0.26	0.96	686.4	524.2	862.9
Georgia	F	55	0.197	0.098	0.351	0.48	0.32	0.70	0.41	0.21	0.73	702.7	541.7	884.3
Georgia	F	65	0.197	0.097	0.356	0.54	0.36	0.78	0.34	0.17	0.60	721.7	551.7	918.8
Georgia	F	75	0.193	0.097	0.347	0.58	0.39	0.84	0.31	0.15	0.54	740.8	570.7	938.4
Georgia	F	90	0.186	0.093	0.331	0.64	0.43	0.91	0.28	0.14	0.51	764.2	594.3	966.9
Georgia	M	25	0.248	0.122	0.458	0.46	0.31	0.68	1.13	0.57	2.03	613.8	474.1	785.8
Georgia	M	35	0.205	0.103	0.372	0.43	0.29	0.62	0.85	0.44	1.49	617.9	478.2	795.9
Georgia	M	45	0.166	0.080	0.303	0.41	0.27	0.59	0.58	0.30	1.03	623.3	479.9	802.6
Georgia	M	55	0.160	0.079	0.291	0.45	0.31	0.64	0.46	0.25	0.83	638.7	492.6	824.0
Georgia	M	65	0.159	0.079	0.291	0.50	0.34	0.73	0.38	0.20	0.69	656.4	505.9	846.1
Georgia	M	75	0.156	0.077	0.284	0.54	0.38	0.77	0.34	0.18	0.61	673.7	518.7	866.7
Georgia	M	90	0.152	0.076	0.278	0.58	0.40	0.83	0.32	0.17	0.55	693.6	536.1	890.5
Ghana	F	25	0.136	0.058	0.257	0.12	0.07	0.21	0.64	0.33	1.12	386.2	302.7	490.9
Ghana	F	35	0.113	0.051	0.214	0.12	0.07	0.20	0.48	0.25	0.82	388.4	305.5	493.9
Ghana	F	45	0.092	0.042	0.177	0.11	0.06	0.19	0.33	0.17	0.56	391.6	303.3	503.2
Ghana	F	55	0.088	0.040	0.168	0.12	0.07	0.20	0.26	0.14	0.44	401.5	315.9	517.2
Ghana	F	65	0.088	0.039	0.168	0.14	0.08	0.23	0.21	0.11	0.37	412.8	324.6	530.5
Ghana	F	75	0.086	0.038	0.163	0.15	0.08	0.25	0.19	0.11	0.33	423.4	333.5	541.5
Ghana	F	90	0.083	0.038	0.158	0.16	0.09	0.26	0.18	0.10	0.31	435.4	344.6	552.0
Ghana	M	25	0.112	0.050	0.222	0.12	0.06	0.20	0.72	0.39	1.25	356.8	267.9	458.8
Ghana	M	35	0.093	0.041	0.179	0.11	0.06	0.18	0.54	0.29	0.94	359.0	273.6	458.3
Ghana	M	45	0.076	0.033	0.148	0.10	0.06	0.18	0.36	0.20	0.63	361.9	275.7	465.7
Ghana	M	55	0.073	0.032	0.141	0.11	0.06	0.19	0.29	0.16	0.49	370.8	283.2	475.9
Ghana	M	65	0.073	0.033	0.144	0.13	0.07	0.21	0.24	0.13	0.41	381.0	288.5	490.5
Ghana	M	75	0.072	0.033	0.141	0.14	0.08	0.23	0.22	0.12	0.37	390.9	298.4	501.3
Ghana	M	90	0.070	0.032	0.135	0.15	0.08	0.25	0.20	0.11	0.34	402.2	306.2	515.5
Guinea	F	25	0.084	0.039	0.155	0.23	0.13	0.38	0.70	0.35	1.21	439.5	342.0	555.9
Guinea	F	35	0.070	0.033	0.130	0.21	0.12	0.35	0.52	0.28	0.89	442.2	343.3	561.0
Guinea	F	45	0.057	0.026	0.109	0.20	0.11	0.34	0.36	0.19	0.63	445.7	343.2	566.8
Guinea	F	55	0.055	0.026	0.102	0.22	0.13	0.37	0.28	0.15	0.49	457.1	355.2	579.8
Guinea	F	65	0.054	0.026	0.102	0.25	0.14	0.42	0.23	0.12	0.40	470.1	364.0	597.5
Guinea	F	75	0.053	0.026	0.100	0.27	0.15	0.45	0.21	0.11	0.36	482.1	372.6	612.2
Guinea	F	90	0.052	0.025	0.097	0.29	0.16	0.49	0.19	0.10	0.33	495.6	382.5	627.3
Guinea	M	25	0.066	0.031	0.125	0.21	0.11	0.36	0.78	0.43	1.36	405.4	310.1	519.6
Guinea	M	35	0.055	0.026	0.101	0.20	0.11	0.34	0.58	0.33	0.99	408.0	312.0	523.7
Guinea	M	45	0.045	0.021	0.085	0.19	0.10	0.31	0.40	0.22	0.67	411.4	313.2	531.8
Guinea	M	55	0.043	0.020	0.081	0.21	0.12	0.34	0.31	0.18	0.53	421.4	324.9	543.8
Guinea	M	65	0.043	0.019	0.081	0.23	0.13	0.39	0.26	0.14	0.43	432.9	331.5	560.3
Guinea	M	75	0.042	0.020	0.079	0.25	0.14	0.42	0.23	0.13	0.39	444.2	342.3	572.2
Guinea	M	90	0.041	0.020	0.076	0.27	0.15	0.45	0.22	0.12	0.37	456.9	352.4	588.0
Gambia	F	25	0.003	0.001	0.006	0.36	0.20	0.62	1.79	0.91	3.18	487.6	374.4	626.7
Gambia	F	35	0.002	0.001	0.005	0.34	0.18	0.58	1.34	0.70	2.39	490.3	379.9	625.7
Gambia	F	45	0.002	0.001	0.004	0.32	0.17	0.56	0.91	0.48	1.62	494.2	382.7	628.4
Gambia	F	55	0.002	0.001	0.004	0.36	0.20	0.61	0.73	0.38	1.28	506.7	393.0	647.7
Gambia	F	65	0.002	0.001	0.004	0.40	0.22	0.68	0.60	0.31	1.05	521.2	399.5	672.7
Gambia	F	75	0.002	0.001	0.004	0.43	0.24	0.72	0.54	0.28	0.95	534.5	411.8	685.7
Gambia	F	90	0.002	0.001	0.004	0.46	0.25	0.79	0.50	0.25	0.89	548.8	422.2	705.5
Gambia	M	25	0.002	0.001	0.005	0.33	0.17	0.56	2.01	1.00	3.51	447.6	345.5	567.5
Gambia	M	35	0.002	0.001	0.004	0.31	0.16	0.52	1.50	0.76	2.64	450.4	350.8	569.6
Gambia	M	45	0.001	0.000	0.003	0.29	0.15	0.50	1.02	0.52	1.79	454.2	351.4	576.0
Gambia	M	55	0.001	0.000	0.003	0.32	0.17	0.53	0.81	0.41	1.41	465.3	359.0	589.4
Gambia	M	65	0.001	0.000	0.003	0.36	0.19	0.60	0.67	0.32	1.17	478.0	364.9	609.3
Gambia	M	75	0.001	0.000	0.003	0.39	0.21	0.65	0.60	0.29	1.06	490.4	375.2	625.4
Gambia	M	90	0.001	0.000	0.003	0.42	0.23	0.71	0.56	0.28	0.97	503.8	389.2	646.0
Guinea-Bissau	F	25	0.042	0.021	0.075	0.27	0.14	0.47	0.79	0.41	1.33	457.1	358.0	596.0
Guinea-Bissau	F	35	0.035	0.018	0.061	0.25	0.14	0.43	0.59	0.32	0.98	459.8	361.0	594.9
Guinea-Bissau	F	45	0.028	0.014	0.050	0.24	0.13	0.40	0.40	0.22	0.69	463.5	360.7	590.9
Guinea-Bissau	F	55	0.027	0.013	0.048	0.27	0.15	0.44	0.32	0.17	0.54	475.3	375.1	605.7
Guinea-Bissau	F	65	0.027	0.013	0.049	0.30	0.16	0.49	0.26	0.14	0.45	488.7	382.0	627.1
Guinea-Bissau	F	75	0.026	0.013	0.047	0.32	0.17	0.53	0.24	0.13	0.40	501.2	393.5	644.3
Guinea-Bissau	F	90	0.026	0.013	0.046	0.35	0.19	0.57	0.22	0.12	0.37	515.3	402.4	656.3
Guinea-Bissau	M	25	0.033	0.016	0.057	0.25	0.14	0.42	0.88	0.47	1.40	419.0	323.9	537.2
Guinea-Bissau	M	35	0.027	0.013	0.047	0.23	0.13	0.40	0.65	0.35	1.05	421.7	327.0	537.4
Guinea-Bissau	M	45	0.022	0.011	0.040	0.22	0.12	0.39	0.44	0.24	0.73	425.2	328.2	544.9
Guinea-Bissau	M	55	0.022	0.010	0.038	0.24	0.14	0.41	0.35	0.19	0.58	435.6	337.8	552.9
Guinea-Bissau	M	65	0.022	0.010	0.038	0.27	0.15	0.45	0.29	0.15	0.47	447.4	344.1	570.5
Guinea-Bissau	M	75	0.021	0.010	0.037	0.29	0.17	0.49	0.26	0.14	0.42	459.0	355.8	582.8
Guinea-Bissau	M	90	0.020	0.010	0.036	0.32	0.18	0.53	0.24	0.13	0.39	472.1	366.7	602.9
Equatorial Guinea	F	25	0.134	0.068	0.238	0.67	0.35	1.16	0.86	0.46	1.45	509.8	381.3	671.9
Equatorial Guinea	F	35	0.112	0.058	0.195	0.63	0.33	1.06	0.65	0.36	1.07	513.3	389.3	668.7
Equatorial Guinea	F	45	0.091	0.047	0.166	0.60	0.30	1.02	0.45	0.25	0.76	518.0	392.8	685.0
Equatorial Guinea	F	55	0.087	0.046	0.159	0.65	0.34	1.11	0.35	0.20	0.60	531.1	407.5	689.6
Equatorial Guinea	F	65	0.087	0.044	0.164	0.73	0.40	1.25	0.29	0.16	0.47	546.3	415.6	709.1
Equatorial Guinea	F	75	0.085	0.044	0.158	0.79	0.43	1.34	0.26	0.14	0.42	560.6	429.9	719.9
Equatorial Guinea	F	90	0.082	0.042	0.151	0.85	0.46	1.43	0.24	0.13	0.39	576.7	441.7	743.9
Equatorial Guinea	M	25	0.111	0.054	0.203	0.62	0.32	1.08	0.97	0.49	1.75	472.9	354.4	630.5
Equatorial Guinea	M	35	0.092	0.046	0.169	0.58	0.30	1.00	0.73	0.37	1.30	476.3	357.0	625.6
Equatorial Guinea	M	45	0.075	0.037	0.144	0.55	0.29	0.96	0.49	0.25	0.89	480.7	357.8	634.3
Equatorial Guinea	M	55	0.072	0.036	0.132	0.60	0.32	1.04	0.39	0.20	0.69	492.7	368.3	647.3
Equatorial Guinea	M	65	0.072	0.036	0.131	0.67	0.34	1.17	0.32	0.16	0.58	506.5	377.8	670.3
Equatorial Guinea	M	75	0.071	0.035	0.125	0.73	0.38	1.26	0.29	0.15	0.52	519.5	388.2	687.0
Equatorial Guinea	M	90	0.068	0.034	0.121	0.79	0.42	1.35	0.27	0.14	0.48	534.0	405.9	699.7
Greece	F	25	0.358	0.273	0.458	0.66	0.54	0.79	0.45	0.35	0.57	1013.0	937.5	1088.4
Greece	F	35	0.288	0.221	0.369	0.62	0.52	0.74	0.32	0.25	0.40	1020.4	952.3	1089.4
Greece	F	45	0.224	0.171	0.293	0.59	0.49	0.71	0.20	0.16	0.26	1029.5	950.3	1110.4
Greece	F	55	0.218	0.167	0.278	0.63	0.53	0.75	0.16	0.13	0.21	1052.2	982.7	1124.1
Greece	F	65	0.222	0.168	0.286	0.68	0.57	0.82	0.14	0.11	0.18	1078.0	1002.4	1159.4
Greece	F	75	0.222	0.170	0.285	0.75	0.63	0.89	0.13	0.10	0.16	1105.4	1034.5	1180.4
Greece	F	90	0.220	0.167	0.286	0.84	0.70	0.99	0.12	0.09	0.15	1138.8	1062.8	1213.7

Country	Sex	Midpoint of age range	Mean juice intake (servings/day)	Juice, lower uncertainty interval	Juice, upper uncertainty interval	Mean milk intake (servings/day)	Milk, lower uncertainty interval	Milk, upper uncertainty interval	Mean SSB intake (servings/day)	SSB, lower uncertainty interval	SSB, upper uncertainty interval	Mean calcium intake (servings/day)	Calcium, lower uncertainty interval	Calcium, upper uncertainty interval
Greece	M	25	0.288	0.218	0.370	0.62	0.52	0.75	0.52	0.41	0.66	932.4	860.2	1006.5
Greece	M	35	0.234	0.180	0.301	0.57	0.48	0.67	0.37	0.29	0.47	936.2	871.9	1001.6
Greece	M	45	0.185	0.139	0.242	0.52	0.43	0.62	0.23	0.18	0.29	941.9	871.6	1015.9
Greece	M	55	0.175	0.132	0.225	0.56	0.47	0.66	0.18	0.14	0.23	962.1	900.3	1029.7
Greece	M	65	0.174	0.129	0.224	0.61	0.50	0.73	0.15	0.11	0.19	985.6	918.3	1060.2
Greece	M	75	0.176	0.134	0.226	0.68	0.57	0.80	0.13	0.10	0.17	1010.5	946.8	1079.8
Greece	M	90	0.179	0.137	0.231	0.76	0.64	0.89	0.12	0.10	0.16	1040.4	970.3	1111.1
Grenada	F	25	0.486	0.248	0.872	0.89	0.47	1.49	3.64	2.03	6.20	863.3	660.6	1115.1
Grenada	F	35	0.407	0.211	0.730	0.83	0.44	1.38	2.71	1.52	4.56	868.1	667.2	1114.1
Grenada	F	45	0.334	0.170	0.597	0.79	0.42	1.29	1.83	1.01	3.22	874.8	670.4	1113.6
Grenada	F	55	0.318	0.164	0.571	0.86	0.47	1.41	1.47	0.82	2.51	896.0	693.9	1139.7
Grenada	F	65	0.314	0.160	0.558	0.96	0.52	1.59	1.23	0.70	2.04	920.8	715.8	1172.0
Grenada	F	75	0.307	0.156	0.544	1.04	0.55	1.72	1.12	0.63	1.87	945.1	732.7	1209.7
Grenada	F	90	0.298	0.152	0.540	1.14	0.60	1.86	1.02	0.57	1.72	975.9	752.4	1258.1
Grenada	M	25	0.406	0.198	0.696	0.83	0.45	1.36	4.08	2.29	6.76	792.0	601.6	1005.7
Grenada	M	35	0.339	0.168	0.579	0.77	0.43	1.27	3.03	1.72	5.02	796.6	612.5	1022.2
Grenada	M	45	0.277	0.136	0.489	0.73	0.40	1.22	2.04	1.15	3.41	802.9	614.1	1039.2
Grenada	M	55	0.266	0.132	0.468	0.80	0.44	1.33	1.63	0.93	2.78	822.2	627.8	1059.5
Grenada	M	65	0.266	0.129	0.461	0.89	0.49	1.49	1.36	0.76	2.36	844.6	639.4	1094.7
Grenada	M	75	0.259	0.125	0.446	0.96	0.53	1.60	1.24	0.70	2.16	867.2	659.5	1122.3
Grenada	M	90	0.249	0.120	0.437	1.05	0.57	1.75	1.14	0.64	1.93	894.5	694.4	1158.6
Guatemala	F	25	0.298	0.150	0.525	0.66	0.36	1.09	2.69	1.53	4.61	650.9	492.9	827.5
Guatemala	F	35	0.246	0.125	0.428	0.62	0.34	1.02	2.10	1.21	3.51	655.8	499.1	837.6
Guatemala	F	45	0.200	0.099	0.352	0.59	0.32	1.01	1.53	0.89	2.59	662.0	500.8	845.0
Guatemala	F	55	0.192	0.097	0.333	0.65	0.35	1.07	1.22	0.71	2.07	678.5	514.9	862.8
Guatemala	F	65	0.192	0.097	0.336	0.72	0.39	1.19	0.99	0.56	1.68	697.1	536.6	886.1
Guatemala	F	75	0.188	0.097	0.326	0.78	0.42	1.28	0.88	0.51	1.50	714.6	548.1	911.2
Guatemala	F	90	0.182	0.091	0.316	0.84	0.46	1.40	0.80	0.46	1.35	734.9	559.4	939.0
Guatemala	M	25	0.248	0.129	0.435	0.60	0.33	1.02	2.90	1.64	4.85	597.8	468.1	758.9
Guatemala	M	35	0.206	0.107	0.358	0.57	0.31	0.95	2.26	1.30	3.70	601.2	471.4	754.9
Guatemala	M	45	0.167	0.084	0.300	0.54	0.30	0.91	1.65	0.95	2.82	606.0	470.5	767.9
Guatemala	M	55	0.160	0.082	0.288	0.60	0.33	0.99	1.32	0.77	2.24	621.8	484.2	785.9
Guatemala	M	65	0.161	0.081	0.288	0.66	0.36	1.11	1.07	0.59	1.82	639.8	496.3	808.6
Guatemala	M	75	0.157	0.080	0.279	0.72	0.39	1.20	0.97	0.53	1.62	656.6	509.0	827.6
Guatemala	M	90	0.153	0.079	0.262	0.78	0.43	1.29	0.88	0.48	1.47	675.7	525.3	854.5
Guyana	F	25	0.040	0.018	0.080	0.68	0.38	1.16	3.14	1.75	5.35	815.7	634.6	1054.7
Guyana	F	35	0.034	0.015	0.066	0.64	0.36	1.07	2.34	1.32	3.92	820.2	638.7	1052.0
Guyana	F	45	0.028	0.013	0.053	0.61	0.34	1.01	1.59	0.89	2.74	826.5	642.8	1062.6
Guyana	F	55	0.026	0.012	0.051	0.66	0.37	1.11	1.27	0.71	2.14	846.7	658.7	1083.8
Guyana	F	65	0.026	0.012	0.052	0.74	0.41	1.25	1.06	0.58	1.78	870.1	675.0	1114.0
Guyana	F	75	0.025	0.012	0.050	0.80	0.45	1.35	0.97	0.53	1.62	893.0	697.5	1140.3
Guyana	F	90	0.025	0.011	0.047	0.87	0.49	1.46	0.89	0.50	1.47	919.8	718.7	1172.4
Guyana	M	25	0.033	0.014	0.068	0.63	0.35	1.04	3.41	1.81	5.62	744.4	566.8	950.3
Guyana	M	35	0.027	0.012	0.056	0.59	0.33	0.98	2.52	1.38	4.15	748.5	576.1	955.2
Guyana	M	45	0.022	0.010	0.046	0.56	0.32	0.94	1.70	0.93	2.84	754.2	583.2	966.0
Guyana	M	55	0.021	0.009	0.044	0.61	0.34	1.01	1.36	0.73	2.26	772.4	592.8	984.9
Guyana	M	65	0.021	0.009	0.044	0.68	0.38	1.10	1.14	0.62	1.88	793.5	609.8	1013.3
Guyana	M	75	0.021	0.009	0.043	0.74	0.41	1.19	1.04	0.57	1.70	814.8	626.4	1039.9
Guyana	M	90	0.020	0.009	0.042	0.80	0.45	1.30	0.96	0.53	1.55	839.6	643.2	1063.5
Honduras	F	25	0.851	0.416	1.523	1.14	0.63	1.85	2.98	1.62	4.97	739.8	566.3	930.6
Honduras	F	35	0.704	0.363	1.218	1.07	0.60	1.71	2.32	1.30	3.86	745.2	572.9	940.0
Honduras	F	45	0.570	0.287	0.975	1.03	0.57	1.67	1.70	0.96	2.91	752.2	576.0	953.6
Honduras	F	55	0.549	0.285	0.934	1.12	0.62	1.83	1.36	0.76	2.29	770.9	589.4	977.6
Honduras	F	65	0.550	0.282	0.971	1.25	0.70	2.03	1.09	0.61	1.82	792.1	605.4	1003.7
Honduras	F	75	0.538	0.277	0.923	1.35	0.76	2.17	0.98	0.54	1.61	812.0	624.0	1024.7
Honduras	F	90	0.520	0.266	0.877	1.46	0.82	2.32	0.88	0.49	1.47	835.7	647.5	1051.8
Honduras	M	25	0.704	0.360	1.220	1.02	0.56	1.70	3.22	1.79	5.50	675.4	522.1	849.8
Honduras	M	35	0.582	0.303	0.996	0.96	0.53	1.60	2.51	1.40	4.16	679.2	529.3	863.2
Honduras	M	45	0.471	0.239	0.820	0.92	0.50	1.56	1.83	0.99	3.08	684.5	535.6	876.5
Honduras	M	55	0.453	0.237	0.793	1.01	0.55	1.71	1.47	0.81	2.47	702.3	550.1	899.0
Honduras	M	65	0.454	0.233	0.814	1.13	0.61	1.92	1.19	0.67	2.01	722.6	561.5	929.6
Honduras	M	75	0.445	0.230	0.785	1.22	0.67	2.07	1.07	0.61	1.80	741.6	578.7	946.5
Honduras	M	90	0.433	0.222	0.754	1.33	0.72	2.23	0.97	0.54	1.62	763.5	597.9	972.6
Croatia	F	25	0.330	0.195	0.521	0.78	0.56	1.07	0.65	0.42	0.97	629.7	487.8	800.1
Croatia	F	35	0.274	0.164	0.425	0.74	0.53	1.00	0.48	0.32	0.70	632.7	492.5	802.1
Croatia	F	45	0.223	0.130	0.348	0.71	0.51	0.97	0.33	0.21	0.48	637.2	496.5	808.5
Croatia	F	55	0.210	0.126	0.326	0.79	0.57	1.07	0.26	0.17	0.38	653.3	511.4	828.4
Croatia	F	65	0.205	0.122	0.319	0.89	0.64	1.21	0.22	0.14	0.33	671.9	525.0	854.4
Croatia	F	75	0.201	0.121	0.310	0.97	0.70	1.31	0.20	0.13	0.30	689.9	539.2	877.8
Croatia	F	90	0.196	0.119	0.300	1.06	0.75	1.45	0.19	0.12	0.28	711.0	552.3	903.9
Croatia	M	25	0.259	0.152	0.424	0.72	0.53	0.98	0.75	0.47	1.14	578.5	446.8	748.3
Croatia	M	35	0.214	0.126	0.349	0.67	0.50	0.91	0.55	0.36	0.83	579.3	453.0	739.7
Croatia	M	45	0.173	0.101	0.283	0.64	0.47	0.88	0.37	0.24	0.56	581.6	457.0	743.4
Croatia	M	55	0.165	0.097	0.264	0.72	0.53	0.97	0.29	0.19	0.44	595.6	466.8	761.3
Croatia	M	65	0.164	0.098	0.268	0.81	0.59	1.11	0.24	0.15	0.36	612.1	474.4	788.7
Croatia	M	75	0.163	0.098	0.266	0.89	0.65	1.20	0.22	0.14	0.32	629.5	492.1	809.3
Croatia	M	90	0.161	0.095	0.258	0.96	0.71	1.31	0.20	0.13	0.30	649.4	505.8	830.9
Haiti	F	25	0.223	0.112	0.398	0.34	0.18	0.58	2.64	1.43	4.42	692.1	538.2	869.8
Haiti	F	35	0.186	0.095	0.327	0.32	0.17	0.54	1.97	1.10	3.22	696.0	541.0	876.2
Haiti	F	45	0.153	0.078	0.269	0.30	0.16	0.51	1.34	0.73	2.19	701.4	543.8	891.1
Haiti	F	55	0.146	0.077	0.250	0.33	0.18	0.57	1.07	0.60	1.77	718.6	558.4	911.0
Haiti	F	65	0.144	0.075	0.253	0.37	0.20	0.63	0.89	0.49	1.47	738.5	573.0	935.5
Haiti	F	75	0.141	0.073	0.246	0.40	0.22	0.68	0.81	0.45	1.35	757.8	589.1	957.4
Haiti	F	90	0.137	0.070	0.240	0.43	0.23	0.73	0.75	0.41	1.27	779.7	609.0	989.0
Haiti	M	25	0.186	0.093	0.343	0.31	0.17	0.52	2.90	1.65	4.84	637.0	488.0	816.1
Haiti	M	35	0.155	0.080	0.280	0.29	0.16	0.48	2.15	1.21	3.48	640.6	493.1	818.6
Haiti	M	45	0.126	0.065	0.227	0.28	0.15	0.46	1.45	0.80	2.35	645.5	495.0	820.2
Haiti	M	55	0.121	0.062	0.217	0.30	0.16	0.50	1.16	0.65	1.87	661.2	505.7	844.1
Haiti	M	65	0.121	0.062	0.219	0.34	0.18	0.56	0.96	0.54	1.63	679.2	518.0	864.3
Haiti	M	75	0.118	0.061	0.213	0.36	0.20	0.61	0.88	0.49	1.49	697.2	534.0	889.0
Haiti	M	90	0.114	0.061	0.201	0.39	0.21	0.66	0.81	0.45	1.34	717.6	546.9	918.5
Hungary	F	25	0.100	0.070	0.138	0.25	0.20	0.31	0.48	0.31	0.71	552.7	511.3	596.6
Hungary	F	35	0.083	0.059	0.116	0.24	0.19	0.30	0.36	0.23	0.53	555.2	518.0	593.9
Hungary	F	45	0.068	0.047	0.097	0.23	0.18	0.29	0.24	0.16	0.36	559.2	517.1	604.7
Hungary	F	55	0.064	0.045	0.090	0.25	0.20	0.31	0.20	0.13	0.29	573.5	534.4	614.1
Hungary	F	65	0.062	0.043	0.087	0.29	0.23	0.35	0.16	0.11	0.24	589.8	545.9	634.0
Hungary	F	75	0.061	0.043	0.084	0.31	0.25	0.38	0.15	0.10	0.22	605.5	564.6	647.5
Hungary	F	90	0.060	0.042	0.083	0.34	0.27	0.42	0.14	0.09	0.20	624.5	581.3	670.7
Hungary	M	25	0.079	0.053	0.112	0.23	0.18	0.29	0.57	0.36	0.84	508.1	469.0	551.6

Country	Sex	Midpoint of age range	Mean juice intake (servings/day)	Juice, lower uncertainty interval	Juice, upper uncertainty interval	Mean milk intake (servings/day)	Milk, lower uncertainty interval	Milk, upper uncertainty interval	Mean SSB intake (servings/day)	SSB, lower uncertainty interval	SSB, upper uncertainty interval	Mean calcium intake (servings/day)	Calcium, lower uncertainty interval	Calcium, upper uncertainty interval
Hungary	M	35	0.065	0.045	0.090	0.22	0.18	0.27	0.42	0.27	0.61	508.8	475.9	546.7
Hungary	M	45	0.053	0.036	0.074	0.21	0.17	0.26	0.29	0.19	0.42	510.9	472.0	552.4
Hungary	M	55	0.050	0.035	0.069	0.23	0.19	0.29	0.22	0.15	0.32	523.2	486.7	558.7
Hungary	M	65	0.050	0.034	0.070	0.26	0.21	0.33	0.18	0.12	0.26	537.6	497.0	577.3
Hungary	M	75	0.050	0.034	0.068	0.29	0.23	0.36	0.16	0.11	0.24	552.8	515.6	590.3
Hungary	M	90	0.049	0.034	0.066	0.31	0.25	0.39	0.15	0.10	0.22	571.0	533.9	610.6
Indonesia	F	25	0.037	0.019	0.067	0.04	0.03	0.06	0.45	0.25	0.76	444.7	401.0	491.3
Indonesia	F	35	0.031	0.016	0.055	0.04	0.03	0.05	0.35	0.19	0.57	448.5	409.0	491.1
Indonesia	F	45	0.026	0.013	0.046	0.04	0.03	0.05	0.24	0.14	0.41	453.0	409.9	499.3
Indonesia	F	55	0.025	0.013	0.045	0.05	0.04	0.06	0.19	0.11	0.32	463.2	423.3	508.1
Indonesia	F	65	0.025	0.013	0.046	0.05	0.04	0.07	0.16	0.09	0.27	475.0	430.9	521.6
Indonesia	F	75	0.025	0.013	0.044	0.06	0.04	0.07	0.15	0.08	0.24	488.2	445.7	532.3
Indonesia	F	90	0.024	0.012	0.041	0.06	0.05	0.08	0.13	0.07	0.22	503.7	459.5	549.6
Indonesia	M	25	0.029	0.015	0.054	0.04	0.03	0.05	0.50	0.27	0.85	407.0	368.3	447.2
Indonesia	M	35	0.025	0.013	0.045	0.04	0.03	0.05	0.37	0.21	0.64	410.5	375.0	446.7
Indonesia	M	45	0.021	0.011	0.039	0.04	0.03	0.05	0.26	0.14	0.45	414.6	377.5	456.5
Indonesia	M	55	0.021	0.011	0.039	0.04	0.03	0.05	0.21	0.11	0.36	424.4	388.7	465.0
Indonesia	M	65	0.021	0.011	0.039	0.05	0.04	0.06	0.17	0.10	0.29	435.5	396.2	479.8
Indonesia	M	75	0.021	0.011	0.038	0.05	0.04	0.06	0.16	0.09	0.26	446.8	407.7	490.5
Indonesia	M	90	0.019	0.010	0.035	0.06	0.04	0.07	0.14	0.08	0.24	459.8	421.0	504.4
India	F	25	0.035	0.018	0.061	0.29	0.24	0.34	0.57	0.29	0.97	482.8	443.9	524.6
India	F	35	0.029	0.015	0.051	0.28	0.24	0.32	0.42	0.22	0.72	486.2	452.2	523.3
India	F	45	0.024	0.012	0.042	0.27	0.23	0.32	0.29	0.15	0.50	490.6	452.0	533.7
India	F	55	0.023	0.012	0.040	0.30	0.25	0.35	0.23	0.12	0.40	502.4	467.0	540.3
India	F	65	0.023	0.012	0.041	0.33	0.27	0.39	0.19	0.10	0.33	515.8	475.2	558.4
India	F	75	0.022	0.012	0.040	0.36	0.30	0.42	0.17	0.09	0.29	529.6	491.4	569.5
India	F	90	0.022	0.012	0.037	0.39	0.33	0.46	0.16	0.09	0.27	545.6	507.5	587.2
India	M	25	0.029	0.014	0.054	0.27	0.22	0.32	0.62	0.34	1.05	444.4	405.8	482.4
India	M	35	0.024	0.012	0.045	0.26	0.22	0.30	0.47	0.26	0.77	447.4	415.7	480.2
India	M	45	0.020	0.010	0.038	0.25	0.21	0.30	0.32	0.17	0.54	451.2	417.1	490.3
India	M	55	0.019	0.010	0.035	0.28	0.24	0.32	0.25	0.14	0.42	462.5	429.4	498.4
India	M	65	0.019	0.010	0.034	0.31	0.26	0.36	0.21	0.12	0.34	475.4	435.6	514.6
India	M	75	0.019	0.009	0.034	0.33	0.29	0.38	0.19	0.11	0.31	488.2	451.6	524.2
India	M	90	0.018	0.009	0.033	0.36	0.31	0.41	0.17	0.10	0.29	502.8	466.9	539.6
Ireland	F	25	0.294	0.201	0.415	0.92	0.71	1.17	0.69	0.49	0.93	911.8	846.1	987.9
Ireland	F	35	0.236	0.163	0.333	0.87	0.68	1.08	0.49	0.36	0.67	918.4	860.2	982.8
Ireland	F	45	0.184	0.127	0.263	0.82	0.64	1.06	0.31	0.22	0.42	926.7	859.1	998.6
Ireland	F	55	0.178	0.124	0.249	0.88	0.69	1.11	0.25	0.18	0.34	947.1	880.3	1014.6
Ireland	F	65	0.182	0.124	0.259	0.95	0.74	1.20	0.21	0.15	0.29	970.4	895.7	1049.3
Ireland	F	75	0.182	0.126	0.256	1.05	0.82	1.32	0.20	0.14	0.27	994.9	922.4	1072.1
Ireland	F	90	0.181	0.124	0.256	1.17	0.91	1.49	0.18	0.13	0.24	1026.0	953.0	1105.1
Ireland	M	25	0.238	0.163	0.327	0.88	0.67	1.10	0.81	0.59	1.13	836.4	773.9	907.2
Ireland	M	35	0.193	0.134	0.266	0.80	0.62	1.00	0.57	0.42	0.79	839.8	784.1	902.1
Ireland	M	45	0.153	0.104	0.213	0.74	0.56	0.94	0.35	0.25	0.49	844.9	781.9	912.5
Ireland	M	55	0.145	0.099	0.202	0.78	0.61	0.98	0.27	0.20	0.38	863.1	805.6	925.1
Ireland	M	65	0.144	0.098	0.203	0.86	0.66	1.08	0.23	0.16	0.31	884.1	818.7	952.6
Ireland	M	75	0.145	0.099	0.203	0.95	0.73	1.19	0.21	0.15	0.28	906.4	842.9	972.2
Ireland	M	90	0.148	0.101	0.205	1.07	0.82	1.33	0.19	0.14	0.27	933.2	865.5	1001.0
Iran (Islamic Republic of)	F	25	0.459	0.328	0.636	0.58	0.47	0.71	0.29	0.22	0.37	524.8	448.7	607.2
Iran (Islamic Republic of)	F	35	0.384	0.274	0.523	0.55	0.46	0.67	0.22	0.18	0.28	528.5	455.3	609.8
Iran (Islamic Republic of)	F	45	0.315	0.215	0.439	0.53	0.43	0.65	0.16	0.12	0.20	533.4	453.5	621.8
Iran (Islamic Republic of)	F	55	0.302	0.213	0.413	0.58	0.48	0.70	0.13	0.10	0.16	546.6	467.8	634.9
Iran (Islamic Republic of)	F	65	0.302	0.214	0.422	0.65	0.52	0.79	0.11	0.08	0.13	561.7	482.7	652.6
Iran (Islamic Republic of)	F	75	0.296	0.214	0.409	0.70	0.58	0.84	0.10	0.08	0.12	576.2	496.8	667.5
Iran (Islamic Republic of)	F	90	0.287	0.207	0.391	0.76	0.62	0.91	0.09	0.07	0.11	592.9	508.1	686.2
Iran (Islamic Republic of)	M	25	0.379	0.259	0.533	0.53	0.42	0.66	0.32	0.26	0.40	482.4	412.1	558.2
Iran (Islamic Republic of)	M	35	0.317	0.220	0.436	0.50	0.41	0.61	0.24	0.20	0.30	485.4	417.9	558.3
Iran (Islamic Republic of)	M	45	0.259	0.177	0.367	0.47	0.38	0.58	0.17	0.13	0.21	489.4	419.6	566.2
Iran (Islamic Republic of)	M	55	0.250	0.175	0.346	0.52	0.43	0.63	0.14	0.11	0.17	501.5	428.5	575.0
Iran (Islamic Republic of)	M	65	0.250	0.170	0.346	0.58	0.47	0.72	0.11	0.09	0.14	515.4	436.5	595.3
Iran (Islamic Republic of)	M	75	0.243	0.168	0.337	0.63	0.52	0.78	0.10	0.08	0.13	528.6	451.2	609.7
Iran (Islamic Republic of)	M	90	0.233	0.161	0.326	0.69	0.57	0.84	0.10	0.08	0.12	543.4	466.5	626.1
Iraq	F	25	0.192	0.100	0.348	0.46	0.26	0.77	0.68	0.37	1.15	614.6	470.0	787.0
Iraq	F	35	0.160	0.084	0.290	0.44	0.25	0.74	0.52	0.29	0.86	618.9	474.7	782.6
Iraq	F	45	0.131	0.066	0.237	0.42	0.23	0.73	0.37	0.20	0.62	624.3	479.9	791.4
Iraq	F	55	0.126	0.065	0.231	0.46	0.26	0.79	0.30	0.17	0.51	640.0	496.0	811.5
Iraq	F	65	0.126	0.063	0.230	0.51	0.29	0.89	0.25	0.14	0.44	657.7	507.9	836.6
Iraq	F	75	0.124	0.063	0.227	0.55	0.32	0.96	0.23	0.12	0.39	674.6	518.2	853.4
Iraq	F	90	0.120	0.062	0.224	0.60	0.34	1.05	0.21	0.11	0.35	693.9	532.3	878.3
Iraq	M	25	0.154	0.081	0.272	0.42	0.23	0.68	0.77	0.43	1.29	560.4	432.1	716.4
Iraq	M	35	0.129	0.069	0.226	0.39	0.22	0.64	0.58	0.33	0.99	564.0	435.3	713.7
Iraq	M	45	0.105	0.055	0.186	0.38	0.21	0.62	0.40	0.23	0.68	568.6	437.2	718.4
Iraq	M	55	0.102	0.052	0.179	0.41	0.23	0.67	0.32	0.18	0.55	582.8	450.9	729.7
Iraq	M	65	0.102	0.053	0.180	0.46	0.25	0.76	0.27	0.15	0.46	598.8	460.8	751.4
Iraq	M	75	0.099	0.052	0.174	0.50	0.28	0.82	0.25	0.14	0.42	613.9	473.8	772.4
Iraq	M	90	0.095	0.050	0.166	0.55	0.30	0.90	0.23	0.13	0.39	630.4	489.5	796.4
Iceland	F	25	0.394	0.326	0.473	1.61	1.36	1.88	0.77	0.63	0.93	1069.5	990.3	1150.5
Iceland	F	35	0.316	0.265	0.375	1.52	1.29	1.74	0.55	0.46	0.66	1077.4	1008.1	1149.8
Iceland	F	45	0.247	0.201	0.297	1.44	1.22	1.68	0.35	0.28	0.42	1087.1	1005.7	1167.9
Iceland	F	55	0.239	0.199	0.286	1.54	1.32	1.77	0.28	0.23	0.33	1111.0	1038.6	1183.1
Iceland	F	65	0.244	0.201	0.296	1.67	1.43	1.94	0.24	0.20	0.29	1138.1	1054.9	1221.8
Iceland	F	75	0.244	0.203	0.290	1.83	1.59	2.13	0.22	0.18	0.27	1167.1	1091.1	1243.3
Iceland	F	90	0.242	0.200	0.289	2.05	1.75	2.40	0.20	0.17	0.25	1203.7	1122.2	1284.3
Iceland	M	25	0.316	0.261	0.378	1.54	1.31	1.80	0.90	0.73	1.10	984.9	912.5	1059.7
Iceland	M	35	0.256	0.216	0.303	1.40	1.21	1.62	0.64	0.52	0.77	989.0	928.1	1055.4
Iceland	M	45	0.202	0.168	0.244	1.28	1.08	1.53	0.39	0.32	0.48	995.0	924.3	1067.4
Iceland	M	55	0.192	0.163	0.228	1.37	1.17	1.60	0.30	0.25	0.37	1016.3	953.7	1078.9
Iceland	M	65	0.191	0.157	0.229	1.50	1.28	1.77	0.25	0.20	0.31	1041.0	970.7	1116.5
Iceland	M	75	0.193	0.161	0.227	1.66	1.42	1.92	0.23	0.19	0.28	1067.4	1001.3	1139.8
Iceland	M	90	0.196	0.165	0.232	1.86	1.60	2.18	0.21	0.17	0.26	1099.6	1031.2	1173.9
Israel	F	25	0.122	0.089	0.165	0.57	0.47	0.68	0.77	0.59	0.99	669.8	620.2	724.8
Israel	F	35	0.098	0.073	0.131	0.54	0.46	0.63	0.55	0.43	0.70	674.7	631.0	723.7
Israel	F	45	0.076	0.056	0.103	0.52	0.43	0.61	0.35	0.27	0.45	680.8	631.8	736.9
Israel	F	55	0.074	0.054	0.098	0.55	0.47	0.64	0.28	0.22	0.35	695.8	650.5	747.6
Israel	F	65	0.075	0.055	0.102	0.60	0.49	0.70	0.24	0.19	0.30	712.8	661.6	771.7
Israel	F	75	0.075	0.055	0.100	0.65	0.55	0.76	0.22	0.18	0.28	731.0	680.7	786.5
Israel	F	90	0.075	0.055	0.100	0.73	0.62	0.86	0.20	0.16	0.26	753.8	702.4	812.2
Israel	M	25	0.097	0.071	0.130	0.54	0.45	0.65	0.91	0.71	1.16	616.8	571.2	668.6
Israel	M	35	0.079	0.058	0.105	0.49	0.42	0.58	0.64	0.51	0.81	619.4	578.3	666.3

Country	Sex	Midpoint of age range	Mean juice intake (servings/day)	Juice, lower uncertainty interval	Juice, upper uncertainty interval	Mean milk intake (servings/day)	Milk, lower uncertainty interval	Milk, upper uncertainty interval	Mean SSB intake (servings/day)	SSB, lower uncertainty interval	SSB, upper uncertainty interval	Mean calcium intake (servings/day)	Calcium, lower uncertainty interval	Calcium, upper uncertainty interval
Israel	M	45	0.062	0.045	0.084	0.45	0.38	0.54	0.39	0.30	0.50	623.1	577.6	673.3
Israel	M	55	0.059	0.043	0.079	0.48	0.41	0.57	0.30	0.24	0.38	636.5	595.4	681.5
Israel	M	65	0.059	0.043	0.078	0.53	0.44	0.63	0.25	0.20	0.32	652.0	603.3	702.1
Israel	M	75	0.059	0.044	0.078	0.59	0.49	0.69	0.23	0.18	0.29	668.5	623.7	715.5
Israel	M	90	0.060	0.044	0.080	0.66	0.55	0.78	0.21	0.17	0.27	688.8	643.4	739.0
Italy	F	25	0.146	0.114	0.185	0.53	0.45	0.63	0.21	0.16	0.25	810.1	754.2	870.4
Italy	F	35	0.117	0.093	0.145	0.50	0.44	0.57	0.15	0.12	0.18	816.0	768.4	869.8
Italy	F	45	0.091	0.072	0.116	0.48	0.41	0.55	0.09	0.07	0.12	823.3	767.1	885.6
Italy	F	55	0.089	0.071	0.110	0.51	0.45	0.58	0.07	0.06	0.09	841.4	790.2	895.5
Italy	F	65	0.090	0.071	0.113	0.55	0.48	0.64	0.06	0.05	0.08	862.1	803.1	923.6
Italy	F	75	0.090	0.071	0.113	0.61	0.54	0.69	0.06	0.05	0.07	884.0	830.5	939.4
Italy	F	90	0.090	0.070	0.113	0.68	0.59	0.79	0.05	0.04	0.07	911.7	854.9	969.0
Italy	M	25	0.117	0.090	0.148	0.51	0.43	0.60	0.24	0.19	0.29	745.4	691.4	803.2
Italy	M	35	0.095	0.075	0.120	0.46	0.40	0.53	0.17	0.14	0.21	748.5	702.3	794.7
Italy	M	45	0.075	0.058	0.096	0.43	0.36	0.49	0.10	0.08	0.13	753.0	701.8	804.1
Italy	M	55	0.071	0.056	0.089	0.45	0.39	0.52	0.08	0.06	0.10	769.2	723.5	814.9
Italy	M	65	0.071	0.055	0.090	0.50	0.43	0.58	0.07	0.05	0.08	788.0	735.2	843.3
Italy	M	75	0.072	0.056	0.090	0.55	0.48	0.63	0.06	0.05	0.08	807.8	759.8	857.5
Italy	M	90	0.073	0.056	0.092	0.62	0.54	0.71	0.06	0.05	0.07	832.0	781.9	881.9
Jamaica	F	25	0.463	0.349	0.604	1.14	0.92	1.36	3.29	2.62	4.15	1029.7	942.5	1121.4
Jamaica	F	35	0.387	0.298	0.491	1.07	0.89	1.25	2.45	2.01	2.97	1035.5	961.9	1117.5
Jamaica	F	45	0.318	0.236	0.414	1.01	0.83	1.23	1.66	1.33	2.06	1043.4	964.5	1135.8
Jamaica	F	55	0.303	0.234	0.381	1.11	0.93	1.30	1.33	1.10	1.62	1068.9	994.8	1151.0
Jamaica	F	65	0.299	0.228	0.381	1.23	1.01	1.48	1.11	0.91	1.35	1098.3	1013.1	1188.2
Jamaica	F	75	0.292	0.228	0.371	1.34	1.12	1.57	1.01	0.83	1.22	1127.5	1045.2	1211.8
Jamaica	F	90	0.284	0.224	0.363	1.46	1.23	1.73	0.93	0.77	1.11	1163.1	1076.5	1255.5
Jamaica	M	25	0.380	0.288	0.492	1.05	0.85	1.27	3.58	2.88	4.41	944.0	866.7	1031.7
Jamaica	M	35	0.316	0.248	0.394	0.98	0.83	1.17	2.64	2.20	3.14	949.4	880.2	1021.7
Jamaica	M	45	0.258	0.196	0.326	0.93	0.76	1.12	1.78	1.43	2.17	956.7	881.2	1037.8
Jamaica	M	55	0.248	0.196	0.307	1.02	0.86	1.21	1.43	1.18	1.69	979.9	911.2	1054.4
Jamaica	M	65	0.248	0.189	0.315	1.13	0.94	1.36	1.19	0.97	1.43	1006.6	931.6	1088.3
Jamaica	M	75	0.241	0.189	0.301	1.23	1.04	1.44	1.09	0.90	1.29	1033.5	961.1	1111.2
Jamaica	M	90	0.232	0.185	0.286	1.33	1.12	1.56	1.00	0.84	1.20	1065.9	987.1	1146.1
Jordan	F	25	0.209	0.105	0.369	0.67	0.36	1.10	1.12	0.62	1.89	663.7	512.4	853.3
Jordan	F	35	0.174	0.088	0.307	0.64	0.35	1.03	0.86	0.48	1.46	668.4	516.7	856.2
Jordan	F	45	0.143	0.073	0.257	0.62	0.33	1.00	0.61	0.34	1.05	674.4	518.6	870.9
Jordan	F	55	0.137	0.069	0.247	0.67	0.37	1.09	0.50	0.28	0.84	691.3	535.5	886.6
Jordan	F	65	0.137	0.068	0.246	0.75	0.41	1.22	0.42	0.23	0.69	710.5	550.1	907.0
Jordan	F	75	0.134	0.070	0.236	0.81	0.44	1.31	0.38	0.21	0.63	728.5	567.7	926.0
Jordan	F	90	0.131	0.068	0.231	0.88	0.48	1.43	0.34	0.19	0.57	749.1	576.1	951.9
Jordan	M	25	0.174	0.085	0.315	0.61	0.33	1.08	1.28	0.69	2.18	609.1	474.4	767.3
Jordan	M	35	0.145	0.071	0.261	0.57	0.33	0.98	0.96	0.52	1.59	612.9	475.8	774.2
Jordan	M	45	0.119	0.057	0.222	0.55	0.30	0.92	0.67	0.36	1.08	618.0	478.5	784.5
Jordan	M	55	0.114	0.056	0.213	0.60	0.33	1.02	0.54	0.29	0.88	633.4	492.3	797.2
Jordan	M	65	0.114	0.056	0.214	0.67	0.36	1.16	0.45	0.24	0.73	650.9	506.2	825.2
Jordan	M	75	0.111	0.054	0.205	0.73	0.40	1.25	0.41	0.23	0.68	667.2	524.3	845.6
Jordan	M	90	0.107	0.050	0.198	0.80	0.44	1.35	0.38	0.21	0.64	685.7	542.1	872.5
Japan	F	25	0.206	0.164	0.251	0.51	0.43	0.59	0.50	0.42	0.60	582.3	540.4	625.5
Japan	F	35	0.171	0.144	0.200	0.48	0.42	0.55	0.38	0.32	0.44	587.3	554.1	624.8
Japan	F	45	0.140	0.114	0.169	0.46	0.39	0.55	0.26	0.22	0.31	593.4	554.0	636.0
Japan	F	55	0.135	0.114	0.158	0.51	0.45	0.59	0.20	0.17	0.24	607.9	573.1	643.2
Japan	F	65	0.136	0.112	0.163	0.57	0.49	0.66	0.16	0.14	0.19	624.0	584.7	665.6
Japan	F	75	0.132	0.111	0.157	0.61	0.53	0.69	0.15	0.13	0.18	639.7	603.4	678.2
Japan	F	90	0.127	0.108	0.150	0.65	0.56	0.74	0.15	0.13	0.17	659.4	622.4	697.1
Japan	M	25	0.168	0.136	0.202	0.46	0.39	0.54	0.59	0.49	0.70	531.4	494.5	567.4
Japan	M	35	0.140	0.118	0.163	0.43	0.37	0.49	0.45	0.38	0.51	535.8	504.6	566.4
Japan	M	45	0.115	0.093	0.138	0.40	0.34	0.47	0.32	0.27	0.37	541.1	503.4	576.4
Japan	M	55	0.110	0.093	0.129	0.44	0.39	0.51	0.25	0.21	0.28	554.2	523.2	586.5
Japan	M	65	0.110	0.091	0.131	0.50	0.43	0.59	0.19	0.16	0.23	568.9	532.7	606.8
Japan	M	75	0.108	0.091	0.126	0.55	0.48	0.63	0.17	0.15	0.20	584.8	551.6	619.2
Japan	M	90	0.104	0.089	0.123	0.60	0.52	0.69	0.16	0.14	0.19	604.2	571.1	640.0
Kazakhstan	F	25	0.040	0.019	0.071	0.76	0.54	1.07	0.67	0.35	1.15	717.5	557.2	938.5
Kazakhstan	F	35	0.033	0.016	0.058	0.71	0.51	0.98	0.50	0.27	0.84	721.8	564.4	924.2
Kazakhstan	F	45	0.027	0.013	0.048	0.68	0.47	0.94	0.34	0.18	0.57	727.6	558.7	933.7
Kazakhstan	F	55	0.026	0.013	0.046	0.74	0.53	1.02	0.27	0.15	0.45	745.0	577.8	946.6
Kazakhstan	F	65	0.026	0.013	0.047	0.83	0.58	1.18	0.22	0.12	0.38	765.1	593.0	975.8
Kazakhstan	F	75	0.025	0.012	0.045	0.90	0.64	1.25	0.20	0.11	0.34	785.4	607.0	993.9
Kazakhstan	F	90	0.024	0.012	0.044	0.98	0.70	1.33	0.19	0.10	0.31	810.4	620.4	1030.6
Kazakhstan	M	25	0.033	0.015	0.066	0.69	0.46	0.97	0.74	0.38	1.31	650.9	508.0	823.3
Kazakhstan	M	35	0.027	0.013	0.054	0.65	0.44	0.91	0.55	0.30	0.96	655.1	515.2	817.0
Kazakhstan	M	45	0.022	0.010	0.043	0.62	0.41	0.88	0.38	0.20	0.65	660.6	519.6	825.5
Kazakhstan	M	55	0.021	0.010	0.041	0.68	0.46	0.96	0.30	0.16	0.51	677.0	535.2	844.0
Kazakhstan	M	65	0.021	0.010	0.041	0.76	0.50	1.06	0.25	0.13	0.42	695.9	540.0	875.8
Kazakhstan	M	75	0.021	0.010	0.040	0.82	0.55	1.15	0.22	0.12	0.38	714.2	558.0	898.4
Kazakhstan	M	90	0.020	0.010	0.038	0.89	0.61	1.24	0.21	0.11	0.35	735.2	575.0	922.3
Kenya	F	25	0.151	0.073	0.278	0.62	0.34	1.13	1.12	0.61	1.93	507.0	394.1	654.6
Kenya	F	35	0.125	0.063	0.229	0.58	0.32	1.02	0.84	0.46	1.41	510.0	394.4	659.5
Kenya	F	45	0.102	0.052	0.187	0.55	0.30	0.95	0.57	0.31	0.96	514.2	393.6	659.8
Kenya	F	55	0.098	0.051	0.177	0.61	0.33	1.03	0.45	0.25	0.76	527.3	407.4	681.8
Kenya	F	65	0.098	0.050	0.175	0.68	0.36	1.16	0.37	0.20	0.64	542.2	419.6	705.0
Kenya	F	75	0.096	0.049	0.170	0.74	0.39	1.25	0.34	0.18	0.58	556.4	431.1	719.4
Kenya	F	90	0.093	0.047	0.167	0.79	0.43	1.37	0.31	0.17	0.53	572.3	442.7	737.3
Kenya	M	25	0.125	0.061	0.227	0.55	0.31	0.95	1.23	0.68	2.19	464.7	356.6	604.9
Kenya	M	35	0.103	0.052	0.188	0.52	0.30	0.89	0.92	0.52	1.60	467.9	361.8	604.9
Kenya	M	45	0.084	0.040	0.156	0.49	0.28	0.86	0.63	0.35	1.11	472.0	359.0	612.3
Kenya	M	55	0.080	0.040	0.147	0.54	0.31	0.92	0.50	0.28	0.87	483.6	370.0	624.6
Kenya	M	65	0.080	0.039	0.146	0.60	0.34	1.04	0.41	0.23	0.73	496.8	383.1	636.3
Kenya	M	75	0.078	0.038	0.141	0.65	0.37	1.13	0.37	0.21	0.66	509.7	394.2	657.7
Kenya	M	90	0.076	0.038	0.135	0.70	0.40	1.21	0.34	0.19	0.60	524.3	405.0	679.6
Kyrgyzstan	F	25	0.085	0.042	0.141	0.64	0.43	0.91	0.62	0.33	1.09	706.3	533.2	911.4
Kyrgyzstan	F	35	0.070	0.036	0.116	0.60	0.41	0.83	0.46	0.24	0.80	710.7	535.4	906.8
Kyrgyzstan	F	45	0.057	0.028	0.096	0.57	0.38	0.80	0.31	0.16	0.55	716.4	537.2	916.9
Kyrgyzstan	F	55	0.055	0.028	0.091	0.62	0.43	0.87	0.25	0.13	0.43	733.3	558.0	940.5
Kyrgyzstan	F	65	0.055	0.028	0.092	0.70	0.48	0.98	0.21	0.11	0.35	753.0	570.8	968.4
Kyrgyzstan	F	75	0.054	0.028	0.090	0.75	0.52	1.06	0.19	0.10	0.32	773.1	590.5	984.9
Kyrgyzstan	F	90	0.052	0.027	0.087	0.82	0.56	1.14	0.17	0.09	0.30	797.5	616.1	1017.3
Kyrgyzstan	M	25	0.070	0.035	0.129	0.59	0.39	0.83	0.68	0.37	1.17	641.0	494.6	817.1
Kyrgyzstan	M	35	0.058	0.029	0.106	0.55	0.38	0.77	0.51	0.28	0.86	645.1	499.5	816.2
Kyrgyzstan	M	45	0.047	0.023	0.086	0.52	0.35	0.73	0.35	0.19	0.61	650.6	501.2	822.4

Country	Sex	Midpoint of age range	Mean juice intake (servings/day)	Juice, lower uncertainty interval	Juice, upper uncertainty interval	Mean milk intake (servings/day)	Milk, lower uncertainty interval	Milk, upper uncertainty interval	Mean SSB intake (servings/day)	SSB, lower uncertainty interval	SSB, upper uncertainty interval	Mean calcium intake (servings/day)	Calcium, lower uncertainty interval	Calcium, upper uncertainty interval
Kyrgyzstan	M	55	0.045	0.023	0.081	0.57	0.39	0.79	0.27	0.15	0.48	666.7	512.8	848.7
Kyrgyzstan	M	65	0.045	0.023	0.083	0.64	0.43	0.90	0.22	0.12	0.38	685.5	524.0	880.6
Kyrgyzstan	M	75	0.044	0.022	0.080	0.69	0.48	0.95	0.20	0.11	0.35	703.6	539.0	906.0
Kyrgyzstan	M	90	0.043	0.022	0.079	0.75	0.52	1.04	0.19	0.11	0.32	725.0	558.2	932.0
Cambodia	F	25	0.009	0.004	0.016	0.13	0.07	0.22	0.32	0.17	0.56	473.5	361.1	599.2
Cambodia	F	35	0.007	0.003	0.013	0.13	0.07	0.21	0.25	0.13	0.42	477.5	366.7	602.6
Cambodia	F	45	0.006	0.003	0.011	0.12	0.07	0.21	0.17	0.09	0.29	482.3	370.5	607.2
Cambodia	F	55	0.006	0.003	0.011	0.14	0.07	0.23	0.14	0.07	0.23	493.2	378.8	619.6
Cambodia	F	65	0.006	0.003	0.011	0.15	0.08	0.26	0.11	0.06	0.19	505.7	388.1	639.2
Cambodia	F	75	0.006	0.003	0.011	0.17	0.09	0.28	0.10	0.05	0.17	519.8	398.9	658.1
Cambodia	F	90	0.005	0.003	0.010	0.18	0.09	0.31	0.09	0.05	0.16	536.1	412.1	678.6
Cambodia	M	25	0.007	0.003	0.013	0.12	0.07	0.21	0.36	0.19	0.61	431.9	331.7	545.0
Cambodia	M	35	0.006	0.003	0.011	0.12	0.06	0.19	0.27	0.14	0.45	435.7	335.8	549.0
Cambodia	M	45	0.005	0.002	0.009	0.11	0.06	0.19	0.19	0.10	0.32	440.1	337.9	562.3
Cambodia	M	55	0.005	0.002	0.009	0.12	0.07	0.20	0.15	0.08	0.25	450.5	347.3	569.4
Cambodia	M	65	0.005	0.002	0.009	0.14	0.08	0.23	0.12	0.07	0.21	462.1	354.0	586.4
Cambodia	M	75	0.005	0.002	0.009	0.15	0.08	0.25	0.11	0.06	0.19	474.1	363.5	600.5
Cambodia	M	90	0.004	0.002	0.008	0.17	0.09	0.27	0.10	0.06	0.17	487.9	373.5	621.7
Kiribati	F	25	0.726	0.342	1.343	0.11	0.06	0.18	1.94	0.97	3.40	396.9	307.5	510.3
Kiribati	F	35	0.603	0.289	1.114	0.10	0.06	0.17	1.45	0.74	2.51	399.1	312.1	509.4
Kiribati	F	45	0.492	0.237	0.925	0.10	0.05	0.16	0.98	0.50	1.73	402.1	313.3	515.4
Kiribati	F	55	0.473	0.234	0.865	0.11	0.06	0.18	0.78	0.41	1.36	412.4	320.4	528.2
Kiribati	F	65	0.473	0.235	0.896	0.12	0.06	0.20	0.65	0.34	1.12	424.2	325.3	542.3
Kiribati	F	75	0.463	0.229	0.873	0.13	0.07	0.21	0.59	0.31	1.01	435.3	335.9	556.2
Kiribati	F	90	0.450	0.218	0.860	0.14	0.08	0.23	0.54	0.29	0.91	447.9	350.4	573.0
Kiribati	M	25	0.584	0.276	1.062	0.10	0.06	0.17	2.11	1.08	3.74	362.8	281.8	463.7
Kiribati	M	35	0.482	0.232	0.869	0.10	0.05	0.16	1.58	0.82	2.74	364.8	284.1	470.5
Kiribati	M	45	0.390	0.183	0.700	0.09	0.05	0.15	1.08	0.54	1.90	367.7	285.4	478.2
Kiribati	M	55	0.375	0.179	0.656	0.10	0.06	0.16	0.86	0.43	1.48	377.1	295.0	486.0
Kiribati	M	65	0.375	0.176	0.679	0.11	0.06	0.19	0.71	0.36	1.24	388.0	304.1	492.7
Kiribati	M	75	0.367	0.178	0.658	0.12	0.07	0.20	0.64	0.33	1.11	398.1	311.3	506.5
Kiribati	M	90	0.357	0.175	0.631	0.13	0.07	0.22	0.59	0.31	1.01	409.6	320.3	520.4
Republic of Korea	F	25	0.140	0.113	0.170	0.29	0.23	0.35	0.22	0.19	0.27	565.3	508.9	629.5
Republic of Korea	F	35	0.117	0.098	0.136	0.27	0.23	0.32	0.17	0.14	0.20	570.2	517.1	629.7
Republic of Korea	F	45	0.095	0.077	0.117	0.26	0.21	0.32	0.11	0.09	0.14	576.1	517.7	638.9
Republic of Korea	F	55	0.092	0.078	0.109	0.29	0.24	0.35	0.09	0.08	0.11	590.0	532.3	651.8
Republic of Korea	F	65	0.092	0.076	0.112	0.32	0.26	0.39	0.07	0.06	0.09	605.8	541.6	673.3
Republic of Korea	F	75	0.090	0.075	0.107	0.34	0.29	0.41	0.07	0.06	0.08	621.1	558.9	686.2
Republic of Korea	F	90	0.087	0.073	0.103	0.37	0.30	0.43	0.07	0.05	0.08	639.1	578.5	705.7
Republic of Korea	M	25	0.115	0.093	0.139	0.26	0.21	0.31	0.26	0.22	0.32	515.8	464.3	575.0
Republic of Korea	M	35	0.096	0.080	0.112	0.24	0.20	0.29	0.20	0.17	0.24	520.1	472.7	578.1
Republic of Korea	M	45	0.078	0.064	0.095	0.23	0.19	0.27	0.14	0.12	0.17	525.2	474.4	587.3
Republic of Korea	M	55	0.075	0.063	0.088	0.25	0.21	0.30	0.11	0.09	0.13	537.8	488.6	595.6
Republic of Korea	M	65	0.075	0.062	0.090	0.28	0.23	0.34	0.09	0.07	0.10	552.2	495.9	616.6
Republic of Korea	M	75	0.073	0.062	0.086	0.31	0.26	0.37	0.08	0.07	0.09	567.6	513.5	631.0
Republic of Korea	M	90	0.071	0.061	0.084	0.34	0.28	0.41	0.07	0.06	0.09	585.4	533.3	650.3
Kuwait	F	25	0.155	0.079	0.277	0.65	0.36	1.05	0.78	0.42	1.33	666.7	604.3	734.8
Kuwait	F	35	0.129	0.066	0.229	0.61	0.34	0.98	0.60	0.33	1.03	671.3	613.8	733.7
Kuwait	F	45	0.106	0.052	0.191	0.59	0.33	0.94	0.43	0.23	0.75	677.3	616.4	746.3
Kuwait	F	55	0.102	0.050	0.183	0.65	0.37	1.03	0.35	0.19	0.60	694.1	636.8	757.7
Kuwait	F	65	0.102	0.050	0.185	0.72	0.41	1.17	0.29	0.16	0.50	713.3	651.5	781.5
Kuwait	F	75	0.100	0.050	0.180	0.78	0.44	1.26	0.26	0.14	0.44	731.8	672.8	798.1
Kuwait	F	90	0.097	0.050	0.174	0.84	0.48	1.35	0.24	0.13	0.41	750.2	687.0	817.4
Kuwait	M	25	0.126	0.064	0.229	0.60	0.32	1.01	0.87	0.48	1.45	610.0	552.8	670.0
Kuwait	M	35	0.106	0.053	0.187	0.56	0.31	0.94	0.66	0.37	1.11	613.8	563.6	669.6
Kuwait	M	45	0.087	0.043	0.157	0.54	0.29	0.89	0.45	0.25	0.78	618.7	564.7	681.1
Kuwait	M	55	0.084	0.042	0.150	0.59	0.32	0.96	0.37	0.20	0.63	634.0	579.1	690.8
Kuwait	M	65	0.084	0.042	0.151	0.66	0.35	1.08	0.31	0.17	0.54	651.6	590.7	714.0
Kuwait	M	75	0.081	0.041	0.147	0.72	0.39	1.18	0.28	0.16	0.48	668.4	609.3	729.4
Kuwait	M	90	0.078	0.040	0.141	0.78	0.42	1.29	0.26	0.15	0.45	685.3	625.2	743.8
Lao People's Democratic Republic	F	25	0.010	0.005	0.018	0.12	0.07	0.21	0.26	0.14	0.45	464.2	355.0	590.6
Lao People's Democratic Republic	F	35	0.008	0.004	0.015	0.12	0.07	0.20	0.20	0.11	0.34	468.3	354.5	596.2
Lao People's Democratic Republic	F	45	0.007	0.003	0.012	0.12	0.06	0.19	0.14	0.07	0.24	473.1	356.7	604.5
Lao People's Democratic Republic	F	55	0.006	0.003	0.012	0.13	0.07	0.21	0.11	0.06	0.19	483.8	366.9	615.7
Lao People's Democratic Republic	F	65	0.006	0.003	0.012	0.15	0.08	0.24	0.09	0.05	0.16	496.0	376.6	626.6
Lao People's Democratic Republic	F	75	0.006	0.003	0.012	0.16	0.09	0.26	0.08	0.04	0.14	509.8	391.2	643.8
Lao People's Democratic Republic	F	90	0.006	0.003	0.011	0.17	0.10	0.29	0.08	0.04	0.13	526.1	404.9	675.8
Lao People's Democratic Republic	M	25	0.007	0.004	0.014	0.12	0.06	0.19	0.29	0.16	0.50	430.1	330.2	555.2
Lao People's Democratic Republic	M	35	0.006	0.003	0.012	0.11	0.06	0.18	0.22	0.12	0.37	433.8	331.7	561.9
Lao People's Democratic Republic	M	45	0.005	0.003	0.011	0.10	0.06	0.17	0.15	0.08	0.26	438.3	331.8	569.7
Lao People's Democratic Republic	M	55	0.005	0.003	0.010	0.12	0.06	0.19	0.12	0.07	0.20	448.6	340.5	589.3
Lao People's Democratic Republic	M	65	0.005	0.003	0.010	0.13	0.07	0.22	0.10	0.05	0.17	460.2	351.5	597.6
Lao People's Democratic Republic	M	75	0.005	0.003	0.010	0.14	0.08	0.23	0.09	0.05	0.15	472.2	361.9	614.4
Lao People's Democratic Republic	M	90	0.005	0.002	0.010	0.16	0.08	0.25	0.08	0.04	0.14	485.9	371.6	629.8
Lebanon	F	25	0.275	0.190	0.378	0.30	0.24	0.38	1.27	0.99	1.60	737.7	676.2	804.3
Lebanon	F	35	0.230	0.161	0.310	0.29	0.23	0.35	0.97	0.78	1.20	742.9	682.4	806.2
Lebanon	F	45	0.188	0.134	0.258	0.28	0.22	0.34	0.70	0.55	0.88	749.5	684.2	821.5
Lebanon	F	55	0.181	0.130	0.247	0.31	0.25	0.37	0.57	0.46	0.71	768.2	708.5	833.2
Lebanon	F	65	0.181	0.129	0.252	0.34	0.27	0.42	0.47	0.37	0.60	789.5	725.9	860.9
Lebanon	F	75	0.177	0.127	0.244	0.37	0.29	0.45	0.43	0.34	0.53	809.7	746.6	879.1
Lebanon	F	90	0.172	0.124	0.235	0.40	0.32	0.48	0.39	0.32	0.48	832.9	770.4	907.2
Lebanon	M	25	0.227	0.161	0.316	0.28	0.22	0.34	1.42	1.10	1.81	674.6	617.8	736.9
Lebanon	M	35	0.189	0.137	0.255	0.26	0.21	0.31	1.07	0.85	1.33	678.8	624.2	731.6
Lebanon	M	45	0.155	0.111	0.211	0.25	0.19	0.31	0.74	0.58	0.95	684.5	624.3	745.0
Lebanon	M	55	0.149	0.111	0.201	0.27	0.22	0.33	0.59	0.48	0.75	701.4	645.8	756.6

Country	Sex	Midpoint of age range	Mean juice intake (servings/day)	Juice, lower uncertainty interval	Juice, upper uncertainty interval	Mean milk intake (servings/day)	Milk, lower uncertainty interval	Milk, upper uncertainty interval	Mean SSB intake (servings/day)	SSB, lower uncertainty interval	SSB, upper uncertainty interval	Mean calcium intake (servings/day)	Calcium, lower uncertainty interval	Calcium, upper uncertainty interval
Lebanon	M	65	0.150	0.108	0.205	0.30	0.24	0.37	0.50	0.39	0.62	720.8	659.3	781.0
Lebanon	M	75	0.145	0.107	0.196	0.33	0.27	0.40	0.46	0.37	0.57	739.0	681.5	799.0
Lebanon	M	90	0.140	0.102	0.190	0.36	0.29	0.44	0.43	0.34	0.53	759.4	701.2	820.1
Liberia	F	25	0.018	0.009	0.033	0.11	0.06	0.18	0.45	0.25	0.76	373.0	286.9	478.1
Liberia	F	35	0.015	0.008	0.027	0.10	0.05	0.17	0.34	0.19	0.56	375.2	290.2	473.8
Liberia	F	45	0.012	0.006	0.023	0.10	0.05	0.16	0.23	0.12	0.39	378.2	291.3	476.6
Liberia	F	55	0.012	0.006	0.021	0.11	0.06	0.17	0.18	0.10	0.31	387.7	298.9	488.4
Liberia	F	65	0.012	0.006	0.021	0.12	0.06	0.20	0.15	0.08	0.25	398.7	303.6	509.3
Liberia	F	75	0.012	0.006	0.021	0.13	0.07	0.21	0.14	0.08	0.23	409.0	310.3	518.4
Liberia	F	90	0.011	0.006	0.020	0.14	0.07	0.23	0.13	0.07	0.21	420.4	322.6	534.4
Liberia	M	25	0.015	0.007	0.027	0.10	0.05	0.17	0.52	0.27	0.88	340.9	260.8	442.0
Liberia	M	35	0.013	0.006	0.023	0.09	0.05	0.16	0.39	0.20	0.65	343.1	263.4	443.9
Liberia	M	45	0.010	0.005	0.019	0.09	0.05	0.15	0.26	0.14	0.44	346.0	265.4	452.0
Liberia	M	55	0.010	0.005	0.018	0.10	0.05	0.16	0.21	0.11	0.35	354.4	273.2	459.6
Liberia	M	65	0.010	0.005	0.017	0.11	0.06	0.18	0.17	0.09	0.30	364.1	279.7	471.5
Liberia	M	75	0.010	0.005	0.017	0.12	0.06	0.19	0.16	0.08	0.27	373.5	286.8	480.0
Liberia	M	90	0.009	0.005	0.017	0.13	0.07	0.22	0.14	0.08	0.25	384.1	293.3	493.4
Libyan Arab Jamahiriya	F	25	0.224	0.112	0.393	0.59	0.32	1.00	0.74	0.40	1.28	648.0	495.7	826.3
Libyan Arab Jamahiriya	F	35	0.187	0.093	0.324	0.56	0.31	0.95	0.57	0.31	0.95	652.4	506.3	828.0
Libyan Arab Jamahiriya	F	45	0.153	0.077	0.267	0.54	0.29	0.92	0.40	0.22	0.69	658.2	511.6	836.3
Libyan Arab Jamahiriya	F	55	0.147	0.076	0.251	0.59	0.33	1.01	0.33	0.18	0.56	674.6	521.8	852.2
Libyan Arab Jamahiriya	F	65	0.147	0.075	0.254	0.66	0.36	1.13	0.27	0.15	0.46	693.3	530.6	877.4
Libyan Arab Jamahiriya	F	75	0.144	0.074	0.248	0.71	0.39	1.20	0.25	0.14	0.41	711.0	549.3	897.1
Libyan Arab Jamahiriya	F	90	0.140	0.071	0.238	0.77	0.43	1.29	0.23	0.12	0.37	731.6	568.7	918.9
Libyan Arab Jamahiriya	M	25	0.187	0.093	0.338	0.54	0.31	0.90	0.84	0.47	1.45	594.0	446.1	750.7
Libyan Arab Jamahiriya	M	35	0.156	0.078	0.276	0.51	0.29	0.84	0.63	0.35	1.10	597.7	453.3	756.0
Libyan Arab Jamahiriya	M	45	0.128	0.064	0.232	0.48	0.28	0.81	0.44	0.24	0.77	602.7	457.1	768.4
Libyan Arab Jamahiriya	M	55	0.123	0.063	0.224	0.53	0.31	0.89	0.35	0.20	0.61	617.6	467.5	784.4
Libyan Arab Jamahiriya	M	65	0.123	0.063	0.227	0.59	0.34	1.00	0.30	0.17	0.50	634.6	478.3	799.3
Libyan Arab Jamahiriya	M	75	0.120	0.061	0.219	0.64	0.38	1.09	0.27	0.15	0.46	650.5	491.7	820.4
Libyan Arab Jamahiriya	M	90	0.115	0.059	0.208	0.70	0.41	1.19	0.25	0.14	0.43	668.4	503.7	847.5
Saint Lucia	F	25	0.381	0.195	0.663	0.86	0.46	1.44	3.13	1.72	5.26	862.8	648.5	1106.6
Saint Lucia	F	35	0.319	0.167	0.545	0.80	0.43	1.35	2.33	1.29	3.92	867.6	653.4	1105.5
Saint Lucia	F	45	0.262	0.136	0.456	0.76	0.42	1.28	1.58	0.86	2.65	874.3	661.6	1127.2
Saint Lucia	F	55	0.249	0.130	0.433	0.83	0.45	1.41	1.27	0.70	2.08	895.6	676.2	1147.6
Saint Lucia	F	65	0.246	0.125	0.421	0.93	0.49	1.59	1.06	0.58	1.74	920.4	692.7	1175.2
Saint Lucia	F	75	0.241	0.125	0.413	1.01	0.54	1.70	0.96	0.53	1.58	944.8	711.7	1210.9
Saint Lucia	F	90	0.234	0.121	0.400	1.10	0.59	1.85	0.88	0.50	1.42	974.5	733.4	1248.2
Saint Lucia	M	25	0.312	0.154	0.561	0.81	0.45	1.38	3.45	1.92	5.64	787.0	609.4	994.4
Saint Lucia	M	35	0.260	0.131	0.474	0.76	0.42	1.28	2.55	1.45	4.15	791.6	615.8	990.7
Saint Lucia	M	45	0.213	0.107	0.396	0.72	0.40	1.23	1.72	0.95	2.82	797.8	619.7	1009.9
Saint Lucia	M	55	0.205	0.104	0.377	0.78	0.44	1.32	1.38	0.78	2.23	817.0	635.1	1033.6
Saint Lucia	M	65	0.204	0.101	0.386	0.87	0.49	1.45	1.15	0.63	1.88	839.3	645.5	1073.2
Saint Lucia	M	75	0.199	0.100	0.364	0.94	0.53	1.56	1.05	0.57	1.70	861.7	665.6	1094.5
Saint Lucia	M	90	0.191	0.097	0.342	1.02	0.57	1.69	0.96	0.54	1.56	888.7	692.9	1124.5
Sri Lanka	F	25	0.094	0.045	0.172	1.23	0.94	1.60	0.91	0.63	1.23	624.6	477.4	801.2
Sri Lanka	F	35	0.080	0.038	0.147	1.18	0.93	1.48	0.69	0.51	0.93	630.0	490.6	807.6
Sri Lanka	F	45	0.066	0.031	0.124	1.14	0.88	1.45	0.48	0.35	0.67	636.4	496.5	819.4
Sri Lanka	F	55	0.064	0.031	0.119	1.27	1.00	1.59	0.39	0.29	0.53	650.8	509.0	832.3
Sri Lanka	F	65	0.064	0.032	0.116	1.44	1.10	1.81	0.32	0.23	0.43	667.2	514.8	848.1
Sri Lanka	F	75	0.062	0.031	0.112	1.56	1.22	1.94	0.29	0.21	0.39	685.8	531.1	875.4
Sri Lanka	F	90	0.060	0.030	0.110	1.70	1.32	2.14	0.26	0.19	0.35	708.5	547.3	917.5
Sri Lanka	M	25	0.075	0.037	0.139	1.16	0.89	1.48	0.99	0.70	1.34	574.8	437.6	745.4
Sri Lanka	M	35	0.064	0.033	0.117	1.09	0.85	1.37	0.75	0.54	0.98	579.7	447.3	741.8
Sri Lanka	M	45	0.055	0.028	0.098	1.04	0.80	1.33	0.52	0.37	0.70	585.6	446.9	744.0
Sri Lanka	M	55	0.054	0.028	0.095	1.15	0.91	1.45	0.42	0.31	0.56	599.4	455.1	760.1
Sri Lanka	M	65	0.055	0.028	0.098	1.29	1.02	1.64	0.35	0.25	0.47	615.0	467.6	783.0
Sri Lanka	M	75	0.052	0.026	0.094	1.41	1.12	1.76	0.31	0.23	0.42	631.0	480.8	801.2
Sri Lanka	M	90	0.049	0.025	0.089	1.54	1.22	1.94	0.29	0.21	0.38	650.3	494.9	825.0
Lesotho	F	25	0.010	0.005	0.018	0.38	0.20	0.63	0.76	0.42	1.27	406.0	313.0	518.8
Lesotho	F	35	0.008	0.004	0.014	0.36	0.19	0.59	0.57	0.31	0.94	408.9	315.2	523.5
Lesotho	F	45	0.007	0.003	0.012	0.34	0.19	0.57	0.39	0.21	0.66	412.6	315.4	531.1
Lesotho	F	55	0.006	0.003	0.011	0.38	0.20	0.62	0.31	0.17	0.51	422.1	324.7	537.0
Lesotho	F	65	0.006	0.003	0.011	0.42	0.22	0.69	0.25	0.14	0.43	432.9	333.2	553.6
Lesotho	F	75	0.006	0.003	0.011	0.45	0.24	0.74	0.23	0.13	0.38	444.3	343.7	566.5
Lesotho	F	90	0.006	0.003	0.010	0.49	0.26	0.81	0.21	0.12	0.35	457.5	352.5	582.4
Lesotho	M	25	0.008	0.004	0.015	0.35	0.19	0.58	0.83	0.44	1.43	370.7	284.8	485.4
Lesotho	M	35	0.007	0.003	0.012	0.33	0.18	0.54	0.62	0.34	1.05	373.0	283.8	483.6
Lesotho	M	45	0.006	0.003	0.010	0.31	0.17	0.51	0.42	0.23	0.70	376.1	287.2	483.3
Lesotho	M	55	0.005	0.003	0.010	0.34	0.19	0.55	0.33	0.18	0.55	385.1	292.1	494.0
Lesotho	M	65	0.005	0.003	0.010	0.38	0.21	0.63	0.27	0.15	0.45	395.4	297.2	509.3
Lesotho	M	75	0.005	0.003	0.010	0.41	0.23	0.67	0.25	0.14	0.41	405.9	307.5	522.7
Lesotho	M	90	0.005	0.002	0.009	0.45	0.25	0.73	0.23	0.13	0.38	417.9	320.3	534.5
Lithuania	F	25	0.151	0.071	0.283	0.94	0.73	1.20	0.57	0.31	0.96	816.7	754.8	884.0
Lithuania	F	35	0.124	0.059	0.235	0.90	0.70	1.12	0.42	0.24	0.72	823.5	770.4	880.3
Lithuania	F	45	0.100	0.048	0.182	0.87	0.67	1.10	0.29	0.16	0.50	831.8	769.1	896.3
Lithuania	F	55	0.096	0.046	0.176	0.95	0.75	1.19	0.23	0.13	0.39	850.9	796.6	906.0
Lithuania	F	65	0.097	0.044	0.179	1.06	0.83	1.35	0.19	0.11	0.32	872.6	811.5	937.9
Lithuania	F	75	0.094	0.044	0.172	1.14	0.91	1.44	0.17	0.10	0.29	894.8	837.7	954.1
Lithuania	F	90	0.091	0.044	0.167	1.23	0.97	1.54	0.16	0.09	0.27	921.7	865.7	981.1
Lithuania	M	25	0.125	0.060	0.223	0.86	0.67	1.09	0.60	0.33	0.99	745.6	687.7	809.6
Lithuania	M	35	0.103	0.049	0.182	0.82	0.65	1.01	0.45	0.25	0.73	749.2	697.7	800.0
Lithuania	M	45	0.083	0.039	0.145	0.79	0.61	1.00	0.31	0.17	0.50	754.6	696.7	811.3
Lithuania	M	55	0.080	0.038	0.142	0.88	0.70	1.09	0.24	0.13	0.40	774.6	723.2	824.8
Lithuania	M	65	0.080	0.039	0.142	1.00	0.79	1.26	0.20	0.11	0.33	797.8	740.9	856.7
Lithuania	M	75	0.078	0.038	0.138	1.07	0.84	1.33	0.18	0.10	0.30	819.3	764.5	875.4
Lithuania	M	90	0.076	0.036	0.134	1.14	0.88	1.42	0.17	0.09	0.27	843.7	788.4	900.4
Luxembourg	F	25	0.215	0.126	0.335	0.71	0.43	1.10	0.61	0.39	0.95	937.7	725.2	1214.6
Luxembourg	F	35	0.173	0.102	0.270	0.67	0.41	1.05	0.43	0.27	0.68	944.6	727.9	1213.8
Luxembourg	F	45	0.135	0.079	0.213	0.64	0.39	1.00	0.28	0.17	0.43	953.0	732.1	1221.3
Luxembourg	F	55	0.131	0.076	0.206	0.68	0.42	1.07	0.22	0.14	0.35	974.1	751.0	1245.8
Luxembourg	F	65	0.133	0.076	0.210	0.74	0.46	1.17	0.19	0.12	0.30	998.2	763.3	1285.9
Luxembourg	F	75	0.133	0.078	0.208	0.82	0.50	1.26	0.17	0.11	0.27	1023.4	787.0	1318.2
Luxembourg	F	90	0.132	0.077	0.206	0.91	0.56	1.38	0.16	0.10	0.26	1055.0	816.6	1361.4
Luxembourg	M	25	0.171	0.101	0.268	0.68	0.42	1.05	0.71	0.45	1.11	859.4	665.0	1106.3
Luxembourg	M	35	0.139	0.083	0.217	0.61	0.38	0.93	0.50	0.32	0.77	863.0	668.2	1098.7
Luxembourg	M	45	0.110	0.066	0.170	0.56	0.35	0.85	0.31	0.19	0.47	868.2	667.3	1107.7
Luxembourg	M	55	0.104	0.063	0.160	0.60	0.37	0.93	0.24	0.15	0.37	886.8	685.4	1126.9
Luxembourg	M	65	0.104	0.062	0.159	0.66	0.40	1.03	0.20	0.13	0.31	908.5	704.6	1169.8

Country	Sex	Midpoint of age range	Mean juice intake (servings/day)	Juice, lower uncertainty interval	Juice, upper uncertainty interval	Mean milk intake (servings/day)	Milk, lower uncertainty interval	Milk, upper uncertainty interval	Mean SSB intake (servings/day)	SSB, lower uncertainty interval	SSB, upper uncertainty interval	Mean calcium intake (servings/day)	Calcium, lower uncertainty interval	Calcium, upper uncertainty interval
Luxembourg	M	75	0.104	0.064	0.161	0.73	0.44	1.13	0.18	0.12	0.28	931.4	721.1	1193.3
Luxembourg	M	90	0.106	0.064	0.165	0.82	0.50	1.24	0.17	0.11	0.26	958.8	743.3	1218.4
Latvia	F	25	0.047	0.032	0.066	0.67	0.53	0.83	0.41	0.31	0.51	829.7	768.2	894.0
Latvia	F	35	0.038	0.027	0.054	0.64	0.52	0.78	0.30	0.24	0.36	836.7	782.4	892.2
Latvia	F	45	0.031	0.021	0.044	0.62	0.49	0.77	0.21	0.16	0.26	845.1	782.2	911.3
Latvia	F	55	0.030	0.021	0.041	0.68	0.55	0.84	0.17	0.13	0.20	864.5	809.9	922.2
Latvia	F	65	0.030	0.021	0.042	0.76	0.60	0.95	0.14	0.11	0.17	886.6	828.4	953.2
Latvia	F	75	0.029	0.021	0.040	0.81	0.65	1.01	0.12	0.10	0.15	909.0	852.8	969.1
Latvia	F	90	0.028	0.020	0.039	0.88	0.71	1.08	0.11	0.09	0.14	936.7	876.6	998.6
Latvia	M	25	0.039	0.027	0.056	0.61	0.49	0.76	0.45	0.35	0.56	756.9	698.5	817.3
Latvia	M	35	0.032	0.023	0.045	0.58	0.47	0.71	0.33	0.27	0.41	760.5	710.7	813.0
Latvia	M	45	0.026	0.018	0.037	0.56	0.44	0.69	0.23	0.18	0.28	766.0	711.4	826.3
Latvia	M	55	0.025	0.018	0.035	0.63	0.51	0.77	0.18	0.15	0.22	786.3	738.1	841.5
Latvia	M	65	0.025	0.017	0.035	0.71	0.57	0.89	0.15	0.12	0.18	809.8	751.6	870.5
Latvia	M	75	0.024	0.017	0.034	0.76	0.62	0.94	0.14	0.11	0.16	831.5	777.7	888.6
Latvia	M	90	0.024	0.017	0.033	0.81	0.67	0.99	0.13	0.10	0.15	856.4	804.9	915.3
Morocco	F	25	0.174	0.086	0.319	0.27	0.15	0.45	0.86	0.49	1.44	545.7	416.6	714.0
Morocco	F	35	0.145	0.074	0.270	0.26	0.14	0.42	0.66	0.37	1.11	549.6	415.0	716.8
Morocco	F	45	0.119	0.061	0.225	0.25	0.13	0.41	0.47	0.26	0.81	554.6	415.2	724.4
Morocco	F	55	0.114	0.059	0.211	0.27	0.15	0.45	0.38	0.21	0.65	568.3	432.1	740.7
Morocco	F	65	0.114	0.058	0.212	0.30	0.16	0.50	0.32	0.18	0.55	583.9	442.7	756.4
Morocco	F	75	0.112	0.057	0.208	0.33	0.18	0.54	0.29	0.16	0.50	598.9	454.6	777.6
Morocco	F	90	0.109	0.055	0.198	0.36	0.19	0.59	0.26	0.15	0.45	616.1	467.6	805.2
Morocco	M	25	0.142	0.071	0.256	0.25	0.14	0.43	0.95	0.51	1.58	496.6	380.3	634.8
Morocco	M	35	0.119	0.061	0.210	0.23	0.13	0.39	0.71	0.39	1.20	499.7	388.0	633.4
Morocco	M	45	0.097	0.049	0.175	0.22	0.12	0.37	0.49	0.27	0.82	503.9	389.8	640.4
Morocco	M	55	0.094	0.047	0.168	0.25	0.14	0.41	0.40	0.22	0.67	516.3	401.0	653.8
Morocco	M	65	0.094	0.048	0.168	0.27	0.15	0.46	0.33	0.18	0.57	530.5	406.7	670.3
Morocco	M	75	0.091	0.046	0.163	0.30	0.17	0.50	0.31	0.16	0.52	544.0	419.9	688.2
Morocco	M	90	0.087	0.044	0.156	0.33	0.18	0.55	0.28	0.15	0.48	559.0	433.1	708.4
Moldova	F	25	0.087	0.042	0.157	0.60	0.40	0.87	0.62	0.33	1.02	777.1	597.1	983.1
Moldova	F	35	0.072	0.035	0.129	0.57	0.39	0.83	0.47	0.26	0.75	783.7	604.4	990.7
Moldova	F	45	0.058	0.028	0.104	0.56	0.37	0.82	0.32	0.17	0.53	791.6	610.0	1003.3
Moldova	F	55	0.056	0.028	0.099	0.61	0.41	0.89	0.25	0.14	0.42	809.8	630.8	1021.6
Moldova	F	65	0.056	0.027	0.099	0.68	0.45	0.98	0.21	0.11	0.35	830.3	645.5	1044.4
Moldova	F	75	0.054	0.027	0.095	0.73	0.49	1.05	0.19	0.10	0.31	851.5	661.7	1066.3
Moldova	F	90	0.053	0.026	0.092	0.79	0.53	1.14	0.17	0.09	0.28	876.7	682.2	1093.8
Moldova	M	25	0.072	0.037	0.128	0.55	0.36	0.78	0.67	0.38	1.13	717.3	543.1	925.2
Moldova	M	35	0.059	0.031	0.105	0.52	0.35	0.73	0.50	0.29	0.85	720.6	548.1	923.9
Moldova	M	45	0.048	0.024	0.085	0.50	0.33	0.71	0.34	0.20	0.58	725.9	552.5	934.8
Moldova	M	55	0.046	0.024	0.080	0.56	0.38	0.79	0.27	0.16	0.47	745.0	573.2	956.0
Moldova	M	65	0.046	0.024	0.079	0.64	0.43	0.89	0.23	0.13	0.39	767.0	588.1	977.4
Moldova	M	75	0.045	0.023	0.077	0.68	0.46	0.95	0.20	0.11	0.35	787.7	605.1	1004.0
Moldova	M	90	0.044	0.023	0.076	0.72	0.48	1.02	0.19	0.11	0.31	811.2	626.1	1041.8
Madagascar	F	25	0.020	0.009	0.036	0.29	0.15	0.51	0.44	0.24	0.72	426.5	326.7	551.5
Madagascar	F	35	0.017	0.008	0.030	0.27	0.15	0.47	0.33	0.18	0.54	429.1	329.2	556.4
Madagascar	F	45	0.014	0.007	0.025	0.26	0.14	0.44	0.22	0.12	0.38	432.5	329.9	561.3
Madagascar	F	55	0.013	0.006	0.023	0.28	0.15	0.48	0.18	0.10	0.29	443.5	340.8	575.0
Madagascar	F	65	0.013	0.006	0.023	0.32	0.17	0.53	0.15	0.08	0.24	456.0	350.5	589.3
Madagascar	F	75	0.013	0.006	0.022	0.34	0.19	0.58	0.13	0.07	0.22	467.9	358.5	597.2
Madagascar	F	90	0.012	0.006	0.022	0.37	0.20	0.61	0.12	0.07	0.20	481.2	370.0	608.3
Madagascar	M	25	0.016	0.008	0.030	0.26	0.14	0.44	0.49	0.27	0.82	390.4	301.0	506.0
Madagascar	M	35	0.014	0.007	0.025	0.24	0.13	0.41	0.37	0.21	0.60	392.9	303.4	503.2
Madagascar	M	45	0.011	0.005	0.020	0.23	0.13	0.40	0.25	0.14	0.41	396.3	303.7	507.7
Madagascar	M	55	0.011	0.005	0.019	0.25	0.14	0.43	0.20	0.11	0.33	406.1	313.1	522.1
Madagascar	M	65	0.011	0.005	0.019	0.28	0.15	0.49	0.16	0.09	0.27	417.2	321.4	539.9
Madagascar	M	75	0.010	0.005	0.019	0.30	0.17	0.52	0.15	0.08	0.24	428.1	331.2	553.8
Madagascar	M	90	0.010	0.005	0.018	0.33	0.18	0.54	0.14	0.08	0.22	440.4	340.6	570.7
Maldives	F	25	0.364	0.183	0.670	0.68	0.37	1.20	1.12	0.57	1.95	688.5	511.5	887.5
Maldives	F	35	0.306	0.156	0.552	0.65	0.35	1.13	0.85	0.45	1.44	694.6	518.3	897.8
Maldives	F	45	0.254	0.126	0.456	0.63	0.34	1.08	0.59	0.31	0.99	701.8	522.4	917.6
Maldives	F	55	0.245	0.125	0.436	0.71	0.38	1.23	0.48	0.25	0.78	717.5	533.5	937.0
Maldives	F	65	0.246	0.127	0.436	0.80	0.43	1.38	0.39	0.20	0.67	735.9	547.7	960.3
Maldives	F	75	0.239	0.123	0.430	0.86	0.47	1.48	0.36	0.18	0.60	756.1	563.9	981.8
Maldives	F	90	0.231	0.120	0.423	0.93	0.51	1.62	0.33	0.17	0.55	779.4	583.1	1009.2
Maldives	M	25	0.282	0.146	0.526	0.64	0.34	1.13	1.21	0.63	2.15	626.1	476.1	815.5
Maldives	M	35	0.241	0.126	0.446	0.60	0.32	1.05	0.91	0.48	1.60	631.5	479.2	820.7
Maldives	M	45	0.205	0.104	0.379	0.57	0.31	0.99	0.63	0.34	1.11	638.0	485.0	835.5
Maldives	M	55	0.202	0.104	0.373	0.64	0.34	1.09	0.51	0.27	0.89	652.9	496.2	844.3
Maldives	M	65	0.204	0.102	0.374	0.72	0.39	1.25	0.42	0.23	0.75	670.0	504.8	862.6
Maldives	M	75	0.196	0.101	0.359	0.78	0.43	1.36	0.38	0.21	0.68	687.5	521.7	889.0
Maldives	M	90	0.185	0.097	0.330	0.85	0.46	1.48	0.35	0.19	0.63	707.1	541.8	921.2
Mexico	F	25	0.337	0.161	0.629	1.10	0.92	1.31	2.40	1.71	3.24	961.9	893.9	1038.0
Mexico	F	35	0.278	0.136	0.506	1.04	0.90	1.20	1.87	1.36	2.48	969.0	909.6	1033.6
Mexico	F	45	0.226	0.108	0.401	1.00	0.84	1.18	1.37	0.99	1.85	978.1	907.5	1057.0
Mexico	F	55	0.218	0.102	0.392	1.09	0.94	1.25	1.09	0.80	1.45	1002.4	936.5	1071.7
Mexico	F	65	0.218	0.101	0.403	1.21	1.01	1.42	0.88	0.62	1.19	1029.9	952.4	1108.1
Mexico	F	75	0.213	0.100	0.398	1.31	1.13	1.50	0.79	0.57	1.06	1055.8	985.4	1126.9
Mexico	F	90	0.206	0.097	0.381	1.42	1.22	1.64	0.71	0.52	0.94	1086.6	1016.9	1159.5
Mexico	M	25	0.275	0.130	0.468	0.99	0.83	1.17	2.61	1.83	3.56	880.9	813.8	952.5
Mexico	M	35	0.227	0.113	0.388	0.94	0.81	1.08	2.03	1.46	2.72	885.9	827.2	945.2
Mexico	M	45	0.184	0.091	0.313	0.90	0.76	1.05	1.48	1.06	2.01	892.9	825.3	959.6
Mexico	M	55	0.177	0.089	0.302	0.98	0.85	1.13	1.19	0.86	1.60	916.0	858.8	974.9
Mexico	M	65	0.177	0.087	0.307	1.10	0.92	1.29	0.97	0.69	1.32	942.5	873.3	1011.8
Mexico	M	75	0.174	0.087	0.299	1.19	1.02	1.37	0.87	0.63	1.18	967.3	906.2	1031.4
Mexico	M	90	0.169	0.084	0.286	1.29	1.11	1.48	0.79	0.57	1.07	996.2	933.6	1060.5
Marshall Islands	F	25	0.200	0.100	0.351	0.27	0.15	0.45	1.01	0.56	1.75	484.8	375.3	625.9
Marshall Islands	F	35	0.166	0.084	0.295	0.25	0.14	0.42	0.75	0.43	1.29	487.6	382.5	622.1
Marshall Islands	F	45	0.136	0.068	0.241	0.24	0.13	0.40	0.51	0.28	0.87	491.4	383.1	631.8
Marshall Islands	F	55	0.130	0.067	0.227	0.26	0.15	0.43	0.41	0.23	0.69	503.8	395.0	645.9
Marshall Islands	F	65	0.130	0.066	0.224	0.29	0.16	0.48	0.34	0.19	0.59	518.0	400.5	660.9
Marshall Islands	F	75	0.128	0.064	0.218	0.32	0.18	0.52	0.31	0.17	0.53	531.7	412.9	678.1
Marshall Islands	F	90	0.124	0.061	0.212	0.34	0.20	0.57	0.28	0.16	0.49	547.2	424.6	698.2
Marshall Islands	M	25	0.164	0.079	0.292	0.25	0.13	0.41	1.11	0.60	1.86	445.6	338.8	575.6
Marshall Islands	M	35	0.135	0.067	0.242	0.23	0.13	0.38	0.83	0.45	1.39	448.0	342.8	576.2
Marshall Islands	M	45	0.110	0.053	0.200	0.22	0.12	0.37	0.57	0.30	0.99	451.4	341.1	580.8
Marshall Islands	M	55	0.105	0.052	0.192	0.24	0.13	0.40	0.45	0.25	0.77	463.1	353.9	591.0
Marshall Islands	M	65	0.105	0.053	0.193	0.27	0.15	0.46	0.37	0.20	0.63	476.6	365.9	611.3
Marshall Islands	M	75	0.103	0.053	0.187	0.29	0.16	0.49	0.34	0.19	0.56	489.1	376.7	629.3

Country	Sex	Midpoint of age range	Mean juice intake (servings/day)	Juice, lower uncertainty interval	Juice, upper uncertainty interval	Mean milk intake (servings/day)	Milk, lower uncertainty interval	Milk, upper uncertainty interval	Mean SSB intake (servings/day)	SSB, lower uncertainty interval	SSB, upper uncertainty interval	Mean calcium intake (servings/day)	Calcium, lower uncertainty interval	Calcium, upper uncertainty interval
Marshall Islands	M	90	0.100	0.050	0.179	0.31	0.17	0.53	0.31	0.17	0.53	503.2	387.7	647.7
Macedonia (Former Yugoslav Republic of)	F	25	0.508	0.252	0.901	0.53	0.28	0.88	0.55	0.31	0.96	578.6	448.0	742.6
Macedonia (Former Yugoslav Republic of)	F	35	0.421	0.209	0.748	0.50	0.26	0.82	0.41	0.23	0.70	581.2	453.6	747.8
Macedonia (Former Yugoslav Republic of)	F	45	0.342	0.164	0.602	0.48	0.25	0.82	0.28	0.16	0.47	585.3	453.1	754.3
Macedonia (Former Yugoslav Republic of)	F	55	0.322	0.157	0.565	0.53	0.29	0.91	0.22	0.13	0.38	600.1	465.7	768.2
Macedonia (Former Yugoslav Republic of)	F	65	0.315	0.154	0.564	0.60	0.33	1.03	0.19	0.11	0.31	617.3	481.1	786.9
Macedonia (Former Yugoslav Republic of)	F	75	0.309	0.151	0.551	0.65	0.36	1.12	0.17	0.10	0.29	633.7	494.9	808.5
Macedonia (Former Yugoslav Republic of)	F	90	0.302	0.145	0.543	0.71	0.38	1.20	0.16	0.09	0.26	652.8	510.8	835.2
Macedonia (Former Yugoslav Republic of)	M	25	0.411	0.197	0.787	0.48	0.26	0.82	0.66	0.37	1.08	528.7	406.7	678.4
Macedonia (Former Yugoslav Republic of)	M	35	0.340	0.162	0.645	0.45	0.25	0.77	0.49	0.28	0.79	529.5	409.2	677.6
Macedonia (Former Yugoslav Republic of)	M	45	0.275	0.124	0.522	0.43	0.23	0.74	0.33	0.19	0.54	531.7	405.5	678.7
Macedonia (Former Yugoslav Republic of)	M	55	0.262	0.120	0.499	0.48	0.26	0.81	0.26	0.15	0.43	544.5	419.7	694.0
Macedonia (Former Yugoslav Republic of)	M	65	0.260	0.121	0.494	0.54	0.29	0.89	0.21	0.12	0.35	559.6	427.5	715.2
Macedonia (Former Yugoslav Republic of)	M	75	0.258	0.121	0.487	0.59	0.32	0.98	0.19	0.11	0.32	575.4	440.8	737.6
Macedonia (Former Yugoslav Republic of)	M	90	0.255	0.123	0.475	0.64	0.35	1.09	0.18	0.10	0.30	593.4	457.7	762.7
Mali	F	25	0.013	0.006	0.024	0.58	0.35	0.88	0.79	0.42	1.31	515.9	422.7	618.3
Mali	F	35	0.011	0.005	0.020	0.55	0.33	0.82	0.59	0.32	0.98	518.8	429.6	617.1
Mali	F	45	0.009	0.004	0.016	0.52	0.31	0.81	0.40	0.22	0.68	522.9	431.0	620.1
Mali	F	55	0.008	0.004	0.015	0.58	0.35	0.89	0.32	0.18	0.54	536.2	443.9	634.1
Mali	F	65	0.008	0.004	0.015	0.65	0.40	1.01	0.26	0.14	0.44	551.4	457.3	651.6
Mali	F	75	0.008	0.004	0.015	0.70	0.43	1.07	0.24	0.13	0.40	565.5	471.0	670.1
Mali	F	90	0.008	0.004	0.014	0.75	0.47	1.15	0.22	0.13	0.36	580.9	483.7	691.5
Mali	M	25	0.010	0.005	0.018	0.55	0.33	0.85	0.90	0.47	1.50	477.1	392.6	570.3
Mali	M	35	0.008	0.004	0.015	0.51	0.32	0.77	0.67	0.36	1.11	480.1	398.9	571.4
Mali	M	45	0.007	0.003	0.012	0.49	0.30	0.74	0.45	0.24	0.77	484.1	400.2	574.9
Mali	M	55	0.007	0.003	0.012	0.53	0.33	0.82	0.36	0.19	0.61	495.9	412.7	586.2
Mali	M	65	0.007	0.003	0.012	0.60	0.36	0.92	0.30	0.16	0.49	509.4	421.6	605.4
Mali	M	75	0.007	0.003	0.011	0.65	0.40	0.99	0.27	0.14	0.45	522.6	435.1	621.7
Mali	M	90	0.006	0.003	0.011	0.70	0.43	1.06	0.25	0.13	0.41	537.2	447.9	638.8
Malta	F	25	0.611	0.494	0.756	1.05	0.78	1.39	1.36	0.93	1.91	894.3	676.5	1159.2
Malta	F	35	0.491	0.399	0.599	0.99	0.74	1.30	0.97	0.67	1.35	900.7	688.3	1150.4
Malta	F	45	0.382	0.305	0.470	0.94	0.69	1.25	0.61	0.42	0.87	908.8	696.3	1152.0
Malta	F	55	0.371	0.301	0.454	1.00	0.74	1.32	0.49	0.34	0.69	929.0	706.2	1179.5
Malta	F	65	0.378	0.304	0.465	1.09	0.81	1.44	0.42	0.29	0.59	951.8	716.7	1212.1
Malta	F	75	0.378	0.306	0.463	1.19	0.90	1.57	0.39	0.27	0.54	975.9	735.4	1244.3
Malta	F	90	0.375	0.302	0.468	1.33	0.99	1.77	0.36	0.25	0.50	1005.5	762.6	1278.2
Malta	M	25	0.490	0.394	0.600	1.00	0.74	1.34	1.61	1.12	2.24	823.2	635.3	1069.9
Malta	M	35	0.398	0.322	0.484	0.91	0.69	1.20	1.13	0.80	1.59	826.5	643.9	1065.9
Malta	M	45	0.313	0.246	0.390	0.84	0.62	1.11	0.69	0.48	0.97	831.6	646.0	1065.6
Malta	M	55	0.297	0.239	0.366	0.89	0.67	1.16	0.54	0.38	0.75	849.5	657.4	1086.4
Malta	M	65	0.296	0.236	0.370	0.97	0.73	1.28	0.45	0.31	0.64	870.2	668.8	1121.3
Malta	M	75	0.299	0.240	0.368	1.08	0.82	1.40	0.41	0.28	0.58	892.2	685.2	1149.9
Malta	M	90	0.304	0.243	0.376	1.21	0.91	1.60	0.38	0.26	0.54	918.7	704.7	1184.9
Myanmar	F	25	0.027	0.014	0.047	0.30	0.17	0.52	0.47	0.26	0.79	571.5	451.3	719.5
Myanmar	F	35	0.022	0.012	0.040	0.29	0.16	0.48	0.36	0.20	0.60	576.4	454.6	728.9
Myanmar	F	45	0.019	0.009	0.033	0.28	0.16	0.46	0.25	0.14	0.44	582.2	457.5	739.9
Myanmar	F	55	0.018	0.009	0.032	0.31	0.17	0.51	0.20	0.11	0.34	595.5	468.5	749.0
Myanmar	F	65	0.018	0.009	0.032	0.35	0.19	0.57	0.17	0.09	0.28	610.6	477.8	762.4
Myanmar	F	75	0.018	0.009	0.032	0.38	0.21	0.63	0.15	0.09	0.25	627.6	493.2	783.9
Myanmar	F	90	0.017	0.009	0.031	0.42	0.23	0.68	0.14	0.08	0.22	647.6	511.5	810.7
Myanmar	M	25	0.021	0.011	0.038	0.29	0.16	0.48	0.52	0.28	0.91	525.2	410.5	685.0
Myanmar	M	35	0.018	0.009	0.032	0.27	0.15	0.45	0.39	0.22	0.67	529.8	412.9	689.5
Myanmar	M	45	0.015	0.008	0.027	0.26	0.14	0.43	0.27	0.14	0.47	535.2	413.3	694.3
Myanmar	M	55	0.015	0.008	0.027	0.29	0.16	0.48	0.22	0.12	0.37	547.7	426.8	709.4
Myanmar	M	65	0.015	0.008	0.028	0.32	0.18	0.54	0.18	0.10	0.31	561.9	438.7	728.5
Myanmar	M	75	0.015	0.007	0.027	0.35	0.19	0.59	0.17	0.09	0.28	576.6	451.4	748.3
Myanmar	M	90	0.014	0.007	0.025	0.38	0.21	0.65	0.15	0.08	0.26	593.5	465.1	767.8
Montenegro	F	25	0.203	0.119	0.327	1.08	0.76	1.51	0.42	0.26	0.65	695.3	539.5	880.8
Montenegro	F	35	0.169	0.100	0.272	1.02	0.72	1.41	0.31	0.20	0.48	698.7	544.9	893.2
Montenegro	F	45	0.137	0.080	0.229	0.98	0.68	1.37	0.21	0.13	0.34	703.7	544.3	902.1
Montenegro	F	55	0.129	0.076	0.212	1.09	0.78	1.52	0.17	0.10	0.27	721.6	560.3	913.6
Montenegro	F	65	0.126	0.074	0.207	1.23	0.89	1.71	0.14	0.09	0.23	742.1	571.5	943.3
Montenegro	F	75	0.124	0.073	0.200	1.34	0.97	1.85	0.13	0.08	0.20	761.9	587.9	965.5
Montenegro	F	90	0.121	0.072	0.192	1.46	1.04	2.00	0.12	0.08	0.19	784.8	612.6	996.3
Montenegro	M	25	0.161	0.097	0.259	1.00	0.70	1.42	0.49	0.29	0.75	635.1	489.0	819.8
Montenegro	M	35	0.133	0.080	0.213	0.94	0.65	1.32	0.37	0.22	0.56	635.9	490.3	814.2
Montenegro	M	45	0.107	0.063	0.175	0.90	0.62	1.28	0.25	0.15	0.38	638.5	492.1	816.5
Montenegro	M	55	0.102	0.062	0.163	1.00	0.70	1.41	0.19	0.12	0.30	653.7	504.4	835.3
Montenegro	M	65	0.102	0.060	0.160	1.13	0.79	1.59	0.16	0.10	0.24	671.8	520.6	857.7
Montenegro	M	75	0.101	0.061	0.158	1.23	0.86	1.74	0.14	0.09	0.22	690.9	534.3	880.5
Montenegro	M	90	0.100	0.060	0.157	1.34	0.93	1.89	0.13	0.08	0.20	712.9	544.9	917.5
Mongolia	F	25	0.010	0.005	0.019	0.59	0.33	1.02	0.36	0.20	0.59	687.1	525.9	884.4
Mongolia	F	35	0.008	0.004	0.015	0.55	0.31	0.94	0.27	0.15	0.44	691.3	531.8	877.5
Mongolia	F	45	0.007	0.003	0.013	0.52	0.29	0.88	0.18	0.10	0.30	696.8	529.6	895.4
Mongolia	F	55	0.007	0.003	0.012	0.57	0.32	0.95	0.14	0.08	0.24	713.4	544.7	907.9
Mongolia	F	65	0.007	0.003	0.012	0.64	0.35	1.07	0.12	0.06	0.20	732.7	559.1	933.3
Mongolia	F	75	0.006	0.003	0.012	0.69	0.39	1.16	0.11	0.06	0.18	752.1	572.9	958.2
Mongolia	F	90	0.006	0.003	0.012	0.75	0.42	1.27	0.10	0.06	0.16	774.9	591.7	979.3
Mongolia	M	25	0.009	0.004	0.016	0.53	0.29	0.87	0.40	0.23	0.67	624.4	475.6	788.3
Mongolia	M	35	0.007	0.003	0.013	0.49	0.27	0.81	0.30	0.17	0.50	628.3	482.3	782.5
Mongolia	M	45	0.006	0.003	0.011	0.47	0.26	0.79	0.20	0.11	0.35	633.5	487.6	795.0
Mongolia	M	55	0.005	0.003	0.010	0.51	0.28	0.87	0.16	0.09	0.27	649.3	500.5	808.7
Mongolia	M	65	0.005	0.003	0.010	0.57	0.31	0.97	0.13	0.07	0.23	667.6	514.6	844.3
Mongolia	M	75	0.005	0.003	0.010	0.62	0.34	1.02	0.12	0.07	0.20	685.1	527.1	861.4
Mongolia	M	90	0.005	0.003	0.010	0.67	0.37	1.10	0.11	0.06	0.19	705.1	543.4	886.6

Country	Sex	Midpoint of age range	Mean juice intake (servings/day)	Juice, lower uncertainty interval	Juice, upper uncertainty interval	Mean milk intake (servings/day)	Milk, lower uncertainty interval	Milk, upper uncertainty interval	Mean SSB intake (servings/day)	SSB, lower uncertainty interval	SSB, upper uncertainty interval	Mean calcium intake (servings/day)	Calcium, lower uncertainty interval	Calcium, upper uncertainty interval
Mozambique	F	25	0.004	0.002	0.008	0.08	0.06	0.10	0.48	0.26	0.80	319.7	280.2	360.8
Mozambique	F	35	0.004	0.002	0.007	0.07	0.06	0.09	0.36	0.19	0.60	321.5	285.9	360.1
Mozambique	F	45	0.003	0.001	0.005	0.07	0.06	0.09	0.25	0.13	0.42	324.1	286.6	363.3
Mozambique	F	55	0.003	0.001	0.005	0.08	0.06	0.09	0.19	0.10	0.32	332.3	297.6	369.0
Mozambique	F	65	0.003	0.001	0.005	0.09	0.07	0.11	0.16	0.08	0.27	341.8	302.3	386.2
Mozambique	F	75	0.003	0.001	0.005	0.09	0.08	0.11	0.14	0.08	0.25	350.6	313.4	393.2
Mozambique	F	90	0.003	0.001	0.005	0.10	0.08	0.12	0.13	0.07	0.23	360.6	322.5	402.2
Mozambique	M	25	0.004	0.002	0.007	0.07	0.06	0.09	0.54	0.30	0.91	293.1	259.3	331.1
Mozambique	M	35	0.003	0.001	0.006	0.07	0.05	0.08	0.40	0.23	0.67	295.0	263.5	328.5
Mozambique	M	45	0.002	0.001	0.004	0.06	0.05	0.08	0.27	0.15	0.47	297.6	262.9	335.4
Mozambique	M	55	0.002	0.001	0.004	0.07	0.06	0.08	0.22	0.12	0.37	304.9	273.9	340.9
Mozambique	M	65	0.002	0.001	0.004	0.08	0.06	0.10	0.18	0.10	0.31	313.3	279.1	353.1
Mozambique	M	75	0.002	0.001	0.004	0.08	0.07	0.10	0.16	0.09	0.28	321.4	288.6	358.9
Mozambique	M	90	0.002	0.001	0.004	0.09	0.07	0.11	0.15	0.08	0.25	330.5	295.5	367.6
Mauritania	F	25	0.033	0.016	0.063	1.07	0.55	1.90	1.63	0.82	2.86	623.7	447.5	821.6
Mauritania	F	35	0.028	0.013	0.051	1.01	0.52	1.79	1.22	0.62	2.12	627.2	450.1	822.4
Mauritania	F	45	0.023	0.011	0.044	0.96	0.49	1.71	0.83	0.40	1.45	632.2	456.5	829.4
Mauritania	F	55	0.022	0.010	0.041	1.06	0.54	1.87	0.66	0.33	1.15	648.1	468.9	844.5
Mauritania	F	65	0.022	0.011	0.041	1.19	0.60	2.10	0.54	0.28	0.98	666.4	477.3	869.2
Mauritania	F	75	0.021	0.010	0.040	1.28	0.65	2.24	0.49	0.25	0.89	683.5	491.7	893.9
Mauritania	F	90	0.020	0.010	0.038	1.38	0.71	2.39	0.46	0.23	0.80	702.0	508.8	915.2
Mauritania	M	25	0.027	0.012	0.052	1.02	0.52	1.78	1.77	0.93	3.18	576.6	426.6	774.4
Mauritania	M	35	0.022	0.010	0.043	0.95	0.49	1.65	1.32	0.71	2.37	580.2	428.7	770.5
Mauritania	M	45	0.018	0.008	0.035	0.90	0.46	1.55	0.90	0.47	1.61	585.0	430.2	776.1
Mauritania	M	55	0.018	0.008	0.033	0.99	0.52	1.70	0.71	0.37	1.27	599.2	443.3	790.0
Mauritania	M	65	0.018	0.008	0.033	1.11	0.59	1.93	0.59	0.31	1.03	615.5	456.3	812.0
Mauritania	M	75	0.017	0.008	0.033	1.20	0.63	2.09	0.53	0.28	0.94	631.5	468.0	835.0
Mauritania	M	90	0.017	0.008	0.032	1.29	0.68	2.22	0.49	0.26	0.89	648.8	487.8	853.5
Mauritius	F	25	0.056	0.028	0.098	0.80	0.41	1.41	0.97	0.51	1.64	717.0	526.3	954.7
Mauritius	F	35	0.047	0.023	0.083	0.76	0.41	1.34	0.73	0.39	1.26	723.2	528.6	957.0
Mauritius	F	45	0.039	0.019	0.070	0.74	0.39	1.31	0.52	0.27	0.90	730.6	533.7	961.2
Mauritius	F	55	0.038	0.019	0.066	0.83	0.44	1.46	0.41	0.22	0.71	747.0	547.3	982.1
Mauritius	F	65	0.038	0.019	0.065	0.93	0.49	1.65	0.34	0.18	0.59	765.8	562.6	1018.7
Mauritius	F	75	0.037	0.019	0.064	1.01	0.54	1.77	0.31	0.17	0.53	787.1	582.8	1040.5
Mauritius	F	90	0.035	0.018	0.063	1.10	0.58	1.93	0.28	0.15	0.48	813.7	601.6	1062.7
Mauritius	M	25	0.044	0.023	0.081	0.75	0.39	1.33	1.07	0.58	1.78	649.9	484.4	871.0
Mauritius	M	35	0.038	0.019	0.068	0.71	0.37	1.24	0.81	0.45	1.31	655.4	490.5	862.0
Mauritius	M	45	0.032	0.016	0.056	0.67	0.35	1.18	0.56	0.31	0.93	661.9	496.5	872.3
Mauritius	M	55	0.031	0.016	0.057	0.75	0.40	1.30	0.45	0.25	0.73	677.5	509.3	882.3
Mauritius	M	65	0.032	0.016	0.059	0.84	0.44	1.49	0.37	0.20	0.62	695.0	525.3	907.4
Mauritius	M	75	0.031	0.015	0.056	0.92	0.49	1.60	0.34	0.19	0.55	713.3	541.9	930.5
Mauritius	M	90	0.029	0.014	0.053	1.00	0.53	1.77	0.31	0.18	0.51	735.2	553.3	968.8
Malawi	F	25	0.081	0.039	0.147	0.07	0.04	0.12	0.68	0.37	1.17	315.9	237.7	408.6
Malawi	F	35	0.067	0.033	0.124	0.07	0.04	0.11	0.51	0.28	0.87	317.7	238.8	411.5
Malawi	F	45	0.055	0.027	0.101	0.06	0.03	0.11	0.35	0.19	0.60	320.3	240.2	415.0
Malawi	F	55	0.053	0.026	0.096	0.07	0.04	0.12	0.28	0.15	0.47	328.5	247.2	426.7
Malawi	F	65	0.053	0.026	0.096	0.08	0.04	0.13	0.23	0.12	0.38	337.9	254.5	440.3
Malawi	F	75	0.051	0.026	0.093	0.09	0.05	0.14	0.21	0.11	0.34	346.6	261.5	451.6
Malawi	F	90	0.050	0.025	0.090	0.09	0.05	0.15	0.19	0.11	0.32	356.4	270.1	464.2
Malawi	M	25	0.067	0.033	0.124	0.07	0.03	0.11	0.75	0.40	1.33	288.4	210.6	380.8
Malawi	M	35	0.055	0.028	0.100	0.06	0.03	0.10	0.56	0.31	0.97	290.3	213.0	381.3
Malawi	M	45	0.045	0.021	0.083	0.06	0.03	0.10	0.38	0.20	0.67	292.8	213.9	386.9
Malawi	M	55	0.043	0.021	0.078	0.06	0.03	0.11	0.30	0.16	0.53	300.1	220.5	396.5
Malawi	M	65	0.043	0.020	0.079	0.07	0.04	0.12	0.25	0.13	0.43	308.3	226.4	409.1
Malawi	M	75	0.042	0.020	0.077	0.08	0.04	0.13	0.23	0.12	0.39	316.2	233.7	416.7
Malawi	M	90	0.041	0.020	0.075	0.08	0.04	0.14	0.21	0.11	0.36	325.0	241.0	426.2
Malaysia	F	25	0.308	0.232	0.402	0.16	0.12	0.21	1.08	0.58	1.91	509.1	445.5	578.8
Malaysia	F	35	0.259	0.201	0.330	0.15	0.12	0.19	0.82	0.44	1.43	513.5	454.7	579.8
Malaysia	F	45	0.215	0.164	0.281	0.15	0.11	0.19	0.58	0.31	1.02	518.7	455.8	590.3
Malaysia	F	55	0.208	0.163	0.266	0.17	0.13	0.21	0.47	0.25	0.81	530.5	468.0	600.4
Malaysia	F	65	0.208	0.161	0.271	0.19	0.14	0.24	0.38	0.20	0.67	543.9	477.1	620.6
Malaysia	F	75	0.203	0.160	0.261	0.20	0.16	0.26	0.35	0.19	0.59	559.0	492.2	634.0
Malaysia	F	90	0.195	0.154	0.249	0.22	0.17	0.28	0.32	0.17	0.53	576.6	509.7	653.4
Malaysia	M	25	0.239	0.180	0.319	0.15	0.12	0.19	1.23	0.63	2.15	466.8	409.0	529.5
Malaysia	M	35	0.204	0.158	0.265	0.14	0.11	0.18	0.92	0.48	1.58	470.9	415.2	533.0
Malaysia	M	45	0.173	0.130	0.224	0.14	0.10	0.17	0.64	0.33	1.11	475.8	415.7	545.2
Malaysia	M	55	0.171	0.131	0.219	0.15	0.12	0.19	0.52	0.27	0.86	486.9	427.6	552.2
Malaysia	M	65	0.173	0.133	0.230	0.17	0.13	0.22	0.43	0.22	0.72	499.4	435.6	569.2
Malaysia	M	75	0.167	0.129	0.219	0.18	0.14	0.23	0.39	0.20	0.65	512.5	449.7	580.3
Malaysia	M	90	0.157	0.122	0.202	0.20	0.16	0.25	0.36	0.19	0.60	527.5	461.3	594.9
Namibia	F	25	0.026	0.013	0.047	0.94	0.50	1.69	1.15	0.65	1.99	498.4	386.9	632.7
Namibia	F	35	0.021	0.011	0.038	0.88	0.47	1.57	0.86	0.50	1.46	502.0	393.9	631.9
Namibia	F	45	0.017	0.008	0.031	0.84	0.45	1.49	0.59	0.33	1.00	506.5	397.1	644.5
Namibia	F	55	0.017	0.008	0.030	0.92	0.50	1.62	0.47	0.27	0.79	518.1	408.6	660.3
Namibia	F	65	0.017	0.008	0.031	1.02	0.56	1.81	0.38	0.22	0.65	531.4	416.7	678.0
Namibia	F	75	0.016	0.008	0.030	1.10	0.60	1.97	0.35	0.20	0.59	545.3	428.5	695.3
Namibia	F	90	0.016	0.008	0.029	1.20	0.64	2.12	0.32	0.18	0.55	561.8	442.1	714.6
Namibia	M	25	0.021	0.010	0.036	0.84	0.46	1.41	1.29	0.69	2.16	457.2	347.2	589.5
Namibia	M	35	0.017	0.009	0.030	0.79	0.43	1.30	0.96	0.53	1.60	460.0	351.4	590.4
Namibia	M	45	0.014	0.007	0.024	0.75	0.41	1.27	0.65	0.36	1.11	463.7	356.7	598.8
Namibia	M	55	0.013	0.007	0.023	0.82	0.45	1.36	0.52	0.29	0.86	474.7	365.4	608.3
Namibia	M	65	0.013	0.007	0.023	0.92	0.50	1.49	0.43	0.23	0.71	487.3	374.2	628.6
Namibia	M	75	0.013	0.007	0.023	0.99	0.55	1.64	0.39	0.21	0.63	500.3	385.1	644.0
Namibia	M	90	0.013	0.006	0.022	1.08	0.59	1.78	0.36	0.20	0.58	515.3	397.5	660.4
Niger	F	25	0.002	0.001	0.005	0.46	0.28	0.73	0.45	0.24	0.77	505.7	432.9	589.0
Niger	F	35	0.002	0.001	0.004	0.43	0.27	0.67	0.33	0.18	0.56	508.6	439.0	588.8
Niger	F	45	0.002	0.001	0.003	0.41	0.25	0.64	0.23	0.12	0.39	512.7	439.2	598.8
Niger	F	55	0.002	0.001	0.003	0.46	0.28	0.71	0.18	0.10	0.31	525.7	452.6	610.4
Niger	F	65	0.002	0.001	0.003	0.51	0.31	0.79	0.15	0.08	0.25	540.6	460.9	631.6
Niger	F	75	0.002	0.001	0.003	0.55	0.35	0.86	0.14	0.07	0.23	554.4	478.3	643.9
Niger	F	90	0.001	0.001	0.003	0.60	0.37	0.93	0.13	0.07	0.21	569.3	490.9	660.5
Niger	M	25	0.002	0.001	0.004	0.43	0.27	0.64	0.51	0.28	0.86	462.5	390.3	543.1
Niger	M	35	0.002	0.001	0.003	0.40	0.25	0.60	0.38	0.21	0.64	465.4	397.9	544.5
Niger	M	45	0.001	0.001	0.003	0.38	0.23	0.57	0.26	0.14	0.45	469.4	399.0	549.8
Niger	M	55	0.001	0.001	0.002	0.42	0.26	0.62	0.20	0.11	0.35	480.7	411.4	564.6
Niger	M	65	0.001	0.001	0.002	0.47	0.29	0.70	0.17	0.09	0.30	493.8	422.6	580.9
Niger	M	75	0.001	0.001	0.002	0.51	0.32	0.75	0.15	0.08	0.27	506.6	434.1	591.9
Niger	M	90	0.001	0.001	0.002	0.55	0.34	0.80	0.14	0.08	0.24	520.6	449.6	608.6
Nigeria	F	25	0.016	0.008	0.028	0.14	0.08	0.25	0.49	0.26	0.80	396.2	307.7	508.6

Country	Sex	Midpoint of age range	Mean juice intake (servings/day)	Juice, lower uncertainty interval	Juice, upper uncertainty interval	Mean milk intake (servings/day)	Milk, lower uncertainty interval	Milk, upper uncertainty interval	Mean SSB intake (servings/day)	SSB, lower uncertainty interval	SSB, upper uncertainty interval	Mean calcium intake (servings/day)	Calcium, lower uncertainty interval	Calcium, upper uncertainty interval
Nigeria	F	35	0.013	0.007	0.023	0.13	0.07	0.23	0.37	0.20	0.61	398.5	309.8	508.9
Nigeria	F	45	0.011	0.006	0.020	0.13	0.07	0.22	0.25	0.14	0.43	401.6	311.8	508.6
Nigeria	F	55	0.011	0.006	0.018	0.14	0.08	0.24	0.20	0.11	0.33	411.8	323.3	524.9
Nigeria	F	65	0.010	0.005	0.019	0.16	0.09	0.27	0.16	0.09	0.27	423.4	330.8	540.2
Nigeria	F	75	0.010	0.005	0.018	0.17	0.09	0.29	0.15	0.08	0.24	434.3	343.2	554.5
Nigeria	F	90	0.010	0.005	0.017	0.18	0.10	0.31	0.14	0.08	0.23	446.5	351.7	570.7
Nigeria	M	25	0.013	0.007	0.024	0.13	0.07	0.22	0.55	0.30	0.90	363.9	281.9	469.7
Nigeria	M	35	0.011	0.005	0.019	0.12	0.07	0.21	0.41	0.23	0.66	366.2	283.3	469.0
Nigeria	M	45	0.009	0.004	0.016	0.12	0.07	0.20	0.28	0.15	0.46	369.2	283.6	474.8
Nigeria	M	55	0.008	0.004	0.015	0.13	0.07	0.22	0.22	0.12	0.36	378.2	292.6	482.8
Nigeria	M	65	0.008	0.004	0.015	0.15	0.08	0.24	0.18	0.10	0.30	388.5	301.2	498.6
Nigeria	M	75	0.008	0.004	0.015	0.16	0.09	0.26	0.16	0.09	0.26	398.6	309.2	509.9
Nigeria	M	90	0.008	0.004	0.015	0.17	0.10	0.28	0.15	0.08	0.24	410.0	316.7	523.4
Nicaragua	F	25	0.128	0.062	0.245	0.95	0.52	1.57	2.91	1.56	4.95	706.8	536.8	909.8
Nicaragua	F	35	0.106	0.052	0.197	0.89	0.50	1.47	2.27	1.22	3.85	712.1	542.3	911.4
Nicaragua	F	45	0.086	0.042	0.158	0.86	0.48	1.41	1.66	0.89	2.88	718.8	546.7	922.4
Nicaragua	F	55	0.083	0.041	0.149	0.93	0.53	1.50	1.33	0.72	2.26	736.5	562.1	948.1
Nicaragua	F	65	0.083	0.040	0.154	1.04	0.58	1.70	1.07	0.58	1.86	756.6	577.7	969.8
Nicaragua	F	75	0.081	0.039	0.150	1.12	0.63	1.82	0.96	0.52	1.65	775.6	593.4	992.9
Nicaragua	F	90	0.078	0.038	0.145	1.22	0.67	2.01	0.86	0.48	1.48	798.4	612.7	1020.6
Nicaragua	M	25	0.108	0.054	0.196	0.86	0.46	1.43	3.19	1.66	5.49	646.0	499.4	825.7
Nicaragua	M	35	0.089	0.045	0.161	0.81	0.42	1.35	2.49	1.32	4.28	649.6	511.3	830.6
Nicaragua	M	45	0.072	0.036	0.133	0.78	0.40	1.31	1.81	0.96	3.16	654.7	515.1	840.3
Nicaragua	M	55	0.070	0.035	0.126	0.85	0.44	1.42	1.46	0.77	2.52	671.7	525.3	867.3
Nicaragua	M	65	0.070	0.035	0.126	0.95	0.50	1.59	1.18	0.61	2.07	691.2	536.0	897.5
Nicaragua	M	75	0.068	0.034	0.124	1.03	0.55	1.73	1.06	0.55	1.83	709.3	551.4	916.3
Nicaragua	M	90	0.067	0.033	0.121	1.12	0.59	1.89	0.96	0.50	1.62	730.5	566.3	932.8
Netherlands	F	25	0.386	0.318	0.466	0.73	0.62	0.85	0.68	0.56	0.81	941.1	881.5	1008.0
Netherlands	F	35	0.310	0.260	0.367	0.69	0.60	0.78	0.48	0.41	0.57	948.0	896.4	1005.9
Netherlands	F	45	0.242	0.198	0.291	0.66	0.57	0.76	0.31	0.25	0.37	956.5	892.0	1026.1
Netherlands	F	55	0.235	0.196	0.277	0.70	0.61	0.80	0.24	0.20	0.29	977.6	924.9	1035.5
Netherlands	F	65	0.239	0.196	0.287	0.76	0.65	0.87	0.21	0.17	0.25	1001.5	940.3	1067.4
Netherlands	F	75	0.239	0.199	0.285	0.83	0.73	0.95	0.19	0.16	0.23	1026.9	970.3	1088.3
Netherlands	F	90	0.237	0.196	0.286	0.93	0.80	1.06	0.18	0.15	0.21	1059.1	998.5	1120.0
Netherlands	M	25	0.308	0.250	0.371	0.70	0.60	0.81	0.79	0.66	0.95	867.0	808.3	929.0
Netherlands	M	35	0.249	0.206	0.296	0.64	0.55	0.72	0.56	0.47	0.66	870.6	820.8	921.0
Netherlands	M	45	0.197	0.162	0.240	0.58	0.50	0.67	0.34	0.28	0.41	875.9	817.0	935.6
Netherlands	M	55	0.187	0.156	0.224	0.62	0.54	0.71	0.27	0.22	0.32	894.7	843.7	945.3
Netherlands	M	65	0.186	0.152	0.224	0.68	0.59	0.79	0.22	0.18	0.27	916.5	859.5	978.5
Netherlands	M	75	0.188	0.156	0.223	0.75	0.66	0.86	0.20	0.17	0.24	939.6	886.2	994.9
Netherlands	M	90	0.191	0.157	0.230	0.85	0.73	0.97	0.19	0.16	0.23	967.4	910.1	1026.4
Norway	F	25	0.461	0.370	0.566	1.01	0.84	1.21	0.98	0.78	1.21	847.2	786.5	912.8
Norway	F	35	0.370	0.300	0.451	0.95	0.80	1.12	0.70	0.57	0.85	853.5	803.9	907.3
Norway	F	45	0.288	0.228	0.357	0.91	0.75	1.09	0.44	0.35	0.54	861.1	803.1	925.9
Norway	F	55	0.280	0.225	0.342	0.97	0.81	1.14	0.35	0.29	0.43	880.1	828.7	937.3
Norway	F	65	0.285	0.227	0.351	1.05	0.88	1.23	0.30	0.24	0.38	901.6	838.1	963.4
Norway	F	75	0.285	0.231	0.349	1.15	0.97	1.34	0.28	0.22	0.34	924.5	865.0	983.4
Norway	F	90	0.283	0.228	0.347	1.29	1.08	1.53	0.26	0.20	0.31	954.3	894.7	1018.0
Norway	M	25	0.369	0.299	0.460	0.96	0.79	1.14	1.14	0.91	1.40	780.2	729.2	834.7
Norway	M	35	0.299	0.244	0.365	0.87	0.74	1.02	0.80	0.65	0.98	783.5	740.0	832.8
Norway	M	45	0.236	0.187	0.297	0.80	0.67	0.96	0.49	0.40	0.61	788.2	736.1	843.3
Norway	M	55	0.224	0.181	0.273	0.86	0.72	1.01	0.38	0.31	0.47	805.2	759.5	854.5
Norway	M	65	0.223	0.178	0.275	0.94	0.77	1.11	0.32	0.25	0.40	824.8	769.8	881.3
Norway	M	75	0.225	0.183	0.277	1.04	0.87	1.22	0.29	0.23	0.36	845.5	795.9	896.9
Norway	M	90	0.228	0.186	0.284	1.17	0.97	1.37	0.27	0.21	0.34	871.5	821.8	923.5
Nepal	F	25	0.006	0.003	0.011	0.27	0.20	0.36	0.23	0.12	0.41	412.2	372.3	453.4
Nepal	F	35	0.005	0.002	0.009	0.26	0.20	0.35	0.17	0.09	0.30	415.1	378.5	452.0
Nepal	F	45	0.004	0.002	0.007	0.26	0.19	0.35	0.12	0.06	0.21	418.8	379.9	459.4
Nepal	F	55	0.004	0.002	0.007	0.28	0.21	0.37	0.09	0.05	0.17	428.9	390.6	467.9
Nepal	F	65	0.004	0.002	0.007	0.31	0.24	0.42	0.08	0.04	0.14	440.4	398.6	485.8
Nepal	F	75	0.004	0.002	0.007	0.34	0.26	0.45	0.07	0.04	0.13	452.1	411.3	496.6
Nepal	F	90	0.004	0.002	0.007	0.37	0.27	0.49	0.07	0.03	0.12	465.6	425.7	510.7
Nepal	M	25	0.005	0.002	0.009	0.25	0.18	0.34	0.25	0.13	0.44	378.0	341.0	418.4
Nepal	M	35	0.004	0.002	0.007	0.24	0.18	0.31	0.19	0.10	0.33	380.5	348.0	418.3
Nepal	M	45	0.003	0.002	0.006	0.24	0.17	0.31	0.13	0.07	0.23	383.8	348.8	424.1
Nepal	M	55	0.003	0.002	0.005	0.26	0.19	0.34	0.10	0.05	0.18	393.4	360.6	431.2
Nepal	M	65	0.003	0.001	0.006	0.29	0.21	0.39	0.08	0.04	0.15	404.4	368.6	444.0
Nepal	M	75	0.003	0.001	0.006	0.31	0.23	0.41	0.08	0.04	0.13	415.2	380.6	452.1
Nepal	M	90	0.003	0.001	0.005	0.34	0.25	0.44	0.07	0.04	0.12	427.3	390.9	465.1
New Zealand	F	25	1.261	0.608	2.285	0.54	0.46	0.64	0.47	0.38	0.57	651.1	600.1	699.8
New Zealand	F	35	1.044	0.525	1.885	0.51	0.44	0.58	0.35	0.30	0.41	656.4	613.5	697.8
New Zealand	F	45	0.849	0.427	1.556	0.49	0.41	0.57	0.24	0.20	0.29	662.9	615.0	714.3
New Zealand	F	55	0.816	0.414	1.499	0.53	0.46	0.61	0.20	0.16	0.23	679.0	637.3	723.3
New Zealand	F	65	0.816	0.411	1.489	0.60	0.51	0.70	0.16	0.14	0.19	697.3	649.4	748.1
New Zealand	F	75	0.800	0.407	1.464	0.65	0.57	0.74	0.15	0.13	0.17	715.2	672.4	762.6
New Zealand	F	90	0.774	0.385	1.415	0.71	0.61	0.82	0.13	0.12	0.16	737.9	690.6	789.4
New Zealand	M	25	1.040	0.501	1.894	0.49	0.42	0.58	0.52	0.42	0.63	594.3	547.5	643.0
New Zealand	M	35	0.862	0.435	1.534	0.46	0.40	0.53	0.39	0.33	0.46	598.5	556.3	641.6
New Zealand	M	45	0.703	0.352	1.271	0.44	0.37	0.51	0.27	0.22	0.32	604.0	557.1	654.0
New Zealand	M	55	0.677	0.341	1.203	0.48	0.42	0.55	0.21	0.18	0.25	619.3	574.7	661.6
New Zealand	M	65	0.677	0.337	1.226	0.54	0.46	0.63	0.18	0.15	0.21	636.8	590.1	685.9
New Zealand	M	75	0.661	0.329	1.183	0.59	0.52	0.67	0.16	0.13	0.19	653.9	609.7	699.5
New Zealand	M	90	0.639	0.321	1.110	0.64	0.56	0.74	0.15	0.13	0.17	674.7	628.6	720.1
Oman	F	25	0.225	0.117	0.393	0.52	0.28	0.88	0.75	0.42	1.19	629.7	488.5	797.7
Oman	F	35	0.188	0.098	0.328	0.50	0.27	0.83	0.57	0.33	0.93	634.3	495.2	802.3
Oman	F	45	0.154	0.081	0.280	0.48	0.26	0.80	0.41	0.24	0.69	640.1	500.9	814.7
Oman	F	55	0.148	0.077	0.261	0.52	0.28	0.89	0.33	0.19	0.55	655.9	511.4	840.0
Oman	F	65	0.148	0.077	0.253	0.58	0.32	1.00	0.28	0.16	0.45	674.0	519.7	855.9
Oman	F	75	0.145	0.074	0.248	0.63	0.35	1.07	0.25	0.14	0.41	691.3	537.8	875.6
Oman	F	90	0.140	0.073	0.244	0.69	0.38	1.15	0.23	0.13	0.37	712.5	556.8	905.1
Oman	M	25	0.187	0.092	0.331	0.47	0.25	0.79	0.82	0.45	1.40	572.2	440.4	726.0
Oman	M	35	0.156	0.077	0.271	0.44	0.24	0.73	0.62	0.34	1.05	575.7	443.6	728.6
Oman	M	45	0.128	0.063	0.227	0.42	0.22	0.70	0.43	0.23	0.74	580.3	447.1	736.3
Oman	M	55	0.123	0.061	0.218	0.46	0.24	0.76	0.34	0.19	0.59	594.7	460.9	749.6
Oman	M	65	0.123	0.062	0.225	0.52	0.27	0.85	0.29	0.16	0.50	611.1	469.0	768.5
Oman	M	75	0.120	0.059	0.218	0.57	0.30	0.94	0.26	0.15	0.45	626.7	483.4	787.0
Oman	M	90	0.115	0.057	0.203	0.62	0.33	1.05	0.24	0.14	0.42	644.9	496.6	817.1
Pakistan	F	25	0.067	0.031	0.119	0.49	0.39	0.61	0.89	0.45	1.58	517.5	398.9	663.7
Pakistan	F	35	0.056	0.026	0.096	0.47	0.38	0.58	0.66	0.35	1.14	521.1	404.3	661.3

Country	Sex	Midpoint of age range	Mean juice intake (servings/day)	Juice, lower uncertainty interval	Juice, upper uncertainty interval	Mean milk intake (servings/day)	Milk, lower uncertainty interval	Milk, upper uncertainty interval	Mean SSB intake (servings/day)	SSB, lower uncertainty interval	SSB, upper uncertainty interval	Mean calcium intake (servings/day)	Calcium, lower uncertainty interval	Calcium, upper uncertainty interval
Pakistan	F	45	0.046	0.022	0.080	0.46	0.37	0.57	0.45	0.24	0.77	525.7	407.5	662.2
Pakistan	F	55	0.044	0.021	0.076	0.51	0.41	0.62	0.36	0.19	0.62	538.3	417.9	681.6
Pakistan	F	65	0.044	0.020	0.079	0.56	0.45	0.70	0.30	0.15	0.52	552.8	426.7	706.6
Pakistan	F	75	0.043	0.020	0.077	0.61	0.49	0.75	0.27	0.14	0.47	567.6	441.2	725.8
Pakistan	F	90	0.042	0.020	0.072	0.67	0.53	0.81	0.25	0.13	0.43	584.8	451.0	745.6
Pakistan	M	25	0.056	0.027	0.100	0.46	0.36	0.57	0.97	0.54	1.64	474.4	357.1	613.4
Pakistan	M	35	0.046	0.023	0.082	0.44	0.35	0.54	0.73	0.40	1.20	477.6	360.0	614.0
Pakistan	M	45	0.038	0.019	0.067	0.43	0.34	0.53	0.50	0.27	0.84	481.7	357.8	620.6
Pakistan	M	55	0.036	0.018	0.063	0.47	0.38	0.58	0.40	0.22	0.67	493.8	370.0	633.6
Pakistan	M	65	0.036	0.018	0.065	0.53	0.42	0.66	0.33	0.18	0.55	507.7	379.3	655.2
Pakistan	M	75	0.035	0.017	0.061	0.57	0.46	0.70	0.30	0.16	0.50	521.2	391.6	671.3
Pakistan	M	90	0.034	0.017	0.060	0.61	0.49	0.75	0.27	0.15	0.46	536.7	403.3	692.7
Panama	F	25	0.352	0.175	0.624	1.18	0.63	2.04	2.19	1.20	3.67	737.4	568.9	956.9
Panama	F	35	0.292	0.149	0.515	1.12	0.60	1.88	1.71	0.96	2.87	742.9	574.2	955.8
Panama	F	45	0.237	0.121	0.429	1.07	0.57	1.83	1.25	0.69	2.13	749.8	578.4	968.2
Panama	F	55	0.228	0.116	0.404	1.17	0.63	1.96	1.00	0.55	1.71	768.5	595.3	991.4
Panama	F	65	0.228	0.113	0.407	1.30	0.68	2.18	0.81	0.44	1.35	789.7	609.4	1022.4
Panama	F	75	0.223	0.112	0.389	1.40	0.75	2.37	0.72	0.39	1.20	809.4	626.9	1045.3
Panama	F	90	0.216	0.106	0.386	1.52	0.81	2.56	0.65	0.36	1.09	832.9	647.0	1075.6
Panama	M	25	0.292	0.153	0.522	1.06	0.58	1.81	2.44	1.37	4.24	681.5	523.1	866.4
Panama	M	35	0.241	0.126	0.423	1.00	0.56	1.68	1.90	1.06	3.17	685.3	528.6	863.0
Panama	M	45	0.195	0.100	0.353	0.96	0.53	1.61	1.39	0.77	2.33	690.7	534.2	869.0
Panama	M	55	0.188	0.098	0.332	1.05	0.59	1.76	1.12	0.61	1.83	708.6	548.6	887.6
Panama	M	65	0.188	0.099	0.339	1.17	0.66	1.96	0.91	0.50	1.47	729.1	564.9	924.4
Panama	M	75	0.185	0.097	0.334	1.27	0.71	2.11	0.81	0.45	1.32	748.2	578.7	945.8
Panama	M	90	0.180	0.093	0.327	1.37	0.77	2.27	0.74	0.42	1.24	770.5	594.5	977.2
Peru	F	25	0.356	0.181	0.649	0.50	0.26	0.84	1.39	0.74	2.36	636.9	486.5	814.9
Peru	F	35	0.296	0.152	0.540	0.46	0.25	0.77	1.04	0.56	1.72	641.0	496.2	812.7
Peru	F	45	0.241	0.120	0.436	0.44	0.24	0.73	0.71	0.37	1.18	646.4	502.2	817.4
Peru	F	55	0.232	0.119	0.420	0.48	0.27	0.81	0.56	0.30	0.93	663.1	519.2	836.1
Peru	F	65	0.231	0.121	0.416	0.54	0.30	0.91	0.46	0.25	0.79	682.1	525.6	870.2
Peru	F	75	0.227	0.120	0.409	0.58	0.32	0.97	0.42	0.23	0.72	699.4	540.1	888.0
Peru	F	90	0.220	0.112	0.398	0.63	0.35	1.05	0.39	0.21	0.66	719.5	553.7	925.2
Peru	M	25	0.290	0.150	0.535	0.46	0.24	0.81	1.52	0.80	2.56	582.1	434.4	759.1
Peru	M	35	0.240	0.126	0.434	0.43	0.23	0.74	1.13	0.62	1.88	585.5	444.7	760.3
Peru	M	45	0.195	0.098	0.371	0.41	0.22	0.71	0.76	0.41	1.29	590.1	446.4	775.3
Peru	M	55	0.189	0.098	0.345	0.45	0.25	0.75	0.61	0.33	1.02	604.4	463.9	782.7
Peru	M	65	0.190	0.099	0.352	0.50	0.27	0.85	0.50	0.27	0.85	620.9	473.0	793.9
Peru	M	75	0.185	0.098	0.343	0.54	0.30	0.91	0.46	0.25	0.77	637.1	485.8	813.0
Peru	M	90	0.179	0.095	0.335	0.58	0.32	1.00	0.42	0.23	0.72	655.9	502.9	838.4
Philippines	F	25	0.120	0.092	0.156	0.73	0.59	0.90	0.74	0.40	1.24	864.7	796.9	937.1
Philippines	F	35	0.101	0.078	0.128	0.70	0.59	0.83	0.56	0.31	0.94	872.2	814.1	931.7
Philippines	F	45	0.084	0.064	0.108	0.68	0.56	0.82	0.40	0.22	0.68	881.1	814.9	953.9
Philippines	F	55	0.081	0.063	0.104	0.75	0.63	0.90	0.32	0.18	0.53	901.0	840.1	964.7
Philippines	F	65	0.081	0.062	0.105	0.85	0.69	1.03	0.26	0.14	0.44	923.8	854.8	998.3
Philippines	F	75	0.079	0.061	0.102	0.92	0.77	1.10	0.24	0.13	0.39	949.4	886.2	1016.9
Philippines	F	90	0.076	0.059	0.098	1.00	0.83	1.20	0.22	0.12	0.35	979.5	914.8	1046.5
Philippines	M	25	0.094	0.071	0.127	0.69	0.56	0.84	0.82	0.45	1.40	790.8	733.8	858.2
Philippines	M	35	0.081	0.062	0.106	0.65	0.54	0.77	0.62	0.34	1.05	797.6	747.8	853.6
Philippines	M	45	0.068	0.051	0.091	0.62	0.50	0.74	0.43	0.23	0.73	805.7	747.5	867.5
Philippines	M	55	0.067	0.051	0.087	0.68	0.57	0.82	0.35	0.19	0.58	824.6	771.7	878.2
Philippines	M	65	0.068	0.051	0.090	0.77	0.63	0.93	0.29	0.16	0.47	846.0	783.1	908.8
Philippines	M	75	0.066	0.049	0.086	0.84	0.69	1.00	0.26	0.15	0.43	868.1	810.4	926.6
Philippines	M	90	0.062	0.046	0.081	0.91	0.74	1.09	0.24	0.13	0.40	893.1	834.6	955.5
Papua New Guinea	F	25	0.076	0.040	0.135	0.13	0.07	0.23	0.65	0.36	1.12	413.2	318.7	520.3
Papua New Guinea	F	35	0.063	0.033	0.112	0.12	0.07	0.21	0.49	0.27	0.83	415.4	320.9	526.0
Papua New Guinea	F	45	0.052	0.027	0.094	0.12	0.06	0.20	0.33	0.18	0.57	418.7	321.7	535.6
Papua New Guinea	F	55	0.050	0.026	0.091	0.13	0.07	0.22	0.26	0.15	0.44	429.3	333.7	548.8
Papua New Guinea	F	65	0.050	0.026	0.090	0.14	0.08	0.25	0.22	0.12	0.36	441.5	343.8	560.8
Papua New Guinea	F	75	0.049	0.025	0.088	0.16	0.09	0.26	0.20	0.11	0.33	453.1	352.0	574.0
Papua New Guinea	F	90	0.047	0.025	0.083	0.17	0.10	0.29	0.18	0.10	0.31	466.3	364.7	592.4
Papua New Guinea	M	25	0.064	0.032	0.116	0.12	0.07	0.21	0.71	0.38	1.22	379.0	292.1	491.9
Papua New Guinea	M	35	0.053	0.028	0.096	0.12	0.06	0.19	0.53	0.29	0.91	380.9	295.6	493.9
Papua New Guinea	M	45	0.043	0.023	0.081	0.11	0.06	0.18	0.37	0.19	0.63	383.8	297.5	499.6
Papua New Guinea	M	55	0.041	0.022	0.076	0.12	0.06	0.20	0.29	0.16	0.50	393.8	308.0	507.6
Papua New Guinea	M	65	0.041	0.022	0.076	0.13	0.07	0.23	0.24	0.13	0.42	405.2	314.3	522.0
Papua New Guinea	M	75	0.040	0.021	0.075	0.15	0.08	0.25	0.22	0.12	0.37	415.8	324.5	535.5
Papua New Guinea	M	90	0.039	0.021	0.073	0.16	0.08	0.26	0.20	0.11	0.34	427.4	333.2	551.3
Poland	F	25	0.316	0.231	0.421	0.69	0.58	0.82	0.18	0.14	0.23	516.6	480.6	556.1
Poland	F	35	0.263	0.197	0.342	0.66	0.56	0.76	0.13	0.10	0.17	519.0	487.1	551.0
Poland	F	45	0.213	0.156	0.287	0.63	0.53	0.75	0.09	0.07	0.12	522.7	484.6	560.1
Poland	F	55	0.201	0.152	0.265	0.70	0.59	0.82	0.07	0.06	0.09	536.0	503.5	569.9
Poland	F	65	0.196	0.147	0.260	0.79	0.65	0.93	0.06	0.05	0.08	551.2	514.4	591.6
Poland	F	75	0.192	0.144	0.250	0.86	0.73	1.00	0.06	0.04	0.07	566.0	531.7	602.3
Poland	F	90	0.188	0.141	0.244	0.94	0.80	1.10	0.05	0.04	0.07	583.7	548.2	621.4
Poland	M	25	0.248	0.186	0.330	0.64	0.52	0.77	0.21	0.16	0.27	475.2	439.9	512.2
Poland	M	35	0.205	0.157	0.266	0.60	0.50	0.69	0.16	0.12	0.20	475.8	444.8	508.1
Poland	M	45	0.166	0.123	0.220	0.57	0.47	0.67	0.11	0.08	0.14	477.8	441.8	517.1
Poland	M	55	0.158	0.120	0.207	0.64	0.54	0.74	0.08	0.06	0.11	489.2	457.3	521.1
Poland	M	65	0.157	0.117	0.210	0.72	0.60	0.86	0.07	0.05	0.09	502.6	465.2	539.7
Poland	M	75	0.156	0.116	0.204	0.79	0.67	0.92	0.06	0.05	0.08	517.0	483.3	551.6
Poland	M	90	0.154	0.116	0.200	0.86	0.72	1.00	0.06	0.04	0.07	533.7	501.0	568.1
Democratic People's Republic of Korea	F	25	0.003	0.001	0.005	0.04	0.02	0.07	0.12	0.06	0.20	376.4	283.2	490.4
Democratic People's Republic of Korea	F	35	0.002	0.001	0.004	0.04	0.02	0.07	0.09	0.05	0.15	379.2	286.3	491.0
Democratic People's Republic of Korea	F	45	0.002	0.001	0.003	0.04	0.02	0.06	0.06	0.03	0.11	382.8	286.6	498.0
Democratic People's Republic of Korea	F	55	0.002	0.001	0.003	0.04	0.02	0.07	0.05	0.03	0.08	392.6	298.4	513.3
Democratic People's Republic of Korea	F	65	0.002	0.001	0.003	0.05	0.02	0.08	0.04	0.02	0.07	403.9	307.7	526.3
Democratic People's Republic of Korea	F	75	0.002	0.001	0.003	0.05	0.03	0.08	0.04	0.02	0.06	414.3	313.6	542.1
Democratic People's Republic of Korea	F	90	0.002	0.001	0.003	0.05	0.03	0.09	0.03	0.02	0.06	426.1	322.4	554.3
Democratic People's Republic of Korea	M	25	0.002	0.001	0.004	0.04	0.02	0.06	0.13	0.07	0.21	342.5	256.0	445.5

Country	Sex	Midpoint of age range	Mean juice intake (servings/day)	Juice, lower uncertainty interval	Juice, upper uncertainty interval	Mean milk intake (servings/day)	Milk, lower uncertainty interval	Milk, upper uncertainty interval	Mean SSB intake (servings/day)	SSB, lower uncertainty interval	SSB, upper uncertainty interval	Mean calcium intake (servings/day)	Calcium, lower uncertainty interval	Calcium, upper uncertainty interval
Democratic People's Republic of Korea	M	35	0.002	0.001	0.003	0.03	0.02	0.06	0.09	0.05	0.16	345.0	258.8	446.5
Democratic People's Republic of Korea	M	45	0.001	0.001	0.003	0.03	0.02	0.05	0.06	0.03	0.11	348.2	262.0	449.2
Democratic People's Republic of Korea	M	55	0.001	0.001	0.002	0.04	0.02	0.06	0.05	0.03	0.09	357.2	268.9	459.1
Democratic People's Republic of Korea	M	65	0.001	0.001	0.002	0.04	0.02	0.07	0.04	0.02	0.07	367.6	273.0	469.0
Democratic People's Republic of Korea	M	75	0.001	0.001	0.002	0.04	0.02	0.07	0.04	0.02	0.06	377.3	282.6	480.9
Democratic People's Republic of Korea	M	90	0.001	0.001	0.002	0.05	0.03	0.08	0.04	0.02	0.06	388.0	289.4	496.2
Portugal	F	25	0.262	0.140	0.457	0.79	0.57	1.08	0.49	0.30	0.75	877.9	800.9	962.4
Portugal	F	35	0.211	0.113	0.368	0.75	0.53	1.02	0.35	0.22	0.53	884.3	812.6	961.1
Portugal	F	45	0.164	0.088	0.286	0.71	0.51	0.98	0.22	0.13	0.34	892.3	808.8	976.2
Portugal	F	55	0.159	0.086	0.281	0.76	0.54	1.05	0.18	0.11	0.27	911.9	836.8	992.0
Portugal	F	65	0.163	0.086	0.287	0.82	0.60	1.15	0.15	0.09	0.23	934.3	851.8	1023.2
Portugal	F	75	0.163	0.087	0.285	0.91	0.66	1.27	0.14	0.09	0.22	958.0	879.5	1043.4
Portugal	F	90	0.161	0.087	0.289	1.01	0.73	1.41	0.13	0.08	0.20	987.2	908.3	1073.3
Portugal	M	25	0.208	0.113	0.356	0.76	0.55	1.02	0.58	0.36	0.88	808.4	739.9	884.9
Portugal	M	35	0.169	0.093	0.282	0.69	0.51	0.93	0.41	0.26	0.62	811.7	745.9	878.8
Portugal	M	45	0.133	0.073	0.227	0.63	0.46	0.87	0.25	0.16	0.38	816.7	746.0	883.5
Portugal	M	55	0.126	0.070	0.214	0.67	0.49	0.92	0.19	0.12	0.29	834.2	767.4	904.5
Portugal	M	65	0.126	0.068	0.212	0.74	0.54	1.00	0.16	0.10	0.25	854.6	782.0	936.9
Portugal	M	75	0.127	0.069	0.216	0.82	0.60	1.09	0.15	0.09	0.23	876.1	806.4	955.2
Portugal	M	90	0.129	0.070	0.221	0.92	0.67	1.23	0.14	0.09	0.21	901.9	829.5	980.5
Paraguay	F	25	0.135	0.069	0.247	0.35	0.19	0.57	0.54	0.29	0.95	518.3	398.0	668.7
Paraguay	F	35	0.112	0.059	0.201	0.32	0.18	0.52	0.41	0.22	0.71	521.2	399.9	673.7
Paraguay	F	45	0.092	0.048	0.165	0.31	0.17	0.51	0.29	0.15	0.50	525.4	401.6	679.7
Paraguay	F	55	0.088	0.046	0.157	0.34	0.19	0.55	0.23	0.12	0.40	539.0	411.9	698.2
Paraguay	F	65	0.088	0.046	0.156	0.38	0.21	0.61	0.19	0.10	0.34	554.6	420.7	724.3
Paraguay	F	75	0.086	0.045	0.153	0.41	0.22	0.66	0.17	0.09	0.30	569.2	432.9	737.4
Paraguay	F	90	0.083	0.043	0.149	0.44	0.25	0.72	0.16	0.09	0.27	586.7	445.0	761.7
Paraguay	M	25	0.112	0.055	0.203	0.31	0.17	0.53	0.61	0.34	1.04	475.1	365.9	604.0
Paraguay	M	35	0.093	0.047	0.167	0.29	0.16	0.50	0.46	0.26	0.78	478.0	368.0	605.9
Paraguay	M	45	0.076	0.038	0.134	0.27	0.15	0.48	0.32	0.17	0.55	481.9	368.5	615.1
Paraguay	M	55	0.073	0.037	0.127	0.30	0.17	0.52	0.26	0.14	0.43	493.9	381.2	624.1
Paraguay	M	65	0.073	0.036	0.127	0.34	0.18	0.58	0.21	0.11	0.36	507.5	393.4	638.6
Paraguay	M	75	0.071	0.035	0.124	0.37	0.20	0.62	0.19	0.10	0.32	520.9	403.9	659.8
Paraguay	M	90	0.069	0.034	0.117	0.40	0.22	0.68	0.17	0.09	0.30	536.8	413.7	683.8
Occupied Palestinian Territory	F	25	0.223	0.112	0.390	0.58	0.32	0.97	0.79	0.42	1.36	647.1	497.7	831.7
Occupied Palestinian Territory	F	35	0.186	0.095	0.320	0.55	0.31	0.93	0.61	0.33	1.03	651.7	500.9	832.5
Occupied Palestinian Territory	F	45	0.152	0.077	0.261	0.53	0.29	0.93	0.43	0.23	0.73	657.5	504.3	832.5
Occupied Palestinian Territory	F	55	0.147	0.076	0.254	0.58	0.32	0.99	0.35	0.19	0.60	673.9	519.3	852.8
Occupied Palestinian Territory	F	65	0.146	0.077	0.259	0.65	0.35	1.07	0.29	0.16	0.51	692.6	534.0	880.0
Occupied Palestinian Territory	F	75	0.143	0.075	0.252	0.70	0.38	1.17	0.27	0.15	0.46	710.4	548.7	908.5
Occupied Palestinian Territory	F	90	0.139	0.073	0.243	0.76	0.42	1.26	0.24	0.13	0.42	730.5	563.1	936.8
Occupied Palestinian Territory	M	25	0.191	0.099	0.341	0.53	0.28	0.90	0.88	0.49	1.46	590.0	457.8	756.6
Occupied Palestinian Territory	M	35	0.159	0.082	0.288	0.50	0.27	0.84	0.66	0.37	1.07	593.5	459.8	758.1
Occupied Palestinian Territory	M	45	0.130	0.067	0.237	0.48	0.26	0.80	0.46	0.25	0.75	598.3	461.8	761.8
Occupied Palestinian Territory	M	55	0.126	0.064	0.223	0.52	0.28	0.87	0.37	0.21	0.60	613.3	474.7	778.3
Occupied Palestinian Territory	M	65	0.126	0.063	0.222	0.58	0.31	0.96	0.31	0.17	0.50	630.4	483.1	806.2
Occupied Palestinian Territory	M	75	0.122	0.063	0.214	0.64	0.34	1.04	0.28	0.16	0.46	646.2	499.0	829.3
Occupied Palestinian Territory	M	90	0.117	0.061	0.207	0.70	0.37	1.14	0.26	0.15	0.42	663.7	511.3	857.0
Qatar	F	25	0.321	0.152	0.586	0.68	0.37	1.14	0.89	0.46	1.57	668.6	516.9	846.9
Qatar	F	35	0.269	0.128	0.478	0.65	0.35	1.08	0.68	0.36	1.18	673.2	519.1	851.8
Qatar	F	45	0.220	0.107	0.403	0.62	0.33	1.04	0.49	0.26	0.85	679.1	520.4	864.2
Qatar	F	55	0.212	0.104	0.382	0.68	0.36	1.14	0.40	0.22	0.69	695.9	533.4	888.4
Qatar	F	65	0.212	0.101	0.375	0.76	0.41	1.28	0.33	0.18	0.59	715.1	544.2	908.9
Qatar	F	75	0.207	0.100	0.371	0.82	0.44	1.37	0.30	0.16	0.53	733.6	561.1	931.6
Qatar	F	90	0.201	0.097	0.359	0.89	0.49	1.48	0.27	0.15	0.47	755.4	578.7	968.9
Qatar	M	25	0.263	0.133	0.451	0.62	0.34	1.10	0.98	0.53	1.58	611.4	472.7	785.7
Qatar	M	35	0.220	0.115	0.376	0.58	0.32	1.03	0.74	0.41	1.20	615.2	471.4	784.9
Qatar	M	45	0.181	0.094	0.316	0.55	0.31	0.98	0.51	0.28	0.83	620.2	475.7	792.3
Qatar	M	55	0.174	0.089	0.304	0.60	0.34	1.06	0.41	0.23	0.67	635.4	485.9	809.5
Qatar	M	65	0.175	0.088	0.311	0.67	0.38	1.19	0.35	0.19	0.57	652.9	499.7	836.8
Qatar	M	75	0.170	0.086	0.302	0.74	0.41	1.29	0.32	0.17	0.52	669.5	513.5	859.6
Qatar	M	90	0.162	0.084	0.281	0.81	0.45	1.40	0.29	0.16	0.47	688.9	527.1	881.3
Romania	F	25	0.114	0.060	0.198	0.88	0.49	1.47	0.39	0.22	0.66	602.2	540.1	666.3
Romania	F	35	0.094	0.051	0.162	0.83	0.46	1.34	0.29	0.16	0.49	605.0	543.7	668.1
Romania	F	45	0.077	0.040	0.133	0.79	0.44	1.31	0.20	0.11	0.33	609.3	543.9	679.3
Romania	F	55	0.072	0.037	0.124	0.88	0.49	1.44	0.16	0.09	0.26	624.9	560.4	691.6
Romania	F	65	0.071	0.036	0.121	0.99	0.56	1.65	0.13	0.07	0.22	642.7	576.4	712.9
Romania	F	75	0.069	0.036	0.119	1.08	0.61	1.79	0.12	0.07	0.20	659.8	596.5	729.7
Romania	F	90	0.068	0.035	0.117	1.18	0.66	1.93	0.11	0.06	0.18	679.9	612.1	755.2
Romania	M	25	0.092	0.047	0.168	0.79	0.44	1.34	0.45	0.25	0.73	553.7	493.1	619.1
Romania	M	35	0.076	0.038	0.134	0.74	0.42	1.24	0.33	0.18	0.54	554.5	499.8	614.4
Romania	M	45	0.062	0.031	0.109	0.70	0.39	1.18	0.23	0.12	0.38	556.7	498.6	619.0
Romania	M	55	0.059	0.030	0.104	0.78	0.44	1.29	0.18	0.10	0.30	570.1	515.2	632.7
Romania	M	65	0.059	0.029	0.108	0.89	0.50	1.46	0.14	0.08	0.23	585.8	524.7	654.8
Romania	M	75	0.058	0.029	0.105	0.97	0.55	1.60	0.13	0.07	0.21	602.4	543.0	668.9
Romania	M	90	0.057	0.029	0.101	1.05	0.59	1.74	0.12	0.07	0.20	621.7	560.3	689.5
Russian Federation	F	25	0.259	0.185	0.354	0.78	0.62	0.96	0.75	0.41	1.22	755.3	575.4	970.9
Russian Federation	F	35	0.214	0.157	0.288	0.74	0.61	0.89	0.56	0.32	0.90	761.6	586.3	973.2
Russian Federation	F	45	0.172	0.123	0.238	0.72	0.58	0.88	0.38	0.22	0.65	769.3	594.6	981.1

Country	Sex	Midpoint of age range	Mean juice intake (servings/day)	Juice, lower uncertainty interval	Juice, upper uncertainty interval	Mean milk intake (servings/day)	Milk, lower uncertainty interval	Milk, upper uncertainty interval	Mean SSB intake (servings/day)	SSB, lower uncertainty interval	SSB, upper uncertainty interval	Mean calcium intake (servings/day)	Calcium, lower uncertainty interval	Calcium, upper uncertainty interval
Russian Federation	F	55	0.166	0.121	0.225	0.79	0.65	0.95	0.30	0.17	0.50	786.9	606.9	999.4
Russian Federation	F	65	0.166	0.119	0.227	0.88	0.71	1.07	0.25	0.14	0.41	806.9	624.6	1019.9
Russian Federation	F	75	0.162	0.118	0.218	0.95	0.78	1.14	0.23	0.13	0.37	827.4	644.2	1047.2
Russian Federation	F	90	0.157	0.116	0.209	1.02	0.84	1.23	0.21	0.12	0.34	852.5	666.0	1090.7
Russian Federation	M	25	0.214	0.153	0.301	0.72	0.57	0.90	0.83	0.44	1.38	698.5	544.9	880.9
Russian Federation	M	35	0.176	0.130	0.243	0.68	0.56	0.83	0.62	0.34	1.01	701.8	549.4	886.8
Russian Federation	M	45	0.142	0.104	0.196	0.65	0.53	0.81	0.42	0.23	0.72	707.0	549.6	904.9
Russian Federation	M	55	0.137	0.101	0.187	0.73	0.61	0.90	0.34	0.19	0.56	725.7	564.6	912.8
Russian Federation	M	65	0.137	0.096	0.189	0.83	0.68	1.03	0.28	0.15	0.46	747.1	581.9	943.0
Russian Federation	M	75	0.134	0.097	0.183	0.89	0.73	1.08	0.25	0.14	0.41	767.4	601.0	966.5
Russian Federation	M	90	0.130	0.093	0.177	0.95	0.78	1.14	0.23	0.13	0.38	790.5	618.1	994.0
Rwanda	F	25	0.077	0.029	0.168	0.19	0.10	0.34	0.22	0.11	0.38	391.6	299.8	495.6
Rwanda	F	35	0.064	0.025	0.136	0.18	0.10	0.32	0.16	0.09	0.28	393.9	300.3	503.3
Rwanda	F	45	0.052	0.019	0.107	0.17	0.09	0.30	0.11	0.06	0.19	397.1	302.0	511.1
Rwanda	F	55	0.050	0.018	0.104	0.19	0.10	0.32	0.09	0.05	0.15	407.2	312.7	521.8
Rwanda	F	65	0.050	0.018	0.103	0.21	0.11	0.37	0.07	0.04	0.13	418.6	319.9	531.5
Rwanda	F	75	0.049	0.018	0.102	0.23	0.12	0.39	0.07	0.03	0.11	429.6	331.2	543.9
Rwanda	F	90	0.047	0.018	0.097	0.25	0.13	0.43	0.06	0.03	0.11	441.9	338.3	556.1
Rwanda	M	25	0.063	0.022	0.131	0.18	0.10	0.29	0.24	0.13	0.40	356.6	275.8	464.4
Rwanda	M	35	0.052	0.019	0.109	0.17	0.09	0.27	0.18	0.10	0.30	359.0	280.1	465.9
Rwanda	M	45	0.042	0.016	0.089	0.16	0.09	0.26	0.12	0.06	0.21	362.2	282.2	470.3
Rwanda	M	55	0.041	0.015	0.087	0.17	0.10	0.28	0.10	0.05	0.17	371.1	289.1	482.7
Rwanda	M	65	0.041	0.015	0.085	0.19	0.11	0.32	0.08	0.04	0.14	381.3	293.9	501.2
Rwanda	M	75	0.040	0.015	0.081	0.21	0.12	0.34	0.07	0.04	0.13	391.2	302.7	513.9
Rwanda	M	90	0.038	0.014	0.078	0.23	0.13	0.38	0.07	0.04	0.11	402.1	311.6	528.0
Saudi Arabia	F	25	0.507	0.242	0.919	0.63	0.35	1.05	0.71	0.39	1.19	651.0	504.6	826.1
Saudi Arabia	F	35	0.424	0.206	0.777	0.60	0.34	1.00	0.54	0.30	0.90	655.6	505.9	828.6
Saudi Arabia	F	45	0.348	0.166	0.646	0.58	0.32	0.99	0.39	0.22	0.64	661.4	507.2	840.2
Saudi Arabia	F	55	0.334	0.160	0.622	0.63	0.35	1.06	0.31	0.18	0.52	677.9	523.9	857.2
Saudi Arabia	F	65	0.333	0.160	0.617	0.70	0.39	1.19	0.26	0.15	0.43	696.7	540.0	882.8
Saudi Arabia	F	75	0.327	0.159	0.599	0.76	0.43	1.28	0.24	0.14	0.40	714.4	555.5	903.6
Saudi Arabia	F	90	0.316	0.153	0.571	0.83	0.46	1.39	0.22	0.12	0.36	736.1	570.9	929.4
Saudi Arabia	M	25	0.420	0.203	0.757	0.58	0.30	0.98	0.79	0.43	1.37	602.2	461.6	768.2
Saudi Arabia	M	35	0.350	0.170	0.626	0.54	0.29	0.92	0.60	0.33	1.01	606.1	469.5	775.1
Saudi Arabia	M	45	0.287	0.141	0.513	0.52	0.27	0.89	0.41	0.22	0.70	611.1	472.5	783.5
Saudi Arabia	M	55	0.276	0.138	0.495	0.57	0.30	0.96	0.33	0.18	0.57	626.3	488.1	807.1
Saudi Arabia	M	65	0.277	0.141	0.500	0.63	0.32	1.07	0.28	0.16	0.49	643.5	500.2	835.9
Saudi Arabia	M	75	0.269	0.138	0.478	0.69	0.37	1.16	0.26	0.14	0.45	659.9	512.1	851.5
Saudi Arabia	M	90	0.258	0.130	0.460	0.75	0.41	1.27	0.24	0.13	0.41	678.2	520.8	868.8
Sudan	F	25	0.073	0.036	0.141	0.98	0.49	1.72	1.16	0.59	2.03	566.3	422.6	751.7
Sudan	F	35	0.061	0.030	0.115	0.92	0.46	1.60	0.87	0.46	1.46	569.6	423.9	763.6
Sudan	F	45	0.049	0.023	0.093	0.88	0.44	1.54	0.59	0.31	0.99	574.1	423.2	774.8
Sudan	F	55	0.047	0.023	0.087	0.96	0.49	1.68	0.47	0.25	0.78	588.7	438.7	783.9
Sudan	F	65	0.047	0.023	0.087	1.07	0.53	1.89	0.38	0.20	0.65	605.3	453.5	799.9
Sudan	F	75	0.046	0.023	0.086	1.16	0.59	1.99	0.35	0.19	0.59	621.0	466.5	817.4
Sudan	F	90	0.045	0.022	0.084	1.25	0.65	2.14	0.32	0.18	0.54	638.4	476.5	850.3
Sudan	M	25	0.060	0.029	0.110	0.89	0.47	1.55	1.28	0.69	2.24	514.9	388.4	663.5
Sudan	M	35	0.050	0.025	0.088	0.83	0.44	1.42	0.96	0.51	1.67	518.5	393.8	663.1
Sudan	M	45	0.040	0.020	0.072	0.79	0.42	1.34	0.65	0.34	1.16	523.0	391.4	673.4
Sudan	M	55	0.039	0.020	0.068	0.87	0.47	1.47	0.52	0.27	0.91	535.8	407.8	685.7
Sudan	M	65	0.039	0.020	0.068	0.97	0.51	1.68	0.43	0.22	0.75	550.4	417.5	710.1
Sudan	M	75	0.038	0.019	0.066	1.05	0.56	1.81	0.39	0.21	0.67	564.7	430.3	727.7
Sudan	M	90	0.037	0.018	0.066	1.13	0.61	1.99	0.36	0.19	0.61	580.6	443.3	752.1
Senegal	F	25	0.007	0.003	0.014	0.37	0.20	0.63	0.92	0.48	1.54	488.5	376.1	624.2
Senegal	F	35	0.006	0.003	0.011	0.35	0.19	0.59	0.69	0.36	1.15	491.3	382.5	623.8
Senegal	F	45	0.005	0.002	0.009	0.33	0.18	0.56	0.47	0.24	0.81	495.1	387.5	635.2
Senegal	F	55	0.005	0.002	0.009	0.37	0.20	0.62	0.37	0.20	0.63	507.7	395.3	644.8
Senegal	F	65	0.005	0.002	0.009	0.41	0.23	0.69	0.31	0.16	0.52	522.2	404.0	664.5
Senegal	F	75	0.004	0.002	0.009	0.45	0.25	0.74	0.28	0.15	0.46	535.4	413.6	677.8
Senegal	F	90	0.004	0.002	0.009	0.48	0.27	0.81	0.26	0.14	0.42	549.5	424.6	702.7
Senegal	M	25	0.006	0.002	0.011	0.35	0.18	0.60	1.05	0.56	1.81	451.2	343.4	580.0
Senegal	M	35	0.005	0.002	0.009	0.32	0.17	0.56	0.78	0.42	1.32	454.1	344.6	571.9
Senegal	M	45	0.004	0.002	0.007	0.31	0.16	0.53	0.53	0.28	0.89	457.9	348.6	576.0
Senegal	M	55	0.004	0.002	0.007	0.34	0.18	0.59	0.42	0.23	0.71	469.0	360.5	585.7
Senegal	M	65	0.004	0.002	0.007	0.38	0.21	0.66	0.35	0.19	0.60	481.8	371.9	608.2
Senegal	M	75	0.004	0.002	0.007	0.41	0.22	0.71	0.32	0.17	0.54	494.3	380.6	619.1
Senegal	M	90	0.003	0.002	0.007	0.44	0.24	0.77	0.29	0.16	0.50	507.7	390.8	640.0
Singapore	F	25	0.333	0.168	0.618	0.31	0.26	0.37	0.64	0.52	0.77	535.9	497.3	579.0
Singapore	F	35	0.277	0.145	0.511	0.30	0.26	0.34	0.47	0.40	0.56	540.5	505.7	575.8
Singapore	F	45	0.226	0.113	0.417	0.29	0.24	0.33	0.32	0.26	0.39	546.1	507.7	585.7
Singapore	F	55	0.218	0.112	0.400	0.31	0.27	0.36	0.25	0.21	0.31	559.3	523.5	593.8
Singapore	F	65	0.219	0.112	0.403	0.35	0.30	0.41	0.20	0.17	0.24	574.2	535.3	617.2
Singapore	F	75	0.214	0.109	0.393	0.37	0.32	0.43	0.19	0.16	0.23	588.7	551.5	627.6
Singapore	F	90	0.206	0.105	0.369	0.40	0.34	0.46	0.19	0.15	0.22	605.0	567.0	644.8
Singapore	M	25	0.268	0.147	0.481	0.28	0.23	0.34	0.74	0.61	0.89	489.5	453.3	525.7
Singapore	M	35	0.224	0.122	0.400	0.26	0.22	0.30	0.56	0.48	0.66	493.5	462.7	525.5
Singapore	M	45	0.184	0.095	0.337	0.25	0.21	0.29	0.40	0.33	0.48	498.4	463.6	536.8
Singapore	M	55	0.177	0.092	0.322	0.27	0.23	0.31	0.31	0.26	0.37	510.3	478.9	544.0
Singapore	M	65	0.176	0.094	0.323	0.31	0.26	0.36	0.24	0.20	0.29	523.9	488.6	564.4
Singapore	M	75	0.172	0.093	0.318	0.34	0.29	0.39	0.22	0.18	0.26	538.6	504.9	575.4
Singapore	M	90	0.167	0.090	0.308	0.37	0.31	0.43	0.20	0.17	0.24	555.5	521.0	594.1
Solomon Islands	F	25	0.036	0.018	0.066	0.14	0.08	0.23	0.50	0.28	0.85	419.7	325.3	529.2
Solomon Islands	F	35	0.030	0.015	0.055	0.13	0.07	0.21	0.37	0.21	0.61	422.1	326.5	529.8
Solomon Islands	F	45	0.024	0.012	0.046	0.12	0.07	0.21	0.25	0.14	0.43	425.4	326.9	540.3
Solomon Islands	F	55	0.023	0.012	0.043	0.13	0.07	0.22	0.20	0.12	0.34	436.2	336.6	551.1
Solomon Islands	F	65	0.023	0.012	0.042	0.15	0.08	0.25	0.17	0.09	0.27	448.6	342.8	572.8
Solomon Islands	F	75	0.023	0.012	0.041	0.16	0.09	0.27	0.15	0.08	0.25	460.4	352.5	586.0
Solomon Islands	F	90	0.022	0.012	0.041	0.17	0.10	0.28	0.14	0.08	0.23	473.5	365.6	598.1
Solomon Islands	M	25	0.031	0.016	0.053	0.13	0.07	0.22	0.54	0.30	0.90	381.6	288.6	482.8
Solomon Islands	M	35	0.025	0.013	0.043	0.12	0.06	0.20	0.41	0.22	0.67	383.6	293.5	485.2
Solomon Islands	M	45	0.020	0.011	0.035	0.11	0.06	0.19	0.28	0.15	0.47	386.6	295.3	490.0
Solomon Islands	M	55	0.020	0.010	0.034	0.12	0.07	0.21	0.22	0.12	0.37	396.6	302.8	503.1
Solomon Islands	M	65	0.020	0.010	0.034	0.14	0.07	0.24	0.18	0.10	0.31	408.2	308.9	518.3
Solomon Islands	M	75	0.019	0.010	0.033	0.15	0.08	0.26	0.16	0.09	0.27	418.9	317.6	529.1
Solomon Islands	M	90	0.019	0.010	0.032	0.16	0.09	0.27	0.15	0.08	0.25	430.6	326.7	539.6
Sierra Leone	F	25	0.007	0.004	0.012	0.13	0.07	0.23	0.45	0.24	0.75	390.7	301.6	502.8
Sierra Leone	F	35	0.006	0.003	0.010	0.12	0.07	0.22	0.33	0.18	0.55	393.0	303.7	502.4
Sierra Leone	F	45	0.005	0.002	0.008	0.12	0.06	0.21	0.23	0.12	0.38	396.1	306.9	501.2
Sierra Leone	F	55	0.005	0.002	0.008	0.13	0.07	0.23	0.18	0.10	0.30	406.2	311.9	515.6

Country	Sex	Midpoint of age range	Mean juice intake (servings/day)	Juice, lower uncertainty interval	Juice, upper uncertainty interval	Mean milk intake (servings/day)	Milk, lower uncertainty interval	Milk, upper uncertainty interval	Mean SSB intake (servings/day)	SSB, lower uncertainty interval	SSB, upper uncertainty interval	Mean calcium intake (servings/day)	Calcium, lower uncertainty interval	Calcium, upper uncertainty interval
Sierra Leone	F	65	0.005	0.002	0.008	0.15	0.08	0.26	0.15	0.08	0.25	417.6	319.0	532.4
Sierra Leone	F	75	0.004	0.002	0.008	0.16	0.08	0.28	0.14	0.07	0.22	428.2	329.8	543.5
Sierra Leone	F	90	0.004	0.002	0.007	0.17	0.09	0.30	0.13	0.07	0.20	439.3	339.6	556.4
Sierra Leone	M	25	0.006	0.003	0.011	0.12	0.06	0.21	0.50	0.27	0.88	358.3	274.4	456.2
Sierra Leone	M	35	0.005	0.002	0.009	0.12	0.06	0.19	0.37	0.21	0.63	360.6	276.7	454.8
Sierra Leone	M	45	0.004	0.002	0.007	0.11	0.06	0.18	0.25	0.14	0.43	363.6	277.2	459.2
Sierra Leone	M	55	0.004	0.002	0.007	0.12	0.07	0.20	0.20	0.11	0.34	372.4	286.3	469.2
Sierra Leone	M	65	0.004	0.002	0.007	0.13	0.07	0.23	0.17	0.09	0.28	382.5	295.6	487.4
Sierra Leone	M	75	0.004	0.002	0.007	0.15	0.08	0.24	0.15	0.08	0.25	392.4	302.4	500.0
Sierra Leone	M	90	0.004	0.002	0.006	0.16	0.08	0.27	0.14	0.08	0.22	403.1	311.1	515.1
El Salvador	F	25	0.458	0.245	0.820	1.12	0.62	1.91	2.79	1.50	4.77	730.2	566.8	922.5
El Salvador	F	35	0.379	0.205	0.671	1.06	0.58	1.77	2.17	1.17	3.68	735.6	564.6	917.0
El Salvador	F	45	0.307	0.163	0.549	1.01	0.55	1.70	1.59	0.83	2.67	742.4	564.6	928.9
El Salvador	F	55	0.295	0.160	0.521	1.11	0.62	1.87	1.27	0.68	2.15	760.9	581.0	954.7
El Salvador	F	65	0.295	0.159	0.524	1.23	0.68	2.11	1.02	0.55	1.71	781.9	596.7	983.9
El Salvador	F	75	0.289	0.158	0.512	1.33	0.74	2.28	0.92	0.50	1.55	801.4	613.3	1008.0
El Salvador	F	90	0.280	0.151	0.489	1.44	0.81	2.44	0.83	0.45	1.38	825.0	633.4	1029.7
El Salvador	M	25	0.379	0.193	0.666	1.01	0.56	1.72	3.03	1.77	5.20	673.0	519.3	860.2
El Salvador	M	35	0.314	0.161	0.557	0.96	0.54	1.60	2.36	1.40	3.96	676.9	525.2	858.6
El Salvador	M	45	0.254	0.128	0.466	0.91	0.51	1.53	1.72	1.00	2.83	682.3	530.1	864.7
El Salvador	M	55	0.244	0.123	0.443	1.00	0.55	1.67	1.38	0.81	2.23	700.0	548.9	881.4
El Salvador	M	65	0.245	0.120	0.449	1.12	0.60	1.87	1.12	0.65	1.84	720.2	560.1	911.7
El Salvador	M	75	0.240	0.118	0.436	1.21	0.67	2.04	1.01	0.58	1.68	739.1	576.4	932.5
El Salvador	M	90	0.233	0.117	0.412	1.32	0.74	2.23	0.91	0.53	1.53	761.9	593.3	967.9
Somalia	F	25	0.015	0.007	0.027	0.16	0.08	0.27	0.45	0.24	0.79	370.6	284.0	478.6
Somalia	F	35	0.013	0.006	0.022	0.15	0.08	0.25	0.34	0.18	0.57	372.9	285.8	477.5
Somalia	F	45	0.010	0.005	0.018	0.14	0.08	0.25	0.23	0.12	0.39	375.9	286.5	479.3
Somalia	F	55	0.010	0.005	0.018	0.16	0.08	0.27	0.18	0.10	0.31	385.5	293.3	492.2
Somalia	F	65	0.010	0.005	0.018	0.17	0.09	0.31	0.15	0.08	0.26	396.3	303.0	513.0
Somalia	F	75	0.010	0.005	0.017	0.19	0.10	0.33	0.14	0.08	0.23	406.6	311.6	519.6
Somalia	F	90	0.009	0.005	0.017	0.20	0.11	0.35	0.13	0.07	0.21	418.2	318.8	533.4
Somalia	M	25	0.012	0.006	0.022	0.14	0.08	0.24	0.51	0.27	0.87	340.0	262.3	440.6
Somalia	M	35	0.010	0.005	0.018	0.13	0.07	0.22	0.38	0.20	0.66	342.2	266.8	434.4
Somalia	M	45	0.008	0.004	0.015	0.13	0.07	0.22	0.26	0.14	0.46	345.1	267.3	441.6
Somalia	M	55	0.008	0.004	0.014	0.14	0.08	0.24	0.21	0.11	0.36	353.6	274.9	450.7
Somalia	M	65	0.008	0.004	0.014	0.15	0.08	0.27	0.17	0.09	0.30	363.3	281.8	464.0
Somalia	M	75	0.008	0.004	0.014	0.17	0.09	0.29	0.15	0.08	0.27	372.7	290.8	476.4
Somalia	M	90	0.008	0.004	0.013	0.18	0.10	0.31	0.14	0.07	0.25	383.2	298.4	487.8
Serbia	F	25	0.608	0.362	0.948	0.60	0.42	0.83	0.52	0.32	0.80	611.6	465.5	788.1
Serbia	F	35	0.505	0.300	0.782	0.57	0.40	0.78	0.39	0.24	0.60	614.5	469.1	788.1
Serbia	F	45	0.410	0.242	0.645	0.55	0.38	0.75	0.26	0.16	0.42	618.9	471.6	792.4
Serbia	F	55	0.386	0.231	0.600	0.61	0.43	0.83	0.21	0.13	0.33	634.6	487.6	810.1
Serbia	F	65	0.377	0.223	0.589	0.68	0.48	0.94	0.18	0.11	0.28	652.6	496.8	840.2
Serbia	F	75	0.370	0.221	0.573	0.74	0.53	1.02	0.16	0.10	0.25	670.2	513.7	860.0
Serbia	F	90	0.362	0.216	0.557	0.81	0.58	1.11	0.15	0.09	0.23	690.3	525.1	885.3
Serbia	M	25	0.482	0.283	0.745	0.55	0.38	0.76	0.61	0.37	0.95	562.1	434.2	711.9
Serbia	M	35	0.399	0.235	0.619	0.52	0.36	0.70	0.46	0.29	0.70	562.9	437.4	709.8
Serbia	M	45	0.322	0.189	0.518	0.49	0.34	0.67	0.31	0.19	0.49	565.2	436.3	715.4
Serbia	M	55	0.307	0.184	0.479	0.55	0.38	0.74	0.24	0.15	0.38	578.8	450.4	730.5
Serbia	M	65	0.305	0.184	0.475	0.62	0.43	0.86	0.19	0.12	0.30	594.8	464.7	752.5
Serbia	M	75	0.302	0.182	0.468	0.68	0.48	0.93	0.18	0.11	0.27	611.7	476.9	774.6
Serbia	M	90	0.299	0.178	0.461	0.74	0.51	1.01	0.17	0.10	0.25	631.3	493.8	804.0
São Tomé and Príncipe	F	25	0.369	0.164	0.710	0.35	0.18	0.61	1.17	0.60	2.09	487.1	375.1	620.9
São Tomé and Príncipe	F	35	0.307	0.137	0.576	0.33	0.18	0.57	0.88	0.46	1.52	490.0	377.9	620.2
São Tomé and Príncipe	F	45	0.249	0.109	0.471	0.32	0.17	0.56	0.60	0.31	1.04	494.0	378.7	630.1
São Tomé and Príncipe	F	55	0.239	0.106	0.459	0.35	0.19	0.61	0.47	0.25	0.83	506.4	392.9	641.8
São Tomé and Príncipe	F	65	0.239	0.105	0.462	0.39	0.22	0.68	0.39	0.20	0.68	520.7	403.7	663.9
São Tomé and Príncipe	F	75	0.234	0.104	0.457	0.42	0.23	0.73	0.35	0.19	0.61	534.1	416.5	677.7
São Tomé and Príncipe	F	90	0.227	0.103	0.450	0.46	0.25	0.79	0.33	0.17	0.56	549.3	424.8	695.1
São Tomé and Príncipe	M	25	0.302	0.140	0.611	0.33	0.19	0.57	1.32	0.69	2.30	448.3	341.8	563.9
São Tomé and Príncipe	M	35	0.251	0.118	0.513	0.31	0.18	0.52	0.99	0.52	1.67	451.2	345.8	569.4
São Tomé and Príncipe	M	45	0.204	0.095	0.416	0.30	0.16	0.50	0.67	0.35	1.13	455.1	346.1	579.3
São Tomé and Príncipe	M	55	0.197	0.092	0.398	0.33	0.18	0.53	0.53	0.29	0.88	466.2	355.5	587.4
São Tomé and Príncipe	M	65	0.197	0.092	0.388	0.36	0.20	0.59	0.44	0.23	0.74	478.9	364.2	608.2
São Tomé and Príncipe	M	75	0.193	0.091	0.388	0.40	0.22	0.65	0.40	0.21	0.68	491.4	376.1	623.9
São Tomé and Príncipe	M	90	0.187	0.087	0.374	0.43	0.24	0.71	0.37	0.19	0.63	505.5	385.5	644.1
Suriname	F	25	0.291	0.144	0.532	0.34	0.19	0.57	4.23	2.44	7.25	697.5	536.4	896.5
Suriname	F	35	0.243	0.123	0.443	0.32	0.18	0.53	3.15	1.83	5.24	701.5	541.3	895.3
Suriname	F	45	0.200	0.101	0.372	0.31	0.16	0.52	2.14	1.22	3.62	706.9	546.3	902.7
Suriname	F	55	0.191	0.097	0.351	0.34	0.18	0.56	1.72	0.99	2.87	724.1	561.6	925.2
Suriname	F	65	0.188	0.095	0.345	0.37	0.20	0.61	1.43	0.82	2.38	744.1	575.4	952.1
Suriname	F	75	0.184	0.093	0.339	0.40	0.22	0.66	1.30	0.76	2.17	763.7	591.0	973.9
Suriname	F	90	0.179	0.089	0.321	0.44	0.24	0.72	1.20	0.69	2.00	786.7	605.2	1004.1
Suriname	M	25	0.240	0.122	0.450	0.32	0.19	0.57	4.59	2.52	7.83	646.3	491.1	831.0
Suriname	M	35	0.200	0.103	0.371	0.30	0.17	0.53	3.40	1.94	5.70	650.1	498.2	833.4
Suriname	M	45	0.163	0.081	0.312	0.29	0.16	0.49	2.29	1.29	3.83	655.2	504.6	842.4
Suriname	M	55	0.157	0.080	0.298	0.31	0.18	0.54	1.84	1.04	3.03	670.9	518.7	863.2
Suriname	M	65	0.157	0.079	0.292	0.35	0.20	0.60	1.53	0.86	2.56	689.1	533.7	884.5
Suriname	M	75	0.153	0.077	0.287	0.38	0.21	0.66	1.40	0.80	2.33	707.5	545.8	910.9
Suriname	M	90	0.147	0.075	0.278	0.41	0.23	0.72	1.29	0.75	2.17	728.8	558.7	937.6
Slovakia	F	25	0.223	0.146	0.322	0.54	0.39	0.75	1.02	0.74	1.38	558.2	430.3	715.5
Slovakia	F	35	0.185	0.123	0.264	0.51	0.37	0.69	0.76	0.56	1.02	560.7	435.4	714.3
Slovakia	F	45	0.150	0.098	0.220	0.49	0.36	0.67	0.51	0.37	0.70	564.6	441.4	719.2
Slovakia	F	55	0.142	0.096	0.206	0.55	0.40	0.73	0.41	0.30	0.56	579.1	449.8	741.8
Slovakia	F	65	0.138	0.093	0.201	0.62	0.45	0.84	0.35	0.25	0.47	595.6	458.0	760.9
Slovakia	F	75	0.136	0.091	0.194	0.67	0.49	0.90	0.32	0.23	0.43	611.5	471.6	774.5
Slovakia	F	90	0.132	0.088	0.187	0.73	0.53	0.99	0.29	0.22	0.40	630.4	488.9	799.4
Slovakia	M	25	0.177	0.117	0.259	0.50	0.36	0.68	1.17	0.82	1.62	517.1	395.7	664.7
Slovakia	M	35	0.146	0.098	0.212	0.47	0.34	0.64	0.87	0.62	1.18	517.9	400.9	663.3
Slovakia	M	45	0.119	0.078	0.174	0.44	0.32	0.61	0.59	0.41	0.80	519.9	401.4	664.9
Slovakia	M	55	0.113	0.076	0.164	0.50	0.37	0.67	0.46	0.33	0.62	532.4	410.1	684.7
Slovakia	M	65	0.112	0.076	0.163	0.56	0.41	0.77	0.37	0.26	0.50	547.1	421.0	708.5
Slovakia	M	75	0.111	0.076	0.162	0.61	0.45	0.83	0.34	0.24	0.45	562.6	433.9	722.8
Slovakia	M	90	0.110	0.074	0.161	0.67	0.49	0.91	0.32	0.23	0.42	581.0	443.7	741.7
Slovenia	F	25	0.475	0.301	0.718	0.77	0.56	1.03	0.55	0.36	0.80	811.9	716.0	912.2
Slovenia	F	35	0.394	0.253	0.586	0.73	0.53	0.98	0.41	0.27	0.59	815.6	727.3	912.0
Slovenia	F	45	0.320	0.202	0.476	0.70	0.51	0.95	0.28	0.18	0.41	821.4	731.4	924.3
Slovenia	F	55	0.301	0.190	0.449	0.77	0.58	1.04	0.22	0.15	0.32	842.3	750.4	945.2
Slovenia	F	65	0.295	0.185	0.445	0.87	0.64	1.18	0.19	0.12	0.28	866.4	764.2	975.5

Country	Sex	Midpoint of age range	Mean juice intake (servings/day)	Juice, lower uncertainty interval	Juice, upper uncertainty interval	Mean milk intake (servings/day)	Milk, lower uncertainty interval	Milk, upper uncertainty interval	Mean SSB intake (servings/day)	SSB, lower uncertainty interval	SSB, upper uncertainty interval	Mean calcium intake (servings/day)	Calcium, lower uncertainty interval	Calcium, upper uncertainty interval
Slovenia	F	75	0.289	0.183	0.439	0.95	0.71	1.27	0.17	0.11	0.25	889.6	788.5	991.8
Slovenia	F	90	0.282	0.179	0.427	1.04	0.77	1.39	0.16	0.10	0.23	917.5	819.3	1026.1
Slovenia	M	25	0.377	0.239	0.571	0.71	0.53	0.96	0.65	0.42	0.95	748.2	654.9	845.4
Slovenia	M	35	0.312	0.199	0.471	0.67	0.50	0.89	0.48	0.32	0.71	749.2	656.7	841.5
Slovenia	M	45	0.252	0.160	0.381	0.64	0.47	0.84	0.33	0.22	0.48	752.2	656.7	844.4
Slovenia	M	55	0.241	0.155	0.360	0.71	0.54	0.94	0.25	0.17	0.38	770.2	677.8	862.1
Slovenia	M	65	0.239	0.155	0.364	0.81	0.60	1.07	0.21	0.14	0.31	791.6	696.4	897.1
Slovenia	M	75	0.237	0.155	0.359	0.88	0.67	1.16	0.19	0.12	0.28	813.9	717.5	918.5
Slovenia	M	90	0.234	0.153	0.344	0.96	0.73	1.27	0.18	0.12	0.26	840.1	741.2	943.9
Sweden	F	25	0.557	0.460	0.670	1.62	1.38	1.88	0.99	0.81	1.19	937.6	859.2	1019.9
Sweden	F	35	0.447	0.376	0.530	1.53	1.33	1.74	0.71	0.59	0.84	944.5	874.1	1022.2
Sweden	F	45	0.349	0.286	0.419	1.45	1.26	1.67	0.45	0.37	0.54	953.0	875.9	1034.7
Sweden	F	55	0.338	0.281	0.400	1.55	1.35	1.75	0.36	0.30	0.43	974.0	900.9	1047.9
Sweden	F	65	0.345	0.284	0.411	1.68	1.44	1.94	0.31	0.25	0.37	997.8	918.3	1077.5
Sweden	F	75	0.345	0.288	0.408	1.85	1.61	2.11	0.28	0.23	0.34	1023.0	945.3	1095.6
Sweden	F	90	0.342	0.283	0.409	2.07	1.79	2.39	0.26	0.21	0.31	1056.0	975.8	1136.8
Sweden	M	25	0.446	0.372	0.537	1.54	1.32	1.77	1.16	0.95	1.39	864.1	789.7	941.5
Sweden	M	35	0.361	0.304	0.425	1.40	1.23	1.59	0.81	0.68	0.96	867.7	804.5	938.4
Sweden	M	45	0.285	0.236	0.339	1.29	1.11	1.49	0.50	0.41	0.60	872.9	806.1	952.7
Sweden	M	55	0.271	0.228	0.319	1.37	1.21	1.57	0.39	0.32	0.47	891.7	827.1	964.3
Sweden	M	65	0.269	0.223	0.325	1.50	1.30	1.74	0.32	0.26	0.39	913.5	845.1	991.3
Sweden	M	75	0.272	0.229	0.325	1.66	1.45	1.89	0.30	0.24	0.35	936.4	868.1	1013.0
Sweden	M	90	0.276	0.233	0.331	1.87	1.62	2.15	0.27	0.23	0.33	965.2	892.4	1041.3
Swaziland	F	25	0.095	0.046	0.171	0.83	0.47	1.39	1.34	0.70	2.23	483.6	375.5	620.7
Swaziland	F	35	0.079	0.039	0.143	0.78	0.44	1.29	1.00	0.53	1.64	487.1	380.3	620.9
Swaziland	F	45	0.064	0.031	0.115	0.74	0.41	1.24	0.68	0.36	1.12	491.5	382.9	630.8
Swaziland	F	55	0.061	0.031	0.111	0.81	0.47	1.35	0.54	0.29	0.88	502.7	389.8	641.0
Swaziland	F	65	0.061	0.030	0.113	0.90	0.52	1.51	0.45	0.24	0.75	515.6	397.6	657.4
Swaziland	F	75	0.060	0.029	0.109	0.98	0.56	1.63	0.41	0.22	0.67	529.2	410.4	670.5
Swaziland	F	90	0.058	0.028	0.105	1.06	0.61	1.74	0.37	0.20	0.62	545.0	418.6	689.8
Swaziland	M	25	0.078	0.037	0.140	0.76	0.41	1.34	1.47	0.84	2.51	442.9	338.9	566.3
Swaziland	M	35	0.065	0.032	0.116	0.72	0.39	1.23	1.10	0.64	1.84	445.6	344.5	571.2
Swaziland	M	45	0.052	0.026	0.093	0.68	0.36	1.16	0.75	0.43	1.22	449.3	347.4	577.3
Swaziland	M	55	0.050	0.025	0.089	0.75	0.40	1.27	0.59	0.35	0.97	460.0	355.9	588.8
Swaziland	M	65	0.050	0.025	0.091	0.83	0.45	1.40	0.49	0.29	0.80	472.2	364.1	603.8
Swaziland	M	75	0.049	0.025	0.087	0.90	0.49	1.51	0.44	0.26	0.73	484.7	374.9	622.1
Swaziland	M	90	0.048	0.024	0.083	0.97	0.54	1.65	0.41	0.24	0.68	499.0	387.6	638.2
Seychelles	F	25	0.127	0.062	0.214	0.71	0.36	1.28	0.80	0.43	1.35	700.7	526.5	912.8
Seychelles	F	35	0.107	0.054	0.181	0.68	0.34	1.18	0.61	0.33	1.02	706.7	533.1	923.5
Seychelles	F	45	0.089	0.045	0.153	0.66	0.33	1.14	0.43	0.23	0.72	713.8	536.1	930.7
Seychelles	F	55	0.086	0.044	0.148	0.73	0.38	1.28	0.34	0.19	0.57	730.0	553.2	951.0
Seychelles	F	65	0.086	0.045	0.147	0.83	0.44	1.45	0.28	0.15	0.47	748.3	567.7	967.2
Seychelles	F	75	0.084	0.044	0.143	0.90	0.48	1.59	0.26	0.14	0.42	769.1	582.4	993.6
Seychelles	F	90	0.081	0.043	0.139	0.98	0.51	1.74	0.23	0.13	0.38	793.7	599.9	1031.3
Seychelles	M	25	0.100	0.047	0.181	0.67	0.35	1.14	0.86	0.48	1.42	635.9	473.2	831.0
Seychelles	M	35	0.085	0.040	0.151	0.63	0.33	1.05	0.65	0.37	1.08	641.2	479.2	828.9
Seychelles	M	45	0.072	0.034	0.127	0.60	0.31	0.99	0.45	0.25	0.77	647.7	485.3	834.5
Seychelles	M	55	0.071	0.034	0.123	0.66	0.34	1.11	0.36	0.21	0.60	663.0	497.8	859.9
Seychelles	M	65	0.072	0.035	0.125	0.74	0.39	1.28	0.30	0.17	0.48	680.2	508.0	882.2
Seychelles	M	75	0.069	0.034	0.120	0.81	0.43	1.40	0.27	0.15	0.44	698.0	521.3	908.0
Seychelles	M	90	0.065	0.032	0.114	0.89	0.47	1.52	0.25	0.14	0.41	718.3	533.8	932.5
Syrian Arab Republic	F	25	0.274	0.139	0.491	0.76	0.41	1.32	0.93	0.52	1.60	680.1	525.1	866.7
Syrian Arab Republic	F	35	0.229	0.118	0.402	0.72	0.39	1.24	0.72	0.40	1.20	684.9	530.7	874.6
Syrian Arab Republic	F	45	0.187	0.096	0.333	0.69	0.37	1.23	0.51	0.28	0.87	691.1	531.9	886.1
Syrian Arab Republic	F	55	0.180	0.095	0.316	0.76	0.40	1.32	0.42	0.24	0.69	708.3	547.8	909.1
Syrian Arab Republic	F	65	0.180	0.093	0.317	0.84	0.45	1.50	0.35	0.20	0.59	727.7	562.7	935.7
Syrian Arab Republic	F	75	0.176	0.093	0.310	0.91	0.49	1.61	0.31	0.18	0.53	746.4	579.8	960.2
Syrian Arab Republic	F	90	0.171	0.088	0.299	0.99	0.52	1.73	0.29	0.16	0.48	767.7	599.8	982.5
Syrian Arab Republic	M	25	0.227	0.110	0.416	0.69	0.37	1.20	1.04	0.57	1.73	624.1	478.7	801.2
Syrian Arab Republic	M	35	0.189	0.091	0.338	0.65	0.35	1.13	0.78	0.43	1.26	628.0	486.6	798.4
Syrian Arab Republic	M	45	0.155	0.074	0.278	0.61	0.33	1.07	0.54	0.29	0.90	633.2	489.3	804.5
Syrian Arab Republic	M	55	0.149	0.072	0.264	0.67	0.36	1.14	0.43	0.24	0.72	649.0	499.7	826.5
Syrian Arab Republic	M	65	0.149	0.074	0.268	0.75	0.40	1.30	0.36	0.20	0.61	666.8	507.2	847.3
Syrian Arab Republic	M	75	0.145	0.071	0.260	0.82	0.45	1.41	0.33	0.18	0.55	683.7	522.8	868.5
Syrian Arab Republic	M	90	0.139	0.067	0.250	0.90	0.49	1.56	0.31	0.17	0.52	703.1	543.4	891.1
Chad	F	25	0.005	0.002	0.009	0.38	0.21	0.63	0.65	0.36	1.07	494.7	384.9	634.3
Chad	F	35	0.004	0.002	0.008	0.36	0.19	0.60	0.49	0.28	0.79	497.6	388.5	633.5
Chad	F	45	0.003	0.002	0.006	0.34	0.18	0.59	0.33	0.18	0.54	501.6	386.5	637.0
Chad	F	55	0.003	0.002	0.006	0.37	0.21	0.64	0.26	0.15	0.43	514.4	398.0	657.8
Chad	F	65	0.003	0.001	0.006	0.42	0.23	0.69	0.22	0.12	0.37	528.9	408.8	680.8
Chad	F	75	0.003	0.001	0.006	0.45	0.25	0.74	0.20	0.11	0.32	542.5	421.0	693.3
Chad	F	90	0.003	0.001	0.006	0.49	0.27	0.81	0.18	0.10	0.29	557.5	432.8	715.2
Chad	M	25	0.004	0.002	0.008	0.36	0.20	0.64	0.73	0.41	1.27	457.1	347.9	589.2
Chad	M	35	0.003	0.002	0.006	0.34	0.19	0.59	0.55	0.31	0.92	460.0	352.1	587.9
Chad	M	45	0.003	0.001	0.005	0.32	0.17	0.56	0.37	0.20	0.61	463.8	351.3	589.8
Chad	M	55	0.003	0.001	0.005	0.35	0.19	0.61	0.29	0.17	0.47	475.1	362.0	603.7
Chad	M	65	0.003	0.001	0.005	0.39	0.22	0.69	0.24	0.13	0.39	488.1	372.2	622.9
Chad	M	75	0.003	0.001	0.005	0.43	0.24	0.74	0.22	0.12	0.35	500.8	382.9	634.6
Chad	M	90	0.003	0.001	0.005	0.46	0.25	0.81	0.20	0.11	0.32	514.9	388.1	650.0
Togo	F	25	0.001	0.001	0.003	0.14	0.07	0.23	0.51	0.27	0.88	395.9	306.5	509.2
Togo	F	35	0.001	0.001	0.002	0.13	0.07	0.21	0.38	0.21	0.65	398.3	309.2	512.0
Togo	F	45	0.001	0.000	0.002	0.13	0.07	0.21	0.26	0.14	0.44	401.5	308.0	517.8
Togo	F	55	0.001	0.000	0.002	0.14	0.08	0.22	0.21	0.12	0.34	411.7	315.7	530.7
Togo	F	65	0.001	0.000	0.002	0.16	0.08	0.25	0.17	0.09	0.29	423.3	323.3	549.5
Togo	F	75	0.001	0.000	0.002	0.17	0.09	0.27	0.16	0.08	0.26	434.2	332.6	560.1
Togo	F	90	0.001	0.000	0.002	0.18	0.10	0.29	0.14	0.08	0.24	446.5	339.6	571.4
Togo	M	25	0.001	0.000	0.002	0.13	0.07	0.22	0.58	0.32	1.00	362.4	281.9	467.9
Togo	M	35	0.001	0.000	0.002	0.13	0.07	0.21	0.43	0.24	0.73	364.7	285.5	468.3
Togo	M	45	0.001	0.000	0.001	0.12	0.07	0.20	0.29	0.15	0.50	367.7	286.6	473.0
Togo	M	55	0.001	0.000	0.001	0.13	0.07	0.21	0.23	0.12	0.39	376.7	294.7	479.5
Togo	M	65	0.001	0.000	0.001	0.15	0.08	0.24	0.19	0.10	0.32	386.9	300.7	491.5
Togo	M	75	0.001	0.000	0.001	0.16	0.09	0.26	0.17	0.10	0.29	397.0	308.8	505.4
Togo	M	90	0.001	0.000	0.001	0.17	0.10	0.28	0.16	0.09	0.27	408.5	316.4	521.9
Thailand	F	25	0.338	0.173	0.605	0.27	0.18	0.41	1.10	0.76	1.52	459.4	420.0	499.4
Thailand	F	35	0.285	0.146	0.517	0.26	0.17	0.38	0.84	0.58	1.14	463.4	428.6	499.3
Thailand	F	45	0.237	0.119	0.447	0.25	0.17	0.37	0.59	0.40	0.81	468.1	429.8	510.2
Thailand	F	55	0.230	0.117	0.425	0.28	0.19	0.41	0.47	0.33	0.64	478.7	444.1	516.4
Thailand	F	65	0.230	0.115	0.425	0.32	0.21	0.46	0.39	0.27	0.53	490.8	453.1	533.4
Thailand	F	75	0.224	0.113	0.411	0.35	0.24	0.49	0.35	0.25	0.48	504.5	468.2	544.0

Country	Sex	Midpoint of age range	Mean juice intake (servings/day)	Juice, lower uncertainty interval	Juice, upper uncertainty interval	Mean milk intake (servings/day)	Milk, lower uncertainty interval	Milk, upper uncertainty interval	Mean SSB intake (servings/day)	SSB, lower uncertainty interval	SSB, upper uncertainty interval	Mean calcium intake (servings/day)	Calcium, lower uncertainty interval	Calcium, upper uncertainty interval
Thailand	F	90	0.215	0.110	0.389	0.38	0.26	0.54	0.32	0.23	0.44	521.1	481.0	560.1
Thailand	M	25	0.271	0.133	0.487	0.26	0.17	0.38	1.21	0.83	1.70	420.8	386.8	455.6
Thailand	M	35	0.231	0.112	0.408	0.24	0.16	0.35	0.91	0.64	1.25	424.4	393.7	455.7
Thailand	M	45	0.197	0.095	0.349	0.23	0.15	0.34	0.63	0.43	0.88	428.8	393.2	464.7
Thailand	M	55	0.194	0.096	0.340	0.25	0.17	0.37	0.51	0.36	0.71	438.8	406.0	471.2
Thailand	M	65	0.197	0.098	0.351	0.29	0.19	0.42	0.42	0.29	0.59	450.2	414.1	485.6
Thailand	M	75	0.189	0.094	0.340	0.31	0.21	0.46	0.38	0.27	0.53	462.0	427.5	495.5
Thailand	M	90	0.177	0.089	0.319	0.34	0.24	0.50	0.35	0.25	0.48	475.9	439.2	511.2
Tajikistan	F	25	0.037	0.018	0.066	0.35	0.19	0.59	0.48	0.26	0.83	617.5	462.4	796.8
Tajikistan	F	35	0.030	0.015	0.053	0.33	0.18	0.56	0.36	0.20	0.61	621.1	466.7	790.5
Tajikistan	F	45	0.025	0.012	0.044	0.31	0.17	0.54	0.24	0.13	0.41	626.0	468.7	802.6
Tajikistan	F	55	0.024	0.012	0.042	0.34	0.19	0.59	0.19	0.11	0.32	640.8	482.5	818.8
Tajikistan	F	65	0.024	0.012	0.043	0.38	0.21	0.65	0.16	0.08	0.27	658.1	492.9	846.1
Tajikistan	F	75	0.023	0.012	0.041	0.42	0.23	0.71	0.14	0.08	0.24	675.6	509.5	866.3
Tajikistan	F	90	0.022	0.011	0.039	0.45	0.25	0.77	0.13	0.07	0.22	696.7	528.3	886.4
Tajikistan	M	25	0.031	0.014	0.055	0.32	0.18	0.54	0.54	0.29	0.91	560.3	435.4	724.1
Tajikistan	M	35	0.025	0.012	0.046	0.30	0.17	0.49	0.40	0.22	0.66	563.8	438.6	722.7
Tajikistan	M	45	0.020	0.010	0.038	0.28	0.15	0.47	0.27	0.15	0.45	568.6	442.2	723.2
Tajikistan	M	55	0.020	0.010	0.036	0.31	0.17	0.53	0.22	0.12	0.36	582.6	451.5	746.2
Tajikistan	M	65	0.020	0.009	0.036	0.34	0.19	0.59	0.18	0.09	0.30	599.0	463.4	776.4
Tajikistan	M	75	0.019	0.009	0.034	0.37	0.21	0.64	0.16	0.08	0.27	614.8	476.5	798.1
Tajikistan	M	90	0.019	0.009	0.033	0.40	0.23	0.69	0.15	0.08	0.25	632.9	490.6	813.7
Turkmenistan	F	25	0.022	0.011	0.039	0.51	0.27	0.87	0.23	0.12	0.39	666.6	516.1	854.7
Turkmenistan	F	35	0.018	0.009	0.032	0.48	0.25	0.80	0.17	0.09	0.28	670.6	521.3	849.4
Turkmenistan	F	45	0.015	0.007	0.026	0.45	0.24	0.76	0.12	0.06	0.20	676.0	523.4	860.5
Turkmenistan	F	55	0.014	0.007	0.025	0.50	0.27	0.84	0.09	0.05	0.16	692.1	537.2	880.2
Turkmenistan	F	65	0.014	0.007	0.026	0.55	0.30	0.94	0.08	0.04	0.13	710.7	545.5	905.2
Turkmenistan	F	75	0.014	0.007	0.025	0.60	0.33	1.01	0.07	0.04	0.11	729.7	565.2	922.2
Turkmenistan	F	90	0.013	0.007	0.024	0.65	0.35	1.10	0.06	0.03	0.11	752.2	581.3	951.5
Turkmenistan	M	25	0.018	0.009	0.032	0.46	0.26	0.78	0.26	0.14	0.45	609.7	460.0	779.1
Turkmenistan	M	35	0.015	0.007	0.027	0.43	0.25	0.72	0.19	0.11	0.33	613.6	469.9	768.6
Turkmenistan	M	45	0.012	0.006	0.022	0.41	0.23	0.68	0.13	0.07	0.23	618.7	470.4	777.6
Turkmenistan	M	55	0.011	0.006	0.021	0.45	0.25	0.73	0.10	0.06	0.18	634.0	485.6	803.5
Turkmenistan	M	65	0.011	0.006	0.020	0.50	0.28	0.81	0.08	0.05	0.14	651.7	495.7	831.8
Turkmenistan	M	75	0.011	0.006	0.019	0.55	0.31	0.87	0.08	0.04	0.13	668.9	512.7	848.1
Turkmenistan	M	90	0.011	0.005	0.020	0.59	0.33	0.95	0.07	0.04	0.12	688.2	530.1	867.1
Timor-Leste	F	25	0.008	0.004	0.015	0.21	0.12	0.35	0.41	0.23	0.71	530.1	405.5	667.5
Timor-Leste	F	35	0.007	0.003	0.012	0.20	0.12	0.33	0.31	0.17	0.53	534.7	408.7	665.9
Timor-Leste	F	45	0.005	0.003	0.010	0.20	0.11	0.33	0.22	0.12	0.37	540.2	411.4	675.9
Timor-Leste	F	55	0.005	0.002	0.010	0.22	0.13	0.36	0.18	0.10	0.30	552.4	419.8	692.4
Timor-Leste	F	65	0.005	0.002	0.010	0.25	0.14	0.41	0.15	0.08	0.25	566.3	429.5	712.5
Timor-Leste	F	75	0.005	0.002	0.009	0.27	0.15	0.44	0.13	0.07	0.23	582.0	441.5	728.8
Timor-Leste	F	90	0.005	0.002	0.009	0.29	0.16	0.48	0.12	0.07	0.21	600.3	457.4	751.8
Timor-Leste	M	25	0.006	0.003	0.011	0.20	0.11	0.34	0.47	0.25	0.84	484.4	375.6	615.6
Timor-Leste	M	35	0.005	0.002	0.010	0.19	0.11	0.31	0.35	0.19	0.63	488.6	378.5	614.4
Timor-Leste	M	45	0.004	0.002	0.008	0.18	0.10	0.30	0.25	0.13	0.43	493.6	382.5	624.7
Timor-Leste	M	55	0.004	0.002	0.008	0.20	0.11	0.34	0.20	0.11	0.34	505.2	392.3	636.9
Timor-Leste	M	65	0.004	0.002	0.008	0.23	0.12	0.38	0.16	0.09	0.28	518.3	402.1	651.9
Timor-Leste	M	75	0.004	0.002	0.008	0.25	0.14	0.41	0.15	0.08	0.25	531.7	414.9	665.6
Timor-Leste	M	90	0.004	0.002	0.007	0.27	0.15	0.45	0.14	0.08	0.23	546.9	424.5	686.9
Tonga	F	25	0.284	0.136	0.516	0.35	0.20	0.60	1.20	0.65	1.99	514.8	397.1	662.5
Tonga	F	35	0.236	0.116	0.422	0.33	0.19	0.56	0.89	0.49	1.48	517.7	396.9	657.8
Tonga	F	45	0.193	0.094	0.343	0.31	0.18	0.54	0.61	0.33	1.04	521.8	396.6	664.2
Tonga	F	55	0.185	0.093	0.335	0.34	0.19	0.58	0.48	0.26	0.81	535.0	411.3	686.9
Tonga	F	65	0.185	0.093	0.339	0.38	0.21	0.64	0.40	0.21	0.65	550.3	423.7	710.2
Tonga	F	75	0.181	0.093	0.328	0.42	0.23	0.68	0.36	0.20	0.59	564.9	435.2	727.9
Tonga	F	90	0.176	0.090	0.307	0.45	0.24	0.76	0.33	0.18	0.55	582.1	449.0	748.8
Tonga	M	25	0.235	0.111	0.417	0.33	0.18	0.56	1.31	0.70	2.29	476.1	366.3	619.5
Tonga	M	35	0.194	0.094	0.341	0.31	0.17	0.51	0.98	0.54	1.68	478.6	372.8	619.7
Tonga	M	45	0.157	0.076	0.279	0.30	0.16	0.49	0.67	0.36	1.17	482.2	376.5	623.8
Tonga	M	55	0.151	0.076	0.267	0.32	0.18	0.54	0.53	0.29	0.95	494.7	385.5	636.8
Tonga	M	65	0.151	0.074	0.273	0.36	0.19	0.61	0.44	0.23	0.79	509.2	394.7	659.4
Tonga	M	75	0.148	0.073	0.268	0.39	0.21	0.67	0.40	0.21	0.73	522.6	402.0	678.0
Tonga	M	90	0.144	0.072	0.248	0.42	0.23	0.69	0.37	0.20	0.65	538.0	416.9	700.7
Trinidad and Tobago	F	25	0.222	0.105	0.401	0.77	0.44	1.26	4.69	2.51	8.06	838.6	639.1	1077.8
Trinidad and Tobago	F	35	0.185	0.092	0.332	0.72	0.40	1.19	3.50	1.90	5.96	843.3	646.4	1080.2
Trinidad and Tobago	F	45	0.152	0.075	0.280	0.68	0.38	1.14	2.37	1.29	4.00	849.9	654.7	1089.9
Trinidad and Tobago	F	55	0.145	0.073	0.265	0.75	0.42	1.24	1.90	1.03	3.23	870.5	669.7	1105.5
Trinidad and Tobago	F	65	0.143	0.070	0.263	0.83	0.46	1.38	1.59	0.86	2.75	894.3	688.1	1136.8
Trinidad and Tobago	F	75	0.140	0.067	0.259	0.90	0.51	1.48	1.45	0.78	2.51	918.0	707.0	1166.2
Trinidad and Tobago	F	90	0.136	0.065	0.252	0.98	0.55	1.60	1.33	0.72	2.29	946.5	725.0	1208.7
Trinidad and Tobago	M	25	0.185	0.087	0.338	0.71	0.39	1.17	5.14	2.86	8.68	764.0	583.6	968.0
Trinidad and Tobago	M	35	0.154	0.072	0.285	0.66	0.37	1.08	3.81	2.14	6.38	768.5	587.6	973.2
Trinidad and Tobago	M	45	0.126	0.059	0.239	0.63	0.35	1.02	2.56	1.40	4.31	774.6	585.9	987.1
Trinidad and Tobago	M	55	0.121	0.057	0.224	0.69	0.38	1.11	2.05	1.16	3.39	793.1	605.7	1004.7
Trinidad and Tobago	M	65	0.121	0.057	0.222	0.77	0.41	1.25	1.71	0.97	2.84	814.6	626.9	1036.7
Trinidad and Tobago	M	75	0.118	0.056	0.217	0.83	0.45	1.35	1.56	0.89	2.56	836.3	645.0	1063.2
Trinidad and Tobago	M	90	0.113	0.054	0.209	0.90	0.49	1.47	1.44	0.82	2.34	861.9	661.4	1088.5
Tunisia	F	25	0.166	0.080	0.303	0.59	0.33	0.97	0.74	0.43	1.20	651.1	500.0	837.6
Tunisia	F	35	0.139	0.068	0.247	0.56	0.31	0.93	0.57	0.33	0.93	655.5	506.3	838.6
Tunisia	F	45	0.114	0.055	0.196	0.54	0.30	0.92	0.41	0.23	0.68	661.3	510.8	845.9
Tunisia	F	55	0.109	0.054	0.190	0.60	0.33	0.99	0.33	0.19	0.55	677.8	522.6	866.0
Tunisia	F	65	0.109	0.053	0.193	0.66	0.37	1.09	0.28	0.15	0.46	696.7	538.2	885.6
Tunisia	F	75	0.107	0.053	0.187	0.71	0.40	1.17	0.25	0.14	0.42	714.6	551.1	908.1
Tunisia	F	90	0.104	0.051	0.178	0.77	0.44	1.30	0.23	0.13	0.38	734.8	564.6	941.3
Tunisia	M	25	0.136	0.067	0.253	0.55	0.30	0.92	0.83	0.45	1.34	591.9	455.0	758.8
Tunisia	M	35	0.114	0.057	0.205	0.51	0.28	0.84	0.62	0.35	0.98	595.7	457.2	762.6
Tunisia	M	45	0.093	0.046	0.172	0.49	0.27	0.80	0.43	0.24	0.69	600.7	454.5	772.2
Tunisia	M	55	0.090	0.045	0.166	0.54	0.30	0.88	0.35	0.19	0.55	615.6	470.5	789.9
Tunisia	M	65	0.090	0.045	0.168	0.60	0.33	0.97	0.29	0.16	0.47	632.4	484.1	812.5
Tunisia	M	75	0.087	0.044	0.162	0.65	0.36	1.06	0.27	0.15	0.43	648.5	499.4	830.0
Tunisia	M	90	0.084	0.042	0.153	0.71	0.39	1.15	0.25	0.14	0.39	666.4	511.8	853.9
Turkey	F	25	0.130	0.065	0.232	0.42	0.33	0.53	0.68	0.52	0.88	669.4	606.8	740.0
Turkey	F	35	0.108	0.054	0.192	0.40	0.32	0.49	0.52	0.41	0.67	674.1	617.1	739.7
Turkey	F	45	0.089	0.042	0.161	0.39	0.30	0.49	0.37	0.28	0.49	680.1	617.4	748.1
Turkey	F	55	0.085	0.042	0.154	0.42	0.33	0.53	0.30	0.23	0.39	697.1	639.7	763.5
Turkey	F	65	0.085	0.041	0.155	0.47	0.37	0.58	0.25	0.19	0.32	716.3	655.9	785.7
Turkey	F	75	0.084	0.040	0.150	0.51	0.40	0.63	0.23	0.18	0.29	734.8	675.4	802.7
Turkey	F	90	0.081	0.040	0.145	0.55	0.43	0.68	0.21	0.16	0.27	755.1	693.9	822.4

Country	Sex	Midpoint of age range	Mean juice intake (servings/day)	Juice, lower uncertainty interval	Juice, upper uncertainty interval	Mean milk intake (servings/day)	Milk, lower uncertainty interval	Milk, upper uncertainty interval	Mean SSB intake (servings/day)	SSB, lower uncertainty interval	SSB, upper uncertainty interval	Mean calcium intake (servings/day)	Calcium, lower uncertainty interval	Calcium, upper uncertainty interval
Turkey	M	25	0.108	0.052	0.184	0.39	0.31	0.49	0.76	0.58	0.97	611.9	553.0	674.3
Turkey	M	35	0.090	0.045	0.152	0.36	0.29	0.45	0.57	0.45	0.71	615.7	560.8	672.6
Turkey	M	45	0.074	0.037	0.130	0.35	0.27	0.43	0.39	0.30	0.50	620.8	561.8	681.9
Turkey	M	55	0.071	0.036	0.123	0.38	0.30	0.47	0.32	0.25	0.40	636.2	580.4	692.8
Turkey	M	65	0.071	0.035	0.124	0.42	0.34	0.52	0.27	0.21	0.34	653.7	594.3	714.9
Turkey	M	75	0.069	0.035	0.119	0.46	0.37	0.57	0.24	0.19	0.31	670.3	614.1	728.5
Turkey	M	90	0.066	0.034	0.111	0.50	0.40	0.63	0.23	0.18	0.28	688.4	631.1	749.3
Taiwan	F	25	0.059	0.047	0.075	0.13	0.11	0.15	0.94	0.74	1.15	631.8	587.7	676.1
Taiwan	F	35	0.049	0.041	0.060	0.12	0.10	0.14	0.70	0.57	0.84	636.4	601.8	671.0
Taiwan	F	45	0.040	0.032	0.051	0.11	0.10	0.13	0.47	0.38	0.59	642.5	600.3	684.3
Taiwan	F	55	0.038	0.031	0.046	0.13	0.11	0.14	0.38	0.31	0.46	659.0	622.5	696.8
Taiwan	F	65	0.038	0.030	0.046	0.14	0.12	0.17	0.31	0.25	0.38	677.7	632.3	722.8
Taiwan	F	75	0.037	0.030	0.045	0.15	0.13	0.17	0.29	0.23	0.35	695.4	654.7	736.4
Taiwan	F	90	0.037	0.030	0.045	0.16	0.14	0.18	0.26	0.22	0.32	716.2	674.6	758.2
Taiwan	M	25	0.049	0.039	0.061	0.11	0.10	0.13	1.02	0.82	1.27	577.3	537.6	622.3
Taiwan	M	35	0.041	0.033	0.049	0.11	0.09	0.12	0.76	0.62	0.92	581.5	550.6	616.5
Taiwan	M	45	0.033	0.026	0.042	0.10	0.09	0.12	0.51	0.40	0.63	586.9	548.0	625.5
Taiwan	M	55	0.032	0.026	0.039	0.11	0.10	0.13	0.41	0.33	0.50	602.1	568.6	636.6
Taiwan	M	65	0.032	0.025	0.040	0.13	0.11	0.15	0.34	0.27	0.43	619.4	579.5	660.8
Taiwan	M	75	0.031	0.026	0.039	0.14	0.12	0.16	0.31	0.25	0.38	636.3	601.7	672.6
Taiwan	M	90	0.031	0.026	0.038	0.15	0.13	0.17	0.28	0.23	0.34	656.6	620.0	696.0
United Republic of Tanzania	F	25	0.065	0.033	0.121	0.24	0.13	0.43	0.56	0.31	1.01	413.1	318.8	537.3
United Republic of Tanzania	F	35	0.054	0.028	0.097	0.23	0.13	0.39	0.42	0.23	0.74	415.5	321.3	533.4
United Republic of Tanzania	F	45	0.044	0.023	0.078	0.21	0.12	0.38	0.29	0.15	0.51	418.9	322.8	539.6
United Republic of Tanzania	F	55	0.042	0.022	0.075	0.24	0.13	0.41	0.23	0.12	0.39	429.5	333.4	547.1
United Republic of Tanzania	F	65	0.042	0.021	0.077	0.26	0.14	0.46	0.19	0.10	0.32	441.6	340.7	563.6
United Republic of Tanzania	F	75	0.041	0.021	0.075	0.28	0.15	0.49	0.17	0.09	0.29	453.1	350.6	574.3
United Republic of Tanzania	F	90	0.040	0.020	0.072	0.31	0.17	0.54	0.16	0.08	0.27	466.1	360.5	592.8
United Republic of Tanzania	M	25	0.054	0.027	0.097	0.22	0.12	0.37	0.62	0.34	1.07	379.0	287.9	486.0
United Republic of Tanzania	M	35	0.045	0.022	0.080	0.20	0.11	0.34	0.47	0.26	0.78	381.5	293.8	487.4
United Republic of Tanzania	M	45	0.036	0.018	0.067	0.19	0.10	0.32	0.32	0.17	0.54	384.8	298.1	495.3
United Republic of Tanzania	M	55	0.035	0.017	0.062	0.21	0.12	0.35	0.25	0.14	0.42	394.3	303.4	506.8
United Republic of Tanzania	M	65	0.035	0.017	0.063	0.24	0.13	0.40	0.21	0.12	0.36	405.2	307.7	523.4
United Republic of Tanzania	M	75	0.034	0.017	0.061	0.26	0.14	0.42	0.19	0.11	0.32	415.7	315.7	533.0
United Republic of Tanzania	M	90	0.033	0.017	0.059	0.28	0.15	0.45	0.17	0.10	0.28	427.4	325.3	544.4
Uganda	F	25	0.258	0.110	0.543	0.22	0.12	0.38	0.48	0.26	0.83	405.6	309.6	526.7
Uganda	F	35	0.215	0.092	0.455	0.21	0.11	0.36	0.36	0.20	0.60	408.0	309.4	525.7
Uganda	F	45	0.175	0.072	0.366	0.20	0.10	0.35	0.24	0.14	0.41	411.4	307.9	533.6
Uganda	F	55	0.167	0.072	0.353	0.22	0.12	0.39	0.19	0.11	0.33	421.8	319.0	544.0
Uganda	F	65	0.167	0.071	0.352	0.24	0.13	0.43	0.16	0.08	0.27	433.7	326.4	558.6
Uganda	F	75	0.164	0.070	0.342	0.26	0.14	0.46	0.14	0.08	0.24	444.9	338.4	571.0
Uganda	F	90	0.159	0.069	0.336	0.29	0.15	0.49	0.13	0.07	0.23	457.6	348.9	585.0
Uganda	M	25	0.217	0.090	0.440	0.20	0.11	0.34	0.54	0.28	0.93	369.4	287.7	477.8
Uganda	M	35	0.179	0.075	0.363	0.19	0.11	0.32	0.40	0.21	0.68	371.8	290.2	479.3
Uganda	M	45	0.145	0.059	0.295	0.18	0.10	0.31	0.27	0.15	0.47	375.1	290.8	484.3
Uganda	M	55	0.139	0.056	0.278	0.20	0.11	0.33	0.22	0.12	0.37	384.3	300.7	499.5
Uganda	M	65	0.139	0.057	0.282	0.22	0.12	0.38	0.18	0.10	0.31	394.9	309.0	511.6
Uganda	M	75	0.136	0.057	0.277	0.24	0.14	0.40	0.16	0.09	0.28	405.1	319.3	522.6
Uganda	M	90	0.132	0.056	0.265	0.26	0.15	0.43	0.15	0.08	0.26	416.5	327.5	537.0
Ukraine	F	25	0.105	0.050	0.194	0.54	0.37	0.79	0.79	0.42	1.35	754.6	582.7	955.8
Ukraine	F	35	0.086	0.042	0.157	0.52	0.36	0.74	0.59	0.32	1.02	760.9	592.7	958.3
Ukraine	F	45	0.069	0.033	0.128	0.50	0.34	0.72	0.40	0.22	0.71	768.5	601.5	970.7
Ukraine	F	55	0.067	0.033	0.123	0.55	0.38	0.77	0.32	0.18	0.55	786.2	613.4	994.5
Ukraine	F	65	0.067	0.033	0.125	0.61	0.42	0.87	0.26	0.15	0.45	806.3	623.4	1030.8
Ukraine	F	75	0.066	0.032	0.120	0.66	0.45	0.93	0.24	0.13	0.40	826.7	640.7	1047.4
Ukraine	F	90	0.063	0.032	0.115	0.71	0.48	1.01	0.22	0.12	0.37	852.2	659.4	1068.1
Ukraine	M	25	0.087	0.045	0.156	0.49	0.33	0.71	0.87	0.45	1.53	688.6	529.2	867.3
Ukraine	M	35	0.072	0.037	0.128	0.47	0.31	0.66	0.65	0.35	1.10	691.9	532.8	870.8
Ukraine	M	45	0.058	0.028	0.106	0.45	0.30	0.63	0.44	0.24	0.76	697.0	537.4	886.2
Ukraine	M	55	0.056	0.028	0.099	0.50	0.34	0.70	0.35	0.19	0.60	715.4	553.9	901.5
Ukraine	M	65	0.056	0.028	0.100	0.57	0.38	0.80	0.29	0.16	0.49	736.7	568.1	928.4
Ukraine	M	75	0.055	0.028	0.098	0.61	0.41	0.85	0.26	0.15	0.45	756.4	583.1	954.8
Ukraine	M	90	0.053	0.028	0.092	0.65	0.44	0.91	0.24	0.13	0.42	779.5	600.2	976.1
Uruguay	F	25	0.432	0.213	0.795	0.50	0.26	0.86	0.99	0.53	1.68	537.5	418.8	680.4
Uruguay	F	35	0.359	0.178	0.644	0.47	0.25	0.77	0.74	0.40	1.25	540.7	417.4	680.5
Uruguay	F	45	0.292	0.143	0.532	0.45	0.24	0.74	0.52	0.28	0.88	545.1	419.8	687.2
Uruguay	F	55	0.280	0.138	0.500	0.49	0.26	0.82	0.41	0.22	0.69	558.6	430.7	701.0
Uruguay	F	65	0.279	0.137	0.506	0.55	0.30	0.93	0.33	0.18	0.56	574.1	439.4	723.4
Uruguay	F	75	0.274	0.136	0.498	0.60	0.32	1.00	0.30	0.16	0.51	589.2	452.8	742.4
Uruguay	F	90	0.265	0.134	0.491	0.65	0.34	1.07	0.27	0.15	0.47	607.8	466.1	767.1
Uruguay	M	25	0.356	0.172	0.647	0.45	0.25	0.75	1.08	0.62	1.80	495.1	378.4	624.6
Uruguay	M	35	0.295	0.145	0.525	0.43	0.23	0.69	0.81	0.46	1.33	497.9	382.6	622.6
Uruguay	M	45	0.240	0.115	0.425	0.41	0.22	0.67	0.55	0.31	0.92	501.8	388.2	623.8
Uruguay	M	55	0.230	0.116	0.398	0.45	0.24	0.72	0.44	0.25	0.72	514.3	398.3	641.3
Uruguay	M	65	0.230	0.113	0.397	0.50	0.27	0.82	0.36	0.20	0.58	528.6	405.6	668.8
Uruguay	M	75	0.226	0.112	0.382	0.54	0.30	0.88	0.33	0.18	0.53	542.7	417.2	683.3
Uruguay	M	90	0.219	0.110	0.386	0.58	0.32	0.94	0.30	0.17	0.49	559.4	428.3	697.0
United States of America	F	25	0.519	0.438	0.617	0.69	0.60	0.79	1.89	1.60	2.21	883.6	827.5	938.5
United States of America	F	35	0.429	0.373	0.492	0.64	0.57	0.71	1.43	1.25	1.62	888.0	841.9	931.4
United States of America	F	45	0.348	0.291	0.411	0.60	0.52	0.69	0.99	0.83	1.15	894.4	837.4	951.9
United States of America	F	55	0.343	0.298	0.395	0.65	0.58	0.73	0.78	0.67	0.90	915.7	871.2	962.6
United States of America	F	65	0.353	0.300	0.413	0.72	0.62	0.83	0.63	0.54	0.73	940.3	887.0	995.3
United States of America	F	75	0.352	0.307	0.407	0.78	0.70	0.88	0.57	0.49	0.65	964.7	919.1	1012.9
United States of America	F	90	0.348	0.303	0.401	0.86	0.77	0.97	0.52	0.45	0.59	995.7	944.1	1045.0
United States of America	M	25	0.441	0.372	0.519	0.65	0.56	0.74	2.17	1.83	2.55	809.0	759.3	861.0
United States of America	M	35	0.368	0.319	0.421	0.60	0.53	0.67	1.65	1.44	1.88	812.7	773.8	855.4
United States of America	M	45	0.303	0.252	0.358	0.56	0.49	0.65	1.16	0.99	1.36	818.1	768.6	870.4
United States of America	M	55	0.297	0.257	0.341	0.61	0.54	0.68	0.91	0.79	1.05	837.5	797.4	878.9
United States of America	M	65	0.302	0.255	0.351	0.68	0.59	0.78	0.72	0.61	0.84	860.0	813.2	911.7
United States of America	M	75	0.297	0.258	0.341	0.74	0.66	0.82	0.64	0.55	0.74	883.2	839.4	929.0
United States of America	M	90	0.289	0.250	0.332	0.82	0.73	0.91	0.57	0.49	0.65	912.0	869.7	956.5
Uzbekistan	F	25	0.033	0.016	0.060	0.56	0.30	0.95	0.23	0.12	0.40	682.3	521.0	876.1
Uzbekistan	F	35	0.027	0.014	0.048	0.52	0.29	0.88	0.17	0.09	0.29	686.6	527.6	877.8
Uzbekistan	F	45	0.022	0.011	0.040	0.49	0.27	0.83	0.12	0.06	0.20	692.2	528.4	891.7
Uzbekistan	F	55	0.021	0.011	0.038	0.54	0.30	0.91	0.09	0.05	0.15	708.6	543.9	905.1
Uzbekistan	F	65	0.021	0.011	0.038	0.60	0.33	1.03	0.08	0.04	0.13	727.6	555.2	924.9
Uzbekistan	F	75	0.021	0.011	0.037	0.65	0.36	1.11	0.07	0.04	0.11	747.0	574.5	946.6
Uzbekistan	F	90	0.020	0.010	0.035	0.71	0.40	1.21	0.06	0.03	0.10	770.3	594.6	976.5
Uzbekistan	M	25	0.027	0.013	0.047	0.51	0.28	0.85	0.26	0.14	0.45	622.8	478.8	786.4

Country	Sex	Midpoint of age range	Mean juice intake (servings/day)	Juice, lower uncertainty interval	Juice, upper uncertainty interval	Mean milk intake (servings/day)	Milk, lower uncertainty interval	Milk, upper uncertainty interval	Mean SSB intake (servings/day)	SSB, lower uncertainty interval	SSB, upper uncertainty interval	Mean calcium intake (servings/day)	Calcium, lower uncertainty interval	Calcium, upper uncertainty interval
Uzbekistan	M	35	0.022	0.011	0.039	0.48	0.27	0.77	0.20	0.10	0.33	626.6	487.1	783.4
Uzbekistan	M	45	0.018	0.009	0.033	0.45	0.26	0.75	0.13	0.07	0.23	631.8	491.3	796.5
Uzbekistan	M	55	0.017	0.009	0.031	0.50	0.28	0.81	0.11	0.06	0.18	647.5	506.1	809.9
Uzbekistan	M	65	0.017	0.009	0.031	0.55	0.31	0.91	0.09	0.04	0.15	665.7	515.5	835.9
Uzbekistan	M	75	0.017	0.009	0.030	0.60	0.33	0.98	0.08	0.04	0.13	683.3	534.2	852.8
Uzbekistan	M	90	0.016	0.008	0.029	0.65	0.36	1.07	0.07	0.04	0.12	703.5	554.8	888.9
Saint Vincent and the Grenadines	F	25	0.386	0.197	0.697	0.58	0.31	1.00	4.05	2.19	6.81	789.0	601.9	1008.0
Saint Vincent and the Grenadines	F	35	0.323	0.168	0.570	0.55	0.29	0.94	3.02	1.67	5.01	793.5	602.9	1008.0
Saint Vincent and the Grenadines	F	45	0.265	0.137	0.470	0.52	0.28	0.88	2.05	1.13	3.42	799.7	606.4	1017.3
Saint Vincent and the Grenadines	F	55	0.252	0.131	0.451	0.57	0.31	0.97	1.64	0.92	2.75	819.2	623.9	1043.7
Saint Vincent and the Grenadines	F	65	0.250	0.125	0.449	0.63	0.35	1.08	1.37	0.77	2.37	841.7	641.3	1071.2
Saint Vincent and the Grenadines	F	75	0.244	0.121	0.440	0.69	0.38	1.17	1.25	0.70	2.14	864.0	659.2	1100.3
Saint Vincent and the Grenadines	F	90	0.238	0.119	0.425	0.75	0.41	1.27	1.15	0.63	1.96	890.6	675.0	1128.1
Saint Vincent and the Grenadines	M	25	0.317	0.157	0.558	0.53	0.30	0.93	4.46	2.54	7.50	719.6	553.7	919.8
Saint Vincent and the Grenadines	M	35	0.264	0.135	0.470	0.50	0.28	0.86	3.31	1.87	5.57	723.8	555.5	925.5
Saint Vincent and the Grenadines	M	45	0.216	0.111	0.392	0.47	0.26	0.79	2.22	1.25	3.70	729.5	559.7	943.2
Saint Vincent and the Grenadines	M	55	0.208	0.106	0.372	0.52	0.29	0.88	1.78	1.00	3.00	747.0	571.0	955.5
Saint Vincent and the Grenadines	M	65	0.207	0.104	0.366	0.58	0.33	0.98	1.49	0.83	2.55	767.5	584.7	974.3
Saint Vincent and the Grenadines	M	75	0.202	0.101	0.351	0.62	0.35	1.04	1.36	0.77	2.29	788.0	601.7	1000.5
Saint Vincent and the Grenadines	M	90	0.194	0.097	0.334	0.67	0.38	1.13	1.25	0.72	2.10	812.0	625.0	1032.8
Venezuela (Bolivarian Republic of)	F	25	0.559	0.288	1.014	0.90	0.48	1.51	3.00	1.64	5.07	699.4	535.5	895.2
Venezuela (Bolivarian Republic of)	F	35	0.463	0.240	0.832	0.85	0.46	1.44	2.34	1.30	3.91	704.6	539.4	891.0
Venezuela (Bolivarian Republic of)	F	45	0.375	0.194	0.663	0.81	0.44	1.39	1.71	0.93	2.81	711.3	543.9	901.7
Venezuela (Bolivarian Republic of)	F	55	0.360	0.189	0.623	0.88	0.48	1.50	1.37	0.75	2.23	728.9	561.9	931.1
Venezuela (Bolivarian Republic of)	F	65	0.360	0.186	0.634	0.98	0.52	1.65	1.10	0.61	1.84	748.8	580.3	956.8
Venezuela (Bolivarian Republic of)	F	75	0.353	0.186	0.613	1.06	0.58	1.80	0.99	0.54	1.64	767.6	596.9	979.5
Venezuela (Bolivarian Republic of)	F	90	0.342	0.180	0.589	1.15	0.63	1.98	0.89	0.48	1.48	789.7	610.8	995.9
Venezuela (Bolivarian Republic of)	M	25	0.475	0.244	0.839	0.83	0.44	1.38	3.27	1.76	5.60	643.9	501.1	802.6
Venezuela (Bolivarian Republic of)	M	35	0.392	0.205	0.687	0.78	0.43	1.27	2.54	1.40	4.22	647.6	509.7	809.4
Venezuela (Bolivarian Republic of)	M	45	0.317	0.162	0.547	0.74	0.40	1.23	1.86	1.02	3.10	652.7	509.4	817.5
Venezuela (Bolivarian Republic of)	M	55	0.305	0.160	0.523	0.82	0.45	1.35	1.49	0.82	2.52	669.5	527.9	838.9
Venezuela (Bolivarian Republic of)	M	65	0.306	0.160	0.530	0.91	0.49	1.53	1.21	0.65	2.10	688.8	539.5	867.1
Venezuela (Bolivarian Republic of)	M	75	0.300	0.160	0.513	0.99	0.53	1.65	1.09	0.59	1.87	706.9	553.7	883.6
Venezuela (Bolivarian Republic of)	M	90	0.292	0.153	0.496	1.07	0.59	1.80	0.99	0.53	1.64	727.9	569.7	918.1
Viet Nam	F	25	0.033	0.016	0.058	0.16	0.08	0.28	0.42	0.23	0.72	488.8	378.4	613.9
Viet Nam	F	35	0.028	0.014	0.048	0.15	0.08	0.26	0.32	0.18	0.54	493.0	382.7	616.0
Viet Nam	F	45	0.023	0.012	0.040	0.15	0.08	0.24	0.22	0.12	0.37	498.0	386.6	624.4
Viet Nam	F	55	0.022	0.011	0.040	0.16	0.09	0.27	0.18	0.10	0.30	509.3	395.3	637.7
Viet Nam	F	65	0.022	0.011	0.040	0.18	0.10	0.31	0.15	0.08	0.24	522.2	403.0	654.0
Viet Nam	F	75	0.022	0.011	0.038	0.20	0.11	0.34	0.13	0.07	0.22	536.8	420.8	671.7
Viet Nam	F	90	0.021	0.010	0.036	0.22	0.11	0.36	0.12	0.07	0.21	554.3	439.5	698.7
Viet Nam	M	25	0.026	0.013	0.047	0.14	0.08	0.25	0.46	0.26	0.78	448.5	343.1	584.7
Viet Nam	M	35	0.022	0.011	0.040	0.13	0.07	0.23	0.35	0.20	0.57	452.4	346.6	593.6
Viet Nam	M	45	0.019	0.009	0.034	0.13	0.07	0.22	0.24	0.13	0.41	457.1	348.7	602.6
Viet Nam	M	55	0.018	0.009	0.034	0.14	0.08	0.25	0.20	0.11	0.33	467.7	356.2	609.7
Viet Nam	M	65	0.019	0.009	0.034	0.16	0.09	0.28	0.16	0.09	0.27	479.8	369.7	618.3
Viet Nam	M	75	0.018	0.009	0.033	0.17	0.10	0.30	0.15	0.08	0.24	492.4	377.1	637.2
Viet Nam	M	90	0.017	0.008	0.031	0.19	0.11	0.33	0.13	0.08	0.22	507.2	382.5	662.6
Vanuatu	F	25	0.257	0.123	0.465	0.27	0.15	0.46	0.74	0.42	1.26	483.5	376.1	603.6
Vanuatu	F	35	0.214	0.105	0.380	0.26	0.14	0.44	0.55	0.32	0.92	486.2	381.0	604.3
Vanuatu	F	45	0.174	0.085	0.313	0.24	0.13	0.43	0.38	0.21	0.64	490.1	379.3	613.0
Vanuatu	F	55	0.168	0.082	0.296	0.27	0.14	0.47	0.30	0.17	0.51	502.5	387.7	624.6
Vanuatu	F	65	0.168	0.082	0.298	0.30	0.16	0.53	0.25	0.13	0.42	516.8	397.1	645.8
Vanuatu	F	75	0.164	0.082	0.290	0.32	0.18	0.58	0.23	0.12	0.38	530.3	409.1	660.6
Vanuatu	F	90	0.159	0.079	0.282	0.35	0.19	0.62	0.21	0.12	0.34	545.8	420.8	686.1
Vanuatu	M	25	0.210	0.105	0.376	0.25	0.13	0.42	0.81	0.44	1.36	448.3	338.9	577.3
Vanuatu	M	35	0.174	0.088	0.311	0.24	0.12	0.39	0.61	0.34	1.01	450.5	346.1	582.4
Vanuatu	M	45	0.140	0.070	0.255	0.23	0.12	0.38	0.42	0.23	0.70	453.9	347.3	583.9
Vanuatu	M	55	0.135	0.069	0.246	0.25	0.14	0.41	0.33	0.18	0.55	465.7	360.3	600.1
Vanuatu	M	65	0.135	0.068	0.252	0.28	0.15	0.46	0.27	0.15	0.46	479.3	369.5	621.0
Vanuatu	M	75	0.132	0.068	0.242	0.30	0.16	0.50	0.25	0.14	0.41	491.9	378.2	634.6
Vanuatu	M	90	0.128	0.066	0.235	0.33	0.17	0.54	0.23	0.13	0.38	506.2	387.2	649.9
Samoa	F	25	0.608	0.274	1.180	0.25	0.13	0.43	0.87	0.46	1.48	472.6	436.5	510.9
Samoa	F	35	0.504	0.233	0.950	0.23	0.13	0.40	0.65	0.35	1.08	475.3	445.9	506.8
Samoa	F	45	0.411	0.190	0.771	0.22	0.12	0.38	0.44	0.24	0.72	479.0	442.3	518.7
Samoa	F	55	0.395	0.187	0.730	0.24	0.13	0.41	0.35	0.19	0.57	491.2	459.2	523.1
Samoa	F	65	0.396	0.184	0.738	0.27	0.15	0.45	0.29	0.16	0.48	505.1	467.4	543.2
Samoa	F	75	0.387	0.182	0.711	0.29	0.16	0.50	0.26	0.14	0.43	518.5	484.2	551.7
Samoa	F	90	0.375	0.175	0.699	0.32	0.18	0.54	0.24	0.13	0.40	534.3	502.4	568.9
Samoa	M	25	0.499	0.234	0.935	0.23	0.12	0.39	0.95	0.54	1.62	433.4	400.5	467.5
Samoa	M	35	0.411	0.196	0.779	0.21	0.12	0.35	0.71	0.41	1.19	435.7	408.9	463.7

Country	Sex	Midpoint of age range	Mean juice intake (servings/day)	Juice, lower uncertainty interval	Juice, upper uncertainty interval	Mean milk intake (servings/day)	Milk, lower uncertainty interval	Milk, upper uncertainty interval	Mean SSB intake (servings/day)	SSB, lower uncertainty interval	SSB, upper uncertainty interval	Mean calcium intake (servings/day)	Calcium, lower uncertainty interval	Calcium, upper uncertainty interval
Samoa	M	45	0.333	0.154	0.653	0.20	0.11	0.34	0.49	0.27	0.83	439.0	406.0	471.8
Samoa	M	55	0.320	0.152	0.600	0.22	0.12	0.37	0.39	0.22	0.65	450.5	421.7	478.0
Samoa	M	65	0.320	0.151	0.581	0.25	0.14	0.41	0.32	0.18	0.54	463.6	431.3	497.7
Samoa	M	75	0.314	0.148	0.569	0.27	0.15	0.44	0.29	0.17	0.48	475.8	446.0	505.8
Samoa	M	90	0.305	0.145	0.562	0.29	0.16	0.48	0.26	0.16	0.44	489.7	458.0	521.0
Yemen	F	25	0.159	0.080	0.283	0.32	0.18	0.53	0.81	0.44	1.38	564.6	433.3	725.1
Yemen	F	35	0.133	0.066	0.234	0.30	0.17	0.50	0.62	0.35	1.05	568.6	439.5	725.4
Yemen	F	45	0.109	0.053	0.195	0.29	0.16	0.49	0.44	0.24	0.74	573.7	442.2	730.2
Yemen	F	55	0.105	0.051	0.190	0.32	0.18	0.53	0.36	0.20	0.61	588.0	454.7	752.8
Yemen	F	65	0.105	0.051	0.196	0.35	0.19	0.59	0.30	0.17	0.51	604.2	466.0	778.4
Yemen	F	75	0.103	0.050	0.189	0.38	0.21	0.64	0.27	0.15	0.46	619.7	477.5	790.2
Yemen	F	90	0.100	0.048	0.177	0.41	0.23	0.70	0.25	0.14	0.42	637.3	488.7	811.2
Yemen	M	25	0.126	0.065	0.225	0.29	0.16	0.48	0.93	0.50	1.57	513.7	390.2	661.0
Yemen	M	35	0.106	0.054	0.182	0.27	0.15	0.44	0.70	0.38	1.17	517.0	391.8	657.0
Yemen	M	45	0.086	0.042	0.150	0.25	0.14	0.42	0.48	0.25	0.81	521.3	395.5	662.3
Yemen	M	55	0.083	0.042	0.146	0.28	0.16	0.45	0.39	0.21	0.64	534.2	408.0	681.5
Yemen	M	65	0.083	0.043	0.150	0.31	0.17	0.51	0.33	0.17	0.55	549.0	418.5	706.8
Yemen	M	75	0.081	0.042	0.144	0.34	0.19	0.56	0.30	0.16	0.50	562.8	430.7	721.1
Yemen	M	90	0.078	0.040	0.138	0.37	0.21	0.62	0.28	0.15	0.47	578.3	445.2	738.2
South Africa	F	25	0.031	0.025	0.037	0.67	0.57	0.77	1.07	0.90	1.27	460.5	429.9	488.3
South Africa	F	35	0.025	0.022	0.029	0.63	0.56	0.70	0.80	0.70	0.92	463.8	443.6	483.6
South Africa	F	45	0.021	0.017	0.025	0.60	0.52	0.69	0.55	0.46	0.65	468.0	439.1	496.7
South Africa	F	55	0.020	0.017	0.023	0.65	0.59	0.73	0.44	0.38	0.50	478.7	458.0	499.3
South Africa	F	65	0.020	0.017	0.023	0.73	0.63	0.83	0.36	0.31	0.42	491.0	464.2	521.5
South Africa	F	75	0.019	0.017	0.022	0.79	0.71	0.87	0.33	0.28	0.37	503.9	481.6	527.1
South Africa	F	90	0.019	0.017	0.021	0.86	0.78	0.95	0.30	0.27	0.34	519.1	497.2	542.3
South Africa	M	25	0.025	0.021	0.030	0.61	0.52	0.70	1.19	0.99	1.40	421.6	395.6	451.0
South Africa	M	35	0.021	0.018	0.024	0.57	0.51	0.63	0.89	0.78	1.01	424.3	405.6	443.9
South Africa	M	45	0.017	0.014	0.020	0.54	0.47	0.62	0.61	0.51	0.72	427.8	402.6	455.5
South Africa	M	55	0.016	0.014	0.019	0.59	0.53	0.66	0.48	0.42	0.55	437.9	418.6	457.9
South Africa	M	65	0.016	0.014	0.019	0.66	0.58	0.75	0.40	0.33	0.46	449.5	425.1	475.4
South Africa	M	75	0.016	0.014	0.018	0.72	0.65	0.79	0.36	0.31	0.41	461.5	442.3	483.5
South Africa	M	90	0.016	0.014	0.018	0.78	0.71	0.85	0.33	0.29	0.37	475.1	454.8	496.4
Zambia	F	25	0.004	0.002	0.007	0.12	0.06	0.21	0.69	0.36	1.16	350.7	272.9	453.9
Zambia	F	35	0.003	0.001	0.006	0.11	0.06	0.19	0.51	0.28	0.86	352.7	273.7	451.0
Zambia	F	45	0.003	0.001	0.005	0.11	0.06	0.19	0.35	0.19	0.59	355.5	273.8	453.0
Zambia	F	55	0.002	0.001	0.005	0.12	0.06	0.20	0.28	0.15	0.46	364.6	281.2	465.7
Zambia	F	65	0.002	0.001	0.005	0.13	0.07	0.22	0.23	0.12	0.38	374.9	288.5	483.9
Zambia	F	75	0.002	0.001	0.005	0.14	0.08	0.24	0.21	0.11	0.34	384.6	297.6	491.4
Zambia	F	90	0.002	0.001	0.004	0.15	0.08	0.27	0.19	0.10	0.32	395.6	307.8	499.3
Zambia	M	25	0.003	0.001	0.006	0.11	0.06	0.18	0.77	0.43	1.35	320.3	242.1	411.9
Zambia	M	35	0.003	0.001	0.005	0.10	0.05	0.17	0.58	0.32	1.00	322.5	243.9	414.2
Zambia	M	45	0.002	0.001	0.004	0.09	0.05	0.16	0.39	0.21	0.67	325.2	246.2	414.7
Zambia	M	55	0.002	0.001	0.004	0.10	0.06	0.17	0.31	0.17	0.53	333.3	252.2	424.3
Zambia	M	65	0.002	0.001	0.004	0.12	0.06	0.20	0.26	0.14	0.45	342.5	258.5	439.0
Zambia	M	75	0.002	0.001	0.004	0.13	0.07	0.21	0.23	0.13	0.40	351.3	265.2	448.3
Zambia	M	90	0.002	0.001	0.004	0.14	0.07	0.23	0.22	0.12	0.37	361.2	273.6	461.3
Zimbabwe	F	25	0.020	0.010	0.035	0.56	0.31	0.93	1.37	0.74	2.33	439.3	339.3	550.9
Zimbabwe	F	35	0.016	0.008	0.029	0.53	0.29	0.89	1.03	0.56	1.73	442.4	344.2	551.7
Zimbabwe	F	45	0.013	0.006	0.024	0.51	0.28	0.85	0.70	0.38	1.20	446.5	348.6	557.8
Zimbabwe	F	55	0.013	0.006	0.022	0.55	0.31	0.93	0.55	0.30	0.94	456.8	354.5	569.9
Zimbabwe	F	65	0.013	0.006	0.022	0.61	0.34	1.04	0.45	0.24	0.75	468.5	363.3	585.4
Zimbabwe	F	75	0.012	0.006	0.022	0.66	0.37	1.12	0.41	0.22	0.69	480.8	372.9	599.0
Zimbabwe	F	90	0.012	0.006	0.021	0.72	0.39	1.20	0.38	0.21	0.63	495.4	382.3	614.4
Zimbabwe	M	25	0.017	0.008	0.032	0.51	0.27	0.83	1.52	0.86	2.62	403.2	315.4	511.1
Zimbabwe	M	35	0.014	0.007	0.026	0.47	0.26	0.79	1.14	0.64	1.94	405.7	318.7	510.7
Zimbabwe	M	45	0.011	0.006	0.021	0.45	0.25	0.76	0.77	0.42	1.33	409.0	320.4	516.2
Zimbabwe	M	55	0.011	0.005	0.020	0.49	0.28	0.83	0.61	0.33	1.04	418.8	327.7	527.0
Zimbabwe	M	65	0.011	0.005	0.020	0.55	0.30	0.91	0.50	0.28	0.85	429.9	337.8	545.6
Zimbabwe	M	75	0.011	0.005	0.019	0.60	0.33	0.99	0.46	0.25	0.77	441.4	346.4	559.6
Zimbabwe	M	90	0.010	0.005	0.018	0.65	0.36	1.07	0.42	0.24	0.71	454.7	355.1	580.2

Table C. Regional consumption of SSBs, fruit juice, milk,and calcium in 2010.

	Mean (95%UI) of beverage intakes			
	SSB (servings/day)	Fruit Juice (servings/day)	Milk (servings/day)	Calcium (mg/day)
		**Asia, Central**		
**Women**				
Ages 20 to 39	0.42 (0.22,0.73)	0.09 (0.04,0.16)	0.52 (0.32,0.80)	677 (518,866)
Ages 40 to 59	0.22 (0.11,0.38)	0.06 (0.03,0.11)	0.50 (0.31,0.77)	692 (530,884)
Ages 60 and older	0.15 (0.08,0.25)	0.06 (0.03,0.11)	0.63 (0.39,0.98)	740 (567,942)
Overall	0.25 (0.13,0.42)	0.07 (0.03,0.12)	0.56 (0.35,0.87)	708 (543,903)
**Men**				
Ages 20 to 39	0.47 (0.25,0.80)	0.07 (0.04,0.13)	0.47 (0.29,0.73)	616 (474,781)
Ages 40 to 59	0.24 (0.13,0.42)	0.05 (0.03,0.10)	0.46 (0.28,0.70)	631 (487,798)
Ages 60 and older	0.16 (0.09,0.28)	0.05 (0.02,0.09)	0.58 (0.36,0.88)	674 (520,858)
Overall	0.27 (0.15,0.47)	0.06 (0.03,0.10)	0.51 (0.32,0.79)	645 (498,819)
**Both sexes overall**	0.26 (0.15,0.42)	0.06 (0.03,0.11)	0.54 (0.35,0.80)	678 (535,845)
		**Asia, East**		
**Women**				
Ages 20 to 39	0.33 (0.26,0.42)	0.02 (0.02,0.02)	0.08 (0.06,0.09)	445 (394,503)
Ages 40 to 59	0.17 (0.14,0.22)	0.01 (0.01,0.02)	0.07 (0.06,0.09)	456 (405,516)
Ages 60 and older	0.12 (0.09,0.15)	0.01 (0.01,0.02)	0.09 (0.07,0.11)	488 (434,552)
Overall	0.20 (0.15,0.25)	0.02 (0.01,0.02)	0.08 (0.07,0.10)	467 (414,528)
**Men**				
Ages 20 to 39	0.36 (0.28,0.46)	0.02 (0.01,0.02)	0.07 (0.05,0.08)	406 (359,460)
Ages 40 to 59	0.19 (0.15,0.24)	0.01 (0.01,0.01)	0.07 (0.05,0.08)	416 (369,469)
Ages 60 and older	0.13 (0.10,0.16)	0.01 (0.01,0.01)	0.09 (0.07,0.11)	446 (396,500)
Overall	0.21 (0.17,0.27)	0.01 (0.01,0.02)	0.07 (0.06,0.09)	426 (378,480)
**Both sexes overall**	0.20 (0.16,0.25)	0.01 (0.01,0.02)	0.08 (0.06,0.10)	447 (401,499)
		**Asia, South**		
**Women**				
Ages 20 to 39	0.44 (0.23,0.76)	0.02 (0.01,0.04)	0.29 (0.20,0.40)	463 (387,551)
Ages 40 to 59	0.23 (0.13,0.40)	0.02 (0.01,0.03)	0.29 (0.21,0.39)	474 (397,563)
Ages 60 and older	0.16 (0.08,0.27)	0.02 (0.01,0.03)	0.36 (0.26,0.50)	507 (424,604)
Overall	0.26 (0.14,0.45)	0.02 (0.01,0.03)	0.32 (0.23,0.44)	485 (406,577)
**Men**				
Ages 20 to 39	0.48 (0.26,0.81)	0.02 (0.01,0.04)	0.26 (0.19,0.37)	424 (351,508)
Ages 40 to 59	0.25 (0.14,0.43)	0.01 (0.01,0.03)	0.26 (0.19,0.36)	434 (360,521)
Ages 65 and older	0.17 (0.09,0.29)	0.01 (0.01,0.03)	0.33 (0.24,0.46)	464 (385,555)
Overall	0.28 (0.15,0.48)	0.02 (0.01,0.03)	0.29 (0.21,0.41)	444 (368,532)
**Both sexes overall**	0.27 (0.16,0.44)	0.02 (0.01,0.03)	0.31 (0.23,0.41)	464 (394,545)
		**Asia, Southeast**		
**Women**				
Ages 20 to 39	0.58 (0.34,0.94)	0.11 (0.07,0.19)	0.36 (0.25,0.52)	559 (458,673)
Ages 40 to 59	0.32 (0.19,0.51)	0.08 (0.05,0.14)	0.36 (0.25,0.52)	573 (471,692)
Ages 60 and older	0.21 (0.13,0.34)	0.08 (0.05,0.13)	0.47 (0.32,0.67)	612 (503,736)
Overall	0.35 (0.20,0.56)	0.09 (0.05,0.15)	0.41 (0.28,0.58)	586 (481,705)

**Men**				
Ages 20 to 39	0.64 (0.37,1.05)	0.09 (0.05,0.15)	0.34 (0.23,0.49)	512 (421,623)
Ages 40 to 59	0.35 (0.20,0.56)	0.07 (0.04,0.11)	0.33 (0.23,0.47)	525 (431,639)
Ages 60 and older	0.23 (0.14,0.37)	0.07 (0.04,0.11)	0.42 (0.29,0.61)	560 (460,679)
Overall	0.38 (0.22,0.62)	0.07 (0.04,0.12)	0.37 (0.26,0.53)	536 (441,651)
**Both sexes overall**	0.37 (0.23,0.57)	0.08 (0.05,0.13)	0.39 (0.28,0.54)	562 (470,667)
		**Asia-Pacific high-income**		
**Women**				
Ages 20 to 39	0.42 (0.31,0.57)	0.26 (0.15,0.41)	0.43 (0.32,0.58)	582 (536,631)
Ages 40 to 59	0.22 (0.16,0.30)	0.19 (0.11,0.30)	0.43 (0.31,0.58)	598 (551,646)
Ages 60 and older	0.15 (0.11,0.20)	0.18 (0.11,0.29)	0.53 (0.39,0.71)	638 (589,688)
Overall	0.25 (0.18,0.33)	0.20 (0.12,0.33)	0.47 (0.35,0.64)	610 (563,660)
**Men**				
Ages 20 to 39	0.49 (0.37,0.66)	0.21 (0.13,0.33)	0.39 (0.29,0.53)	531 (490,576)
Ages 40 to 59	0.27 (0.20,0.36)	0.15 (0.09,0.24)	0.37 (0.27,0.50)	545 (504,591)
Ages 60 and older	0.17 (0.13,0.23)	0.14 (0.09,0.23)	0.48 (0.36,0.65)	583 (539,632)
Overall	0.29 (0.22,0.39)	0.16 (0.10,0.26)	0.42 (0.31,0.57)	558 (515,604)
**Both sexes overall**	0.27 (0.21,0.35)	0.18 (0.12,0.28)	0.45 (0.34,0.59)	584 (544,628)
		**Australasia**		
**Women**				
Ages 20 to 39	0.59 (0.49,0.70)	0.92 (0.47,1.66)	0.59 (0.51,0.69)	696 (650,742)
Ages 40 to 59	0.31 (0.26,0.37)	0.67 (0.34,1.20)	0.58 (0.50,0.67)	714 (669,762)
Ages 60 and older	0.21 (0.18,0.25)	0.64 (0.33,1.14)	0.74 (0.64,0.85)	763 (715,813)
Overall	0.35 (0.29,0.41)	0.73 (0.37,1.31)	0.65 (0.56,0.75)	730 (683,778)
**Men**				
Ages 20 to 39	0.65 (0.54,0.77)	0.75 (0.37,1.35)	0.54 (0.46,0.63)	635 (592,680)
Ages 40 to 59	0.34 (0.29,0.41)	0.54 (0.27,0.98)	0.52 (0.45,0.60)	651 (608,697)
Ages 60 and older	0.23 (0.19,0.27)	0.52 (0.26,0.92)	0.67 (0.58,0.77)	698 (653,743)
Overall	0.38 (0.32,0.46)	0.59 (0.30,1.06)	0.59 (0.51,0.68)	667 (623,712)
**Both sexes overall**	0.37 (0.31,0.43)	0.66 (0.36,1.13)	0.62 (0.55,0.71)	699 (658,741)
		**Caribbean**		
**Women**				
Ages 20 to 39	3.15 (1.86,5.09)	0.35 (0.18,0.61)	0.70 (0.41,1.11)	820 (647,1026)
Ages 40 to 59	1.65 (0.97,2.66)	0.26 (0.14,0.44)	0.67 (0.39,1.07)	839 (663,1049)
Ages 60 and older	1.12 (0.66,1.80)	0.24 (0.13,0.42)	0.85 (0.50,1.35)	897 (710,1119)
Overall	1.85 (1.09,2.99)	0.28 (0.15,0.48)	0.75 (0.44,1.20)	858 (679,1072)
**Men**				
Ages 20 to 39	3.44 (2.03,5.57)	0.29 (0.15,0.49)	0.65 (0.38,1.04)	751 (595,937)
Ages 40 to 59	1.78 (1.05,2.86)	0.21 (0.11,0.36)	0.62 (0.37,1.00)	768 (610,959)
Ages 60 and older	1.21 (0.72,1.94)	0.20 (0.11,0.34)	0.78 (0.47,1.25)	821 (652,1024)
Overall	2.01 (1.19,3.24)	0.23 (0.12,0.39)	0.70 (0.42,1.12)	786 (624,981)
**Both sexes overall**	1.93 (1.20,2.98)	0.25 (0.14,0.42)	0.73 (0.45,1.11)	823 (667,1008)
		**Europe, Central**		
**Women**				
Ages 20 to 39	0.42 (0.27,0.63)	0.25 (0.15,0.39)	0.72 (0.50,1.00)	618 (512,745)
Ages 40 to 59	0.22 (0.14,0.33)	0.18 (0.11,0.28)	0.71 (0.50,0.99)	632 (523,761)

Ages 60 and older	0.15 (0.10,0.23)	0.17 (0.10,0.26)	0.91 (0.65,1.28)	677 (560,813)
Overall	0.25 (0.16,0.37)	0.19 (0.12,0.30)	0.80 (0.56,1.12)	647 (536,779)
**Men**				
Ages 20 to 39	0.49 (0.31,0.74)	0.20 (0.12,0.32)	0.66 (0.46,0.92)	568 (467,687)
Ages 40 to 59	0.25 (0.16,0.38)	0.14 (0.08,0.23)	0.64 (0.45,0.89)	577 (474,698)
Ages 60 and older	0.17 (0.11,0.25)	0.14 (0.08,0.22)	0.84 (0.59,1.16)	618 (509,748)
Overall	0.28 (0.18,0.43)	0.16 (0.09,0.25)	0.73 (0.51,1.02)	592 (487,716)
**Both sexes overall**	0.27 (0.18,0.39)	0.17 (0.11,0.27)	0.77 (0.56,1.04)	620 (522,736)
			**Europe, Eastern**	
**Women**				
Ages 20 to 39	0.56 (0.32,0.91)	0.13 (0.07,0.22)	0.71 (0.54,0.92)	766 (642,908)
Ages 40 to 59	0.29 (0.17,0.48)	0.09 (0.05,0.16)	0.70 (0.53,0.91)	786 (661,930)
Ages 60 and older	0.19 (0.11,0.32)	0.09 (0.05,0.15)	0.88 (0.68,1.14)	837 (706,988)
Overall	0.33 (0.19,0.53)	0.10 (0.06,0.17)	0.78 (0.60,1.01)	802 (675,949)
**Men**				
Ages 20 to 39	0.61 (0.35,0.99)	0.11 (0.06,0.18)	0.64 (0.49,0.83)	699 (587,830)
Ages 40 to 59	0.32 (0.18,0.52)	0.08 (0.04,0.13)	0.64 (0.49,0.82)	716 (601,850)
Ages 60 and older	0.21 (0.12,0.35)	0.07 (0.04,0.12)	0.82 (0.63,1.05)	768 (646,908)
Overall	0.36 (0.20,0.58)	0.08 (0.05,0.14)	0.72 (0.55,0.92)	733 (616,869)
**Both sexes overall**	0.34 (0.21,0.53)	0.09 (0.06,0.15)	0.75 (0.59,0.95)	770 (658,898)
			**Europe, Western**	
**Women**				
Ages 20 to 39	0.66 (0.48,0.88)	0.39 (0.29,0.53)	0.82 (0.64,1.06)	909 (814,1019)
Ages 40 to 59	0.31 (0.23,0.42)	0.27 (0.20,0.36)	0.79 (0.61,1.02)	931 (833,1043)
Ages 60 and older	0.22 (0.16,0.29)	0.27 (0.20,0.36)	0.98 (0.76,1.26)	991 (887,1109)
Overall	0.37 (0.27,0.50)	0.30 (0.22,0.41)	0.88 (0.68,1.13)	950 (850,1064)
**Men**				
Ages 20 to 39	0.76 (0.56,1.03)	0.32 (0.23,0.42)	0.77 (0.60,0.99)	836 (747,936)
Ages 40 to 59	0.34 (0.25,0.47)	0.22 (0.16,0.29)	0.70 (0.54,0.90)	851 (762,952)
Ages 60 and older	0.23 (0.17,0.31)	0.21 (0.16,0.29)	0.88 (0.68,1.13)	905 (811,1013)
Overall	0.42 (0.30,0.56)	0.24 (0.18,0.33)	0.80 (0.62,1.03)	870 (779,974)
**Both sexes overall**	0.39 (0.30,0.51)	0.27 (0.21,0.36)	0.84 (0.67,1.06)	911 (824,1009)
			**Latin America, Andean**	
**Women**				
Ages 20 to 39	1.38 (0.74,2.35)	0.48 (0.24,0.86)	0.50 (0.27,0.85)	639 (488,825)
Ages 40 to 59	0.72 (0.38,1.23)	0.35 (0.17,0.62)	0.48 (0.26,0.82)	655 (504,838)
Ages 60 and older	0.48 (0.26,0.82)	0.33 (0.17,0.60)	0.61 (0.33,1.03)	700 (538,900)
Overall	0.80 (0.43,1.37)	0.38 (0.19,0.68)	0.54 (0.29,0.92)	670 (514,861)
**Men**				
Ages 20 to 39	1.48 (0.82,2.46)	0.39 (0.19,0.72)	0.47 (0.25,0.79)	585 (449,754)
Ages 40 to 59	0.77 (0.42,1.31)	0.28 (0.14,0.53)	0.45 (0.24,0.77)	599 (462,769)
Ages 60 and older	0.51 (0.29,0.87)	0.27 (0.13,0.50)	0.57 (0.31,0.96)	640 (493,816)
Overall	0.86 (0.48,1.45)	0.31 (0.15,0.57)	0.51 (0.27,0.86)	613 (472,785)
**Both sexes overall**	0.83 (0.49,1.34)	0.35 (0.18,0.60)	0.52 (0.30,0.85)	642 (506,805)
			**Latin America, Central**	
**Women**				

Ages 20 to 39	2.52 (1.48,4.07)	0.50 (0.25,0.89)	1.02 (0.63,1.60)	730 (587,894)
Ages 40 to 59	1.45 (0.85,2.35)	0.36 (0.18,0.63)	0.99 (0.61,1.54)	748 (600,919)
Ages 60 and older	0.94 (0.55,1.51)	0.34 (0.18,0.61)	1.25 (0.77,1.94)	799 (645,980)
Overall	1.54 (0.90,2.48)	0.39 (0.20,0.70)	1.11 (0.68,1.73)	765 (615,938)
**Men**				
Ages 20 to 39	2.75 (1.62,4.41)	0.42 (0.21,0.74)	0.92 (0.57,1.44)	669 (543,819)
Ages 40 to 59	1.58 (0.93,2.54)	0.30 (0.15,0.53)	0.90 (0.55,1.40)	685 (558,839)
Ages 60 and older	1.03 (0.61,1.66)	0.29 (0.14,0.52)	1.14 (0.70,1.79)	734 (597,898)
Overall	1.68 (0.99,2.70)	0.33 (0.16,0.58)	1.01 (0.62,1.58)	702 (570,859)
**Both sexes overall**	1.61 (1.00,2.48)	0.36 (0.20,0.61)	1.06 (0.69,1.59)	734 (606,883)
		**Latin America, Southern**		
**Women**				
Ages 20 to 39	0.93 (0.60,1.39)	0.36 (0.18,0.65)	0.45 (0.30,0.66)	528 (439,630)
Ages 40 to 59	0.50 (0.32,0.75)	0.26 (0.13,0.47)	0.43 (0.29,0.64)	540 (449,644)
Ages 60 and older	0.32 (0.21,0.49)	0.25 (0.13,0.45)	0.55 (0.37,0.81)	578 (480,692)
Overall	0.55 (0.35,0.82)	0.29 (0.14,0.51)	0.49 (0.33,0.72)	553 (459,661)
**Men**				
Ages 20 to 39	1.02 (0.67,1.53)	0.30 (0.15,0.53)	0.41 (0.28,0.60)	487 (404,584)
Ages 40 to 59	0.53 (0.35,0.80)	0.22 (0.11,0.38)	0.40 (0.27,0.58)	499 (414,596)
Ages 60 and older	0.35 (0.23,0.52)	0.21 (0.11,0.36)	0.50 (0.34,0.74)	533 (443,639)
Overall	0.60 (0.39,0.89)	0.23 (0.12,0.42)	0.45 (0.30,0.66)	510 (423,611)
**Both sexes overall**	0.57 (0.39,0.82)	0.26 (0.14,0.45)	0.47 (0.33,0.67)	532 (450,627)
		**Latin America, Tropical**		
**Women**				
Ages 20 to 39	0.59 (0.43,0.82)	0.26 (0.20,0.34)	0.39 (0.29,0.53)	537 (461,629)
Ages 40 to 59	0.32 (0.23,0.44)	0.19 (0.14,0.24)	0.38 (0.28,0.51)	550 (471,644)
Ages 60 and older	0.21 (0.16,0.30)	0.18 (0.14,0.23)	0.48 (0.36,0.64)	589 (505,690)
Overall	0.35 (0.25,0.49)	0.20 (0.16,0.26)	0.43 (0.32,0.57)	563 (483,659)
**Men**				
Ages 20 to 39	0.65 (0.48,0.90)	0.21 (0.16,0.28)	0.36 (0.26,0.49)	491 (423,569)
Ages 40 to 59	0.35 (0.25,0.49)	0.15 (0.12,0.20)	0.34 (0.25,0.47)	503 (433,582)
Ages 60 and older	0.23 (0.17,0.32)	0.15 (0.11,0.19)	0.43 (0.32,0.59)	538 (465,621)
Overall	0.39 (0.28,0.53)	0.17 (0.13,0.22)	0.38 (0.29,0.53)	514 (444,595)
**Both sexes overall**	0.37 (0.28,0.49)	0.18 (0.15,0.24)	0.41 (0.31,0.54)	539 (470,618)
		**North Africa and Middle East**		
**Women**				
Ages 20 to 39	0.70 (0.41,1.14)	0.23 (0.12,0.41)	0.57 (0.34,0.89)	643 (518,791)
Ages 40 to 59	0.39 (0.23,0.64)	0.17 (0.09,0.30)	0.56 (0.34,0.88)	659 (531,812)
Ages 60 and older	0.27 (0.16,0.44)	0.16 (0.09,0.28)	0.70 (0.42,1.11)	704 (568,865)
Overall	0.43 (0.25,0.70)	0.19 (0.10,0.32)	0.62 (0.38,0.98)	673 (543,829)
**Men**				
Ages 20 to 39	0.78 (0.45,1.25)	0.19 (0.10,0.33)	0.51 (0.31,0.82)	588 (473,722)
Ages 40 to 59	0.42 (0.24,0.67)	0.14 (0.07,0.24)	0.50 (0.30,0.78)	602 (484,739)
Ages 60 and older	0.29 (0.17,0.46)	0.13 (0.07,0.23)	0.63 (0.38,1.00)	642 (517,788)
Overall	0.47 (0.27,0.75)	0.15 (0.08,0.26)	0.56 (0.34,0.89)	615 (495,755)
**Both sexes overall**	0.45 (0.28,0.70)	0.17 (0.10,0.28)	0.59 (0.37,0.90)	644 (530,777)

		**North America high-income**		
**Women**				
Ages 20 to 39	1.32 (1.14,1.53)	0.55 (0.47,0.65)	0.65 (0.57,0.74)	870 (818,921)
Ages 40 to 59	0.71 (0.60,0.82)	0.40 (0.34,0.47)	0.62 (0.54,0.70)	889 (839,941)
Ages 60 and older	0.46 (0.39,0.52)	0.41 (0.35,0.47)	0.77 (0.68,0.87)	950 (899,1002)
Overall	0.78 (0.67,0.89)	0.45 (0.38,0.52)	0.69 (0.61,0.78)	910 (859,962)
**Men**				
Ages 20 to 39	1.52 (1.31,1.77)	0.47 (0.40,0.55)	0.61 (0.54,0.69)	797 (750,845)
Ages 40 to 59	0.83 (0.71,0.96)	0.35 (0.30,0.41)	0.58 (0.51,0.65)	813 (767,862)
Ages 60 and older	0.51 (0.44,0.59)	0.34 (0.30,0.40)	0.73 (0.64,0.82)	870 (824,920)
Overall	0.89 (0.77,1.03)	0.38 (0.33,0.44)	0.65 (0.57,0.74)	833 (787,882)
**Both sexes overall**	0.83 (0.73,0.95)	0.42 (0.36,0.48)	0.67 (0.60,0.75)	872 (828,918)
		**Oceania**		
**Women**				
Ages 20 to 39	0.87 (0.47,1.48)	0.25 (0.12,0.45)	0.23 (0.12,0.38)	465 (368,581)
Ages 40 to 59	0.45 (0.25,0.77)	0.18 (0.09,0.33)	0.22 (0.12,0.37)	475 (375,597)
Ages 60 and older	0.30 (0.17,0.51)	0.17 (0.08,0.31)	0.28 (0.15,0.46)	509 (402,636)
Overall	0.51 (0.28,0.86)	0.19 (0.09,0.36)	0.24 (0.14,0.41)	487 (385,609)
**Men**				
Ages 20 to 44	0.95 (0.51,1.62)	0.20 (0.10,0.37)	0.21 (0.11,0.35)	426 (335,538)
Ages 40 to 59	0.50 (0.27,0.86)	0.14 (0.07,0.26)	0.20 (0.11,0.34)	436 (343,550)
Ages 60 and older	0.33 (0.18,0.57)	0.14 (0.07,0.25)	0.25 (0.14,0.43)	467 (368,588)
Overall	0.56 (0.30,0.95)	0.16 (0.08,0.29)	0.23 (0.12,0.38)	447 (351,563)
**Both sexes overall**	0.53 (0.31,0.87)	0.18 (0.09,0.31)	0.24 (0.14,0.38)	467 (377,574)
		**Sub-Saharan Africa, Central**		
**Women**				
Ages 20 to 39	0.78 (0.41,1.32)	0.25 (0.11,0.49)	0.61 (0.31,1.05)	492 (370,648)
Ages 40 to 59	0.41 (0.22,0.71)	0.18 (0.08,0.35)	0.58 (0.30,1.00)	504 (380,663)
Ages 60 and older	0.27 (0.14,0.46)	0.17 (0.08,0.34)	0.74 (0.38,1.26)	539 (409,705)
Overall	0.46 (0.24,0.78)	0.19 (0.09,0.39)	0.66 (0.34,1.13)	516 (389,677)
**Men**				
Ages 20 to 39	0.87 (0.46,1.52)	0.20 (0.09,0.37)	0.55 (0.29,0.96)	451 (340,590)
Ages 40 to 59	0.45 (0.24,0.80)	0.14 (0.07,0.27)	0.53 (0.28,0.92)	462 (349,605)
Ages 60 and older	0.30 (0.16,0.52)	0.14 (0.06,0.26)	0.67 (0.35,1.16)	494 (373,645)
Overall	0.51 (0.27,0.89)	0.15 (0.07,0.29)	0.60 (0.31,1.04)	473 (357,618)
**Both sexes overall**	0.48 (0.27,0.80)	0.17 (0.09,0.32)	0.63 (0.35,1.04)	495 (384,632)
		**Sub-Saharan Africa, East**		
**Women**				
Ages 20 to 39	0.59 (0.31,1.00)	0.07 (0.03,0.13)	0.32 (0.17,0.56)	440 (337,567)
Ages 40 to 59	0.31 (0.17,0.53)	0.05 (0.02,0.09)	0.31 (0.17,0.54)	451 (344,580)
Ages 60 and older	0.21 (0.11,0.35)	0.05 (0.02,0.09)	0.40 (0.21,0.68)	482 (370,618)
Overall	0.34 (0.18,0.59)	0.05 (0.02,0.10)	0.35 (0.19,0.61)	461 (353,592)
**Men**				
Ages 20 to 39	0.65 (0.35,1.10)	0.05 (0.02,0.10)	0.29 (0.16,0.50)	402 (309,517)
Ages 40 to 59	0.34 (0.18,0.58)	0.04 (0.02,0.08)	0.28 (0.15,0.48)	411 (316,528)
Ages 60 and older	0.23 (0.12,0.39)	0.04 (0.02,0.07)	0.36 (0.20,0.61)	439 (339,564)
Overall	0.38 (0.20,0.65)	0.04 (0.02,0.08)	0.32 (0.17,0.54)	421 (324,540)

**Both sexes overall**	0.36 (0.21,0.59)	0.05 (0.02,0.09)	0.33 (0.19,0.55)	441 (348,554)
		**Sub-Saharan Africa, Southern**		
**Women**				
Ages 20 to 39	1.04 (0.62,1.66)	0.04 (0.02,0.06)	0.73 (0.43,1.19)	469 (378,580)
Ages 40 to 59	0.54 (0.32,0.87)	0.03 (0.01,0.05)	0.71 (0.42,1.16)	481 (387,594)
Ages 60 and older	0.36 (0.22,0.57)	0.03 (0.01,0.04)	0.89 (0.53,1.45)	513 (413,632)
Overall	0.61 (0.36,0.97)	0.03 (0.02,0.05)	0.79 (0.47,1.29)	491 (396,607)
**Men**				
Ages 20 to 39	1.15 (0.69,1.86)	0.03 (0.02,0.05)	0.66 (0.39,1.05)	430 (343,535)
Ages 45 to 64	0.60 (0.36,0.96)	0.02 (0.01,0.04)	0.63 (0.38,1.01)	440 (353,546)
Ages 60 and older	0.40 (0.24,0.64)	0.02 (0.01,0.04)	0.80 (0.49,1.26)	470 (377,583)
Overall	0.67 (0.40,1.08)	0.02 (0.01,0.04)	0.71 (0.43,1.13)	450 (361,558)
**Both sexes overall**	0.64 (0.40,0.98)	0.03 (0.02,0.04)	0.75 (0.47,1.16)	471 (386,572)
		**Sub-Saharan Africa, West**		
**Women**				
Ages 20 to 39	0.68 (0.36,1.16)	0.05 (0.02,0.09)	0.33 (0.18,0.56)	465 (364,586)
Ages 40 to 59	0.35 (0.19,0.61)	0.03 (0.02,0.07)	0.32 (0.18,0.54)	476 (372,599)
Ages 60 and older	0.24 (0.13,0.40)	0.03 (0.02,0.06)	0.41 (0.23,0.68)	508 (398,641)
Overall	0.40 (0.21,0.68)	0.04 (0.02,0.07)	0.36 (0.20,0.60)	486 (381,613)
**Men**				
Ages 20 to 39	0.76 (0.40,1.31)	0.04 (0.02,0.08)	0.31 (0.17,0.52)	427 (332,541)
Ages 40 to 59	0.39 (0.21,0.68)	0.03 (0.01,0.05)	0.30 (0.16,0.50)	437 (341,552)
Ages 60 and older	0.26 (0.14,0.45)	0.03 (0.01,0.05)	0.38 (0.21,0.63)	467 (365,590)
Overall	0.44 (0.23,0.76)	0.03 (0.01,0.06)	0.34 (0.19,0.56)	447 (349,565)
**Both sexes overall**	0.42 (0.24,0.69)	0.03 (0.02,0.06)	0.35 (0.20,0.56)	467 (374,577)
